# Discovery of Potent Isoquinolinequinone *N*-Oxides to Overcome Cancer Multidrug Resistance

**DOI:** 10.1021/acs.jmedchem.4c00705

**Published:** 2024-08-02

**Authors:** Ryan D. Kruschel, Mélanie A. G. Barbosa, Maria João Almeida, Cristina P. R. Xavier, M. Helena Vasconcelos, Florence O. McCarthy

**Affiliations:** † School of Chemistry, Analytical and Biological Chemistry Research Facility, University College Cork, Cork T12 K8AF, Ireland; ‡ i3S−Instituto de Investigação e Inovação em Saúde, Universidade do Porto, 4200-135 Porto Portugal; § Cancer Drug Resistance Group, IPATIMUP−Institute of Molecular Pathology and Immunology, University of Porto, 4200-135 Porto Portugal; ∥ FFUP−Faculty of Pharmacy of the University of Porto, 4050-313 Porto Portugal

## Abstract

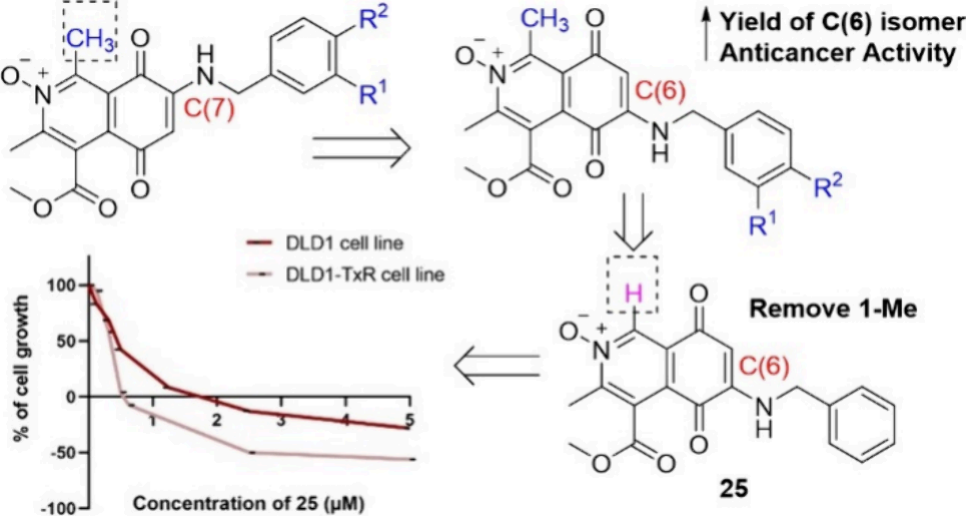

Multidrug resistance (MDR) of human tumors has resulted in an immediate need to develop appropriate new drugs. This work outlines the development of 20 potent IQQ *N*-oxide derivatives in two isomeric families, both exhibiting nanomolar GI_50_ against human tumor cell lines. Preliminary NCI-60 tumor screening sees the C(6) isomers achieve a mean GI_50_ > 2 times lower than the corresponding C(7) isomers. MDR evaluation of nine selected compounds reveals that each presents lower GI_50_ concentrations in two MDR tumor cell lines. Four of the series display nanomolar GI_50_ values against MDR cells, having selectivity ratios up to 2.7 versus the sensitive (parental) cells. The most potent compound **25** inhibits the activity of drug efflux pumps in MDR cells, causes significant ROS accumulation, and potently inhibits cell proliferation, causing alterations in the cell cycle profile. Our findings are confirmed by 3D spheroid models, providing new candidates for studies against MDR cancers.

## Introduction

Multidrug resistance (MDR) of human tumors reduces the clinical efficacy of current anticancer agents.^1−3^ Cancer is one of the leading causes of death worldwide and despite significant therapeutic innovations and advancements in clinical formulation, MDR is one of the major challenges in cancer treatment, being responsible for many cases of refractory cancer and tumor recurrence.^4−7^ One of the mechanisms responsible for MDR is the overexpression of ATP-binding cassette (ABC) drug efflux pumps, such as P-glycoprotein (P-gp), causing increased cellular drug efflux. Interestingly, it has been observed that cancer cells expressing high levels of drug efflux pumps show an unexpected hypersensitivity, called collateral sensitivity (CS), to some chemical compounds (e.g., NSC297366, NSC73306).^8−10^

For many years, nature has provided compounds which have been manipulated to generate potent anticancer agents.^11^ The isoquinolinequinone (IQQ) natural product metabolites, caulibugulones and mansouramycins were discovered from marine sponges in the early 2000s.^12,13^ The isoquinoline and the quinone moieties are well-established anticancer frameworks found in the structures of many broad-spectrum anticancer agents approved for clinical use including doxorubicin, daunorubicin, ellipticine, mitomycin C and mitoxantrone. Quinone is a soft electrophile, allowing it to participate in adduct formation *in vivo* to sulfur based nucleophiles including the antioxidant glutathione, amino acid cysteine and indeed DNA, which can result in cancer cell death.^14^ Additionally, redox cycling of the quinone moiety to the semiquinone radical occurs *in vivo* and is a mechanism of cytotoxicity through generation of reactive oxygen species (ROS) leading to cell cycle arrest and cancer cell death.^15^

**Figure 1:**
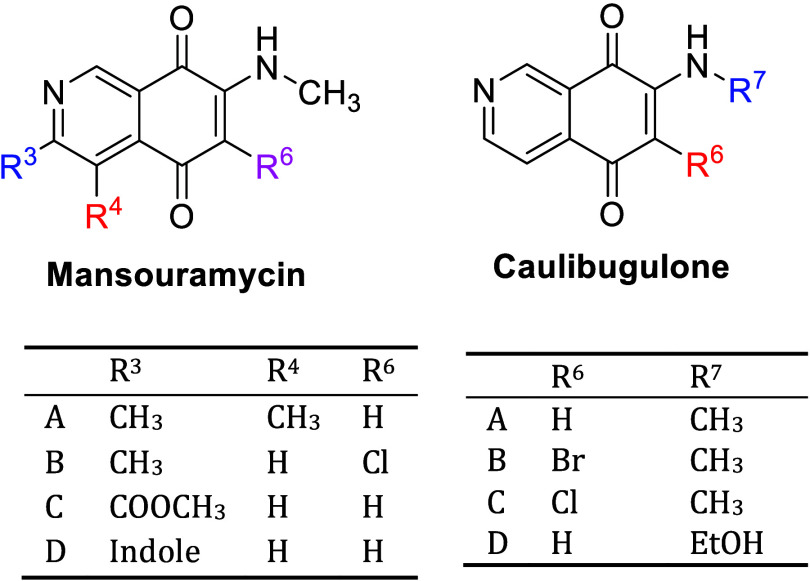
Structures of known cytotoxic isoquinolinequinone caulibugulones and mansouramycins metabolites.

The mansouramycins exhibit nM cytotoxicity across multiple cancer cell lines, including ovarian, breast, and melanoma. The most active analogue of the family is mansouramycin C (Figure 1, R^3^ = COOCH_3_), which was shown to selectively affect cancer cells in preference to normal human cells through redox cycling mediated ROS generation and subsequent cell death by opening of the mitochondrial permeability transition pore.^13,16^ The introduction of a chemical species which amplifies ROS, damages the cancer cell leading to cell death but affects normal cells minimally due to reduced basal ROS levels.^17^ Accordingly, MDR cells with higher basal ROS levels tend to exhibit increased sensitivity to ROS, a proposed mechanism of collateral sensitivity.^8^ The related caulibugulone series also exhibit sub μM effects against tumor cell lines, with mechanistic studies suggesting that caulibugulone A generates modest levels of ROS and irreversibly inhibits Cdc25b, leading to cell cycle arrest at the G1 and G2/M phases.^18^

**Figure 2:**
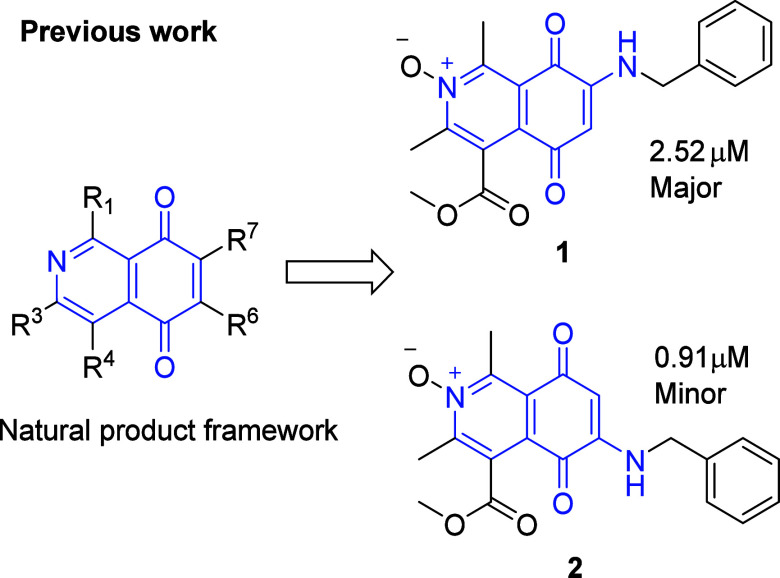
IQQ benzylamine *N*-oxides **1** and **2**.

ROS production is heavily entwined in the cytotoxicity of both mansouramycins and caulibugulones (and doxorubicin and quinones in general). Through chemical substitution the quinone can be made to more readily undergo redox cycling and be more susceptible to attack by biological nucleophiles.^19−21^ Delgado et al. identified that addition of an electron withdrawing bromine to the C(6) or C(7) site of IQQs results in more cytotoxic derivatives.^22−24^ Structural refinement of this allowed us to recently identify a potent IQQ *N*-oxide anticancer template.^25^ While scoping the effect of amine substitution on the IQQ framework, both C(6) and C(7) amino isomers were formed with the C(7) isomer as the major product (Figure 2).

The C(7) and C(6) benzylamine isomers, **1** and **2** exhibited a mean GI_50_ of 2.52 and 0.91 μM, respectively, against 53 human tumor cell lines. Although 6-substituted **2** was isolated as the minor product in 6% yield, it exhibited a 2.8-fold more potent average GI_50_ than **1**, possessing nanomolar activity across melanoma, ovarian and leukemia tumor cell lines. An electrochemical study identified the ability of both isomers to undergo redox cycling *in vitro* and biological adduct formation to cysteine and glutathione *in vitro* was identified.^19−21^ In terms of sensitivity, **1** and **2** exhibited notable growth inhibition against the MDR cell line NCI/ADR-RES (2.67 μM and 535 nM respectively), which expresses high levels of P-gp. The C(6) isomer **2** exhibited a GI_50_ on the NCI/ADR-RES cells 40 times greater than that of doxorubicin. This led us to hypothesize that isomer **2** exerts a collateral sensitivity effect in MDR cells. Thus, compounds **1** and **2** were chosen as leads to expand the marine metabolite based isoquinolinequinones, to exploit their activity on MDR cells.^25^

**Figure 3:**
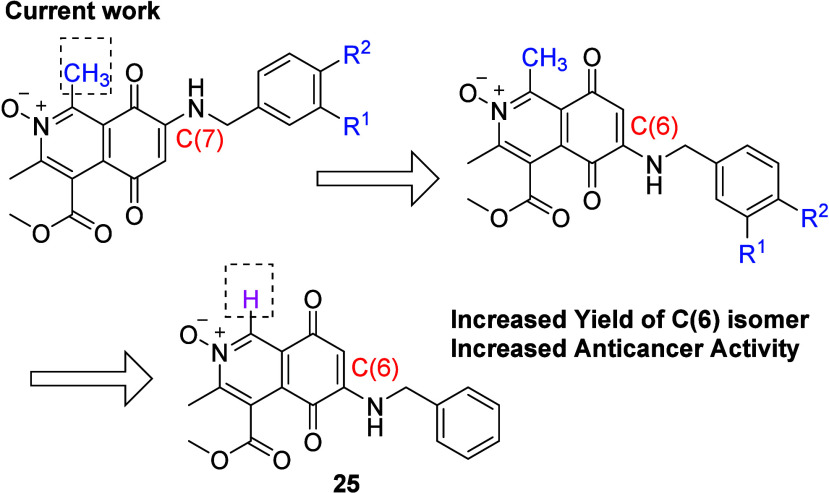
This exploration of diverse benzylamines in an IQQ *N*-oxide scaffold to probe anticancer activity on sensitive and MDR cells and regiocontrol of quinone substitution.

We outline the exploration of the IQQ *N*-oxide framework to probe the effects against sensitive and MDR counterpart tumor cell lines (Figure 3). Evaluation of the compounds by the National Cancer Institute 60 Cell line (NCI60) screen was used to identify potential lead anticancer agents. To further confirm their antitumor activity and investigate their possible collateral sensitivity effect, a selected panel was evaluated against two pairs of drug sensitive and their MDR (P-gp-overexpressing) counterpart human tumor cell lines. Mechanistic studies were performed to test the nature of their effect on MDR tumor cells cultured in 2D and in 3D spheroid models.

## Results and Discussion

### Chemical Synthesis of IQQ *N*-Oxides

To date much effort has been conducted to explore the chemical space around the IQQ framework, primarily encompassing the amination, halogenation and thiolation at the C(6) and C(7) positions, ring extension and dimer formation.^22−24,26−34^ Regioselective control remains problematic with amination at the C(7) position predominant in literature.^32,35^ Recently, in tandem with our own work, the use of the *N*-oxide to drive regiocontrol in C(6) oxidative amination was reported on route to ellipticine and isocaulibugulones.^36,37^

**Scheme 1:**
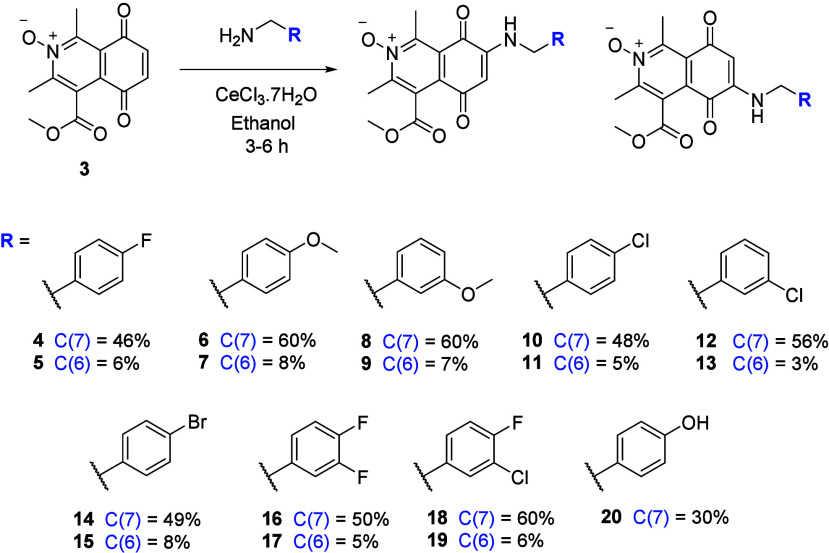
Amination of IQQ *N*-Oxide with Benzylamines Isolated yields quoted.

The IQQ *N*-oxide framework **3** was synthesized using a one-pot oxidation/Michael addition followed by an *m*CPBA oxidation to furnish the *N*-oxide as previously described.^25^ A library of 17 IQQ *N*-oxide analogues was built to probe the effect of electronic and steric properties of the benzylamine unit on cancer cell growth as our primary evaluation (Scheme 1). Benzylamine additions to the framework resulted in a mixture of C(6) and C(7) isomers, as previously noted when using cerium chloride, C(7) was the main isomer in all cases after isolation by chromatography.^25^ Isolated yields ranged from 46−61% for the C(7) isomers and 3−10% for the C(6) isomers. In the case of amination with 4-hydroxybenzylamine, the isolation of the sole C(7) isomer **20** in 30% yield was seen.

### NCI60 Antitumor Screening

Initially, screening compounds for cell growth inhibition at 10 μM identified that 16 out of the 17 IQQ *N*-oxides potently reduced the growth of 54 of 59 tumor cell lines (Table 1, mean growth % 10 μM, and see Supporting Information (SI), Figures S4−S52 and Tables S2−S17). Only the C(7) *p*-Cl **10** did not achieve sufficient growth inhibition at the initial screen to progress to further testing. The 16 active compounds were then assayed against the full NCI60 tumor cell line panel (57−60 cell lines) at five concentrations (ranging from 0.01 to 100 μM). The concentration−response curve generated enables the determination of three criteria, GI_50_ (drug concentration at which 50% of cell growth is inhibited), total growth inhibition (TGI) and LC_50_ (drug concentration at which 50% of tumor cells are killed) which can be used to compare effectiveness against any compound tested in the screen since its inception. In general, the IQQ *N*-oxide series exhibited GI_50_ values in the low micromolar to nanomolar range across multiple tumor cell lines (Table 1). As can be seen, the C(6) isomers are significantly more potent than the C(7) isomers, with the mean GI_50_ values of the C(6) benzylamine isomers being from 2 to 3.1 times lower than those of their C(7) counterparts. Literature reports confirm that the C(6) aminated IQQ isomers often possess better anticancer activity and are associated with problematic synthesis.^22,23^

**Table 1:** IQQ *N*-Oxide Analogue NCI60 Mean Growth, Mean GI_50_ and GI_50_ Values in Eight Selected Tumour Cell Lines

	one dose^a^	five dose (μM)^b^								
			breast	leu	colon	melanoma		ovarian		
entry	mean growth (%, 10 μM)	mean GI_50_	MCF-7	K-562	COLO-205	UACC-62	LOX-IMVI	OVCAR-3	OVCAR-8	NCI-ADR/RES
C(7) Regioisomers										
**4**	18	1.54	1.11	2.43	0.376	1.25	1.94	0.319	1.59	1.68
**6**	19	2.12	1.41	2.67	0.839	1.72	2.41	0.315	2.78	2.42
**8**	23	2.29	1.65	2.98	1.10	1.70	2.36	0.512	2.52	2.36
10*	57									
**12**	12	1.48	1.10	2.17	0.388	1.24	1.76	0.322	1.52	1.64
**14**	18	1.80	1.33	2.38	0.659	1.17	1.67	0.301	1.83	1.97
**16**	11	1.91	1.48	2.23	0.673	1.53	1.98	0.333	1.96	2.30
**18**	4	1.51	1.32	2.67	0.588	1.38	2.34	0.306	2.38	2.08
**20**	−21	1.58	0.416	1.63	0.579	1.36	0.938	0.266	1.08	2.20
C(6) Regioisomers										
**5**	−37	0.76	0.296	0.352	0.260	0.229	0.226	0.240	0.406	0.487
**7**	−34	0.90	0.300	0.491	0.325	0.374	0.408	0.284	0.452	0.419
**9**	−52	0.74	0.286	0.416	0.252	0.306	0.302	0.259	0.339	0.441
**11**	−32	0.55	0.267	0.058	0.238	0.055	0.039	0.208	0.292	0.267
**13**	−42	0.62	0.270	0.109	0.263	0.144	0.094	0.243	0.297	0.352
**15**	−50	0.78	0.289	0.047	0.313	0.046	0.084	0.273	0.465	0.439
**17**	−49	0.58	0.290	0.113	0.260	0.068	0.064	0.258	0.299	0.345
**19**	−47	0.56	0.229	0.051	0.211	0.041	0.031	0.199	0.249	0.321

a Mean cell growth % represents the mean cell growth of 60 tumor cell lines incubated with IQQ analogue at 10 μM concentration.

b All GI_50_ values are quoted as μM concentration. Mean GI_50_ values were calculated based on 52 human tumor cell lines common to all analogues. *This compound did not attain activity threshold to progress to Five Dose testing.

All C(6) substituted compounds perform better than our starting reference IQQ **2**. The MDR ovarian cancer cell line NCI-ADR/RES is responsive to the full panel of compounds, and again, the C(6) isomers exhibit 3−8 times more potency against NCI-ADR/RES relative to their corresponding C(7) isomers. This doxorubicin resistant cell line is derived from the same individual and shares a number of karyotypic abnormalities with OVCAR-8, for which similar potency was observed.^38,39^ This result suggests the increased P-gp expression of NCI-ADR/RES cells does not affect the activity of the IQQ *N*-oxide series and confirms our previous findings.^25^

Many cell lines in the NCI60 cell panel harbor mutations or/and chromosomal abnormalities. For example, the leukemia cell line K-562 harbors the Philadelphia chromosome translocation (BCR-ABL) and is responsive to tyrosine kinase inhibitors imatinib and lapatinib.^40,41^ There is a significant difference in the response of K-562 cells to the regioisomers, with C(6) halogenated benzylamine IQQ analogues **11**, **13**, **15**, **17**, and **19**, exhibiting GI_50_ values of <150 nM against K-562 cells, up to 50-fold more potent GI_50_ values than their C(7) isomers pointing to excellent potency and the potential of this compound class.^42^

Lipophilicity and steric bulk were investigated through substituted aromatic moieties and a pattern in mean GI_50_ values is evident.^43,44^ The methoxy IQQ analogues **6**−**9**, possess notable nanomolar GI_50_’s in the selected cell lines, but were the least active in this series. The *p*-F analogues **4**−**5**, **16**−**19** are consistently more potent against the NCI-60 panel and merit further investigation. The activity of *p*-OH **20** in the screen suggests future work to probe H-bonding moieties and indeed its corresponding 6-isomer. Overall, we can conclude that the C(6) substituted panel of compounds have pronounced anticancer activity across a broad panel of cells including those with mutations/resistance mechanisms. However, synthesis of C(6) substituted IQQs is a limiting factor, so we next set out to address this.

### Improving the Yield of the C(6) Benzylamine Isomer

**Figure 4:**
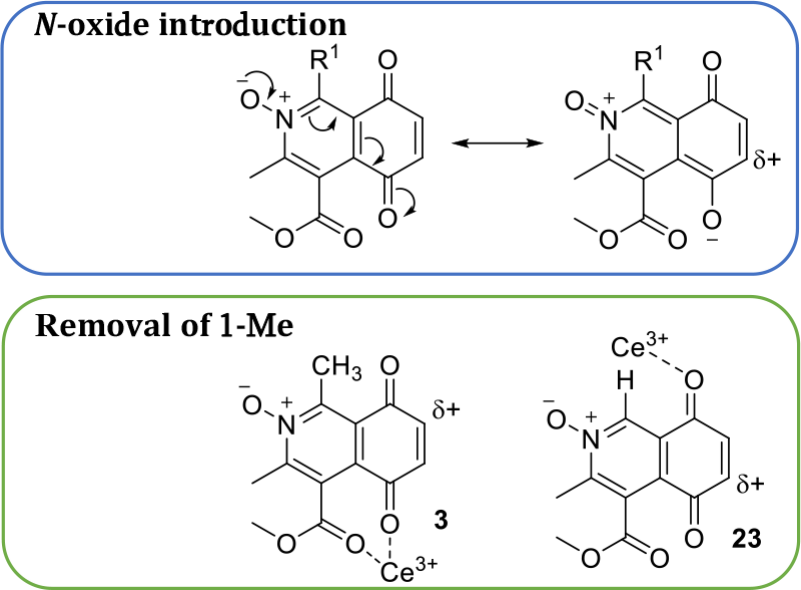
Proposed cerium coordination to C(8) carbonyl oxygen to promote oxidative amination at C(6) position.

**Scheme 2:**
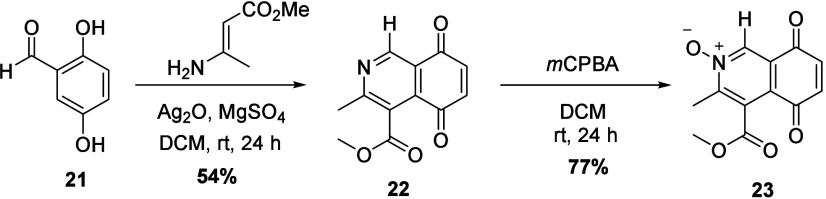
Synthesis of 1-H IQQ *N*-Oxide **23**

**Scheme 3:**
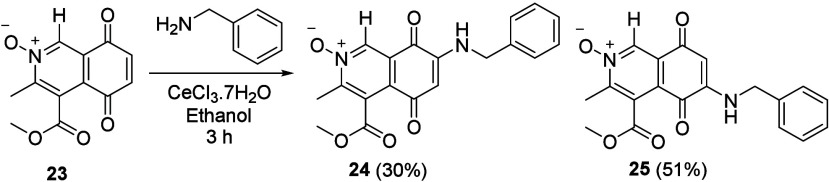
Amination of **23** with Benzylamine

The C(6) IQQ *N*-oxide isomers present a better anticancer NCI screening profile in comparison to their C(7) counterparts. Although a direct molecular target has not been defined, the ability of IQQ compounds to induce ROS has been linked to redox potentials where the C(6) isomers are more effective and so a robust route to C(6) substitution is desirable.^22,23,25^ At the outset of this work, it was reasoned that the presence of the *N*-oxide moiety would result in a resonance directing effect to promote nucleophilic attack at C(6) (Figure 4) and should prevent cerium coordination to nitrogen which promotes C(7) addition.^36,37^ However, as evidenced in Scheme 1, C(7) products dominate despite the influence of the *N*-oxide.^25^ Changing tack, it was postulated that the removal of the C(1) methyl group of **3** would allow more effective Lewis acid coordination to the C(8)carbonyl which directs nucleophilic addition to the C(6) site.^35^ Literature provides evidence that replacement of IQQ C(1) methyl group with hydrogen maintains significant antitumor activity.^22,23,26^ This hypothesis was tested commencing from the C(1)H IQQ *N*-oxide **23**, synthesized utilizing the same methodology as for **3** (Scheme 2). As a direct comparison of lead compounds **1** and **2**, benzylamine was reacted with **23** under the same conditions. Pleasingly, this resulted in formation of the C(6) aminated IQQ *N*-oxide **25** in a ratio of 1.7:1 in comparison to the C(7) isomer **24** (Scheme 3, isolated yields).

Antitumor screening was performed and the removal of the C(1) methyl moiety afforded the most potent compound of the series, **25** (Table 2). This analogue exhibited a mean cell growth of −71% at 10 μM concentration and this translated into ≤200 nM GI_50_ values in 31/60 cell lines, possessing an average GI_50_ of 210 nM. The C(7) isomer **24** exhibits a mean GI_50_ of 0.69 μM. which outperforms the related IQQ *N*-oxides reported in Table 1 by between 2.2- and 3.3-fold and the parent **1**, **2**, and **3** (mean GI_50_ of 2.52, 0.91, and 1.75 μM. respectively) and intriguingly **23** is less potent than **3**.

**Table 2:** NCI60 Mean Cell Growth (%), Mean GI_50_ and Selected Cell GI_50_ Values of C1(H) IQQ *N*-Oxide Analogues

		**23**	**24**	**25**
mean cell growth (one dose, %)^a^		44.5	−35.1	−70.6
mean GI_50_ (five dose, μM)^b^		2.15	0.69	0.21
Selected Cell Line GI_50_				
breast	MCF-7	1.65	0.20	0.15
leukemia	K-562	2.51	0.62	0.25
colon	COLO-205	1.85	0.33	0.16

melanoma	UACC-62	1.81	0.35	0.16
	LOX-IMVI	1.96	0.30	0.19

ovarian	OVCAR-3	0.32	0.27	0.048
	OVCAR-8	2.93	0.32	0.24
	NCI-ADR/RES	3.27	0.31	0.23

a Mean cell growth % represents the mean cell growth of 60 cancer cell lines incubated with IQQ analogue at 10 μM concentration.

b All GI_50_ values are quoted as μM concentration. Mean GI_50_ values were calculated based on 52 human tumor cell lines common to all analogues.

Given the increase in potency, tumor cell line selectivity is less evident (SI, Figures S53−S61 and Tables S18−S20). The C(6) isomer **25** was 2−3 times more effective against the tumor cell lines K-562, UACC-62 and LOX-IMVI, however, both isomers exhibited nanomolar activity unlike the C(1)CH_3_ isomeric series listed in Table 1. Particularly low GI_50_ values were noted for ovarian tumor cell lines OVCAR-3 (48 nM) and OVCAR-4 (69 nM) and melanoma tumor cell line MALME-3M (48 nM). Evidence of diverging cell line response is seen at 1 μM in both the ovarian and renal dose/response curves (SI, Figure S60).

The mean GI_50_ for **25** across the tumor cell line panel is lower than 83% of the FDA’s approved anticancer agents and performed better than 86% of these in terms of mean LC_50_.^45^

Of keen importance to this study, **24** and **25** exhibit nanomolar GI_50_ values against the MDR ovarian cell line NCI-ADR/RES, at 310 and 227 nM. respectively. In addition, the NSCLC (GI_50_ 174−397 nM) and colon (GI_50_ 157−247 nM) cell line panels show a consistent response to treatment with **25**.

### Effect of IQQ *N*-Oxides on the Growth of NSCLC and Colorectal Cancer Sensitive and MDR Counterpart Cell Lines

Taking into consideration the promising results of preliminary NCI screening, nine compounds (**1**, **2**, **3**, **4**, **5**, **20**, **23**, **24**, **25**) were selected for further screening in two counterpart pairs of sensitive and MDR cell lines. Compounds **1**−**3** and **23−25** were chosen as a direct structural comparison of the 1-position substituent (H/CH_3_) and 6/7 substitution; **20** was selected based on being the most effective C7-isomer; **4**−**5** were selected as a representative pair of C6/C7-isomers with consistent potency. Two distinct pairs of counterpart drug-sensitive and MDR human tumor cell lines were used, namely the non-small lung cancer (NSCLC) NCI-H460 (same cell line as in the NCI screen) and its MDR counterpart cell line NCI-H460/R and the colorectal cancer DLD1 and its MDR counterpart cell line DLD1-TxR (Table 3). The screening was performed using the SRB assay, and the GI_50_ for each compound was determined by interpolation on the attained concentration−response curves and compared with NCI60 screening data (SI, Figures S62−S63; Table S21).

**Table 3:** GI_50_ Concentrations (μM) of Selected Compounds and Positive Controls (Doxorubicin or Paclitaxel) Determined in NSCLC (NCI-H460 and NCI-H460/R) and Colorectal Cancer (DLD1 and DLD1-TxR) Counterpart Cell Lines with the SRB Assay

	NSCLC			colorectal cancer		
compd	NCI-H460	NCI-H460/R	selectivity ratio^a^	DLD1	DLD1-TxR	selectivity ratio^a^
**1**	3.06 ± 0.15	2.29 ± 0.28	1.3	4.89 ± 0.51	2.97 ± 0.13	1.7
**2**	1.43 ± 0.13	0.83 ± 0.02	1.7	2.33 ± 0.38	0.88 ± 0.09	2.7
**3**	8.43 ± 1.41	4.28 ± 1.08	2	6.62 ± 0.35	4.80 ± 0.07	1.4
**4**	2.70 ± 0.30	2.25 ± 0.27	1.2	5.94 ± 0.30	3.13 ± 0.10	1.9
**5**	0.56 ± 0.20	0.36 ± 0.11	1.6	1.22 ± 0.14	0.67 ± 0.05	1.8
**20**	6.02 ± 0.34	4.84 ± 0.74	1.2	9.67 ± 1.15	5.93 ± 0.55	1.6
**23**	38.02 ± 8.32	17.90 ± 3.36	2.1	30.23 ± 4.08	14.87 ± 0.68	2
**24**	0.75 ± 0.14	0.38 ± 0.02	2	1.39 ± 0.17	0.72 ± 0.03	1.9
**25**	0.35 ± 0.02	0.34 ± 0.03	1	0.61 ± 0.11	0.42 ± 0.03	1.5
doxorubicin	0.03 ± 0.01	2.46 ± 0.04	0.01			
paclitaxel				0.02 ± 0.003	0.97 ± 0.21	0.02

a Selectivity ratio = GI_50_ from the sensitive (parental cell line)/GI_50_ from the MDR cell line.

Remarkably, all nine synthesized compounds presented lower GI_50_ concentrations in both MDR tumor cell lines when compared to their respective drug-sensitive counterparts (corresponding to a selectivity ratio above 1 in most cases). It was also confirmed that NCI-H460/R and DLD1-TxR MDR cell lines were resistant to doxorubicin and paclitaxel, respectively, when compared with their sensitive counterpart cells, confirming cell line resistance to conventional chemotherapeutic drugs used in treatment of cancers. It is important to note the use of multiple compounds and two MDR counterpart cell lines with different resistance origins allows confidence in our results.

Where a direct structural comparison can be made (**1**/**2**, **4**/**5**, **24**/**25**) the C(6) isomer was more potent in each case which again points to the importance of substitution. Four compounds, **2**, **5**, **24**, and **25** exhibited GI_50_ concentrations in a nanomolar range against both MDR tumor cell lines and selectivity ratios up to 2.7 evident. Compound **25** was selected for in depth evaluation having the lowest GI_50_ among the tested IQQs, which is in agreement with the results obtained in the NCI60 antitumor screening (Table 2).

Initially, the antitumor activity of **25** was further verified in two other sensitive NSCLC cell lines, A549 and NCI-H322 (Table 4). Our data revealed that **25** also limited the growth of the NSCLC A549 and NCI-H322 cells, in the same nanomolar range as observed for the NCI-H460 and NCI-H460/R cancer cell lines (SI, Figure S64) and in excellent agreement with NCI data (SI, Table S20, GI_50_ of 0.232 and 0.174 μM respectively). The antitumor effect of **25** on the NCI-H460 cell line was further confirmed with the Trypan blue exclusion assay, where a significant decrease in the percentage of viable cells was detected when compared with control (SI, Figure S65).

**Table 4:** GI_50_ Concentrations of Compound **25** in A549 and NCI-H322 NSCLC Cell Lines

cell lines	A549	NCI-H322
GI_50_ ± SEM (μM)^a^	0.22 ± 0.02	0.45 ± 0.06

a As determined by SRB assay.

### Noncytotoxic Effect of **25** in Human MRC-5 and MCF12A Nontumorigenic Cells

**Figure 5:**
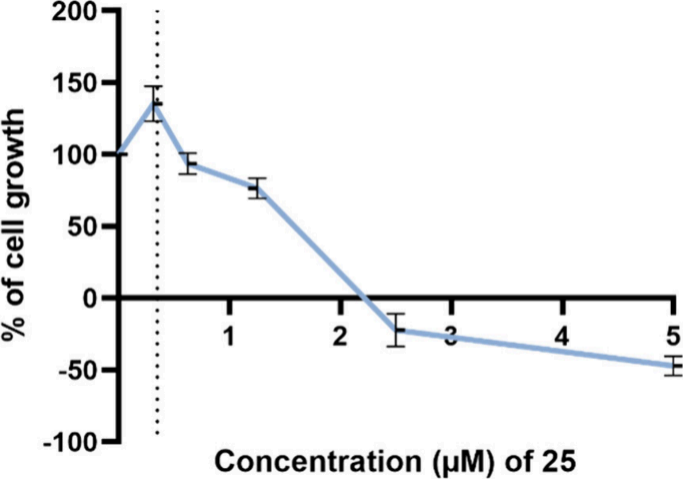
Cytotoxic effect of compound **25** on the nontumorigenic MRC-5 cell line (fibroblasts isolated from the lung tissue), analyzed by the SRB assay. Concentration−response curves of MRC-5 cells exposed to compound **25** for 48 h. Results are presented as percentage (%) of cell growth compared to control cells (vehicle, DMSO). For comparison purposes, the GI_50_ concentration of compound **25** in the NCI-H460 cell line is represented by the vertical dotted line. Results are the mean ± SEM from at least three independent experiments.

Selectivity over nontumorigenic cells is a key requirement in cancer therapy so the cytotoxicity of **25** against nontumorigenic lung fibroblast (MRC-5) and breast cell lines (MCF12A) was evaluated with the SRB assay. The concentration−response curve of MRC-5 exposed to compound **25** is presented in Figure 5. Additionally, results from the MCF12A cell line following 48 h cell treatment with the GI_50_ concentration of **25** previously obtained on the NCI-H460 cells are presented in SI, Figure S67.

The concentration−response curve in the MRC-5 cell line indicates that at low concentrations (below 0.5 μM), compound **25** does not reduce the growth of this cell line. The determined GI_50_ concentration for compound **25** in the MRC-5 cell line was 1.53 μM (±0.075 SEM), which is 4.4 x the GI_50_ concentrations of this compound in the drug-sensitive and MDR NSCLC cells. Moreover, compound **25**, at the concentration tested in the MCF12A cell line, did not cause significant alterations in the growth of these nontumorigenic cells when compared to the controls (vehicle and blank).

### Effect of Compound **25** on ROS Accumulation in NCI-H460 Cells

**Figure 6:**
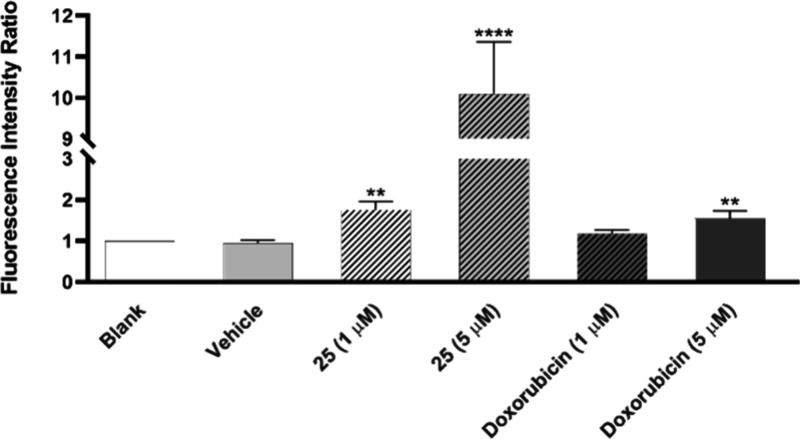
Effect of the compound **25** on ROS accumulation in NCI-H460 cells. Cells were incubated for 6 h with medium, DMSO (vehicle at the % used in the highest tested concentration of **25**), 1 μM and 5 μM of doxorubicin (positive control), and 1 μM and 5 μM of compound **25**. Results are presented as mean fluorescence values, normalized to the blank. Data are expressed as mean ± SEM from at least 3 independent experiments.

To uncover the mechanisms of action of **25**, we analyzed the cellular accumulation of ROS by flow cytometry, using a fluorescent marker of ROS, 5-(and-6)-chloromethyl-2′,7′-dichlorodihydrofluorescein diacetate acetyl ester (CM-H2DCFDA). Figure 6 shows that **25** had a potent effect on the accumulation of ROS in NCI-H460 cells, represented by a statistically significant increase on fluorescence intensity, after cells were treated with this compound for 6 h at concentrations of 0.7 (data not shown), 1, and 5 μM. Importantly, this effect was concentration dependent.

### Effect of Compound **25** on NCI-H460 Cell Proliferation

**Figure 7:**
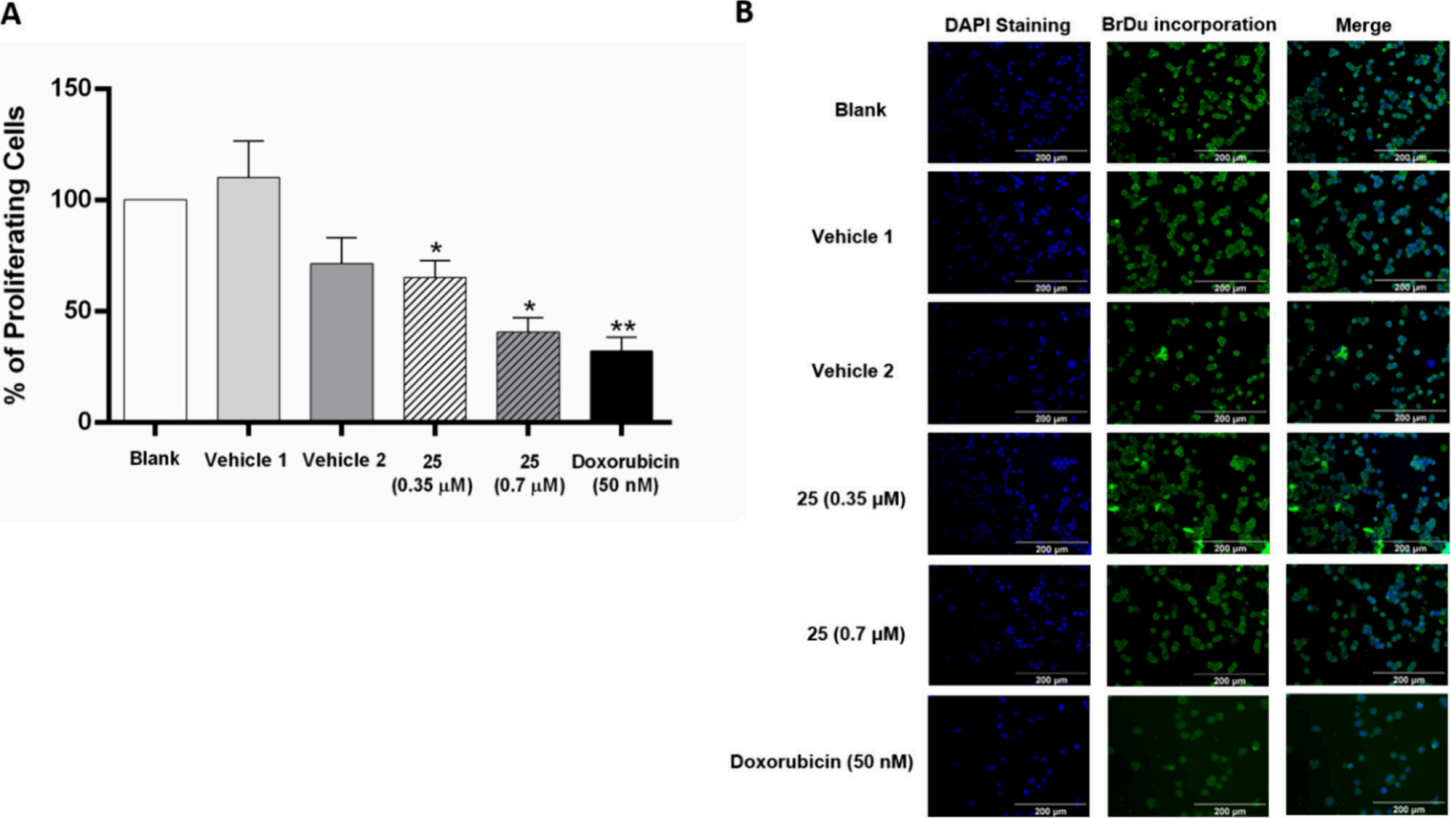
Effect of compound **25** on the % of proliferating NCI-H460 cells, determined with the BrdU assay. Cells were incubated for 48 h with medium, DMSO (vehicle 1, % of vehicle used at the GI_50_ concentration of compound; vehicle 2, % of vehicle used at the 2GI_50_ concentration of compound), doxorubicin (positive control), GI_50_ concentration (0.35 μM), or 2 × GI_50_ concentration (0.7 μM) of compound **25**. (A) Percentage of BrdU-incorporating cells under different treatments. Results are the mean ± SEM from at least 3 independent experiments. * *p* ≤ 0.05, ** *p* ≤ 0.01, when comparing DMSO vs compound treatment. (B) Representative fluorescence microscopy images of BrdU incorporation (green) and DAPI stained nuclei (blue); doxorubicin was used as a positive control.

To assess the impact of compound **25** on cell proliferation, the bromodeoxyuridine (BrdU) incorporation assay was performed. NCI-H460 cells were treated for 48 h with the GI_50_ and 2 × GI_50_ concentrations of **25**. Figure 7 demonstrates that both tested concentrations of compound **25**, as well as the positive control doxorubicin, significantly reduced the percentage of NCI-H460 proliferating cells.

### Effect of Compound **25** on the Cell Cycle Profile of NCI-H460 Cells

**Figure 8:**
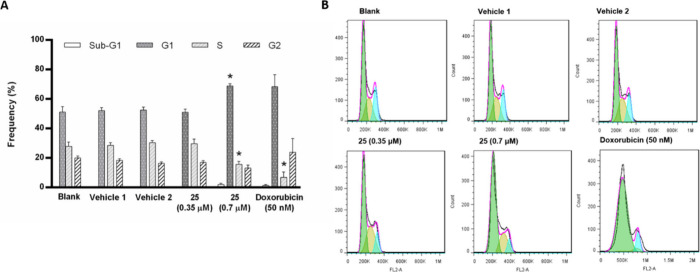
Effect of compound **25** on the cell cycle profile of NCI-H460 cells, analyzed by flow cytometry (PI staining). Cells were incubated for 48 h with medium (blank), DMSO (vehicle 1, % of vehicle used at the GI_50_ concentration of compound; vehicle 2, % of vehicle used at the 2GI_50_ concentration of compound), doxorubicin (positive control), GI_50_ concentration (0.35 μM), or 2 × GI_50_ concentration (0.7 μM) of compound **25**. (A) Frequency (%) of cell cycle phases for each condition. (B) Representative cell cycle histograms. Results are the mean ± SEM from at least 3 independent experiments. * *p* ≤ 0.05, when comparing DMSO vs compound treatment.

The effect of **25** on the cell cycle profile of NCI-H460 cells was assessed. NCI-H460 cells were treated with **25** at the GI_50_ and 2 × GI_50_ concentrations and the cell cycle was analyzed on a flow cytometer following propidium iodide staining. Figure 8 shows that **25** influenced the cell cycle of NCI-H460 cells through an increase in the G1 phase and decrease in the S phase. Furthermore, **25** caused a slight increase in the percentage of cells in the sub-G1 phase, although not statistically significant, suggesting a possible small induction of apoptosis. As described in the literature, the positive control, doxorubicin, caused major alterations in the cell cycle profile of the NCI-H460 cells.^46,47^ Therefore, our data demonstrates that **25** altered the cell cycle profile of these cells, which is in accordance with effects of this compound on cell proliferation and ROS accumulation.

### Effect of Compound **25** on Apoptosis in the NCI-H460 Cells

**Figure 9:**
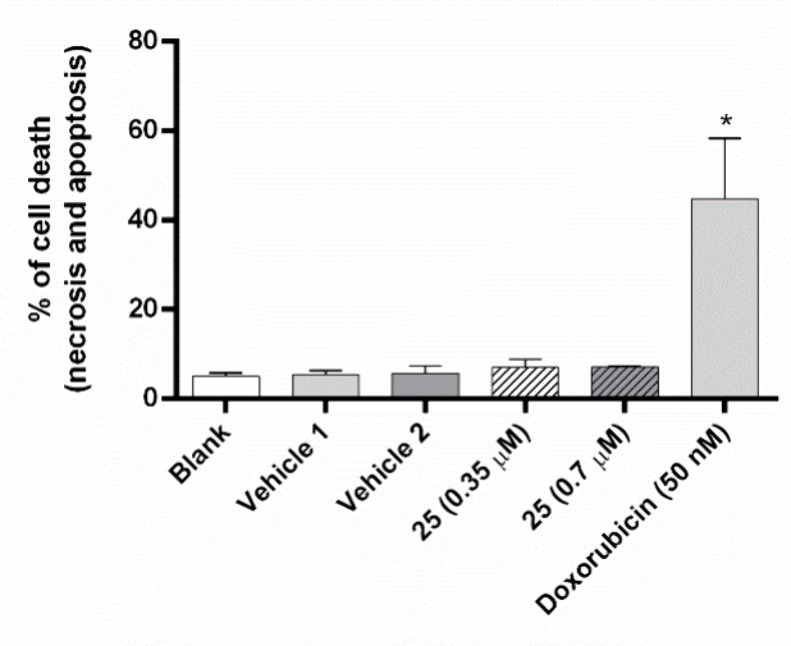
Effect of compound **25** on the levels of death of NCI-H460 cells, analyzed by flow cytometry (annexin V−PI assay). Cells were incubated for 48 h with medium (blank), DMSO (vehicle 1, % of vehicle used at the GI_50_ concentration of compound; vehicle 2, % of vehicle used at the 2GI_50_ concentration of compound), doxorubicin (positive control), GI_50_ concentration (0.35 μM), or the 2 × GI_50_ concentration (0.7 μM) of compound **25**. Results are the mean ± SEM from at least 3 independent experiments. * *p* ≤ 0.05, when comparing DMSO vs compound treatment.

The effect of compound **25** on the levels of apoptosis and necrosis was evaluated using annexin V-FITC/PI labeling followed by flow cytometry analysis. The results, displayed in Figure 9, indicate that treatment of NCI-H460 cells with compound **25**, at the GI_50_ and 2 × GI_50_ concentrations, did not cause an increase in the percentage of dead cells.

### Analysis of the Expression Levels of Several Proteins Involved in Proliferation, Cell Cycle, Apoptosis, and DNA Damage in the NCI-H460 Cells Treated with **25**

**Figure 10:**
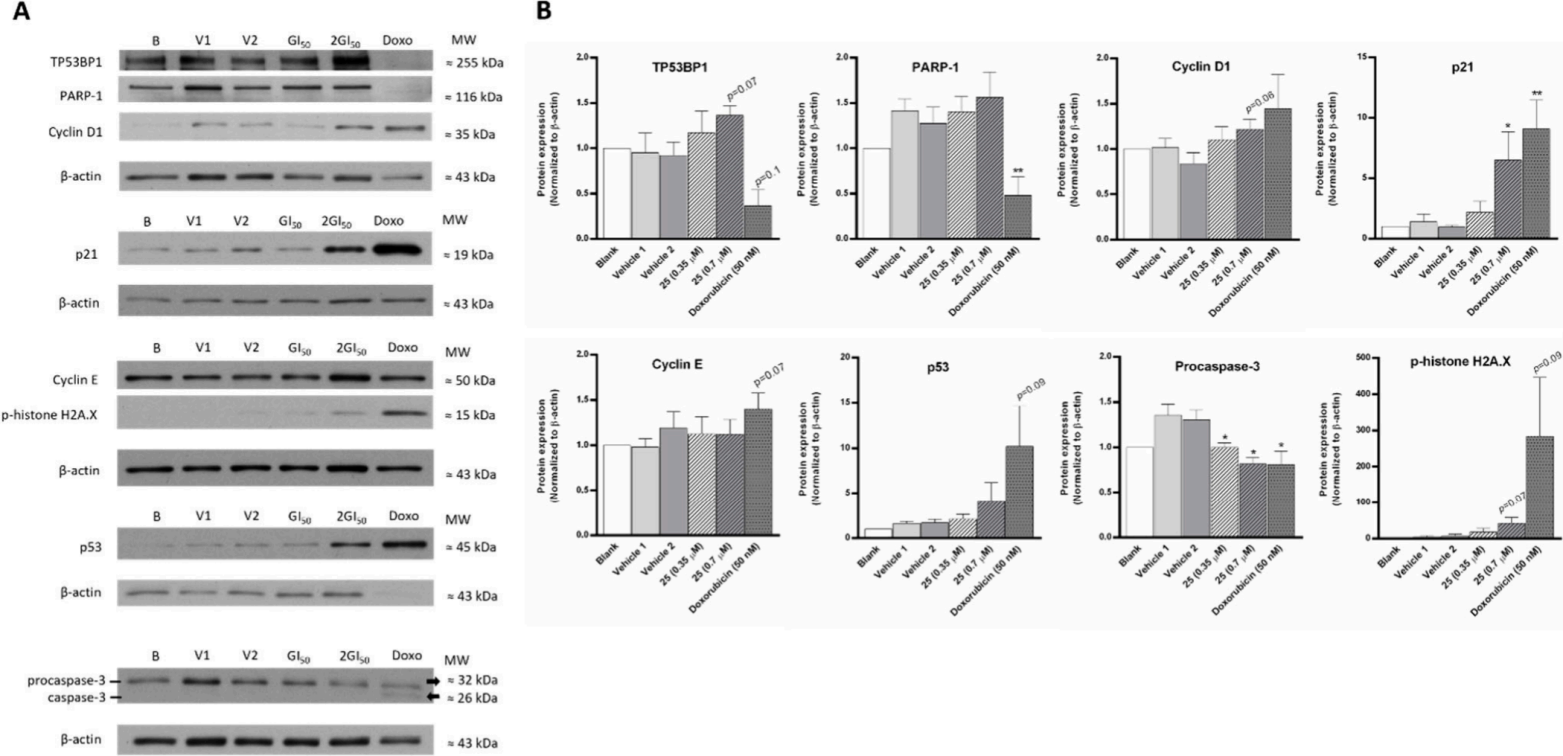
Effect of compound **25** on the NCI-H460 cellular expression of proteins involved in proliferation, cell death and DNA damage, analyzed by Western Blotting. (A) Representative blots of TP53BP1, PARP-1, cyclin D1, p21, cyclin E, *p*-histone H2A.X, p53, and procaspase-3, after cells treatment for 48 h with medium, DMSO (vehicle), doxorubicin (positive control), GI_50_ concentration (0.35 μM), or 2 × GI_50_ concentration (0.7 μM) of compound **25**. V1, % of vehicle used at the GI_50_ concentration of compound; V2, % of vehicle used at 2 × GI_50_ concentration of compound. Doxo: doxorubicin at 50 nM. (B) Graphical representation of the expression of proteins analyzed. β-actin was used as loading control. Results are the mean ± SEM from 3 independent experiments. * *p* ≤ 0.05, ** *p* ≤ 0.01 when comparing DMSO vs compound treatment.

Western blot analysis was performed to evaluate the expression of known proteins associated with cell proliferation, cell cycle, apoptosis, and DNA damage (Figure 10). Compound **25** does not alter the expression levels of PARP-1, at the tested concentrations, although a significant decrease in procaspase-3 was observed in both.

An overexpression of procaspase-3 has been reported in a variety of cancer types, and linked to other oncogenic functions independently of its conversion to caspase-3, such as promotion of a tumorigenic proliferative state, alterations in cell differentiation and promotion of cell migration and survival.^48−54^ Thus, our data suggests that compound **25** did not affect the expression of PARP-1, in accordance with the previously observed lack of effect of this compound on cell death, but a reduction of pro-caspase-3 levels might be involved in its mechanism of action. Compound **25** increased the expression of cyclin D1 (although not statistically significantly). which is in accordance with the observed increase in percentage of cells in the G1 phase of the cell cycle. Compound **25** slightly increased the expression of p53, although not statistically significant. Moreover, compound **25** at 2 × GI_50_ clearly increased the expression of p21, which is downstream of p53.^55−57^ Thus, the effect of **25** on the expression levels of cyclin D1, p53. and p21 in these cells is in accordance with the previously observed effect in impairing the transition of the cell cycle from G1 to S. In addition, the effect of compound **25** on autophagy protein levels was also analyzed. Preliminary results showed that this compound did not alter the expression levels of p62, LC3-I. and LC3-II (data not shown). The expression of proteins involved in DNA damage was also analyzed. Although not statistically significant, compound **25** at the higher concentration tested increased the expression levels of TP53BP1 and of the p-histone H2A.X, which is indicative of DNA damage. However, 48 h treatment of cells with 2 × GI_50_ concentration of compound **25** did not increase DNA damage, as observed on a preliminary Comet Assay analysis (data not shown).

### Effect of Compound **25** on Drug Efflux of NCI-H460/R Cells

Because compound **25** showed a low GI_50_ concentration in the MDR cell line (NCI-H460/R), and because these cells overexpress the MDR1 gene which encodes for P-gp, in contrast to their drug-sensitive counterpart cells of origin (NCI-H460), the activity of compound **25** (following 1 h treatment at 10 or 20 μM) on Pgp-mediated cellular drug efflux was evaluated.^58^

**Figure 11:**
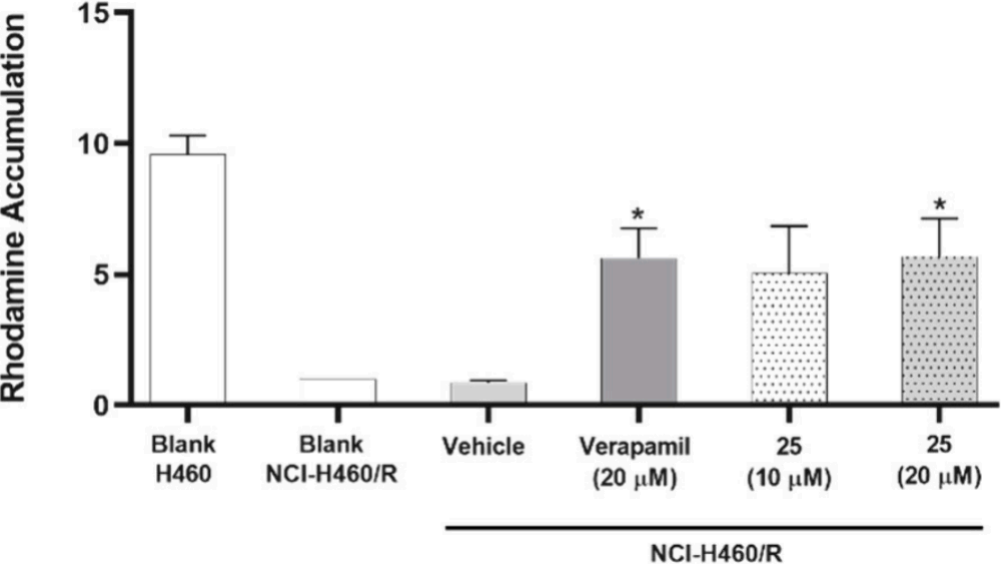
Relative rhodamine-123 accumulation in NCI-H460 or in NCI-H460/R cells following treatment with compound **25**, evaluated by flow cytometry (rhodamine-123 efflux assay). Cells were incubated for 1 h with medium, DMSO (vehicle at the % used in the highest tested concentration of **25**), verapamil (positive control), or compound **25** at two different concentrations (10 and 20 μM). Verapamil (20 μM) was used as a positive control (P-gp inhibitor). Results are the mean ± SEM of at least 3 independent experiments. * *p* ≤ 0.05, when comparing vehicle (DMSO) vs compound treatment in NCI-H460/R cells.

The obtained results from the rhodamine-123 assay (Figure 11) confirmed that **25** caused rhodamine accumulation within the cells, in a similar extent to the positive control verapamil, a known inhibitor of MDR1.^59^ Thus, our data demonstrates that **25** is an inhibitor of P-gp mediated drug efflux.

### Effect of Compound **25** on the Structure and Viability of 3D Spheroids from the NSCLC and Colon Cancer Sensitive and MDR Counterpart Cell Lines

**Figure 12:**
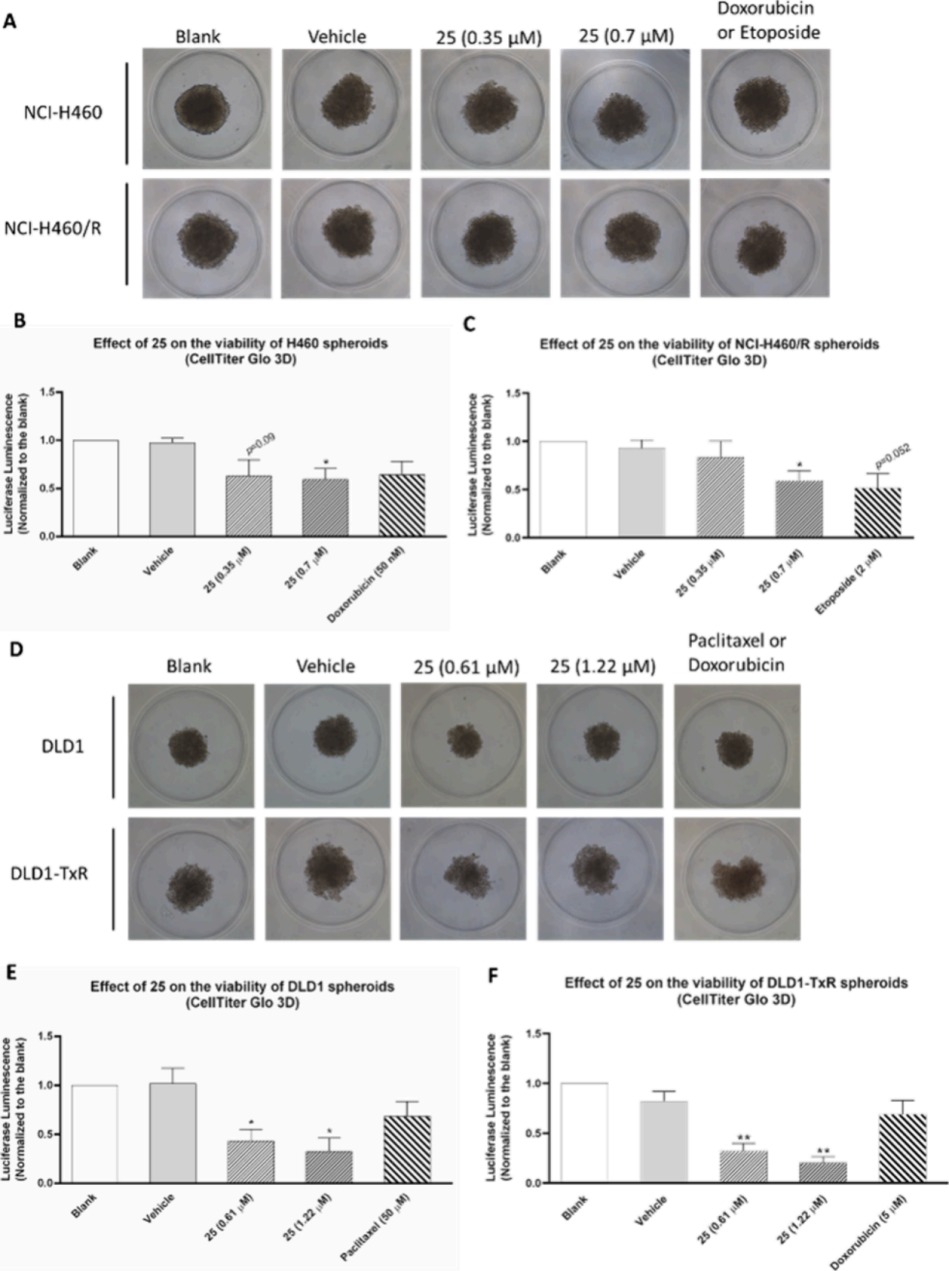
Effect of compound **25** on the viability of spheroids established from two counterpart pairs of sensitive and MDR cell lines, from NSCLC (NCI-H460 and NCI-H460/R) and colorectal cancer (DLD1 and DLD1-TxR), using MicroTissue 3D Petri dishes. (A) Bright-field images of 5 days spheroids treated for 48 h with medium (blank), DMSO (vehicle at the % used in the highest tested concentration of **25**), or compound **25** at the GI_50_ and 2 × GI_50_ concentrations (previously determined in a 2D setting). Doxorubicin (50 nM) and etoposide (2 μM) were used as positive controls for NCI-H460 and NCI-H460/R cell lines, respectively. (B) Effect of compound **25** on the viability of NCI-H460 spheroids analyzed by Cell Titer-Glo 3D viability assay. (C) Effect of compound **25** on the viability of NCI-H460/R spheroids analyzed with the Cell Titer-Glo 3D viability assay. Results are presented as the % of luciferase luminescence relative to the “blank” cells (medium). (D) Bright-field images of 6 days spheroids treated for 48 h with medium (blank), DMSO (vehicle), or compound **25** at the GI_50_ and 2 × GI_50_ concentrations (previously determined in a 2D setting). Paclitaxel (50 μM) and doxorubicin (5 μM) were used as positive controls for DLD1 and DLD1-TxR cell lines, respectively. (E) Effect of compound **25** on the viability of DLD1 spheroids analyzed by Cell Titer-Glo 3D viability assay. (F) Effect of compound **25** on the viability of DLD1-TxR spheroids analyzed with the Cell Titer-Glo 3D viability assay. Data are expressed as mean ± SEM from 3 independent experiments. * *p* ≤ 0.05 and ** *p* ≤ 0.01, when comparing DMSO vs compound treatment.

The three-dimensional (3D) cell models are known to mimic the features of *in vivo* tumors, bridging the existing gap between two-dimensional (2D) cell culture models and animal models and are more sustainable than *in vivo* models.^60^ Thus, 3D models are suggested as promising tools to investigate tumor biology, improve the efficiency of drug screening, and assess drug activity.^60−62^ To confirm the antitumoral activity of compound **25**, we evaluated the effect of this compound on spheroids established from the NSCLC and colorectal cancer sensitive and MDR counterpart cell lines (Figure 12).

Our data showed that spheroids grown in MicroTissue 3D Petri dishes, with sizes between 300 and 400 μm, treated with the GI_50_ and 2 × GI_50_ concentrations of compound **25** (concentrations previously determined by SRB, on 2D cell culture) did not display statistical differences in the AnaSP-calculated morphological properties, such as diameter, compactness and circularity when compared to the controls (SI, Figure S67).

However, the CellTiter-Glo 3D viability assay, which is based on ATP measurements, indicated a significant decrease in the viability of the spheroids from all 4 cell lines when treated with compound **25**. Moreover, the reduction in spheroid viability was more evident in the resistant cell line DLD1-TxR, when compared with the sensitive counterpart cell line. This result is in accordance with the previously obtained results in the 2D cell models (SRB assay), in which a lower GI_50_ concentration was obtained on the DLD1-TxR cells than on the DLD1 cells (selectivity ratio 1.5).

Importantly, besides morphological parameters, quantitative cell viability assessment in spheroids should be employed to better evaluate the effect of a drug.^63^ Furthermore, doxorubicin and etoposide, used as positive controls for both sensitive and MDR cell lines, evaluated at the same concentrations as the ones used in the 2D cell culture assays, did not significantly affect the viability of the spheroids. In fact, it has been described that some drugs do not exhibit the same potency in 3D cell culture models as in 2D, even when using the same cell lines.^60,64−66^ For instance, Ahmed-Cox et al. showed that doxorubicin reduced NCI-H460 spheroid viability only at a very high concentration (20 μM), when compared to the concentration that reduced cell viability of NCI-H460 cells in 2D models (0.1 μM).^67^

Taken together, our 3D results validate the efficacy of compound **25** as an antitumor and anti-MDR agent on NSCLC and colorectal cancer cells at nanomolar concentrations.

### *In Silico* Analysis of Compound **25**

**Figure 13:**
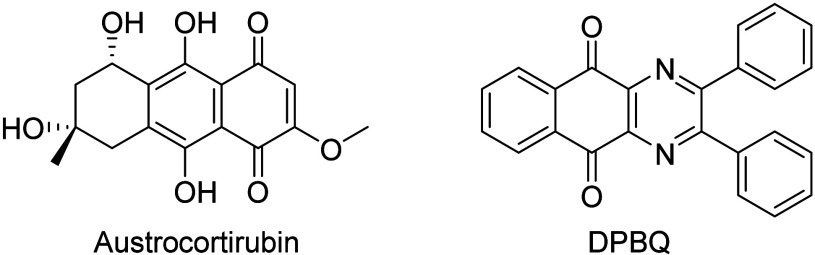
COMPARE analysis of IQQ *N*-oxide **25**.

Given the history of collateral sensitivity emanating from the NCI screening program (e.g., NSC297366, NSC73306), COMPARE analysis was used as a tool to correlate the GI_50_ trends of **25** (NSC810564) against other anticancer compounds to identify similarities.^68^ High correlation within the synthetic data set is seen within the IQQ series, with correlations ranked 1−5 (0.87−0.85) **6** > **8** > **16** > **18** > **14** interestingly all from the C-7 series as opposed to C-6 as expected. A high correlation with NSC805946 (0.77, compound **1**) also confirms the consistent effect on diverse cancer cells. Quinoline quinone methylazaanthraquinone (NSC677735; 0.76) and austrocortirubin (0.69), a naphthazarin based anticancer agent which acts through inducing DNA damage though electrophilic binding and redox cycling were also correlated (Figure 13).^69^ A correlation of 0.75 is seen to MMV009085, a potent human glucose transporter inhibitor, suggesting this as a potential role.^70^ From the mechanistic set, a moderate correlation (0.61) was identified with DPBQ (NSC 629713), a polyploid-specific disruptor of proliferation which elicits the activation of p53 partially through oxidative stress resulting in phosphorylation of p53.^71^

A correlation of 0.61 is also seen with Steffimycin from this set, a potent inhibitor of DNA dependent-RNA synthesis identified as an antibiotic. Steffimycin strongly induces p53 so a mechanistic trend of activity can be seen and will be investigated in further studies.^72^

As IQQ *N*-oxide **25** exhibits potent effects on cells, the *in silico* model SWISSADME was employed to calculate physicochemical descriptors and ADME parameters (SI, Table S4.1, 4.2).^73,74^ Compound **25** is predicted to possess good water solubility and high GI absorption. The Ghose, Lipiniski, Veber, Egan, and Muegge rules for druglikeness were all satisfied and suggests that **25** has pharmaceutical potential. The quinone moiety is a known toxic pharmacophore flagged in the PAINS toxicology screen, but our data shows that at effective antitumor concentrations, **25** has limited if any effect on related non-cancer cells. Further toxicological studies are planned to confirm the nature of these interactions.^75^

## Conclusions

In summary we report the synthesis and screening of 20 novel isoquinolinequinone *N*-oxides with extensive anti-MDR capability. The addition of substituted benzylamines to the IQQ *N*-oxide frameworks **3** and **23** resulted in isolation of two isomers and improved GI_50_ values over lead compounds in most cases. We can conclude that the C(6) isomers are optimal to reduce the growth of cancer cells, and we report improved access to the most potent 6-substituted isomer **25**, which exhibits a mean GI_50_ of 0.21 μM over 52 human tumor cell lines. In-depth MDR analysis confirms that nine of the compounds exhibit a more pronounced growth inhibition effect against MDR cells than sensitive parent cells.

Compound **25** displays nanomolar GI_50_ values against MDR cells from NSCLC and colorectal cancer in 2D sulforhodamine B assays, and at these concentrations, **25** also successfully reduced spheroid viability derived from the same cancer cell lines, using 3D models. On investigation of the mechanism of action, aside from inhibiting the activity of drug efflux pumps in MDR cells, **25** has significant impact on ROS accumulation. However, preliminary data showed that **25** did not induce DNA damage at the 2 × GI_50_ concentration. Because ROS play roles in multiple types of cell death, the effect of **25** on other types of cell death, such as ferroptosis, should be investigated. This has been identified as a potential mechanism of targeting MDR cells and will be explored in future studies. In addition, the effect on other cancer cell phenotypes and those with non-MDR mechanisms of resistance should be assessed.

Compound **25** had a potent inhibitory effect on cell proliferation and affected the cell cycle profile, possibly mediated by p21. In addition, this compound did not interfere with apoptosis, although the role of procaspase-3 in **25**-mediated antitumor activity should be further explored. Overall, our discovery of new anti-MDR compounds indicates that compound **25** is a promising starting point to target a fundamental limitation of cancer chemotherapy.

## Experimental Section

### Chemistry

All solvents were distilled prior to use by the following methods: dichloromethane was distilled from phosphorus pentoxide, ethyl acetate was distilled from potassium carbonate, and hexane was distilled prior to use. Organic phases were dried over anhydrous magnesium sulfate. All commercial reagents were procured from both international and local suppliers such as Fluorochem, Acros, Merck, and Alpha Aesar and were used without further purification unless stated otherwise. ^1^H (300/400/600 MHz) and ^13^C (75/100/150 MHz) NMR spectra were recorded on a Bruker Avance 300 or 400 NMR spectrometer. All spectra were recorded at 20 °C in deuterated dimethyl sulfoxide (DMSO-*d*_*6*_) or deuterated chloroform (CDCl_3_) using tetramethylsilane (TMS) as an internal standard unless otherwise stated. Chemical shifts (δ_H_ and δ_C_) are reported in parts per million (ppm) relative to the reference peak. The order of citation in parentheses is (a) number of protons, (b) multiplicity (s = singlet, bs = broad singlet, d = doublet, t = triplet, q = quartet, dd= doublet of doublet, quin. = quintet, m = multiplet), (c) coupling constants, (coupling constants (*J*) are reported in hertz (Hz)). Infrared spectra were recorded as thin films on sodium chloride plates for oils or as potassium bromide (KBr) discs for solids on a PerkinElmer Spectrum 100 FT-IR spectrometer or a PerkinElmer Spectrum One FT-IR spectrometer. Nominal mass spectra were recorded on a Waters Quattro Micro triple quadrupole spectrometer (QAA1202) in ESI mode using 50% acetonitrile−water containing 0.1% formic acid as eluent. High resolution mass spectra (HRMS) were recorded on a Waters LCT Premier time-of-flight spectrometer (KD160) or a Waters Vion IMS mass spectrometer (SAA055 K) in Supporting Information were dissolved in acetonitrile, water, or 10% DMSO/acetonitrile. Melting points were measured on a unimelt Thomas−Hoover capillary melting point apparatus and are uncorrected. Thin layer chromatography (TLC) was carried out on precoated silica gel plates (Merck 60 PF254). Visualization was achieved by UV light detection (254 and 366 nm). Wet flash column chromatography was performed using Merck PF254 silica gel unless otherwise stated. The purity of title compounds was determined as at least 95% using an Waters Acquity I-Class ultra high-performance liquid chromatography (UPLC) system with a Waters Acquity BEH peptide C18 column (2.1 mm × 100 mm, 1.7 μm) a Waters Acquity PDA and a Waters Vion IMS mass spectrometer (SAA055K) in ESI mode using a gradient (10:90 to 90:10) of acetonitrile−water containing 0.1% formic acid as eluent with peak detection at 254 nm. The general procedures and NMR data of all title compounds and the corresponding intermediates are described in the Supporting Information.

### Biological Materials and Methods

#### Preparation of Compounds

The IQQ *N*-oxides were dissolved in dimethyl-sulfoxide (DMSO; Merck Life Science, Darmstadt, Germany) at stock solutions of 60 mM. Appropriate fresh working solutions were done in DMSO for the cell-based assays. Doxorubicin, etoposide, paclitaxel (Merck Life Science), and verapamil (Sigma-Aldrich) were used as positive controls.

#### Cell Culture Conditions

Two pairs of drug-sensitive and multidrug-resistant (MDR) cell lines from human nonsmall cell lung cancer (NSCLC), NCI-H460 and NCI-H460/R, and colorectal cancer, DLD1 and DLD1-TxR, kindly provided by Dr M. Pešić (Belgrade, Serbia), were used to evaluate the growth inhibition potential of the compounds **1**, **2**, **3**, **4**, **5**, **20**, **23**, **24**, an **25**.^76,77^ Additionally, growth inhibition assays were performed with compound **25** on two other NSCLC cell lines, A549 and NCI-H322, obtained from the American Type Culture Collection (ATCC) and the European Collection of Authenticated Cell Cultures, respectively. The A549 cell line was maintained in Dulbecco’s Modified Eagle Medium (DMEM) supplemented with 4.5 g/L Glucose with UltraGlutamine with sodium pyruvate (Lonza, Basel Stücki, Switzerland), enriched with 10% fetal bovine serum (FBS; Biowest, Nuaillé, France). The lung fibroblast MRC-5 cell line and the remaining NSCLC and colorectal cancer cell lines were cultured in Roswell Park Memorial Institute (RPMI) 1640 (with stable glutamine and 25 mM HEPES) medium (Biowest), supplemented with 10% fetal bovine serum. The resistant cell lines, NCI-H460/R and DLD1-TxR, were exposed to 100 nM doxorubicin and paclitaxel, respectively, every 2 weeks in order to maintain cell resistance. For the sulforhodamine B (SRB) and 3D cell culture assays, the media were supplemented with 5% FBS. The remaining experiments were performed with medium supplemented with 10% FBS.

The nontumorigenic cell line MCF-12A, also purchased from ATCC, was cultured in a modified DMEM/F12 medium, as previously described.^79^

All cell lines were grown at 37 °C in a humidified incubator with 5% CO_2_ in air. Cells were regularly observed using the Leica DMi1 microscope (Leica Biosystems, Wetzlar, Germany), and all experiments were executed with cells in their exponential growth phase, ensuring viability over 90%. Moreover, cell lines were genotyped and monitored for mycoplasma contamination (Cell Culture and Genotyping Service, i3S).

#### Cell Growth Inhibition and Cytotoxicity using the Sulforhodamine B (SRB) Assay

Cells were seeded at a previously determined optimal cell concentration (5.0 × 10^4^ cells/mL for NSCLC cells and the nontumorigenic MRC-5 cell line and 1.0 × 10^5^ cells/mL for colorectal cancer cells) and incubated at 37 °C for 24 h. Then, adhered cells were treated with five serial dilutions (1:2) of the compounds or antitumor drugs used as controls (doxorubicin and paclitaxel) and incubated at 37 °C for further 48 h. The SRB assay was performed as previously described.^78,79^ Briefly, after fixing cells with 10% (w/v) ice-cold trichloroacetic acid (Merck Life Science), the proteins were stained with 0.4% of SRB (Merck Life Science) in acetic acid (Merck Life Science). Following washing steps in acetic acid, the bound dye was solubilized with 10 mM Tris base solution (Merck Life Science). Absorbance was measured at 510 nm in a multiplate reader (Synergy Mx, Biotek Instruments Inc., Winooski, Vermont, USA) using the Gen5 software (Biotek Instruments Inc.).

Compound **25** was also tested on the nontumorigenic MCF-12A human breast epithelial cell line. For that, cells seeded at 5.0 × 10^4^/mL were incubated with the highest GI_50_ concentration (that caused 50% of cell growth inhibition) previously obtained in the tumor cell lines, for 48 h. Following this treatment, the SRB assay was performed as described above.

#### Cell Viability: Trypan Blue Exclusion Assay

NCI-H460 cells (5 × 10^4^ cells/mL) were treated with the GI_50_ and 2 × GI_50_ concentrations of compound **25** for 48 h. After this treatment, cells were mixed with 0.2% (v/v) trypan blue dye (Merck LifeScience) and counted on an hemocytometer (Neubauer Chamber). Doxorubicin (50 nM) was used as a positive control.

#### ROS Accumulation: Flow Cytometry Analysis of CM-H2DCFDA Staining

To study the effect of compound **25** on ROS levels, 5-(and-6)-chloromethyl-2′,7′-dichlorodihydrofluorescein diacetate, acetyl ester (CM-H2DCFDA; Invitrogen) analysis by flow cytometry was performed in the drug-sensitive cells. NCI-H460 cells (1.5 × 10^5^/mL) were plated in 6-well plates for 24 h. Cells were first incubated with 10 μM CM-H2DCFDA for 45 min at 37 °C in the dark and then treated with the following for 6 h: culture media, vehicle (DMSO), doxorubicin (positive control at 1 μM and 5 μM) and compound 25 at 0.70 μM (corresponding to 2x GI50), 1 μM and 5 μM. Harvested cells were subsequently washed twice in phosphate-buffered saline (PBS) and the CM-H2DCFDA fluorescence was analyzed in a BD AccuriTM flow cytometer (BD Biosciences, San Jose, California, USA). Mean fluorescence intensity was calculated after correction for autofluorescence.

#### Cell Proliferation: 5-Bromo-2′-Deoxyuridine (BrdU) Incorporation Assay

NCI-H460 cells were seeded in 6-well plates (5 × 10^4^ cells/mL) for 24 h and then treated with the GI_50_ and 2 × GI_50_ concentrations of compound **25** for 48 h. Doxorubicin (at 50 nM) was used as a positive control. At 4 h before the end of this treatment, cells were incubated with 1 μM of BrdU (Merck Life Science). After collection of cells, they were fixed with paraformaldehyde 4% (Merck Life Science) for 40 min at room temperature (RT). Then, cells were subjected to a cytospin (500 rpm for 5 min), and cellular DNA was denaturated using 2 M of HCl (Merck Life Science) for 20 min. Cells were incubated with a PBS solution containing 0.5% Tween (Promega, Madison, Wisconsin, USA) and 0.05% bovine serum albumin (BSA; Merck Life Science). The cells were further incubated with a primary monoclonal mouse anti-BrdU Clone Bu20a antibody (1:10; Dako, Glostrup, Denmark) for 1 h, followed by incubation with a polyclonal rabbit antimouse immunoglobulins/FITC secondary antibody (1:100; Dako) for 30 min at RT. Slides were mounted in Vectashield mounting media with 4′,6-diamidino-2-phenylindole (DAPI; Vector Laboratories Inc., Peterborough, UK), and the cell images were obtained on a Zeiss Axio Imager Z1 (Carl Zeiss, Jena, Germany) microscope, equipped with the Axiovision 4.9 (Carl Zeiss) software. The cells were counted using the ImageJ 2.1.0 software.

#### Cell Cycle Profile: Propidium Iodide (PI) Staining and Flow Cytometry

NCI-H460 cells (5 × 10^4^ cells/mL) were plated in 6-well plates and then treated with the GI_50_ and 2 × GI_50_ concentrations of compound **25** or the positive control (doxorubicin at 50 nM) for 48 h. Collected cells were then fixed in ice-cold 70% ethanol (Fisher Scientific) overnight. Cells were then centrifuged again and resuspended in 5 μg/mL of PI (Merck Life Science) and 0.1 mg/mL RNase A (Invitrogen) in PBS, and kept in the dark for at least 30 min. Cellular DNA content was analyzed using the BD Accuri C6 Flow cytometer. At least 10 000 events per sample were acquired. FlowJo 7.6.5 software (Tree Star, Inc., Ashland, Oregon, USA) was used to determine the % of cells in the different phases of the cell cycle.

#### Cell Death: Annexin V-FITC Apoptosis Detection Assay

Analysis of cell death was performed using the Annexin V-FITC apoptosis detection kit (eBioscience, Thermo Fisher Scientific). NCI-H460 cells were plated in 6-well plates (5 × 10^4^ cells/mL) for 24 h and then treated with the GI_50_ and 2 × GI_50_ concentrations of compound **25** for 48 h. Exposure to 5% ethanol for 1 h was used as a positive control for necrosis. Collected cells were resuspended in the binding buffer solution and incubated with the Annexin V-FITC conjugate for 10 min at RT, in the dark. Afterward, PI was added, and samples were analyzed using the BD Accuri C6 flow cytometer, where at least 10 000 events per sample were plotted. Data analysis was carried out using the BD Accuri C6 Software.

#### Inhibition of Drug Efflux Pumps: Rhodamine-123 Accumulation Assay

The NSCLC cells (1.5 × 10^5^ cells/mL) were incubated for 1 h at 37 °C in the presence of 10 μM or 20 μM of compound **25** and with 1 μM of rhodamine-123 (Sigma-Aldrich). NCI-H460 cells without treatment were used as a negative control and NCI-H460/R cells treated with verapamil (a known P-gp inhibitor) at concentrations of 10 and 20 μM were used as a positive controls.^80,81^ After the incubation time, cells were washed in cold PBS and kept at 4 °C, protected from light until analysis in the flow cytometer. At least 10 000 cells per sample were acquired and analyzed on the BD Accuri C6 flow cytometer.

#### Protein Expression Analysis: Protein Extraction and Western Blotting

The NCI-H460 cells (5 × 10^4^ cells/mL) were plated in 6-well plates and then treated with the GI_50_ and 2 × GI_50_ concentrations of compound **25** for 48 h. After this treatment, cells were centrifuged at 1200 rpm for 5 min at 4 °C, and then lysed in Wynman’s buffer, as previously described,^79^ containing EDTA-free protease (Roche, Basel, Switzerland) and phosphatase inhibitors (Merck Life Science) for 30 min with agitation and at 4 °C. Protein lysates were obtained after centrifugation at 13 000 rpm, for 10 min at 4 °C, and stored at −20 °C until used. A modified Lowry protocol, DC Protein assay kit (Bio-Rad, Hercules, California, USA), was used to quantify the total protein content (using a BSA standard calibration curve). The absorbance was analyzed with the Gen5 software on the microplate reader Synergy Mx, at 488 nm excitation and read at 655 nm emission.

Then 20 μg of protein per treatment condition was loaded and separated in 12% SDS-PAGE gels and electrophoretically transferred into nitrocellulose membranes (GE Healthcare Life Science, Chalfont St Giles, UK) for 2 h using a Mini Trans-Blot Cell system (Bio-Rad). The membranes were then stained with Ponceau S solution (PanReac AppliChem, Barcelona, Spain) to confirm that proper protein transfer occurred. Then, the membranes were blocked in Tris-buffered saline solution (TBS) pH 7.4 containing 5% (w/v) nonfat dry milk (Molico, Nestlé, Vevey, Switzerland) or 5% (w/v) BSA for 2 h at RT. The membranes were incubated for 90 min at RT with the following primary antibodies: caspase-3 (1:200; sc-56053), cyclin D1 (1:200; sc-8396), cyclin E (1:100; sc-377100), p21 (1:200; sc-6246), p53 (1:200; sc-126), PARP-1 (1:200; sc-8007), *p*-Histone H2A.X (1:200; sc-517348), TP53BP1 (1:100; sc-515841), and β-actin (1:200; sc-13118) from Santa Cruz Biotechnology, Dallas, Texas, USA. Following washing steps in TBS-T, the membranes were incubated with their respective secondary antibodies, antimouse IgG horseradish peroxidase (HRP) (Cytiva, Marlborough, Massachusetts, USA) or antirabbit IgG-HRP (Cytiva) for 1 h at RT in an orbital shaker. After 3 washing steps with TBS-T, peroxidase activity was revealed in Amersham Hyperfilm ECL (Cytiva), using the ECL Western Blotting detection reagents (Cytiva). The signal was detected with a Fuji Medical Film Processor (FPM-100A model), and the immunoblots were digitalized using the GS-800 calibrated densitometer (Bio-Rad). Band quantification was carried out with Software Image Lab version 6.0.1 (Bio-Rad), and the molecular weight of protein bands were estimated by comparison with the bands from a protein marker (PageRuler Plus Prestained Protein Ladder, 10−250 kDa, Thermo Fisher Scientific).

#### DNA Damage Analysis: Alkaline Comet Assay

NCI-H460 cells (5 × 10^4^ cells/mL) in 6-well plates were treated with compound **25** at 0.7 μM (2 × GI_50_ concentration), 1 μM, and 2 μM for 48 h. Hydrogen peroxide (Sigma-Aldrich) was used as a positive control (50 μM). Harvested cells were resuspended in 0.5% low melting point agarose (Thermo Fisher Scientific) and poured onto slides precoated with 1% normal melting point agarose (Sigma-Aldrich). Cells were then lysed with ice-cold lysis buffer [100 mM Na_2_EDTA (Sigma-Aldrich), 25 M NaCl (Sigma-Aldrich), 10 mM Tris-Base (Sigma-Aldrich), pH 10.0, 1% and Triton X-100 (Sigma-Aldrich)) for 2 h in the dark at 4 °C. Then, after two washes with distilled water, slides were placed on an electrophoresis tank filled with electrophoresis buffer [300 mM NaOH (Sigma-Aldrich), 1 mM Na_2_EDTA; pH 13.0] and incubated for 40 min to allow DNA to unwind. Electrophoresis was carried out for 20 min at 32 V. Then slides were immersed twice in pure ethanol and allowed to dry at RT. Slides were stained with SyberGold staining solution (Invitrogen) in Tris-EDTA buffer [10 mM Tris-HCl (Sigma-Aldrich) and 1 mM EDTA (Sigma-Aldrich); pH 7.5] for 30 min with agitation and protected from light. In the end, slides washed and dried were visualized on a Zeiss Axio Imager Z1 microscope, equipped with the Axiovision 4.9 software.

#### 3D Cell Culture. Spheroid Formation and Disruption: MicroTissues 3D Petri Dish Micromold Spheroids

Spheroid formation was conducted in 12-series agarose 3D Petri Dish micromolds (MicroTissues Technology, MicroTissues Inc., Sigma-Aldrich). The agarose micromolds were prepared using 1% agarose in a 0.9% NaCl solution and then placed in 12-well plates and equilibrated with RPMI supplemented with 10% FBS for at least 2 h before cell seeding. Cell suspensions corresponding to 500 cells per spheroid of the NSCLC cell lines (NCI-H460 and NCI-H460/R), and 1000 cells per spheroid of the colorectal cancer cell lines (DLD1 and DLD1-TxR) were seeded in the corresponding micromolds. The choice of cell seeding concentration was based on a previous optimization assay (data not shown), ensuring that at the end of the assay the spheroids would have a size of around 400 μm. A 20 min incubation period was used for cells to settle into the micromolds and additional medium was finally added to the wells. Spheroid formation was monitored with the Leica DMi1 microscope. The NSCLC spheroids were allowed to form for 3 days and the colorectal cancer spheroids for 4 days, prior to the 48 h treatment with the following: culture medium, vehicle (DMSO), compound **25** at the GI_50_ and 2 × GI_50_ concentrations previously determined in 2D culture (for NCI-H460 and NCI-H460/R cells: 0.35 and 0.7 μM, respectively; and for DLD1 and DLD1-TxR cells: 0.61 and 1.22 μM respectively), and positive controls such as doxorubicin (at 50 nM for NCI-H460 and 5 μM for DLD1-TxR), etoposide (at 2 μM for NCI-H460/R) and paclitaxel (at 50 μM for DLD1). At the end of the treatment, images from at least 8 different spheroids per condition were captured on the Leica Dmi1 microscope, and posteriorly analyzed with the automated open-source software AnaSP.^82^ Spheroid parameters, such as equivalent diameter, circularity, and compactness, were calculated.

#### Spheroid Viability: Cell Titer-Glo 3D Cell Viability Assay

Spheroid viability was determined through the luminescence-based Cell Titer-Glo 3D cell viability assay (Promega). Spheroids grown on MicroTissues 3D Petri Dish micromolds were exposed to the treatments, as described in the previous section. Then, the spheroids were transferred into 96-well plates in 50 μL of RPMI medium. The same volume of 3D Cell Titer-Glo reagent was added to each well, according to the manufacturer’s instructions. The plate was then incubated in the dark for 5 min with agitation and allowed to rest for 25 min before luminescence measurement. Luminescence was quantified using the microplate reader Synergy MX coupled with the GEN5 software. Three internal replicates of each sample were performed per experiment.

#### Statistical Analysis

All presented data resulted from at least 3 independent experiments, and results are expressed as mean ± SEM. Statistical analysis was performed using the two-tailed unpaired *t* test, with GraphPad Prism 8.0 software. Results were considered statistically significant when *p* ≤ 0.05.

## Supporting Information Available

Chemical experimental and characterization; NCI data; growth effects of **25** on parental cells and their P-gp expressing drug resistant pairs; COMPARE analysis and predicted physicochemical properties of IQQ *N*-oxides (PDF)
Molecular formula strings (CSV)

## Abbreviations Used

ATCC: American Type Culture Collection
ATP: adenosine triphosphate
BrdU: bromodeoxyuridine
BSA: bovine serum albumin
CM-H2DCFDA: 5-(and-6)-chloromethyl-2′,7′-dichlorodihydrofluorescein diacetate acetyl ester
DAPI: 4′,6′-diamino-2-phenyl-indol
DMEM: Dulbecco’s Modified Eagle Medium
DMSO: dimethyl sulfoxide
DNA: deoxyribonucleic acid
FBS: fetal bovine serum
FITC: fluorescein isothiocyanate
GI: growth inhibition
HRP: horseradish peroxidase
IQQ: isoquinolinequinone
MDR: multidrug resistance
NSCLC: nonsmall cell lung cancer
PBS: phosphate buffered saline
PI: propidium iodide
ROS: reactive oxygen species
RPMI: Roswell Park Memorial Institute
RT: room temperature
SEM: standard error of the mean
SRB: sulforhodamine B
TBS: tris-buffered saline
