# Supplementary material for: Discovery of Potent Isoquinolinequinone N-Oxides to Overcome Cancer Multidrug Resistance
Source: J Med Chem. 2024 Aug 2;67(16):13909–24. doi: 10.1021/acs.jmedchem.4c00705 (PMC11345829; doi:10.1021/acs.jmedchem.4c00705)
Supplement: Supplementary file 1 — jm4c00705_si_001.pdf [file jm4c00705_si_001.pdf]

Supplementary Information

Discovery of Potent Isoquinolinequinone *N*-oxides to Overcome Cancer Multidrug Resistance

Ryan D. Kruschel [1,#], Mélanie A. G. Barbosa [2,3,4,#], Maria João Almeida [2,3], Cristina P. R. Xavier [2,3], M. Helena Vasconcelos [2,3,4,*], Florence O. McCarthy [1,*]

[1] School of Chemistry, Analytical and Biological Chemistry Research Facility, University College Cork, Cork T12 K8AF, Ireland.

[2] i3S – Instituto de Investigação e Inovação em Saúde, Universidade do Porto, 4200-135 Porto, Portugal.

[3] Cancer Drug Resistance Group, IPATIMUP – Institute of Molecular Pathology and Immunology, University of Porto, 4200-135 Porto, Portugal,

[4] FFUP – Faculty of Pharmacy of the University of Porto, 4050-313 Porto, Portugal

[#] These authors contributed equality to this work.

[*] Corresponding authors. hvasconcelos@ipatimup.pt (M. Helena Vasconcelos); f.mccarthy@ucc.ie (Florence O. McCarthy).

Contents

S1: Chemical experimental and characterisation — **Page**
- **S1.1** Chemical Experimental Procedures and Characterisation — S2
- **S1.2** Structural confirmation of substitution at C-6 and C-7 (*Figure S1*) — S15
- **S1.3** Structural confirmation of C-6 substitution of **25** (*Figure S2*) — S16
- **S1.4** HPLC purity of **25** (*Figure S3; Table S1*) — S17
- **S1.5** ^{1}H and ^{13}C NMR spectra for compounds **3-25** — S18

S2: NCI data
- **S2.1** NCI Experimental details — S40
- **S2.2** One-dose and Five-dose data for **4-25** (*Figure S4-S61; Table S2-S20*) — S41
- **S2.3** Comparison of NCI-H460 cell line GI_{50}'s (*Table S21)* — S119

S3: Growth effects of 25 on parental cells and their P-gp expressing drug resistant pairs.
- **S3.1** Dose response of NCI-H460/NCI-H460/R NSCLC cell lines to **25** (*Figure S62*) — S120
- **S3.2** Dose response of DLD1/DLD1-TxR cancer cell lines to **25** (*Figure S63*) — S121
- **S3.3** Dose response curves of A549/NCI-H322 NSCLC cell lines to **25** *(Figure S64)* — S122
- **S3.4** Effect of **25** on the viable cell number of NCI-H460 cells (*Figure S65*) — S123
- **S3.5** Effect of **25** on the non-tumorigenic MCF12A cell line (*Figure S66*) — S124
- **S3.6** Effect of **25** on spheroids from NSCLC and colorectal cancer (*Figure S67*) — S125

S4: COMPARE analysis and predicted physicochemical properties of IQQ *N*-oxides
- **S4.1** Compare Analysis — S126
- **S4.2** Predicted physicochemical properties of IQQ *N*-oxides (*Table S22-S23*) — S127

Supplementary Information References — S129

S1: Chemical experimental and characterisation

S1.1 Chemical Experimental Procedures and Characterisation

Methyl 1,3-dimethyl-5,8-dioxo-5,8-dihydroisoquinoline-4-carboxylate[1]

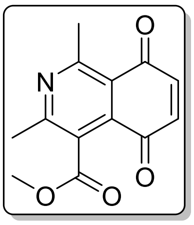

A suspension of 2,5-dihydroxyacetophenone (2.040 g, 13.41 mmol), silver (I) oxide (6.081 g, 26.24 mmol), methyl 3-aminocrotonate (1.620 g, 14.07 mmol) and magnesium sulfate (6.405 g) was stirred vigorously at room temperature in dichloromethane (330 mL) resulting in a black suspension. The reaction mixture was stirred for 18 hours at room temperature until reaction completion, confirmed by TLC (1:1 Ethyl acetate: Hexane), resulting in a yellow solution with grey precipitate. The crude reaction mixture was concentrated under reduced pressure yielding a crude black solid which was subjected to flash column chromatography (1:0 Hexane: Ethyl Acetate – 4:6 Hexane: Ethyl acetate) yielding the product as a yellow solid, (2.872 g, 88%) (R_f: 0.6, Hexane-Ethyl acetate 1:1). m.p. 121.0 – 123.0 °C (Lit. 120.0 – 122.0 °C)[1]; ν_{max}/cm^{-1} (KBr): 3028, 2954, 1726, 1679, 1663, 1568, 1541, 1430, 1383, 1327, 1296, 1254, 1218, 1118, 1090, 871; ^{1}H-NMR (300MHz, $CDCl_3$): δ 2.62 (s, 3H), 2.97 (s, 3H), 4.02 (s, 3H) and 6.95 (s, 2H) ppm; *m/z* (ESI^+): 246.2 $[M+H]^+$.

4-(Methoxycarbonyl)-1,3-dimethyl-5,8-dioxo-5,8-dihydroisoquinoline 2-oxide 3[2]

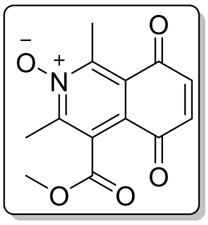

Methyl 1,3-dimethyl-5,8-dioxo-5,8-dihydro-isoquinoline-4-carboxylate (0.211 g, 0.860 mmol) was dissolved in dichloromethane (3 mL) with stirring resulting in a dark yellow solution. *meta*-Chloroperoxybenzoic acid (0.282 g, 1.634 mmol) was then added resulting in a transparent orange solution. The reaction was stirred at room temperature for 24 hours resulting in a dark orange solution with white precipitate. TLC analysis was undertaken at this point to ensure reaction completion (1:1 Hexane: Ethyl Acetate). The reaction mixture was concentrated under reduced pressure yielding a crude orange solid. Column chromatography was performed on this crude material (1:0 Hexane: Ethyl acetate – 3:7 Hexane: Ethyl acetate) yielding the product as an orange solid, **3** (0.150 g, 70%) (R_f: 0.5, Hexane-Ethyl acetate 1:1). m.p. 119.0 – 120.0 °C (degrad.) (Lit. 119.0 – 120.0 °C)[2]; ν_{max}/cm^{-1} (KBr): 3069, 2952, 1743, 1662, 1436, 1375, 1331, 1299, 1231, 1064, 853; ^{1}H-NMR (400MHz, $CDCl_3$): δ 2.52 (s, 3H), 2.98 (s, 3H), 4.05 (s, 3H) and 6.97 (ab quartet, *J* = 3.8 Hz, 2H) ppm; ^{13}C-NMR (100 MHz, $CDCl_3$): δ 14.8, 16.2, 53.6,

122.2, 125.0, 128.2, 137.3, 139.9, 151.1, 152.1, 166.3, 181.8 and 184.3 ppm; *m/z* (ESI$^+$): 262.2 [M+H]$^+$; HRMS (ESI$^+$): Exact mass calculated for $C_{13}H_{12}NO_5$ 262.0715. Found 262.0705.

7-(Benzylamino)-4-(methoxycarbonyl)-1,3-dimethyl-5,8-dioxo-5,8-dihydroisoquinoline 2-oxide 1[2]

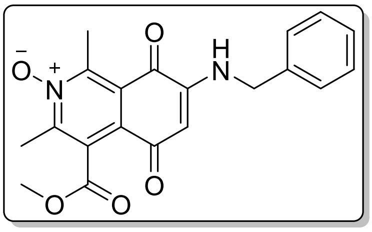

A suspension of 4-(methoxycarbonyl) -1,3-dimethyl-5,8-dioxo-5,8-dihydroiso-quinoline 2-oxide **3** (0.181 g, 0.69 mmol), cerium chloride heptahydrate (0.025 g, 0.07 mmol), benzylamine (0.15 mL, 1.37 mmol) in absolute ethanol (20 mL) was stirred for three hours at room temperature. The consumption of **3** was monitored using TLC (1:1 Hexane: Ethyl acetate The reaction mixture was concentrated under reduced pressure yielding a crude dark red oil, which was subjected to flash column chromatography (1:0 Hexane: Ethyl acetate – 6:4 Hexane: Ethyl acetate The eluents were concentrated under reduced pressure to yield the product as a red/orange solid, **1** (0.141 g, 56%) (R_f: 0.2, Hexane-Ethyl acetate 1:1). m.p. 160 – 163 °C (Lit. 160.0 – 163.0 °C)[2]; ν_{max}/cm^{-1} (KBr): 3324, 1742, 1692, 1605, 1542, 1511, 1311, 1225, 1064; ^{1}H-NMR (300MHz, $CDCl_3$): δ 2.52 (s, 3H), 2.99 (s, 3H), 4.01 (s, 3H), 4.37 (d, *J* = 5.7 Hz, 2H), 5.75 (s, 1H), 6.35 (bs, 1H) and 7.27 – 7.39 (m, 5H) ppm; ^{13}C-NMR (100 MHz, $CDCl_3$): δ 15.1, 16.3, 47.0, 53.3, 101.1, 123.9, 124.7, 127.6 [2C], 128.30, 128.38, 129.1 [2C], 135.2, 148.0, 151.6, 151.7, 166.9, 178.9 and 181.1 ppm; *m/z* (ESI$^+$): 367.3 [M+H]$^+$, 100%; HRMS (ESI$^+$): Exact mass calculated for $C_{20}H_{19}N_2O_5$ 367.12885. Found 367.12911.

Analysis of the C(6) regioisomer 2

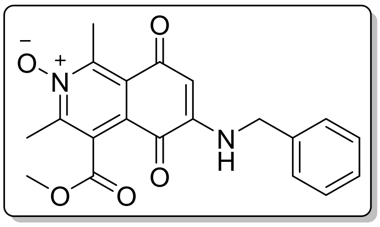

Orange solid, **2** (0.0144 g, 6%) (R_f: 0.4, Hexane-Ethyl acetate 1:1). m.p. 79 – 80 °C (Lit. 79.0 – 80.0 °C)[2]; ν_{max}/cm^{-1} (NaCl): 3334, 2923, 2852, 1739, 1671, 1616, 1574, 1516, 1453, 1435, 1373, 1315, 1280, 1231, 1143, 1070, 834, 811, 738; ^{1}H-NMR (300MHz, $CDCl_3$): δ 2.48 (s, 3H), 3.00 (s, 3H), 4.01 (s, 3H), 4.34 (d, *J* = 5.7 Hz, 2H), 5.79 (s, 1H), 6.02 (bs, 1H) and 7.24 – 7.41 (m, 5H) ppm; ^{13}C-NMR (75MHz, $CDCl_3$): δ 14.7, 15.9, 46.8, 53.3, 103.7, 127.6 [2C], 128.3, 129.1 [2C], 135.4, 146.2, 166.7, 179.1 and 182.4 ppm; *m/z* (ESI$^+$): 367.3 [M+H]$^+$, 60%; HRMS (ESI$^+$): Exact mass calculated for $C_{20}H_{19}N_2O_5$ 367.12885. Found 367.12870.

7-((4-Fluorobenzyl)amino)-4-(methoxycarbonyl)-1,3-dimethyl-5,8-dioxo-5,8-dihydroisoquinoline 2-oxide 4

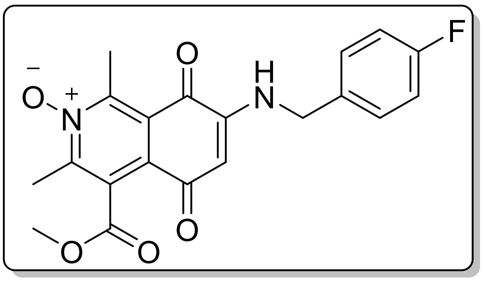

A suspension of 4-(methoxycarbonyl) -1,3-dimethyl-5,8-dioxo-5,8-dihydroiso-quinoline 2-oxide **3** (0.303 g, 1.160 mmol), cerium chloride heptahydrate (0.040 g, 0.107 mmol), 4-fluorobenzylamine (0.26 mL, 2.28 mmol) in absolute ethanol (30 mL) was stirred for two hours at room temperature. The consumption of **3** was monitored using TLC (1:1 Hexane: Ethyl acetate). The reaction mixture was concentrated under reduced pressure yielding a dark red oil, which was subjected to flash column chromatography (1:0 Hexane: Ethyl acetate – 1:1 Hexane: Ethyl acetate). The eluents were concentrated under reduced pressure to yield the product as a red oil. An ethyl acetate: hexane recrystallisation was performed to yield the product as a red solid, **4** (0.207 g, 46%) (R_f: 0.2, Hexane-Ethyl acetate 1:1). m.p. 90.0 – 92.0 °C; ν_{max}/cm^{-1} (KBr): 3371, 3009, 2952, 1737, 1684, 1614, 1541, 1509, 1313, 1282, 1226, 1157, 1069, 829; ^{1}H-NMR (300MHz, $CDCl_3$): δ 2.52 (s, 3H), 2.98 (s, 3H), 4.01 (s, 3H), 4.35 (d, *J* = 5.7 Hz, 2H), 5.73 (s, 1H), 6.30 (t, *J* = 5.7 Hz, 1H), 7.07 (t, *J* = 9.0 Hz, 2H) and 7.27 (t, *J* = 9.0 Hz, 2H) ppm; ^{13}C-NMR (75MHz, $CDCl_3$): δ 15.1, 16.3, 46.3, 53.3, 101.2, 116.1 (d, *J* = 21 Hz, 2C), 123.9, 124.6, 128.3, 129.3 (d, *J* = 8.2 Hz, 2C), 131.0 (d, *J* = 3.2 Hz), 147.9, 151.70, 151.73, 162.5 (d, *J* = 247.6 Hz), 166.9, 179.0 and 181.1 ppm; *m/z* (ESI): 385.2 $[M+H]^+$; HRMS (ESI): Exact mass calculated for $C_{20}H_{18}N_2O_5F$ 385.1200. Found 385.1182.

Analysis of the C(6) regioisomer 5:

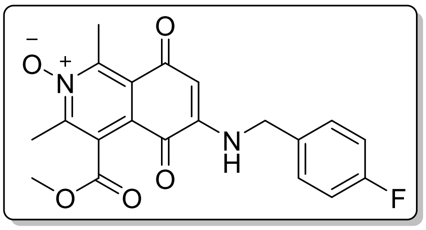

Red solid, **5** (0.027 g, 6%) (R_f: 0.4, Hexane-Ethyl acetate 1:1). m.p. 79.0-80.0 °C (degrad.); ν_{max}/cm^{-1} (NaCl): 3336, 3056, 3007, 2292, 2252, 1739, 1671, 1616, 1573, 1510, 1435, 1373, 1344, 1316, 1280, 1226, 1143, 1071, 828; ^{1}H-NMR (300MHz, $CDCl_3$): δ 2.48 (s, 3H), 2.99 (s, 3H), 4.02 (s, 3H), 4.32 (d, *J* = 5.7 Hz, 2H), 5.76 (s, 1H), 6.01 (t, *J* = 5.7 Hz, 1H), 7.07 (t, *J* = 9.0 Hz, 2H) and 7.27 (t, *J* = 9.0 Hz, 2H) ppm; ^{13}C-NMR (75MHz, $CDCl_3$): δ 14.8, 16.0, 46.1, 53.4, 103.8, 116.0 (d, *J* = 21 Hz, 2C), 120.9, 125.8, 128.0, 129.4 (d, *J* = 8.0 Hz, 2C), 131.2 (d, *J* = 3.1 Hz), 146.2, 149.2, 152.2, 162.5 (d, *J* = 247.1 Hz), 166.5, 179.0 and 182.4 ppm; *m/z* (ESI): 383.2 $[M-H]^-$; HRMS (ESI): Exact mass calculated for $C_{20}H_{18}N_2O_5F$ 385.1200. Found 385.11953.

7-((4-Methoxybenzyl)amino)-4-(methoxycarbonyl)-1,3-dimethyl-5,8-dioxo-5,8-dihydroisoquinoline 2-oxide 6

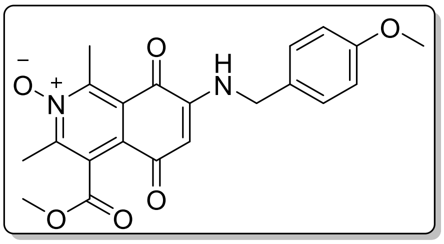

A suspension of 4-(methoxycarbonyl) -1,3-dimethyl-5,8-dioxo-5,8-dihydroiso-quinoline 2-oxide **3** (0.204 g, 0.78 mmol), cerium chloride heptahydrate (0.025 g, 0.067 mmol), 4-methoxybenzylamine (0.20 mL, 1.53 mmol) in absolute ethanol (20 mL) was stirred for three hours at room temperature. The consumption of **3** was monitored using TLC (1:1 Hexane: Ethyl acetate). The reaction mixture was concentrated under reduced pressure yielding a dark red oil, which was subjected to flash column chromatography (1:0 Hexane: Ethyl acetate – 1:1 Hexane: Ethyl acetate). The eluents were concentrated under reduced pressure to yield the product as a red oil. An ethyl acetate: hexane recrystallisation was performed to yield the product as a red solid, **6** (0.187 g, 60%) (R_f: 0.2, Hexane-Ethyl acetate 1:1). m.p. 94.0 – 95.0 °C; ν_{max}/cm^{-1} (KBr): 3384, 2952, 1738, 1684, 1612, 1541, 1513, 1312, 1284, 1229, 1068, 830; ^{1}H-NMR (300MHz, $CDCl_3$): δ 2.52 (s, 3H), 2.98 (s, 3H), 3.81 (s, 3H), 4.01 (s, 3H), 4.29 (d, *J* = 5.6 Hz, 2H), 5.76 (s, 1H), 6.25 (t, *J* = 5.6 Hz, 1H), 6.90 (d, *J* = 8.7 Hz, 2H) and 7.22 (d, *J* = 8.7 Hz, 2H) ppm; ^{13}C-NMR (75MHz, $CDCl_3$): δ 15.0, 16.3, 46.6, 53.3, 55.4, 101.0, 114.5 (2C), 123.9, 124.8, 127.2, 128.3, 129.1 (2C), 147.9, 151.62, 151.67, 159.7, 167.0, 178.9 and 181.1 ppm; *m/z* (ESI): 395.2 [M-H]$^-$; HRMS (ESI): Exact mass calculated for $C_{21}H_{21}N_2O_6$ 397.13941. Found 397.13949.

Analysis of the C(6) regioisomer 7:

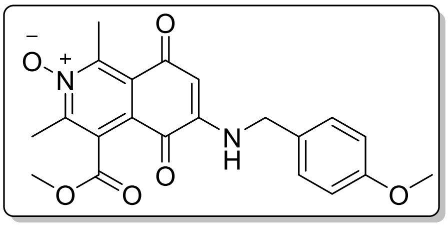

Red solid, **7** (0.025 g, 8%) (R_f: 0.6, Hexane-Ethyl acetate 1:1). m.p. 96.0 – 97.0 °C; ν_{max}/cm^{-1} (KBr): 3323, 2953, 1739, 1673, 1616, 1572, 1511, 1436, 1380, 1357, 1305, 1281, 1244, 1142, 1071, 1027, 833, 814; ^{1}H-NMR (300MHz, $CDCl_3$): δ 2.48 (s, 3H), 3.00 (s, 3H), 3.81 (s, 3H), 4.01 (s, 3H), 4.27 (d, *J* = 5.5 Hz, 2H), 5.80 (s, 1H), 5.95 (t, *J* = 5.7 Hz, 1H), 6.90 (d, *J* = 8.7 Hz, 2H) and 7.22 (d, *J* = 8.7 Hz, 2H) ppm; ^{13}C-NMR (75MHz, $CDCl_3$): δ 14.8, 15.9, 46.4, 53.4, 55.3, 103.5, 114.5 (2C), 121.0, 125.9, 127.4, 127.9, 129.1 (2C), 146.2, 149.0, 152.2, 159.6, 166.6, 179.1 and 182.4 ppm; *m/z* (ESI): 395.2 [M-H]$^-$; HRMS (ESI): Exact mass calculated for $C_{21}H_{21}N_2O_6$ 397.13941. Found 397.13962.

7-((3-Methoxybenzyl)amino)-4-(methoxycarbonyl)-1,3-dimethyl-5,8-dioxo-5,8-dihydroisoquinoline 2-oxide 8

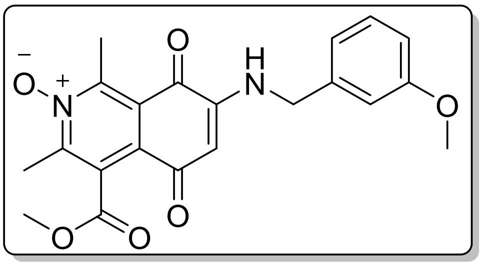

A suspension of 4-(methoxycarbonyl) -1,3-dimethyl-5,8-dioxo-5,8-dihydroiso-quinoline 2-oxide **3** (0.201 g, 0.769 mmol), cerium chloride heptahydrate (0.025 g, 0.067 mmol), 3-methoxybenzylamine (0.20 mL, 1.53 mmol) in absolute ethanol (20 mL) was stirred for three hours at room temperature. The consumption **3** was monitored using TLC (1:1 Hexane: Ethyl acetate). The reaction mixture was concentrated under reduced pressure yielding a dark red oil, which was subjected to flash column chromatography (1:0 Hexane: Ethyl acetate – 1:1 Hexane: Ethyl acetate). The eluents were concentrated under reduced pressure to yield the product as a red oil. An ethyl acetate: hexane recrystallisation was performed to yield the product as a red solid, **8** (0.184 g, 60%) (R_f: 0.2, Hexane-Ethyl acetate 1:1). m.p. 82.0 – 84.0 °C; ν_{max}/cm^{-1} (KBr): 3351, 3004, 2950, 2837, 1737, 1684, 1614, 1541, 1513, 1491, 1455, 1436, 1312, 1283, 1227, 1067, 773; ^{1}H-NMR (300MHz, $CDCl_3$): δ 2.52 (s, 3H), 2.99 (s, 3H), 3.81 (s, 3H), 4.01 (s, 3H), 4.34 (d, *J* = 5.7 Hz, 2H), 5.74 (s, 1H), 6.34 (t, *J* = 5.7 Hz, 1H), 6.83 (d, *J* = 9.0 Hz, 2H), 6.88 (s, 1H), 7.31 (t, *J* = 9.0 Hz, 1H) ppm; ^{13}C-NMR (75MHz, $CDCl_3$): δ 15.1, 16.3, 46.9, 53.3, 55.3, 101.2, 113.40, 113.46, 119.7, 123.9, 124.7, 128.3, 130.2, 136.8, 148.0, 151.63, 151.70, 160.2, 167.0, 178.9 and 181.1 ppm; *m/z* (ESI): 395.2 $[M-H]^-$; HRMS (ESI): Exact mass calculated for $C_{21}H_{21}N_2O_6$ 397.13941. Found 397.13926.

Analysis of the C(6) regioisomer 9:

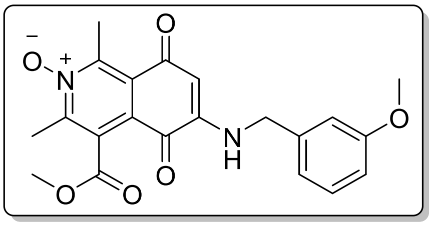

Dark red solid, **9** (0.020 g, 7%) (R_f: 0.4, Hexane-Ethyl acetate 1:1). m.p. 174.0-176.0 °C (degrad.); ν_{max}/cm^{-1} (NaCl): 3355, 3054, 2954, 2838, 1738, 1672, 1615, 1574, 1518, 1490, 1435, 1343, 1316, 1280, 1140, 1071, 810; ^{1}H-NMR (300MHz, $CDCl_3$): δ 2.48 (s, 3H), 3.00 (s, 3H), 3.81 (s, 3H), 4.02 (s, 3H), 4.32 (d, *J* = 5.7 Hz, 2H), 5.78 (s, 1H), 6.04 (t, *J* = 5.7 Hz, 1H), 6.84 (d, *J* = 9.0, 2H), 6.88 (s, 1H), 7.30 (d, *J* = 9.0 Hz, 1H) ppm; ^{13}C-NMR (75MHz, $CDCl_3$): δ 14.8, 16.0, 46.8, 53.4, 55.3, 103.8, 113.4 (2C), 119.8, 121.0, 125.9, 127.9, 130.2, 137.0, 146.3, 149.1, 152.2, 160.2, 166.6, 179.0 and 182.4 ppm; *m/z* (ESI): 395.2 $[M-H]^-$; HRMS (ESI): Exact mass calculated for $C_{21}H_{21}N_2O_6$ 397.13941. Found 397.13929.

7-((4-Chlorobenzyl)amino)-4-(methoxycarbonyl)-1,3-dimethyl-5,8-dioxo-5,8-dihydroisoquinoline 2-oxide 10

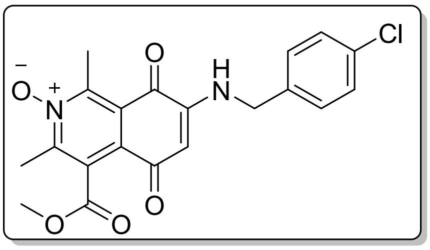

A suspension of 4-(methoxycarbonyl) -1,3-dimethyl-5,8-dioxo-5,8-dihydroiso-quinoline 2-oxide **3** (0.204 g, 0.781 mmol), cerium chloride heptahydrate (0.022 g, 0.059 mmol), 4-chlorobenzylamine (0.19 mL, 1.56 mmol) in absolute ethanol (20 mL) was stirred for four hours at room temperature. The consumption of **3** was monitored using TLC (1:1 Hexane: Ethyl acetate). The reaction mixture was concentrated under reduced pressure yielding a dark red oil, which was subjected to flash column chromatography (1:0 Hexane: Ethyl acetate – 1:1 Hexane: Ethyl acetate). The eluents were concentrated under reduced pressure to yield the product as a red oil. An ethyl acetate: hexane recrystallisation was performed to yield the product as a dark orange solid, **10** (0.151 g, 48%) (R_f: 0.2, Hexane-Ethyl acetate 1:1). m.p. 205.0 – 206.0 °C; ν_{max}/cm^{-1} (NaCl): 3302, 3050, 2926, 2304, 1736, 1613, 1536, 1490, 1435, 1407, 1308, 1228, 1090, 1014, 801; ^{1}H-NMR (300MHz, $CDCl_3$): δ 2.52 (s, 3H), 2.99 (s, 3H), 4.01 (s, 3H), 4.36 (d, *J* = 5.8 Hz, 2H), 5.70 (s, 1H), 6.31 (t, *J* = 5.8 Hz, 1H), 7.22 (d, *J* = 9.0 Hz, 2H) and 7.35 (d, *J* = 9.0 Hz, 2H) ppm; ^{13}C-NMR (75MHz, $CDCl_3$): δ 15.1, 16.3, 46.3, 53.3, 101.4, 123.9, 124.5, 128.3, 128.8 (2C), 129.3 (2C), 133.8, 134.3, 147.9, 151.70, 151.73, 166.9, 179.0 and 181.1 ppm; *m/z* (ESI): 399.2 $[M-H]^-$, 401.1 $[M-H]^-$; HRMS (ESI): Exact mass calculated for $C_{20}H_{18}{}^{35}ClN_2O_5$ 401.08988. Found 401.08983.

Analysis of the C(6) regioisomer 11:

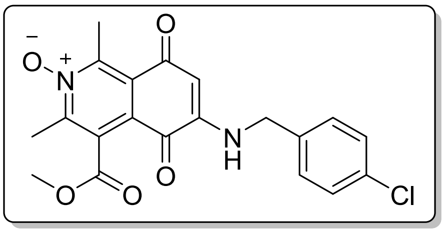

Red solid, **11** (0.017 g, 5%) (R_f: 0.4, Hexane-Ethyl acetate 1:1). m.p. 154.0 – 156.0 °C; ν_{max}/cm^{-1} (NaCl): 3351, 2923, 2852, 1739, 1671, 1616, 1573, 1516, 1492, 1435, 1373, 1315, 1279, 1232, 1144, 1071, 1014, 811; ^{1}H-NMR (300MHz, $CDCl_3$): δ 2.48 (s, 3H), 2.99 (s, 3H), 4.02 (s, 3H), 4.33 (d, *J* = 5.7 Hz, 2H), 5.74 (s, 1H), 6.01 (t, *J* = 5.7 Hz, 1H), 7.22 (d, *J* = 9.0 Hz, 2H) and 7.35 (d, *J* = 9.0 Hz, 2H); ^{13}C-NMR (75MHz, $CDCl_3$): δ 14.8, 16.0, 46.1, 53.4, 104.0, 120.9, 125.8, 128.6, 128.9 (2C), 129.3 (2C), 133.9, 134.2, 146.2, 149.2, 152.3, 166.5, 179.0 and 182.4 ppm; *m/z* (ESI): 399.2 $[M-H]^-$, 401.2 $[M-H]^-$; HRMS (ESI): Exact mass calculated for $C_{20}H_{18}{}^{35}ClN_2O_5$ 401.08988. Found 401.08988.

7-((3-Chlorobenzyl)amino)-4-(methoxycarbonyl)-1,3-dimethyl-5,8-dioxo-5,8-dihydroisoquinoline 2-oxide 12

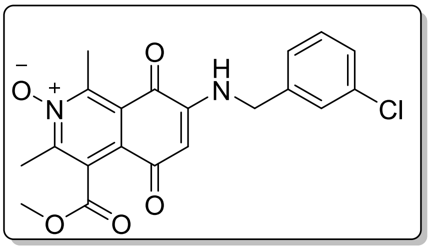

A suspension of 4-(methoxycarbonyl) -1,3-dimethyl-5,8-dioxo-5,8-dihydroiso-quinoline 2-oxide **3** (0.207 g, 0.792 mmol), cerium chloride heptahydrate (0.026 g, 0.070 mmol), 3-chloro benzylamine (0.19 mL, 1.55 mmol) in absolute ethanol (20 mL) was stirred for four hours at room temperature. The consumption of **3** was monitored using TLC (1:1 Hexane: Ethyl acetate). The reaction mixture was concentrated under reduced pressure yielding a dark red oil, which was subjected to flash column chromatography (1:0 Hexane: Ethyl acetate – 1:1 Hexane: Ethyl acetate). The eluents were concentrated under reduced pressure to yield the product as a red oil. An ethyl acetate: hexane recrystallisation was performed to yield the product as a red solid, **12** (0.178 g, 56%) (R_f: 0.2, Hexane-Ethyl acetate 1:1). m.p. 197.0 – 198.0 °C; ν_{max}/cm^{-1} (KBr): 3323, 2949, 1748, 1733, 1681, 1609, 1576, 1433, 1391, 1352, 1316, 1282, 1226, 1066, 832, 776; ^{1}H-NMR (300MHz, $CDCl_3$): δ 2.52 (s, 3H), 3.00 (s, 3H), 4.01 (s, 3H), 4.37 (d, *J* = 6.0 Hz, 2H), 5.70 (s, 1H), 6.36 [t, *J* = 6.0 Hz, 1H], 7.17 (t, *J* = 8.0 Hz, 1H), 7.29 (d, *J* = 8.0 Hz, 2H) and 7.30 (s, 1H) ppm; ^{13}C-NMR (75MHz, $CDCl_3$): δ 15.1, 16.3, 46.3, 53.3, 101.5, 123.9, 124.5, 125.5, 127.5, 128.3, 128.6, 130.4, 135.1, 137.4, 147.9, 151.70, 151.76, 166.9, 179.0 and 181.0 ppm; *m/z* (ESI): 401.1 $[M+H]^+$, 403.1 $[M+H]^+$; HRMS (ESI): Exact mass calculated for $C_{20}H_{18}{}^{35}ClN_2O_5$ 401.08988. Found 401.08989.

Analysis of the C(6) regioisomer 13:

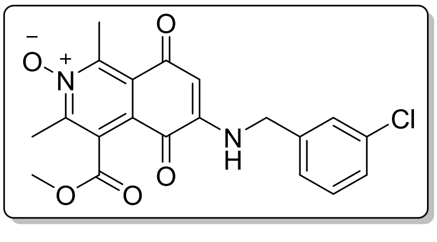

Red solid, **13** (0.011 g, 3%) (R_f: 0.5, Hexane-Ethyl acetate 1:1). m.p. 82 – 84 °C; ν_{max}/cm^{-1} (NaCl): 3315, 2923, 2853, 1736, 1683, 1613, 1540, 1512, 1433, 1373, 1346, 1311, 1282, 1228, 1164, 1067, 832, 774; ^{1}H-NMR (300MHz, $CDCl_3$): δ 2.48 (s, 3H), 2.99 (s, 3H), 4.03 (s, 3H), 4.35 (d, *J* = 6.0 Hz, 2H), 5.74 (s, 1H), 6.06 (t, *J* = 6.0 Hz, 1H), 7.17 (t, *J* = 8.0 Hz, 1H), 7.29 (d, *J* = 8.0 Hz, 2H) and 7.30 (s, 1H) ppm; ^{13}C-NMR (75MHz, $CDCl_3$): δ 14.8, 16.0, 46.2, 53.4, 104.0, 120.9, 125.5, 125.7, 127.5, 128.0, 128.5, 130.4, 135.1, 137.5, 146.2, 149.2, 152.3, 166.5, 178.9 and 182.4 ppm; *m/z* (ESI): 399.3 $[M-H]^-$, 401.3 $[M-H]^-$; HRMS (ESI): Exact mass calculated for $C_{20}H_{18}{}^{35}ClN_2O_5$ 401.08988. Found 401.08927.

7-((4-Bromobenzyl)amino)-4-(methoxycarbonyl)-1,3-dimethyl-5,8-dioxo-5,8-dihydroisoquinoline 2-oxide 14

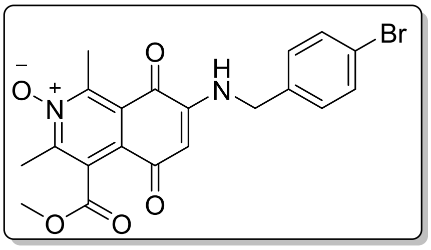

A suspension of 4-(methoxycarbonyl) -1,3-dimethyl-5,8-dioxo-5,8-dihydroiso-quinoline 2-oxide **3** (0.5031 g, 1.926 mmol), cerium chloride heptahydrate (0.085 g, 0.228 mmol), 4-bromobenzylamine (0.7221 g, 3.881 mmol) in absolute ethanol (50 mL) was stirred for four hours at room temperature. The consumption of **3** was monitored using TLC (1:1 Hexane: Ethyl acetate). The reaction mixture was concentrated under reduced pressure yielding a red oil, which was subjected to flash column chromatography (1:0 Hexane: Ethyl acetate – 1:1 Hexane: Ethyl acetate). The eluents were concentrated under reduced pressure to yield the product as a red oil. An ethyl acetate: hexane recrystallisation was performed to yield the product as an orange solid, **14** (0.4158 g, 49%) (R_f: 0.3, Hexane-Ethyl acetate 1:1). m.p. 195.0 – 196.0 °C; ν_{max}/cm^{-1} (KBr): 3362, 2956, 2919, 2850, 1722, 1680, 1612, 1544, 1505, 1487, 1307, 1282, 1246, 1071, 1009, 829; ^{1}H-NMR (300MHz, $CDCl_3$): δ 2.52 (s, 3H), 2.99 (s, 3H), 4.01 (s, 3H), 4.34 (d, *J* = 5.9 Hz, 2H), 5.70 (s, 1H), 6.31 (t, *J* = 5.9 Hz, 1H), 7.16 (d, *J* = 9.0 Hz, 2H) and 7.50 (d, *J* = 9.0 Hz, 2H) ppm; ^{13}C-NMR (75MHz, $CDCl_3$): δ 15.1, 16.3, 46.3, 53.3, 101.4, 122.3, 123.9, 124.5, 128.3, 129.1 (2C), 132.3 (2C), 134.3, 147.9, 151.72, 151.75, 166.9, 179.0 and 181.1 ppm; *m/z* (ESI): 445.1 $[M+H]^+$, 447.1 $[M+H]^+$; HRMS (ESI): Exact mass calculated for $C_{20}H_{18}N_2O_5{}^{79}Br$ 445.03936. Found 445.03962.

Analysis of the C(6) regioisomer 15:

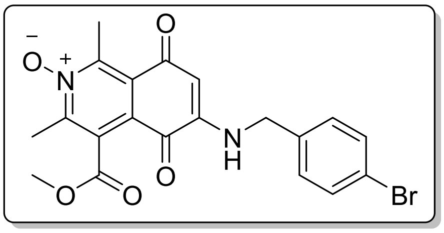

Red solid, **15** (0.0674 g, 8%) (R_f: 0.6, Hexane-Ethyl acetate 1:1). m.p. 133.0 – 135.0 °C; ν_{max}/cm^{-1} (NaCl): 3437, 3334, 1737, 1676, 1616, 1573, 1519, 1382, 1346, 1319, 1280, 1235, 1072, 811; ^{1}H-NMR (300MHz, $CDCl_3$): δ 2.48 (s, 3H), 2.99 (s, 3H), 4.02 (s, 3H), 4.32 (d, *J* = 6.0 Hz, 2H), 5.74 (s, 1H), 6.01 (bs, 1H), 7.16 (d, *J* = 9.0 Hz, 2H) and 7.50 (d, *J* = 9.0 Hz, 2H) ppm; ^{13}C-NMR (75MHz, $CDCl_3$): δ 14.8, 16.0, 46.2, 53.4, 104.0, 122.3, 129.1 (2C), 131.9, 132.3 (2C), 134.4, 137.3, 139.9, 146.2, 149.2, 152.3, 166.5, 178.9, and 182.4 ppm; *m/z* (ESI): 443.1 $[M-H]^-$, 445.1 $[M-H]^-$; HRMS (ESI): Exact mass calculated for $C_{20}H_{18}N_2O_5Br^{79}$ 445.03936. Found 445.03949.

7-((3,4-Difluorobenzyl)amino)-4-(methoxycarbonyl)-1,3-dimethyl-5,8-dioxo-5,8-dihydroisoquinoline 2-oxide 16

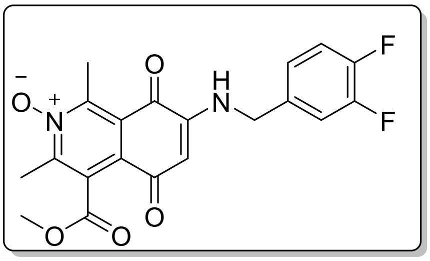

A suspension of 4-(methoxycarbonyl) -1,3-dimethyl-5,8-dioxo-5,8-dihydroiso-quinoline 2-oxide **3** (0.201 g, 0.769 mmol), cerium chloride heptahydrate (0.025 g, 0.067 mmol), 3,4-difluorobenzylamine (0.18 mL, 1.52 mmol) in absolute ethanol (20 mL) was stirred for three hours at room temperature. The consumption **3** was monitored using TLC (1:1 Hexane: Ethyl acetate). The reaction mixture was concentrated under reduced pressure yielding a dark red oil, which was subjected to flash column chromatography (1:0 Hexane: Ethyl acetate – 1:1 Hexane: Ethyl acetate). The eluents were concentrated under reduced pressure to yield the product as a red oil. An ethyl acetate: hexane recrystallisation was performed to yield the product as an orange solid, **16** (0.155 g, 50%) (R_f: 0.2, Hexane-Ethyl acetate 1:1). m.p. 159.0 – 160.0 °C; ν_{max}/cm^{-1} (KBr): 3331, 3022, 2954, 1742, 1681, 1609, 1517, 1435, 1316, 1282, 1233, 1069; ^{1}H-NMR (300MHz, $CDCl_3$): δ 2.52 (s, 3H), 2.99 (s, 3H), 4.01 (s, 3H), 4.36 (d, *J* = 5.7 Hz, 2H), 5.68 (s, 1H), 6.32 (t, *J* = 5.7 Hz, 1H) and 6.98 – 7.22 (m, 3H) ppm; ^{13}C-NMR (75MHz, $CDCl_3$): δ 15.1, 16.3, 45.9, 53.3, 101.6, 116.7 (d, *J* = 17.7 Hz), 118.0 (d, *J* = 17.7 Hz), 123.40 (dd, *J* = 6.6, 3.8 Hz), 123.9, 124.4, 128.4, 132.3 (dd, *J* = 6.6, 3.8 Hz), 147.8, 150.2 (dd, *J* = 249.9, 12.9 Hz), 150.7 (dd, *J* = 249.9, 12.9 Hz), 151.7, 151.8, 166.8, 179.0 and 181.0 ppm; m/z (ESI): 403.2 $[M+H]^+$; HRMS (ESI): Exact mass calculated for $C_{20}H_{17}F_2N_2O_5$ 403.11000. Found 403.11043.

Analysis of the C(6) regioisomer 17:

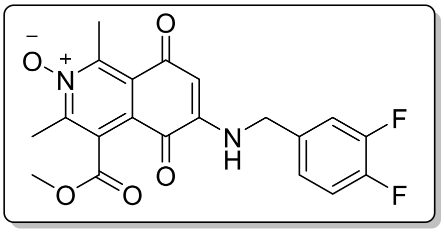

Orange solid, **17** (0.0154 g, 5%) (R_f: 0.5, Hexane-Ethyl acetate 1:1). m.p. 89.0 – 91.0 °C; ν_{max}/cm^{-1} (KBr): 3333, 3055, 2955, 2854, 2405, 1739, 1671, 1616, 1574, 1517, 1435, 1344, 1316, 1280, 1233, 1154, 1115, 1072, 811; ^{1}H-NMR (300MHz, $CDCl_3$): δ 2.48 (s, 3H), 2.99 (s, 3H), 4.03 (s, 3H), 4.33 (d, *J* = 5.8 Hz, 2H), 5.72 (s, 1H), 6.02 (t, *J* = 5.7 Hz, 1H) and 6.98 – 7.22 (m, 3H) ppm; ^{13}C-NMR (150MHz, $CDCl_3$): δ 14.8, 16.0, 45.7, 53.5, 104.1, 116.5 (d, *J* = 17.7Hz), 118.0 (d, *J* = 17.7Hz), 120.8, 123.5 (dd, *J* = 6.4, 3.6 Hz), 125.7, 128.0, 132.4 (dd, *J* = 6.4, 3.6 Hz), 146.1, 149.3, 150.1 (dd, *J* = 249.9, 13.0 Hz), 150.7 (dd, *J* = 249.9, 13.0 Hz), 152.3, 166.5, 178.9 and 182.4 ppm; *m/z* (ESI): 401.2 $[M-H]^-$; HRMS (ESI): Exact mass calculated for $C_{20}H_{17}F_2N_2O_5$ 403.11000. Found 403.10988.

7-((3-Chloro-4-fluorobenzyl)amino)-4-(methoxycarbonyl)-1,3-dimethyl-5,8-dioxo-5,8-dihydroisoquinoline 2-oxide 18

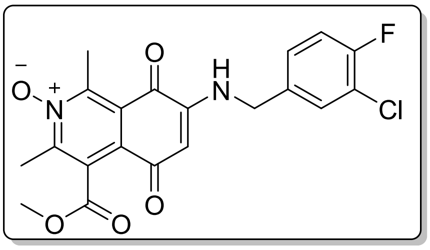

A suspension of 4-(methoxycarbonyl) -1,3-dimethyl-5,8-dioxo-5,8-dihydroiso-quinoline 2-oxide **3** (0.203 g, 0.78 mmol), cerium chloride heptahydrate (0.026 g, 0.070 mmol), 3-chloro-4-fluorobenzylamine (0.20 mL, 1.55 mmol) in absolute ethanol (20 mL) was stirred for three hours at room temperature. The consumption of **3** was monitored using TLC (1:1 Hexane: Ethyl acetate). The reaction mixture was concentrated under reduced pressure yielding a dark red oil, which was subjected to flash column chromatography (1:0 Hexane: Ethyl acetate – 1:1 Hexane: Ethyl acetate). The eluents were concentrated under reduced pressure to yield the product as a red oil. An ethyl acetate: hexane recrystallisation was performed to yield the product as a dark orange solid, **18** (0.192 g, 60%) (R_f: 0.2, Hexane-Ethyl acetate 1:1). m.p. 103.0 – 105.0 °C; ν_{max}/cm^{-1} (KBr): 3362, 3035, 2952, 1738, 1684, 1614, 1541, 1500, 1436, 1410, 1374, 1313, 1283, 1228, 1071, 830, 773; ^{1}H-NMR (300MHz, $CDCl_3$): δ 2.52 (s, 3H), 2.99 (s, 3H), 4.01 (s, 3H), 4.35 (d, *J* = 5.7 Hz, 2H), 5.68 (s, 1H), 6.32 (t, *J* = 5.7 Hz, 1H), 7.15 (d, *J* = 9.0 Hz, 2H), and 7.34 (d, *J* = 7.0 Hz, 1H) ppm; ^{13}C-NMR (75MHz, $CDCl_3$): δ 15.1, 16.3, 45.8, 53.3, 101.6, 117.3 (d, *J* = 19.1 Hz), 121.9 (d, *J* = 19.1 Hz), 123.9, 124.4, 127.3 (d, *J* = 7.2 Hz), 128.3, 129.6 (d, *J* = 1.5 Hz, 132.4 (d, *J* = 4.0 Hz), 147.8, 151.76, 151.79, 157.9 (d, *J* = 250.4 Hz), 166.8, 179.0 and 181.0 ppm; *m/z* (ESI): 419.0 $[M+H]^+$, 421.0 $[M+H]^+$; HRMS (ESI): Exact mass calculated for $C_{20}H_{17}{}^{35}ClFN_2O_5$ 419.08045. Found 419.08048.

Analysis of the C(6) regioisomer 19:

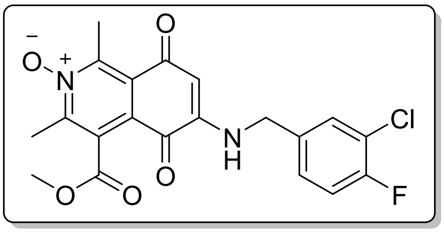

Dark red solid, **19** (0.021 g, 6%) (R_f: 0.5, Hexane-Ethyl acetate 1:1). m.p. 89.0 – 90.0 °C; ν_{max}/cm^{-1} (NaCl): 3353, 3055, 2953, 2855, 2407, 1739, 1672, 1616, 1574, 1500, 1435, 1373, 1316, 1279, 1246, 1148, 1072, 812; ^{1}H-NMR (300MHz, $CDCl_3$): δ 2.40 (s, 3H), 2.90 (s, 3H), 3.95 (s, 3H), 4.26 (d, *J* = 5.7 Hz, 2H), 5.64 (s, 1H), 6.03 (t, *J* = 5.7 Hz, 1H), 7.07 (t, *J* = 6.0 Hz, 2H) and 7.26 (dd, *J* = 6.9, 1.7 Hz, 1H) ppm; ^{13}C-NMR (75MHz, $CDCl_3$): δ 14.8, 15.9, 45.6, 53.4, 104.0, 117.2 (d, *J* = 21.4 Hz), 120.8, 121.8 (d, *J* = 18.2

Hz), 125.7, 127.2 (d, J = 7.4 Hz), 128.0, 129.6 (d, J = 1.4 Hz), 132.6 (d, J = 4.0 Hz), 146.1, 149.2, 152.2, 157.8 (d, J = 250.1 Hz), 166.5, 178.9 and 182.4 ppm; *m/z* (ESI): 417.2 $[M-H]^-$, 419.1 $[M-H]^-$; HRMS (ESI): Exact mass calculated for $C_{20}H_{17}{}^{35}ClFN_2O_5$ 419.08045. Found 419.07997.

7-((4-Hydroxybenzyl)amino)-4-(methoxycarbonyl)-1,3-dimethyl-5,8-dioxo-5,8-dihydroisoquinoline 2-oxide 20

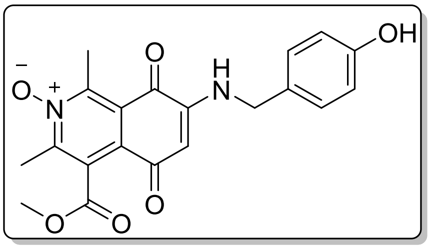

A suspension of 4-(methoxycarbonyl) -1,3-dimethyl-5,8-dioxo-5,8-dihydroiso-quinoline 2-oxide **3** (0.206 g, 0.789 mmol), cerium chloride heptahydrate (0.025 g, 0.067 mmol), 4-hydroxybenzylamine (0.20 g, 1.62 mmol) in absolute ethanol (20 mL) was stirred for three hours at room temperature. The consumption of **3** was monitored using TLC (1:1 Hexane: Ethyl acetate). The reaction mixture was concentrated under reduced pressure yielding a dark red oil, which was subjected to flash column chromatography (1:0 Hexane: Ethyl acetate – 1:1 Hexane: Ethyl acetate). The eluents were concentrated under reduced pressure to yield the product as a red oil. An ethyl acetate: hexane recrystallisation was performed to yield the product as an orange solid, **20** (0.088 g, 30%) (R_f: 0.2, Hexane-Ethyl acetate 1:1). m.p. 116.0 – 118.0 °C; ν_{max}/cm^{-1} (KBr): 3354, 2958, 1731, 1688, 1541, 1514, 1453, 1354, 1309, 1246, 1224, 1067, 825; ^{1}H-NMR (300MHz, DMSO-d_6): δ 2.34 (s, 3H), 2.82 (s, 3H), 3.85 (s, 3H), 4.32 (d, J = 6.2 Hz, 2H), 5.57 (s, 1H), 6.71 (d, J = 8.2 Hz, 2H), 7.15 (d, J = 8.2 Hz, 2H), 8.34 (t, J = 6.2 Hz, 1H) and 9.33 (s, 1H) ppm; ^{13}C-NMR (75 MHz, DMSO-d_6): δ 16.2, 16.3, 45.2, 53.2, 99.5, 115.6 (2C), 124.5, 125.2, 127.53, 127.56, 129.0 (2C), 149.8, 149.9, 150.8, 157.0, 167.0, 178.2 and 181.5 ppm; *m/z* (ESI): 381.3 $[M-H]^-$; HRMS (ESI): Exact mass calculated for $C_{20}H_{19}N_2O_6$ 383.12376. Found 383.12293.

Methyl 3-methyl-5,8-dioxo-5,8-dihydroisoquinoline-4-carboxylate 22

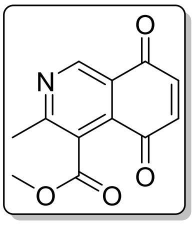

A suspension of 2,5-dihydroxybenzaldehyde **21** (0.503 g, 3.64 mmol), silver (I) oxide (1.622 g, 6.99 mmol), methyl 3-aminocrotonate (0.421 g, 3.657 mmol) and magnesium sulfate (1.667 g) was stirred vigorously at room temperature in dichloromethane (90 mL) resulting in a black suspension. The reaction mixture was stirred for 18 hours at room temperature until reaction completion, confirmed by TLC (1:1 Hexane: Ethyl acetate), resulting in a dark orange solution with dark

grey precipitate. The crude reaction mixture was concentrated under reduced pressure yielding a crude black solid which was subjected to flash column chromatography (1:0 Hexane: Ethyl acetate – 7:3 Hexane: Ethyl acetate) yielding the product as a yellow solid, **22** (0.453 g, 54%) (R_f: 0.8, Hexane-Ethyl acetate 1:1). m.p. 109.0 – 110.0 °C (Lit. 109.0 – 111.5 °C)[3]; ν_{max}/cm^{-1} (KBr): 3449, 3021, 2961, 1738, 1673, 1577, 1433, 1369, 1318, 1300, 1253, 1222, 1107, 1080, 853; ^1H-NMR (300MHz, $CDCl_3$): δ 2.69 (s, 3H), 4.05 (s, 3H), 7.02 (s, 2H) and 9.28 (s, 1H) ppm; *m/z* (ESI): 232.3 $[M+H]^+$.

4-(Methoxycarbonyl)-3-methyl-5,8-dioxo-5,8-dihydroisoquinoline 2-oxide 23

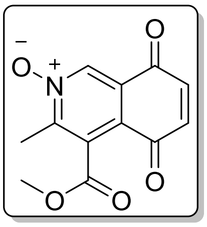

Methyl 3-methyl-5,8-dioxo-5,8-dihydroisoquinoline-4-carboxylate **22** (0.401 g, 1.734 mmol) was dissolved in dichloromethane (8 mL) with stirring resulting in a dark yellow solution. *meta*-Chloroperoxybenzoic acid (0.603 g, 3.494 mmol) was then added resulting in an orange solution. The reaction was stirred at room temperature for 24 hours resulting in a dark orange suspension. TLC analysis was undertaken at this point to ensure reaction completion (1:1 Hexane: Ethyl acetate). The reaction mixture was concentrated under reduced pressure yielding a crude orange oil. Column chromatography was performed on this crude material (1:0 Hexane: Ethyl acetate – 7:3 Hexane: Ethyl acetate) yielding the product as an orange solid, **23** (0.332 g, 77%) (R_f: 0.4, Hexane-Ethyl acetate 1:1). m.p. 147 – 148 °C (degrad.); ν_{max}/cm^{-1} (KBr): 3469, 3097, 3023, 2957, 2924, 1744, 1683, 1666, 1590, 1432, 1376, 1336, 1299, 1222, 1090, 1054, 856; ^1H-NMR (300MHz, $CDCl_3$): δ 2.50 (d, *J* = 0.40 Hz, 3H), 4.06 (s, 3H), 7.02 (s, 2H) and 8.76 (d, *J* = 0.40 Hz, 1H) ppm; ^{13}C-NMR (75MHz, $CDCl_3$): δ 15.2, 53.7, 121.9, 127.2, 130.6, 136.9, 137.9, 139.5, 152.3, 165.4, 180.8, 181.6; *m/z* (ESI): 248.3 $[M+H]^+$; HRMS (ESI): Exact mass calculated for $C_{12}H_{10}NO_5$ 248.0559. Found 248.0567.

7-(Benzylamino)-4-(methoxycarbonyl)-3-methyl-5,8-dioxo-5,8-dihydroisoquinoline 2-oxide 24

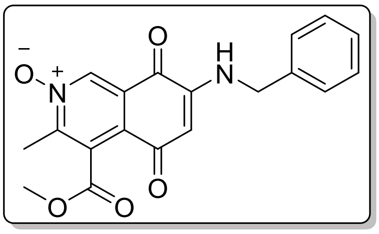

A suspension of 4-(methoxycarbonyl)-3-methyl-5,8-dioxo-5,8-dihydroisoquinoline 2-oxide **23** (0.071 g, 0.287 mmol), cerium chloride heptahydrate (0.035 g, 0.093 mmol), benzylamine (0.060 mL, 0.551 mmol) in absolute ethanol (8 mL) was stirred for two hours at room temperature. The consumption of **23** was monitored using TLC (1:1

Hexane: Ethyl acetate). TLC confirmed the presence of both regioisomers. The reaction mixture was concentrated under reduced pressure yielding a dark red oil, which was subjected to flash column chromatography (0:1 Ethyl acetate: Dichloromethane – 3:7 Ethyl acetate: Dichloromethane). The eluents were concentrated under reduced pressure to yield the product as a red solid, **24** (0.0305 g, 30%) (R_f: 0.2, Hexane-Ethyl acetate 1:1). m.p. 195 – 196 °C (degrad.). ν_{max}/cm^{-1} (KBr): 3251, 2954, 2923, 2854, 1739, 1693, 1605, 1549, 1515, 1433, 1351, 1331, 1272, 1062; ^{1}H-NMR (400MHz, $CDCl_3$): δ 2.49 (s, 3H), 4.03 (s, 3H), 4.38 (d, *J* = 5.5 Hz, 2H), 5.78 (s, 1H), 6.27 (bs, 1H), 7.27-7.43 (m, 5H) and 8.77 (s, 1H) ppm; ^{13}C-NMR (100 MHz, $CDCl_3$): 15.4, 47.0, 53.6, 102.6, 124.3, 126.2, 127.7 (2C), 128.4, 129.1 (2C), 130.7, 135.1, 136.5, 147.1, 152.7, 166.2, 178.8 and 179.0 ppm; *m/z* (ESI): 353.3 $[M+H]^+$; HRMS (ESI): Exact mass calculated for $C_{19}H_{17}N_2O_5$ 353.1137. Found 353.1125.

Analysis of the C(6) regioisomer 25:

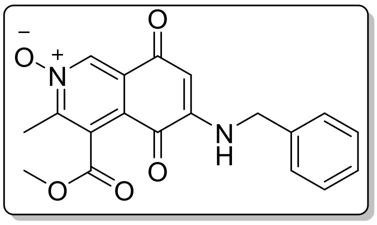

Dark red solid, **25** (0.0517 g, 51%) (R_f: 0.3, Hexane-Ethyl acetate 1:1). m.p. 90 – 92 °C; ν_{max}/cm^{-1} (KBr): 3306, 2954, 2924, 2854, 1739, 1677, 1606, 1581, 1513, 1453, 1437, 1354, 1321, 1261, 1119, 1069; ^{1}H-NMR (400MHz, $CDCl_3$): δ 2.46 (s, 3H), 4.03 (s, 3H), 4.38 (d, *J* = 5.5 Hz, 2H), 5.82 (s, 1H), 6.30 (bs, 1H), 7.27-7.42 (m, 5H) and 8.76 (s, 1H) ppm; ^{13}C-NMR (100 MHz, $CDCl_3$): δ 15.0, 47.0, 53.6, 101.5, 120.6, 127.7 (2C), 128.5, 128.8, 129.2 (2C), 130.2, 135.0, 137.4, 147.9, 150.2, 165.8, 178.0 and 178.8 ppm; *m/z* (ESI): 353.3 $[M+H]^+$; HRMS (ESI): Exact mass calculated for $C_{19}H_{17}N_2O_5$ 353.1137. Found 353.1131.

S1.2 Structural confirmation of substitution at the C-6 or C-7 position

^{1}H NMR analysis identified that across the series, the C(6) regioisomer amine proton experiences a greater electron shielding effect relative to the C(7) regioisomer. 2D ^{1}H-^{13}C NMR analysis identified the ^{1}H NMR amine signals for the C(7) regioisomers ranged between 6.20-6.35 ppm, and the C(6) isomer amine signal ranged between 5.97-6.06 ppm. The ^{1}H NMR spectra for **16** and **17** are superimposed in **Figure S1**illustrating the shielding of the amine proton of **17**.

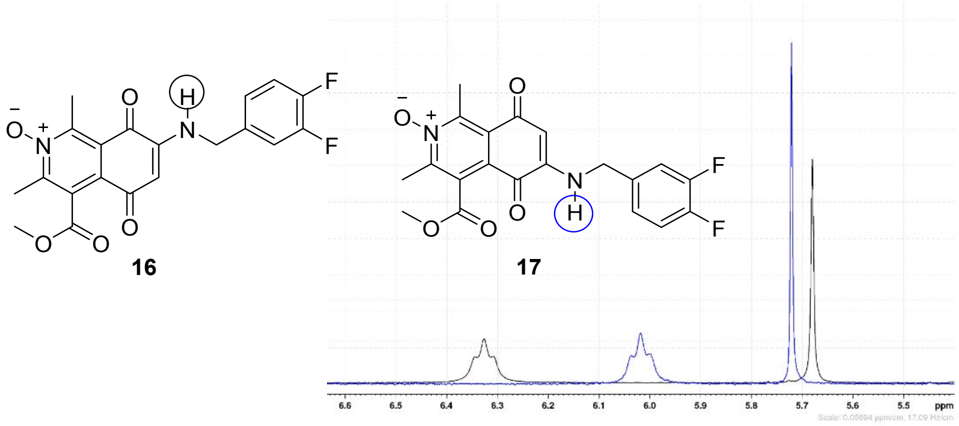

Figure S1 *Superimposed ^{1}H NMR spectra of IQQ **16** (black) and IQQ **17**(blue) focusing on amine proton region.*

S1.3 NMR structural confirmation of 25

^{1}H-^{13}C 2D NMR was used to characterise the C(6) isomer **25**. Correlations of C(7)H to both C(1) and C(5) were observed (**Figure S2**). The C(7)H-C(5) correlation has been previously reported method to distinguish IQQ regioisomers post amine addition.[4] The C(8) carbonyl was identified through a three-bond correlation to C(1)H. The amine proton correlated to the C(5) carbonyl, suggesting the amine has attached to the C(6) site.

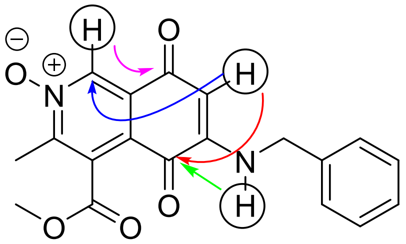

25

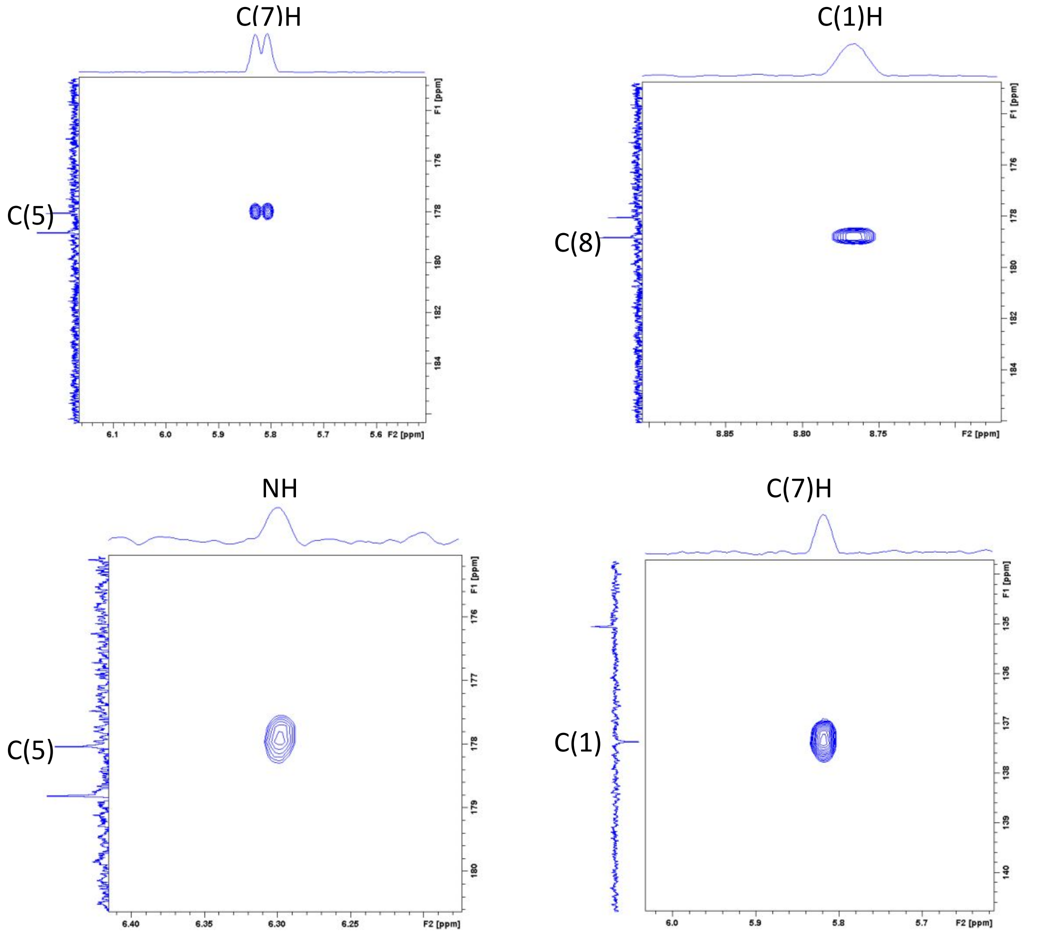

Figure S2 *^{1}H-^{13}C correlations identified through HMBC analysis to characterise* ***25****.*

S1.4 HPLC purity of 25

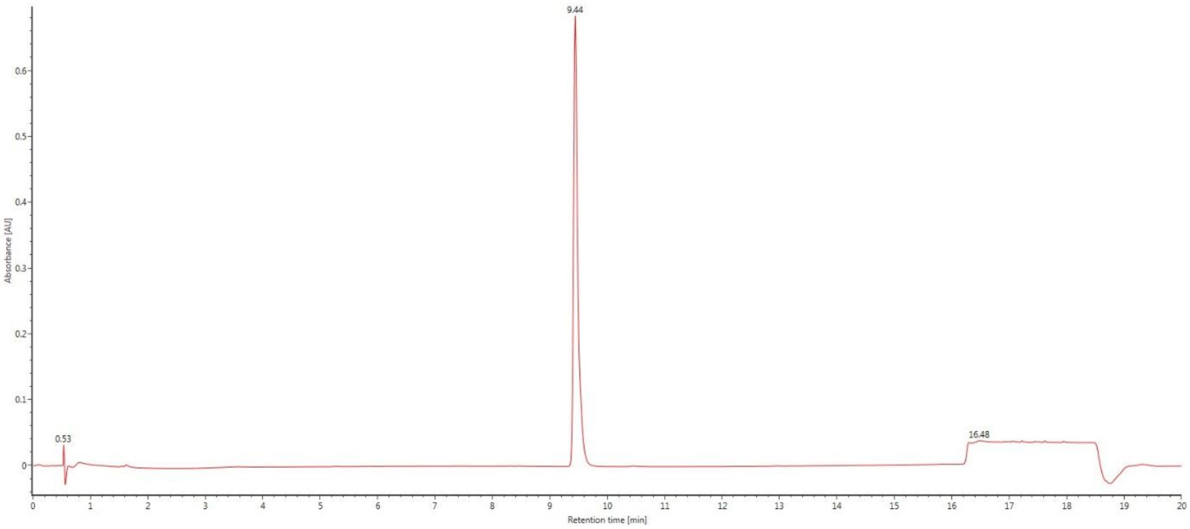

Figure S3 *HPLC chromatogram to confirm purity of* ***25****.*

Table S1 *Peak list of HPLC chromatogram for compound* ***25***

Peak	Retention time [mins]	Peak area [%]
1	9.44	98.0

Instrument: Waters Acquity I-Class

Chromatographic column: Waters Acquity peptide BEH C18 Column, 130Å, 1.7 µm, 2.1 mm X 100 mm

Mobile phase: Water (+0.1% formic acid): Acetonitrile (+0.1% formic acid)

Gradient: 0 mins 90:10; 1 mins 90:10; 16mins 10:90: 16.1 mins 0 :100 ; 18.5 mins 90 :10

Detection wavelength: 254 nm

Detection temperature: 20 °C

Injection volume: 10 µL

S1.5 ^{1}H and ^{13}C NMR spectra for compounds **3-25**

^{1}H NMR of **Compound 3**

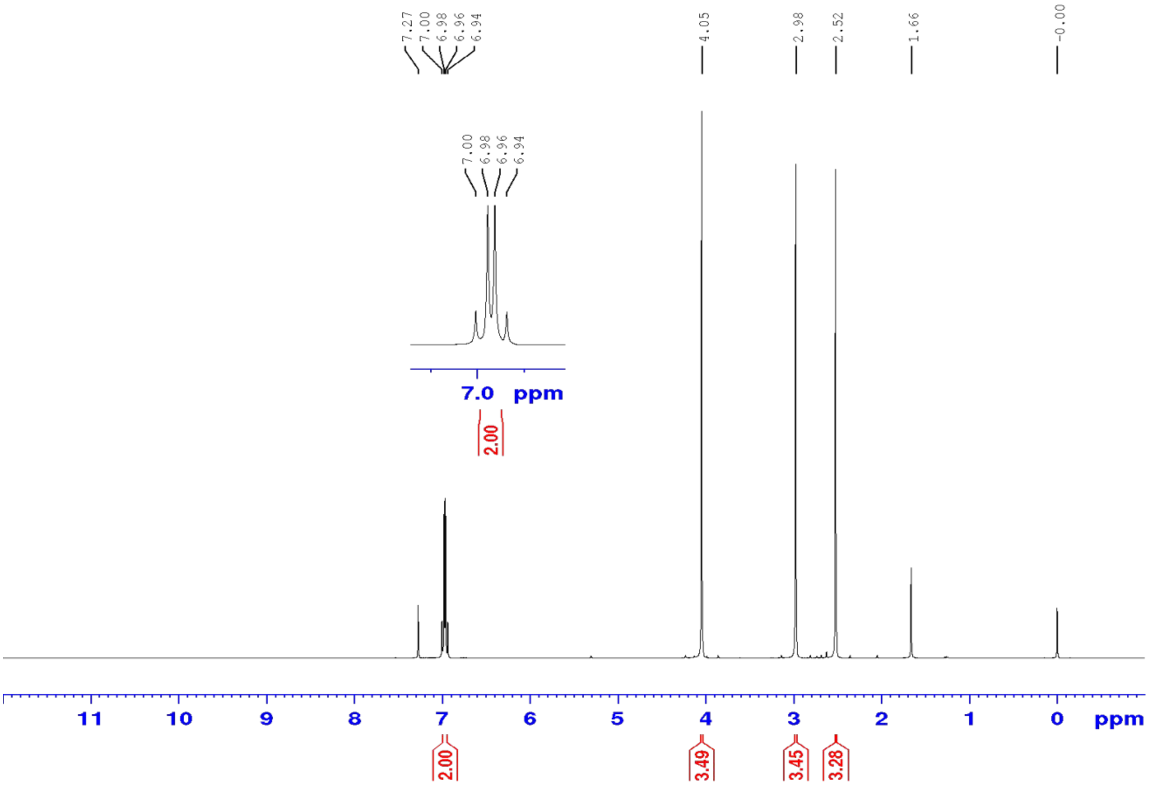

^{13}C NMR of **Compound 3**

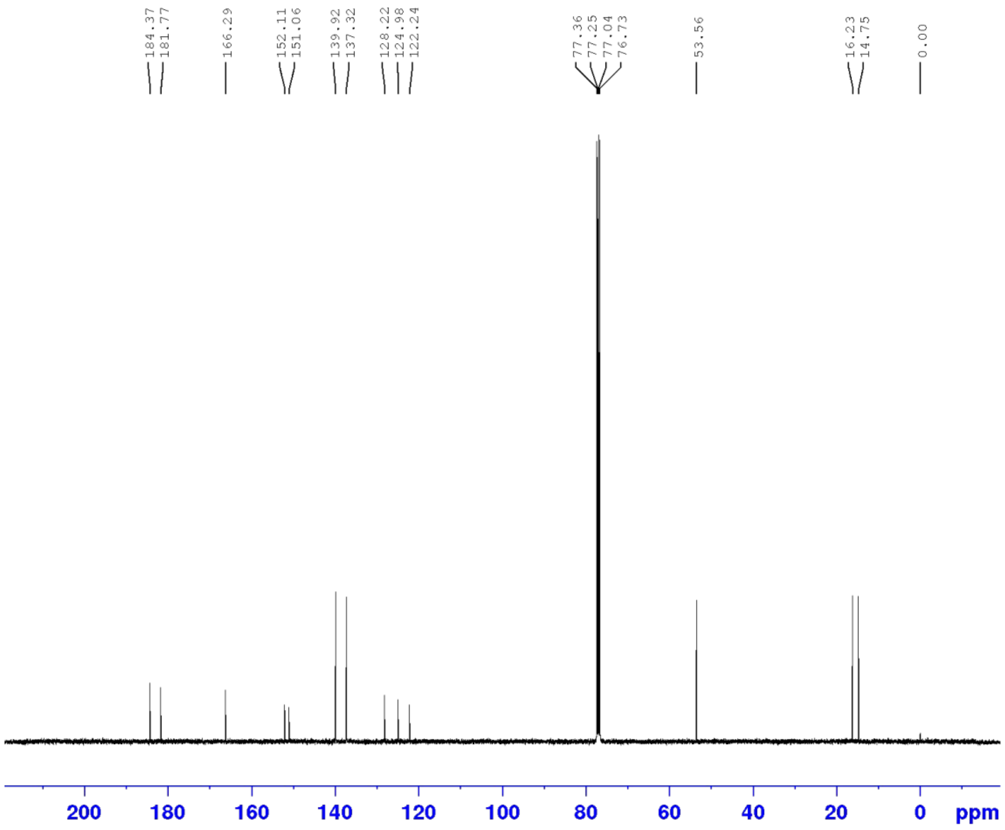

^{1}H NMR of **Compound 4**

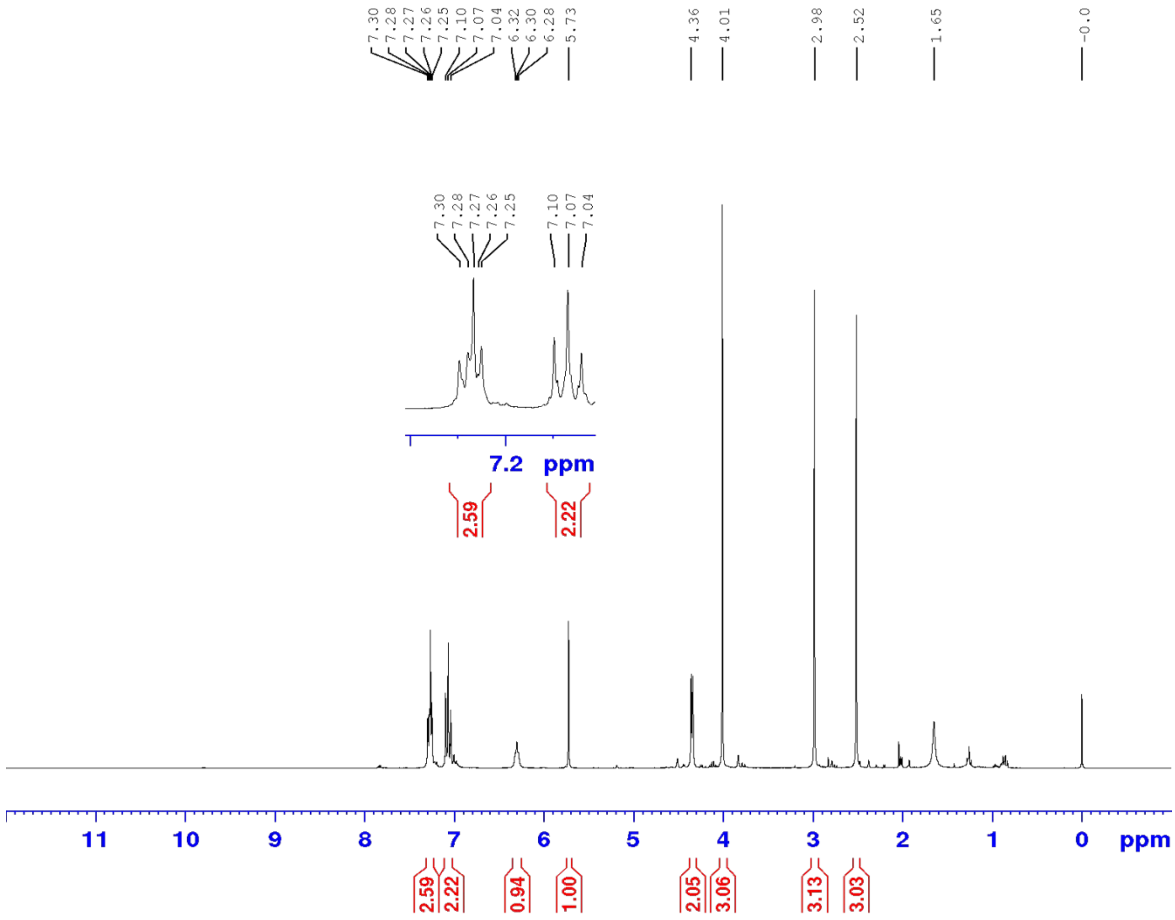

^{13}C NMR of **Compound 4**

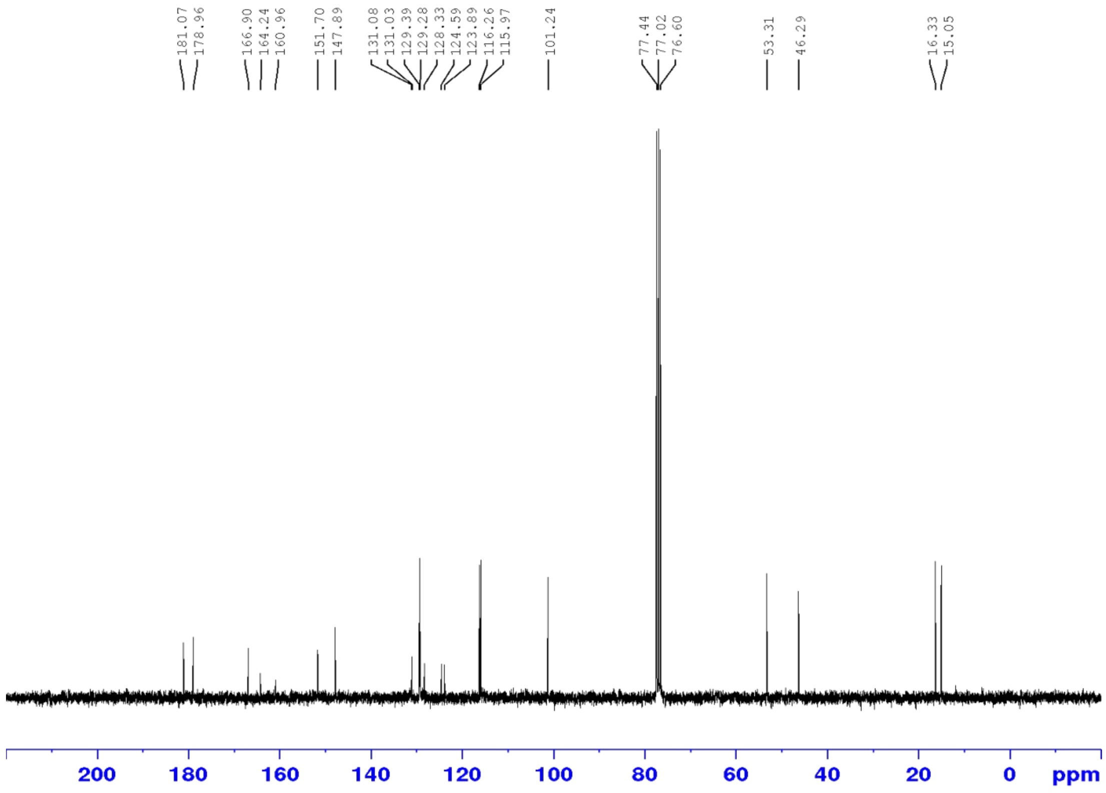

^{1}H NMR of **Compound 5**

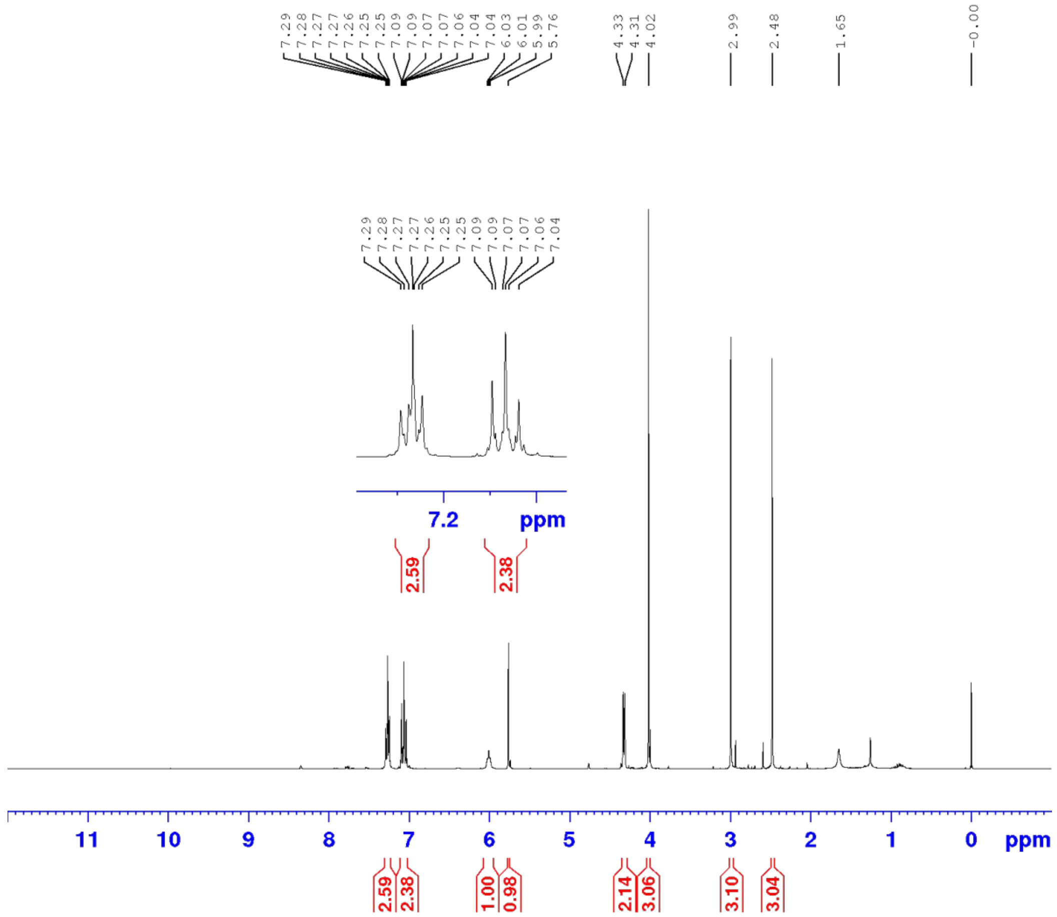

^{13}C NMR of **Compound 5**

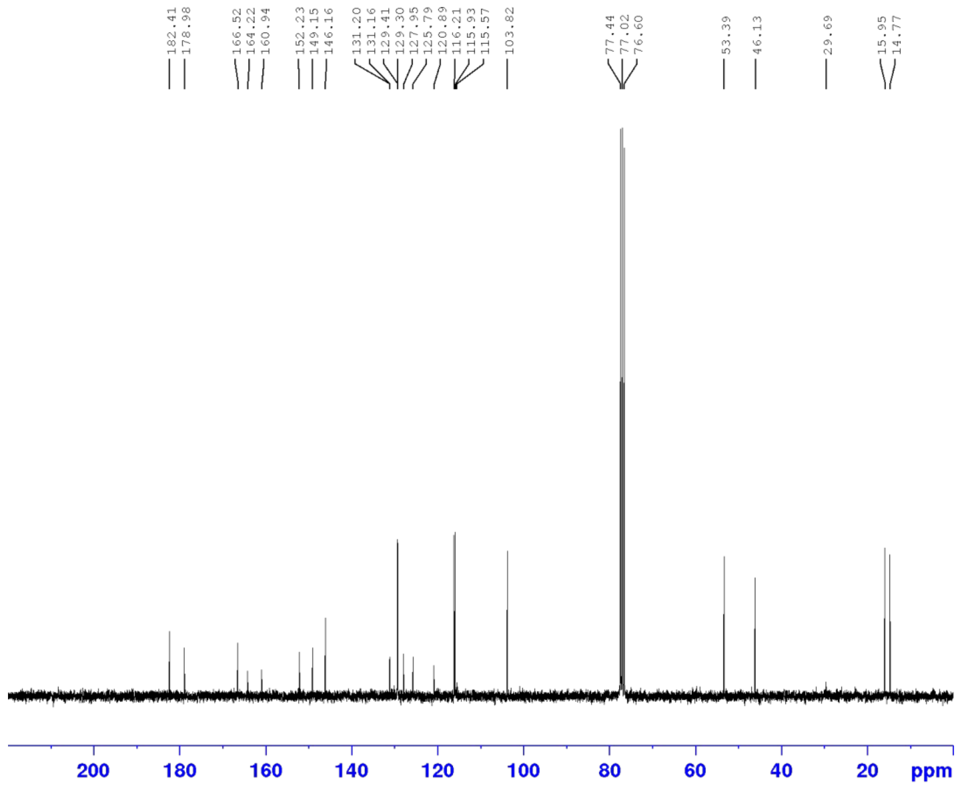

^{1}H NMR of **Compound 6**

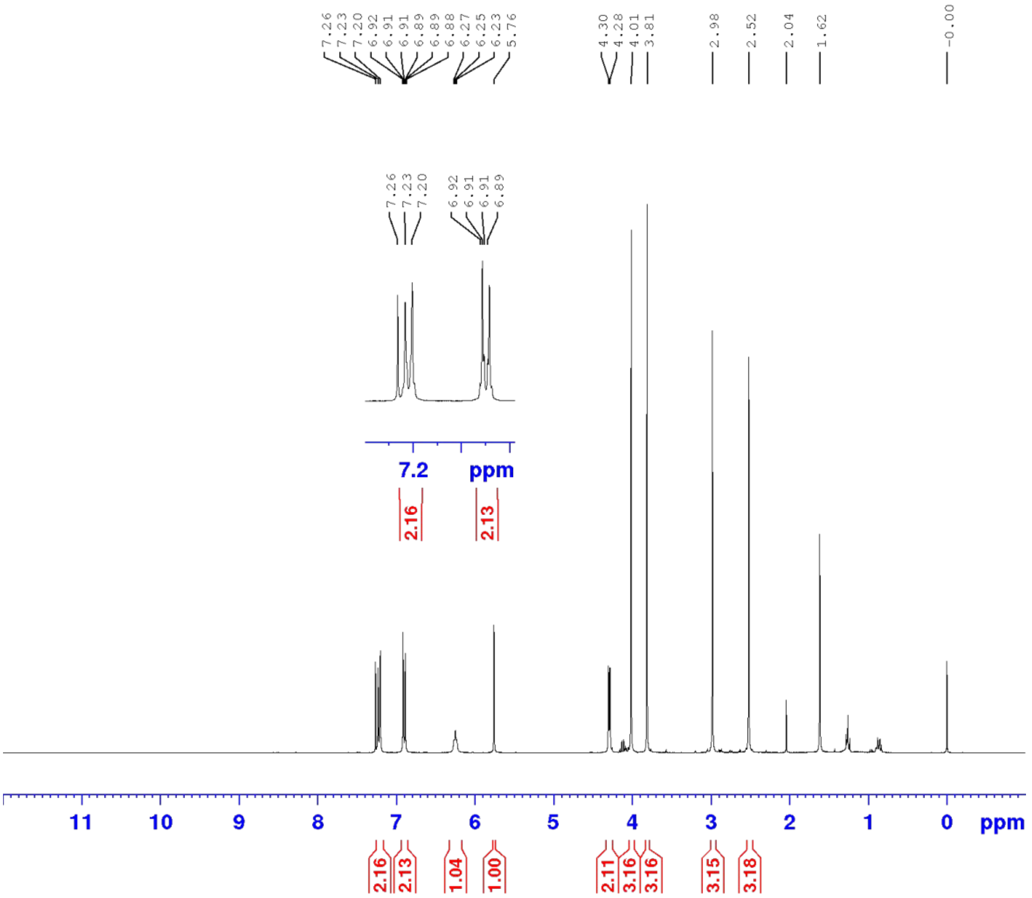

^{13}C NMR of **Compound 6**

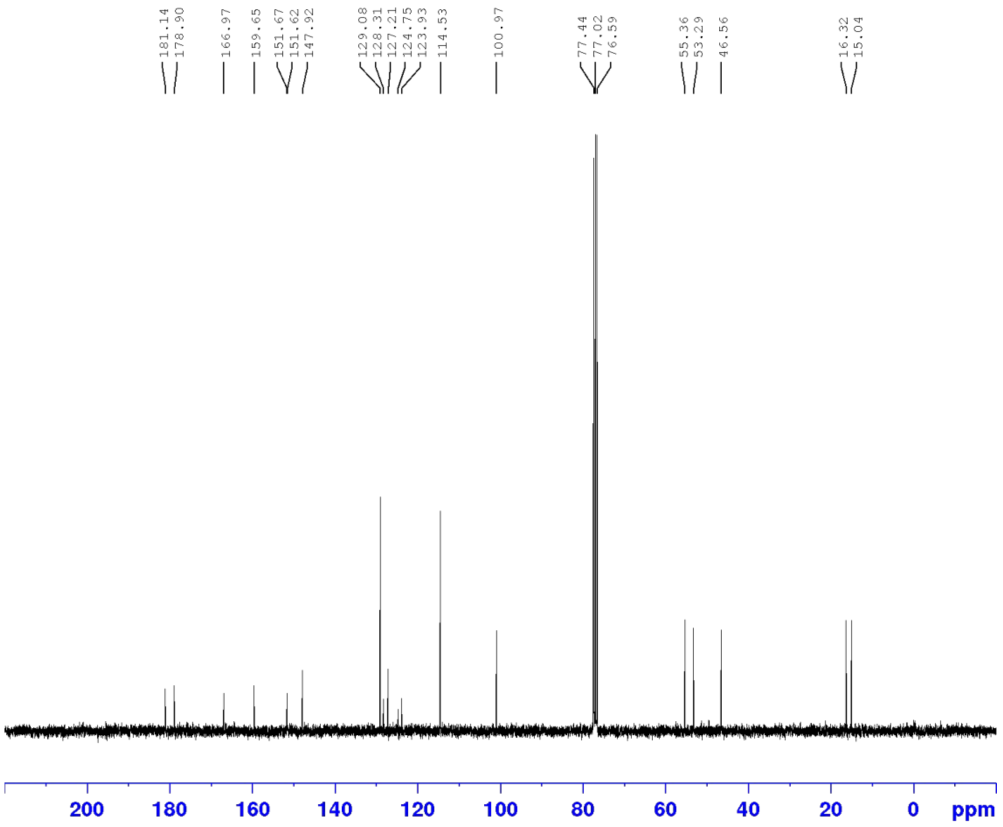

^{1}H NMR of **Compound 7**

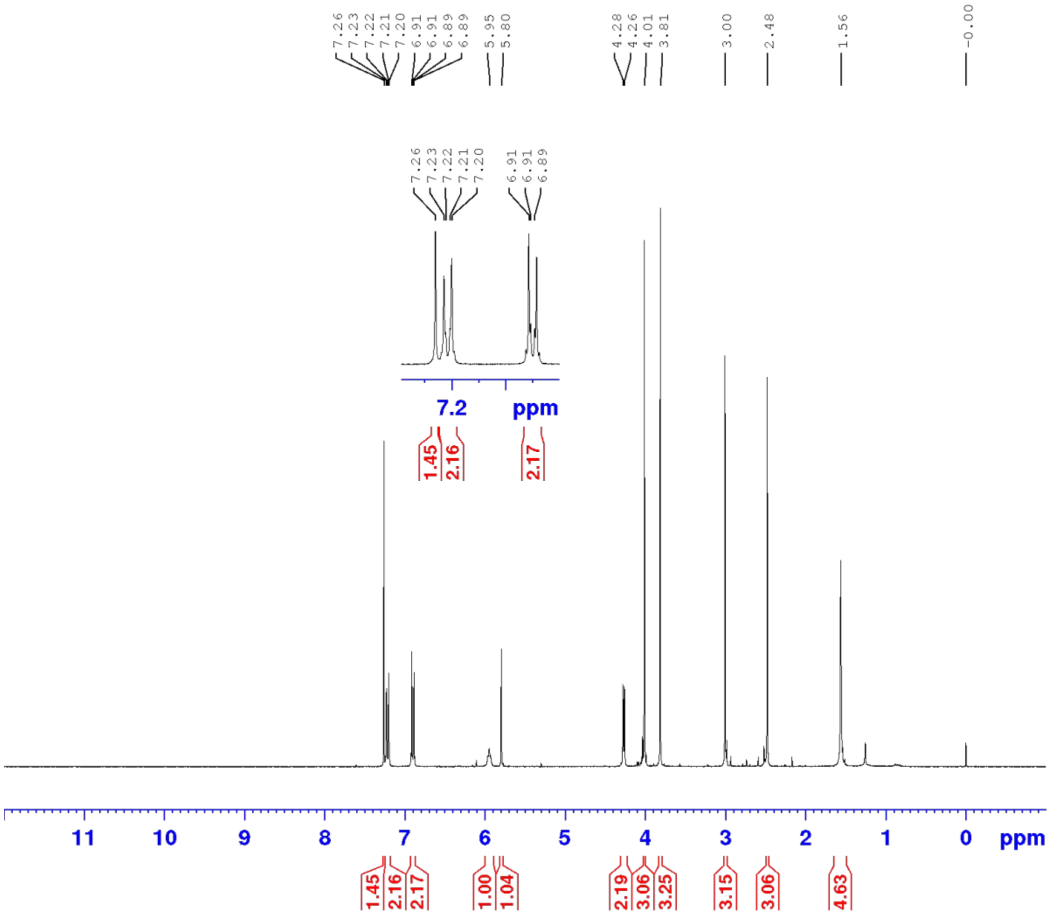

^{13}C NMR of **Compound 7**

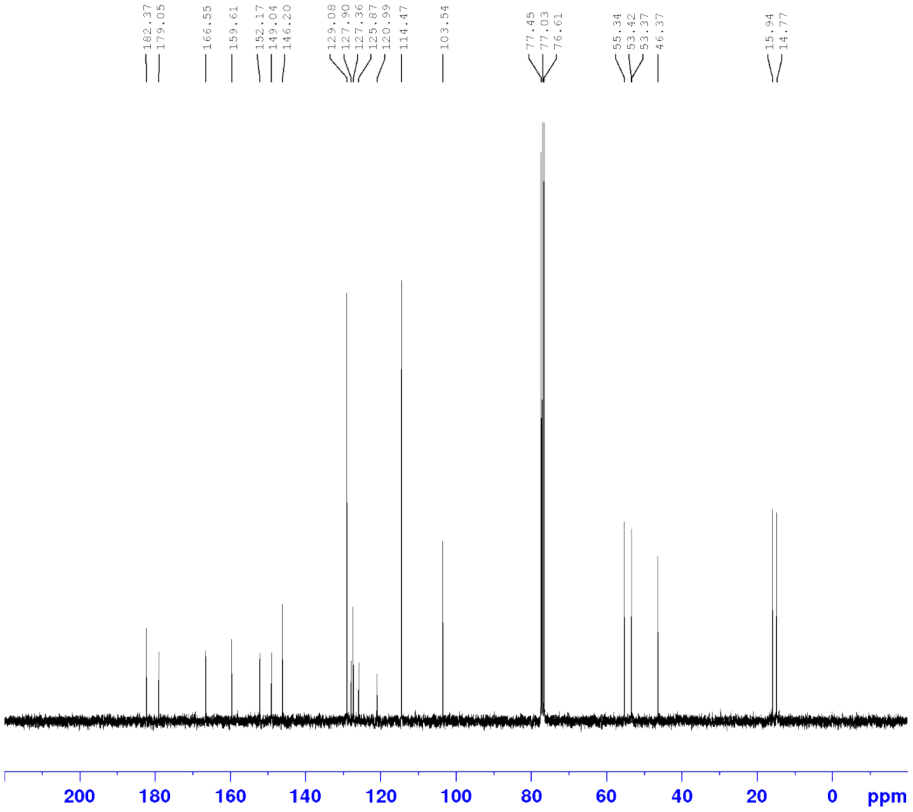

^{1}H NMR of **Compound 8**

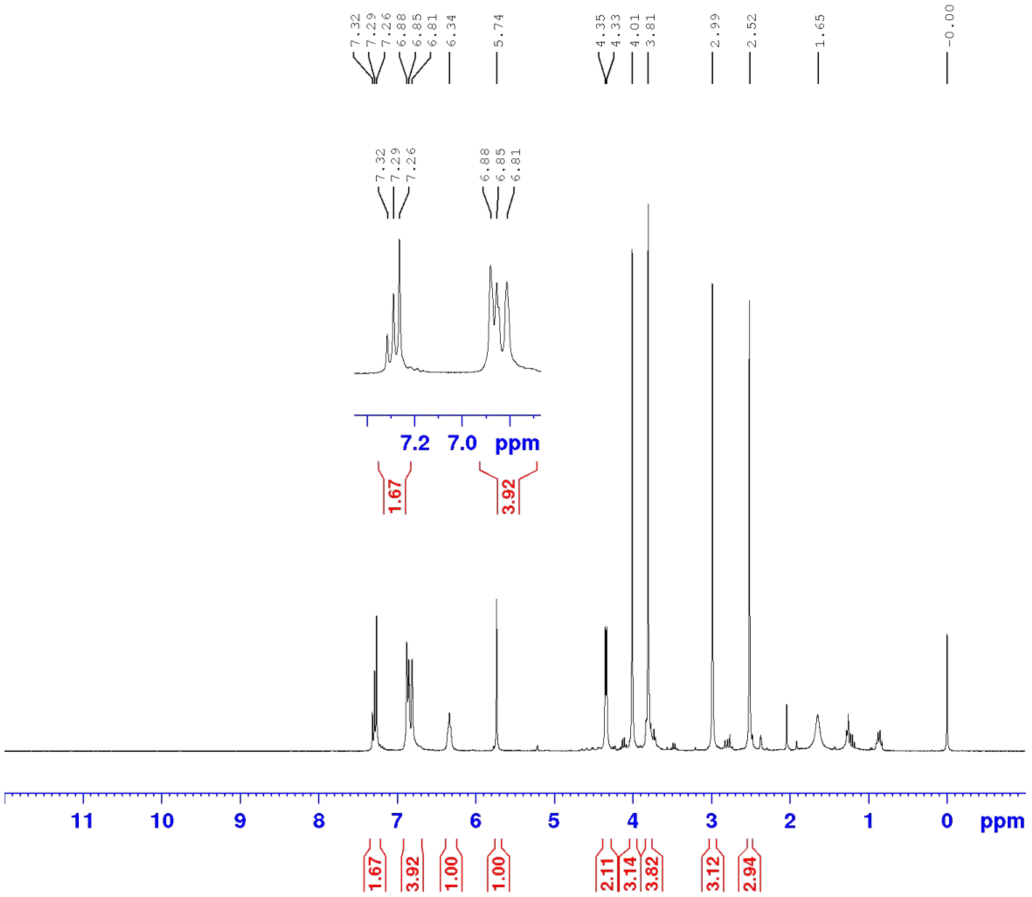

^{13}C NMR of **Compound 8**

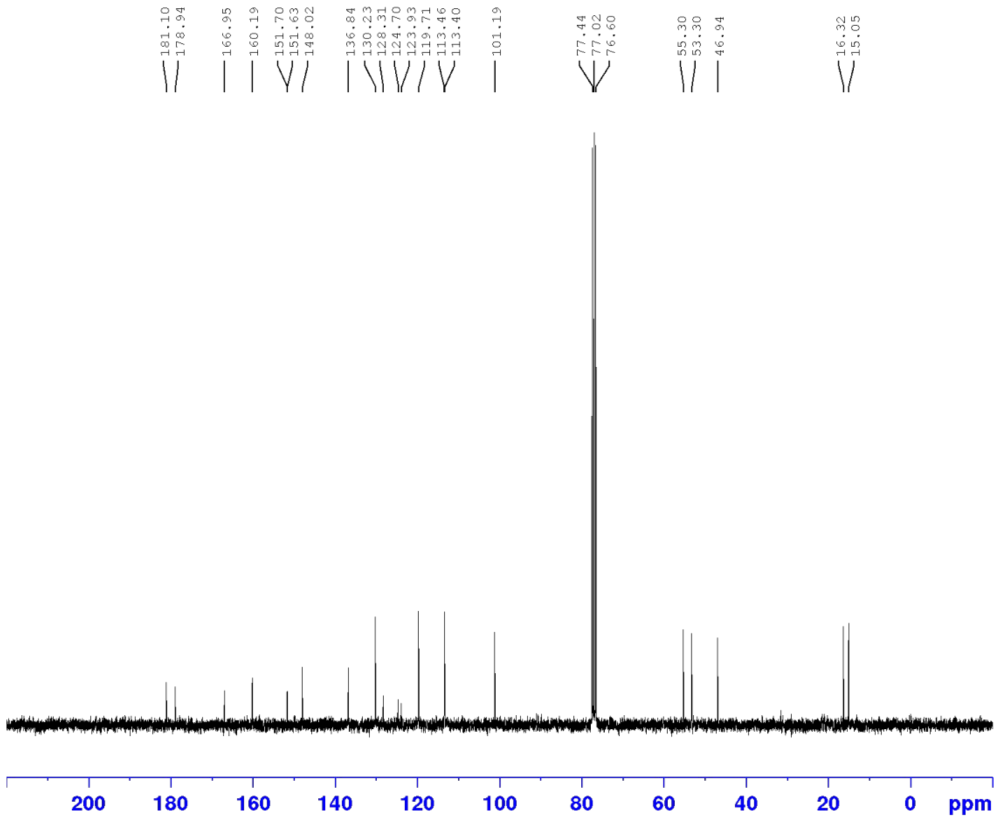

^{1}H NMR of **Compound 9**

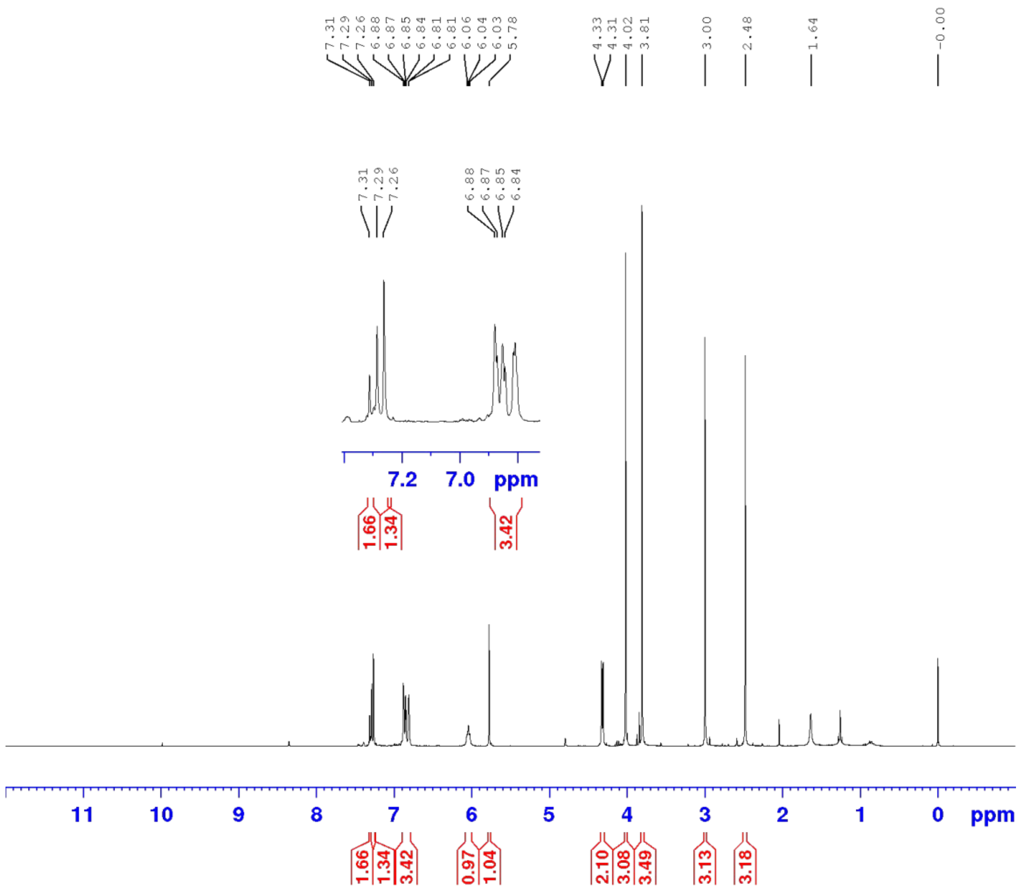

^{13}C NMR of **Compound 9**

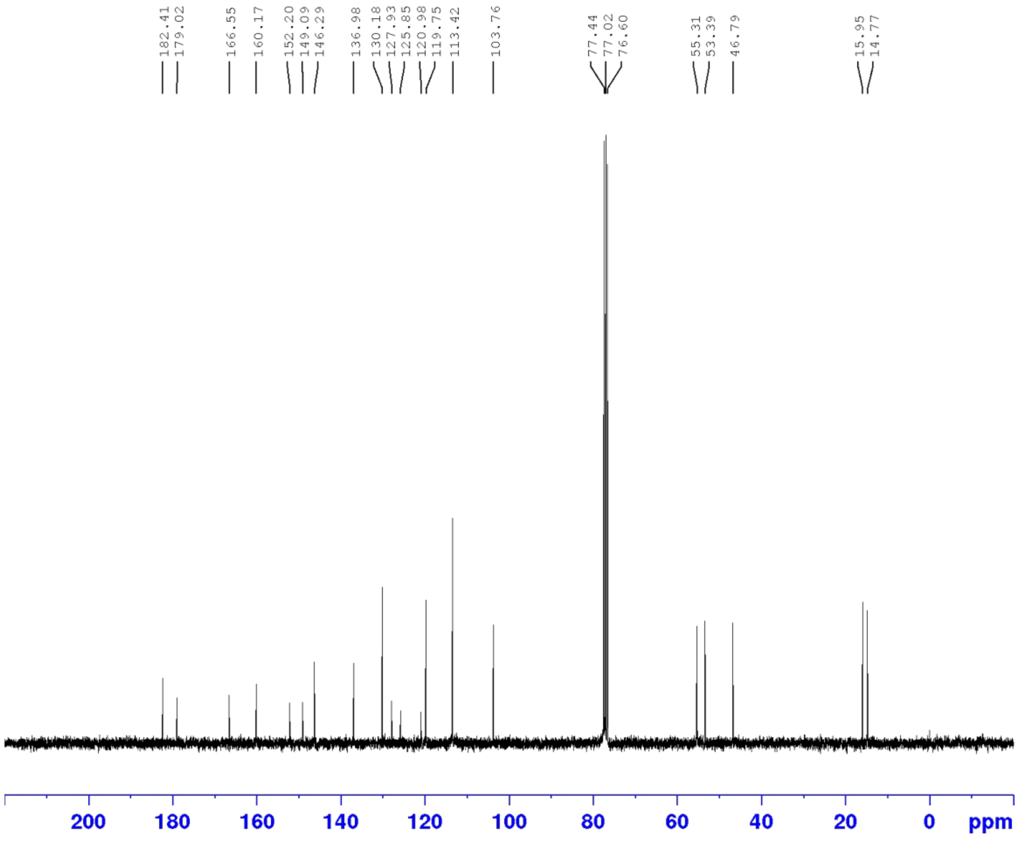

^{1}H NMR of **Compound 10**

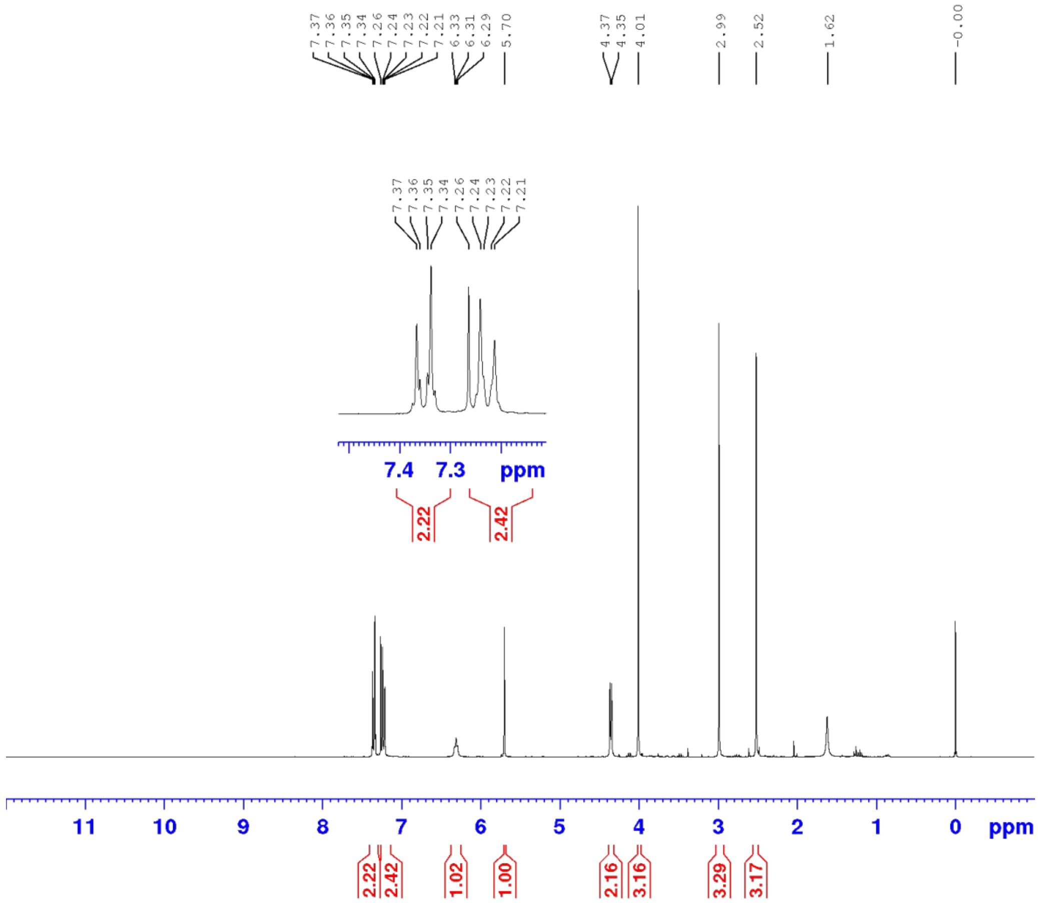

^{13}C NMR of **Compound 10**

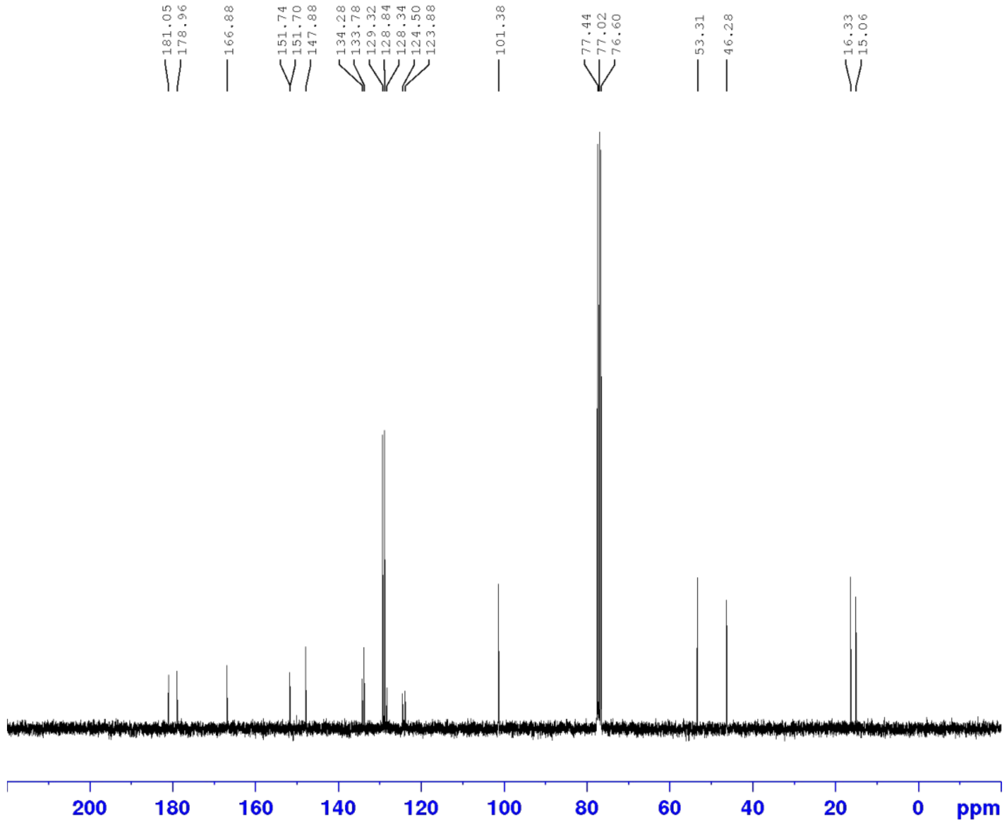

^{1}H NMR of **Compound 11**

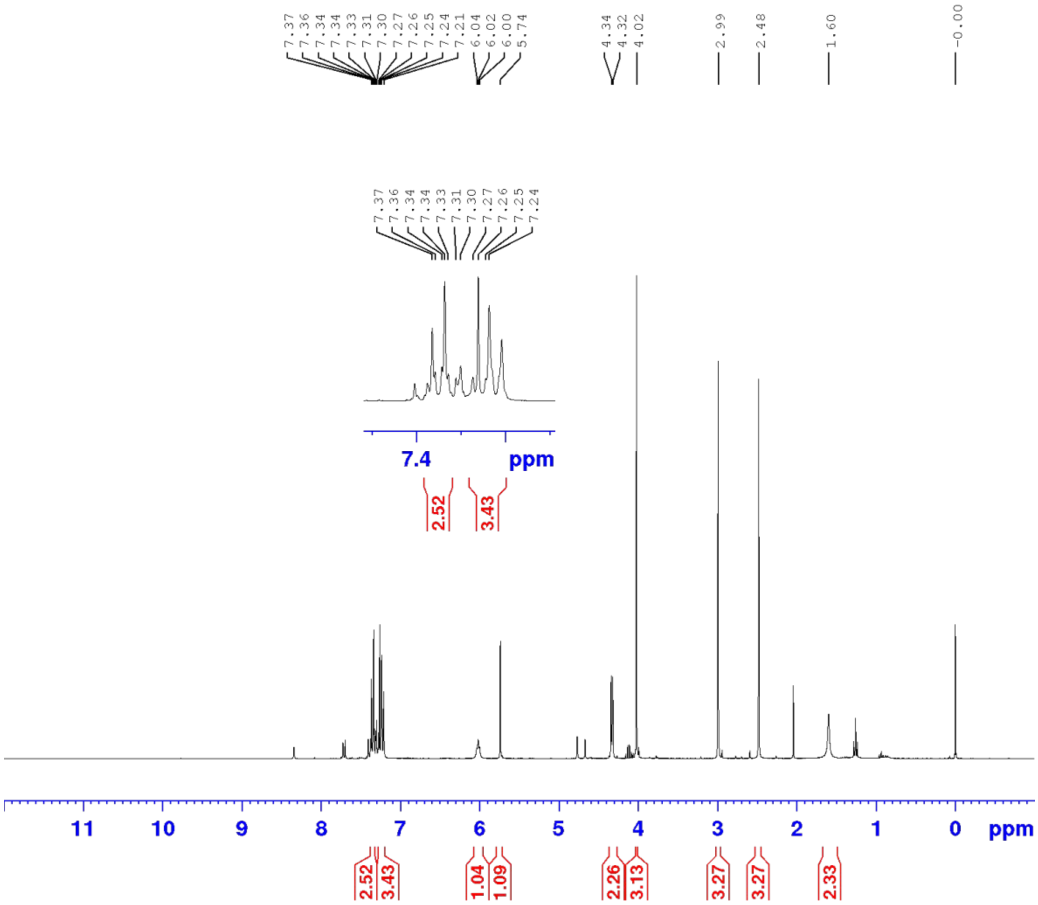

^{13}C NMR of **Compound 11**

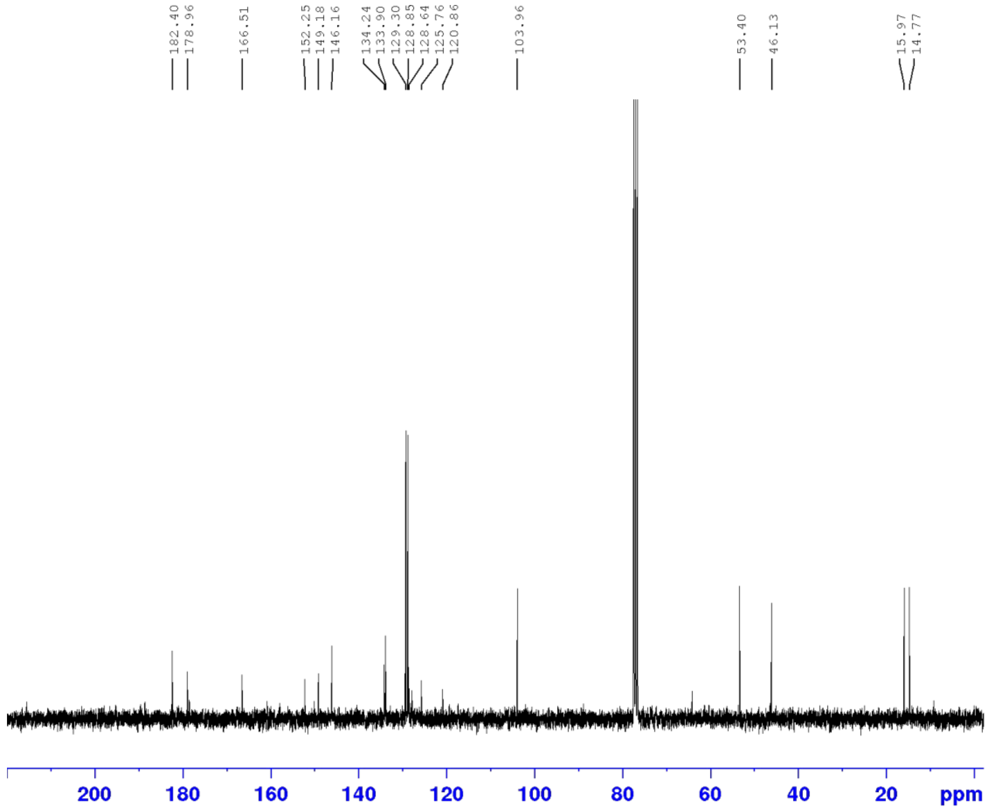

^{1}H NMR of **Compound 12**

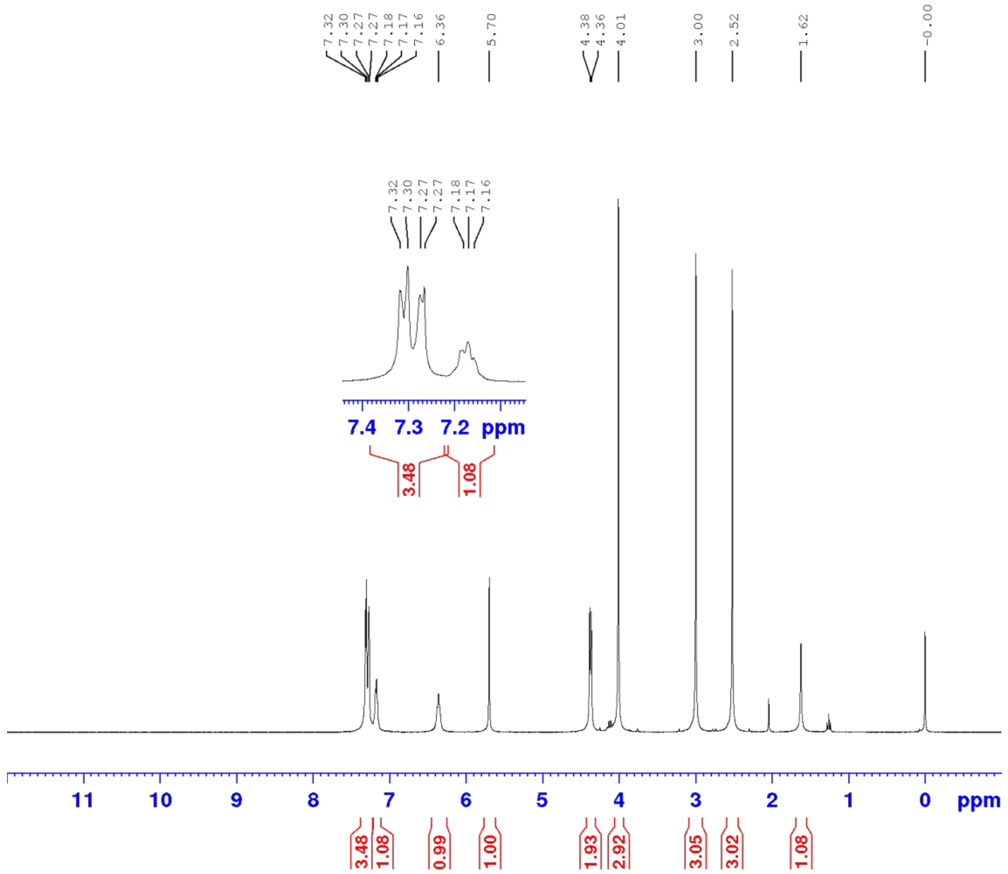

^{13}C NMR of **Compound 12**

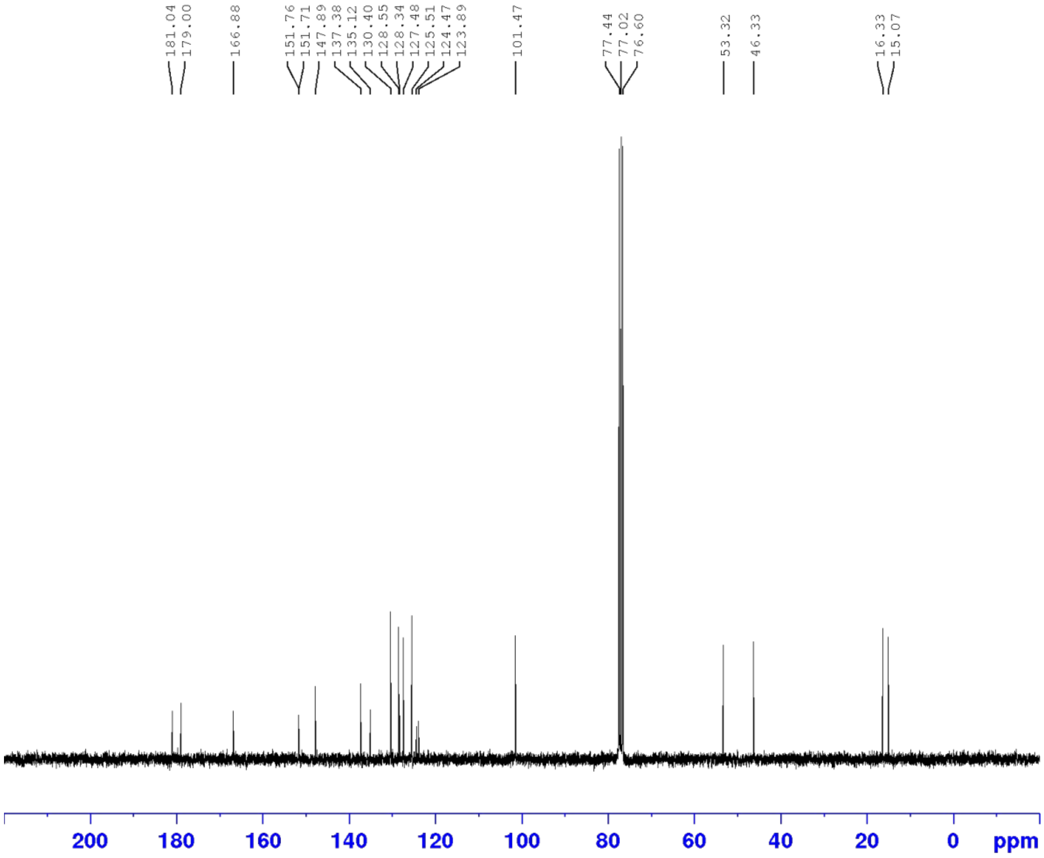

^{1}H NMR of **Compound 13**

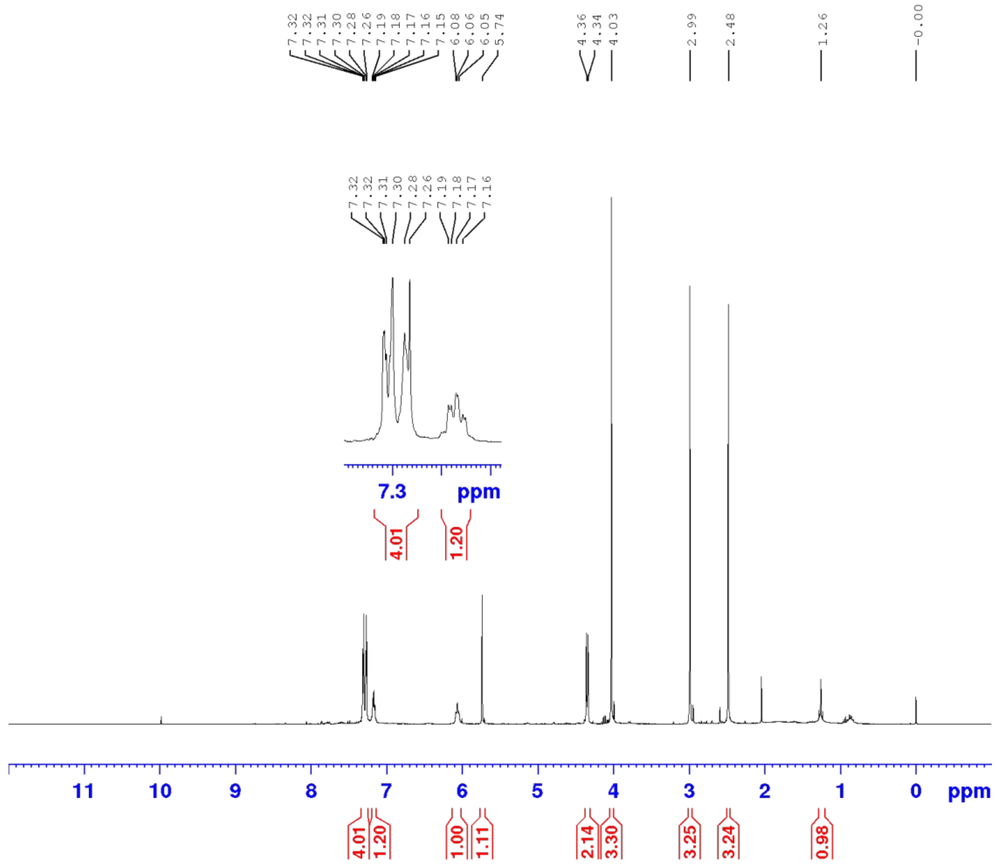

^{13}C NMR of **Compound 13**

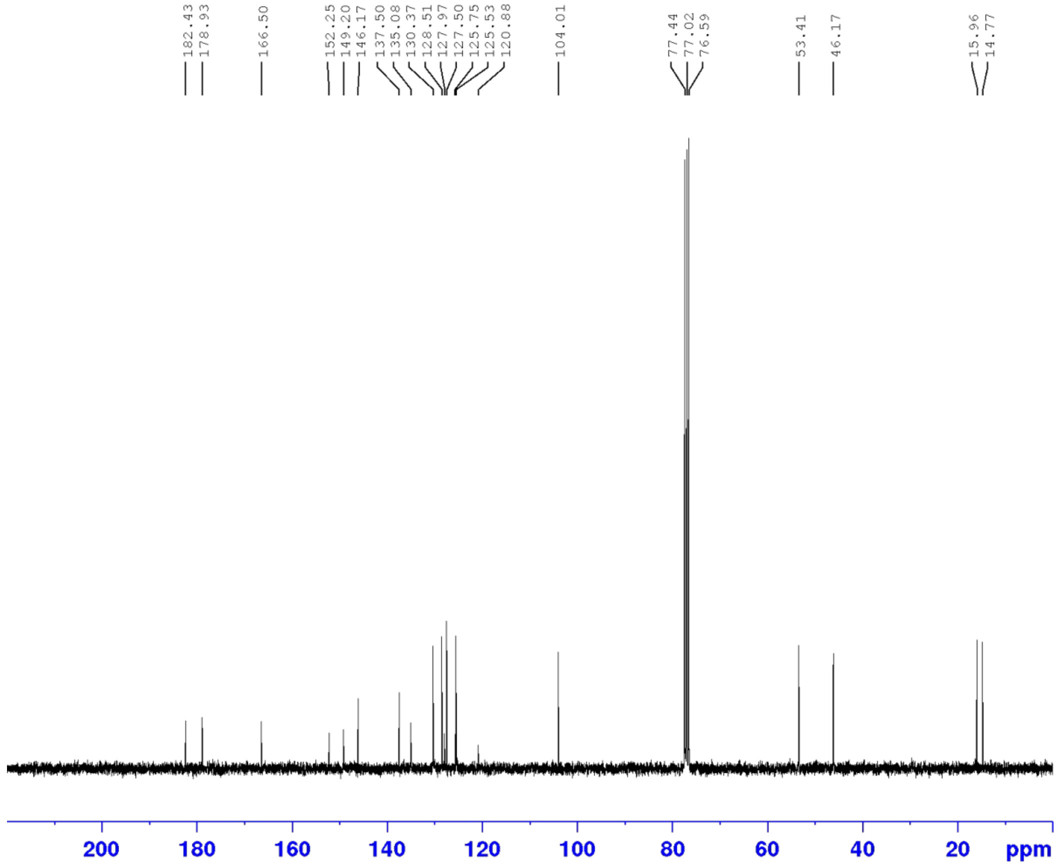

^{1}H NMR of **Compound 14**

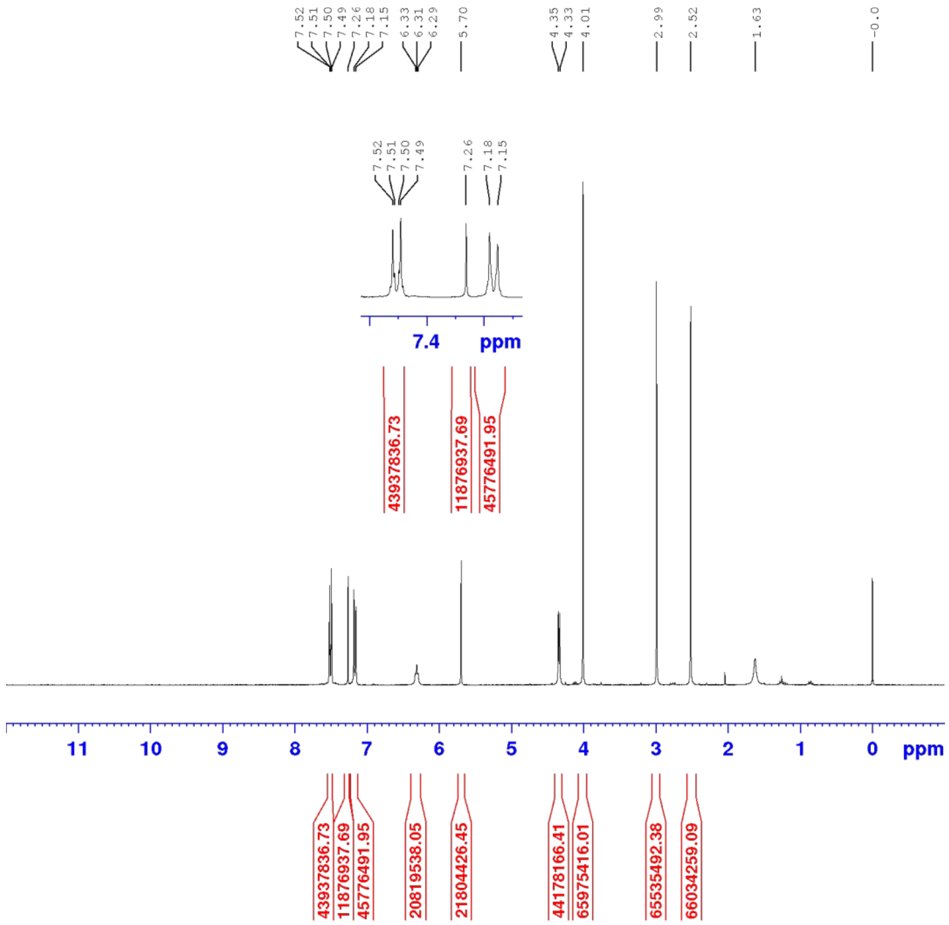

^{13}C NMR of **Compound 14**

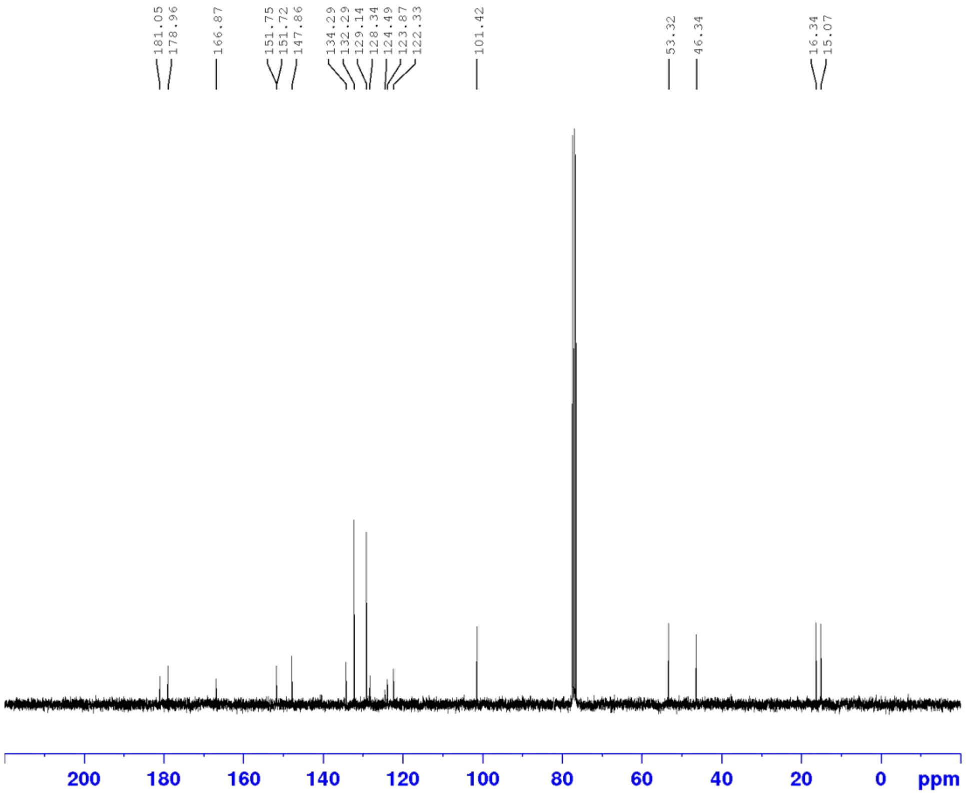

^{1}H NMR of **Compound 15**

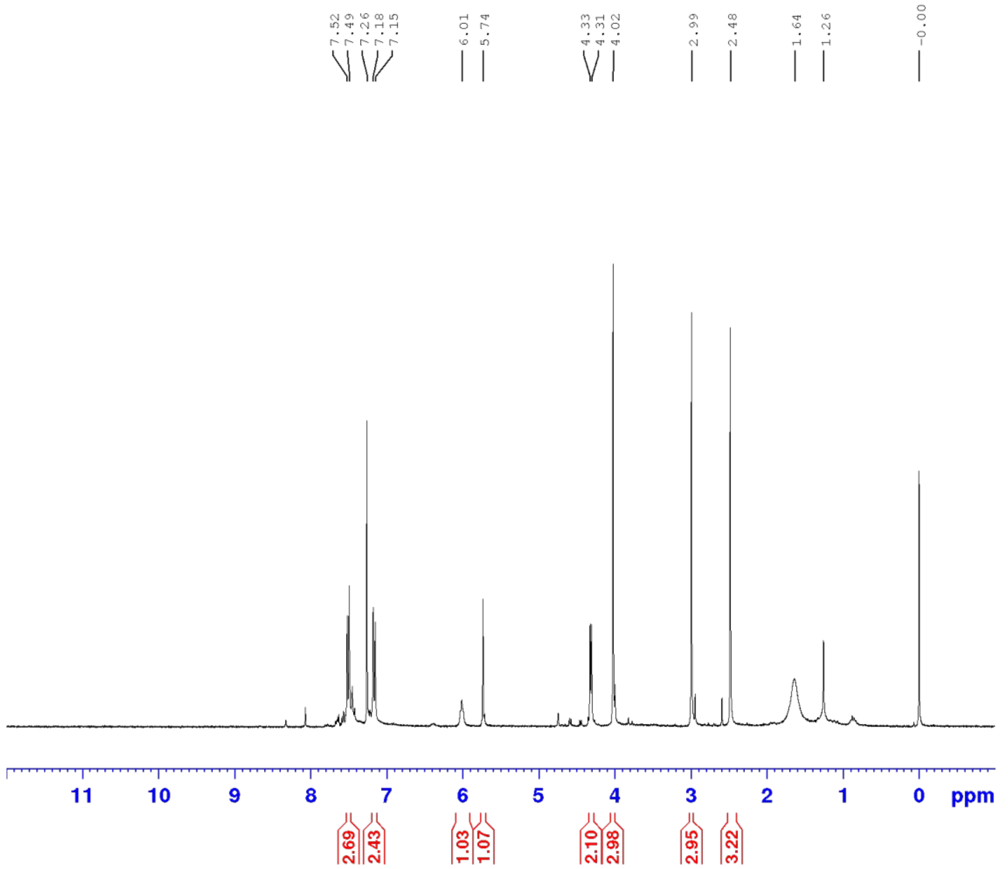

^{13}C NMR of **Compound 15**

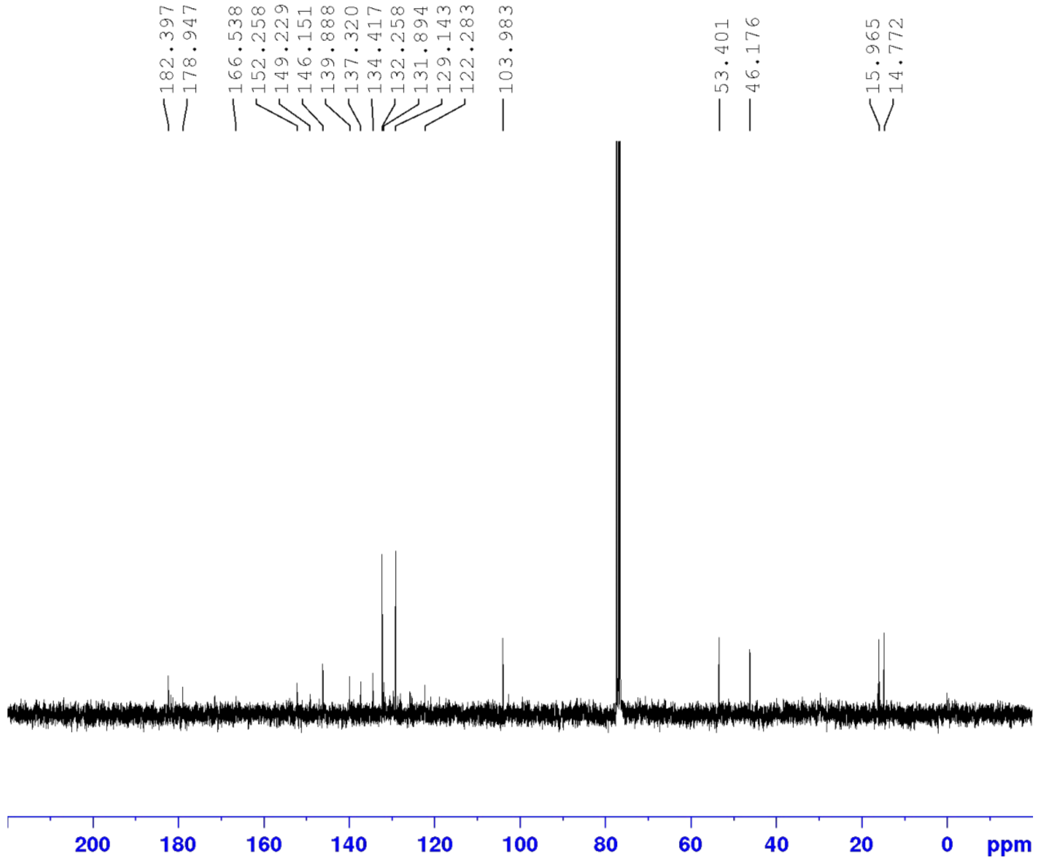

^{1}H NMR of **Compound 16**

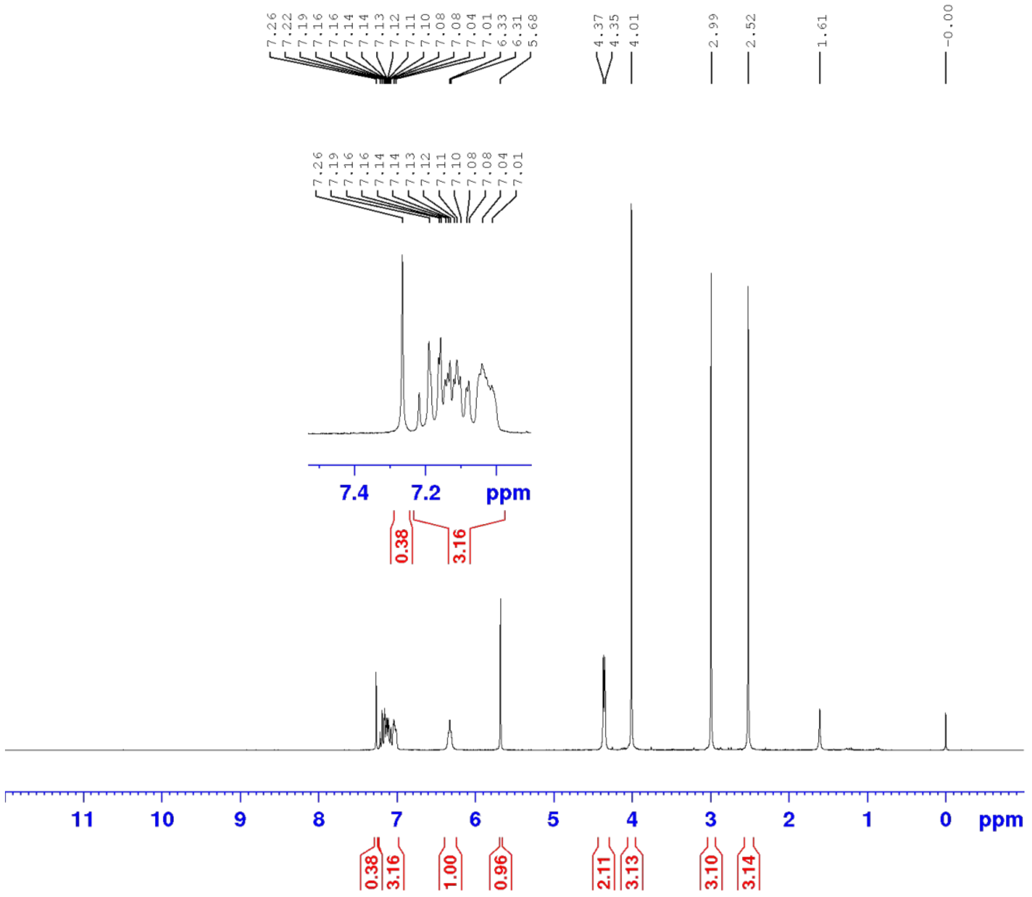

^{13}C NMR of **Compound 16**

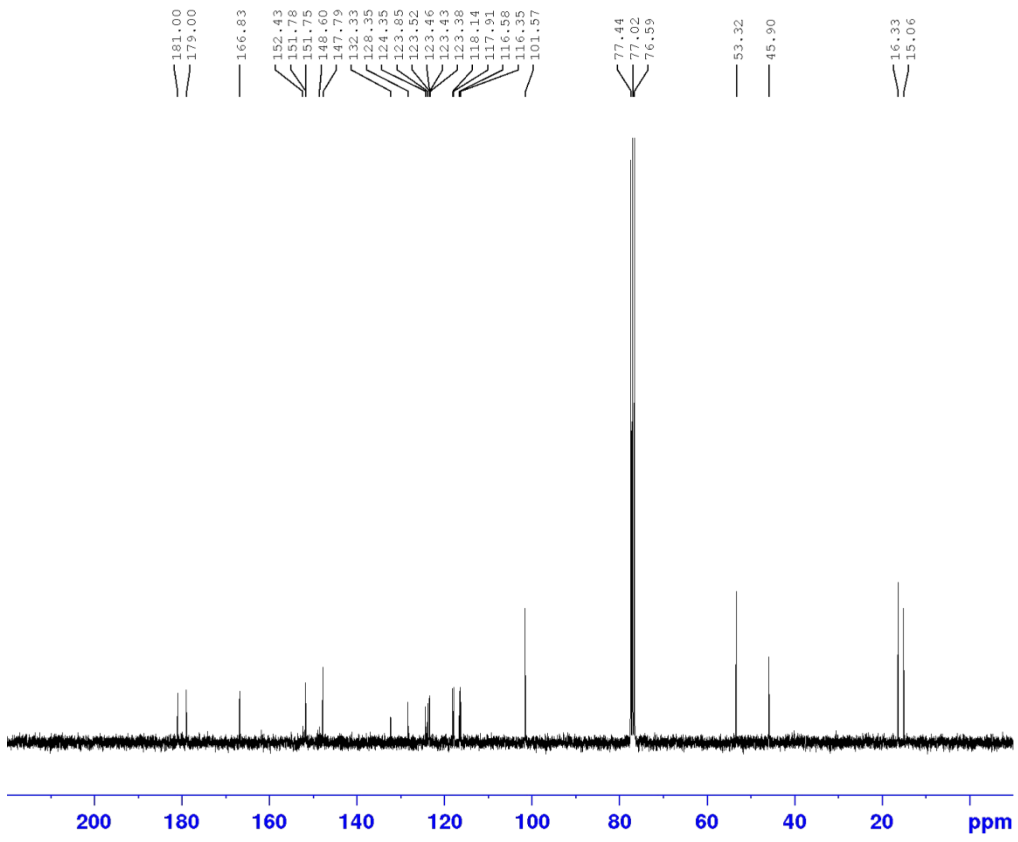

^{1}H NMR of **Compound 17**

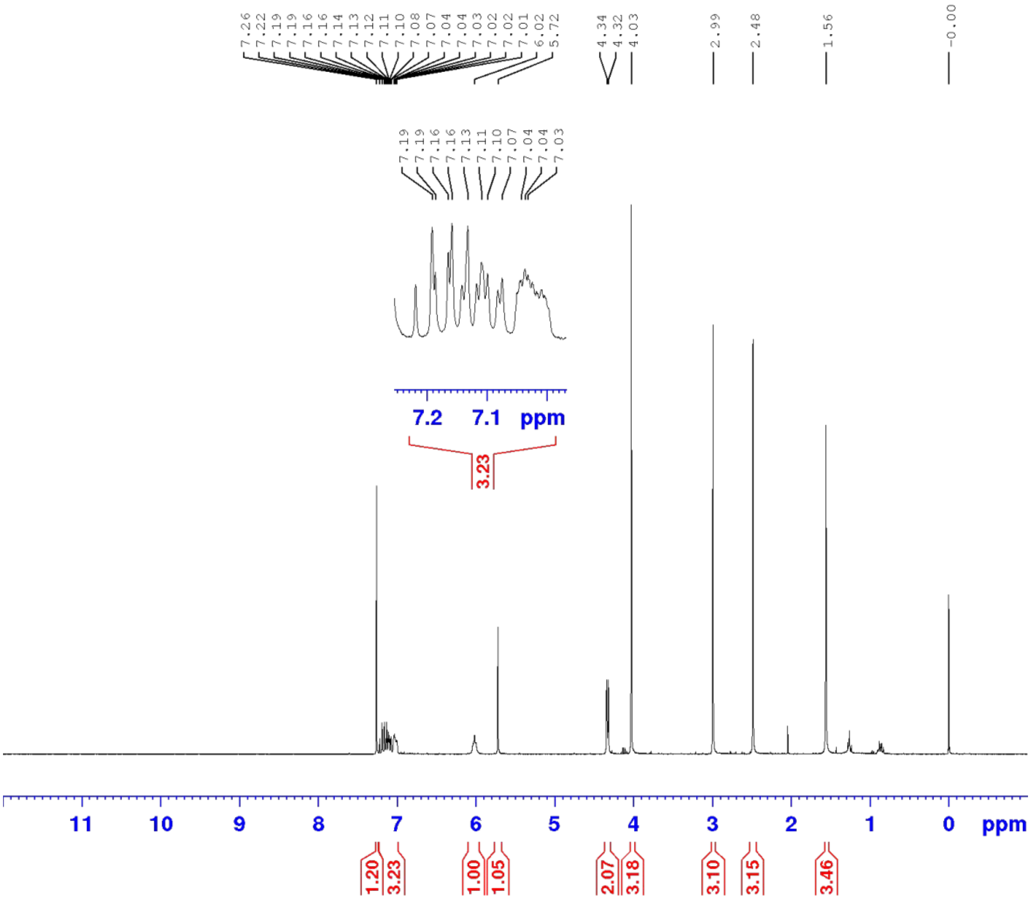

^{13}C NMR of **Compound 17**

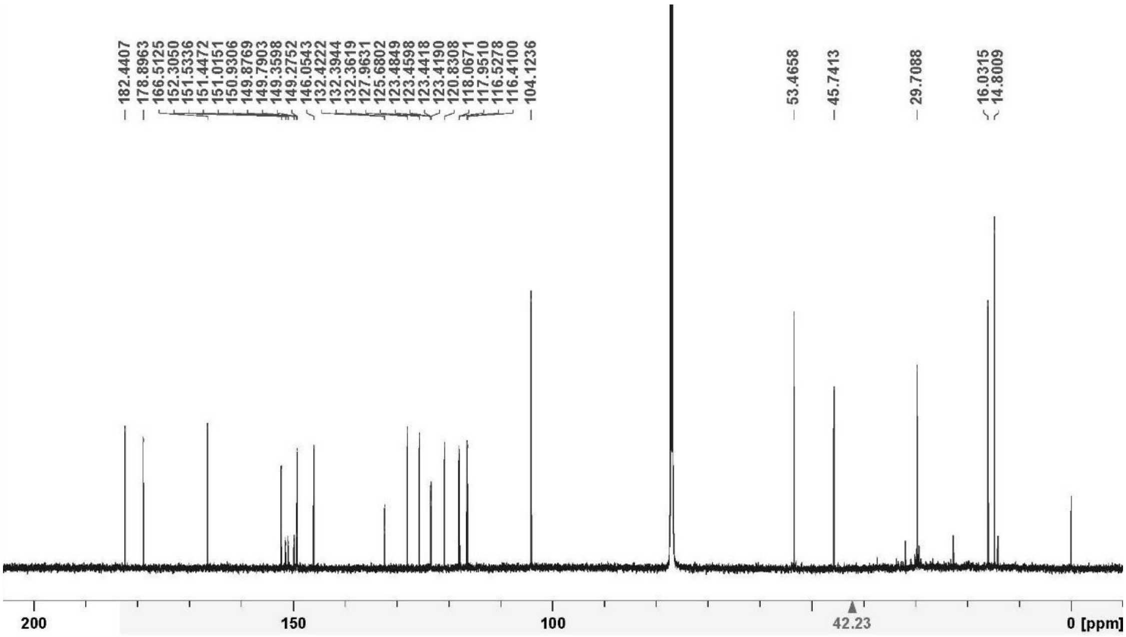

^{1}H NMR of **Compound 18**

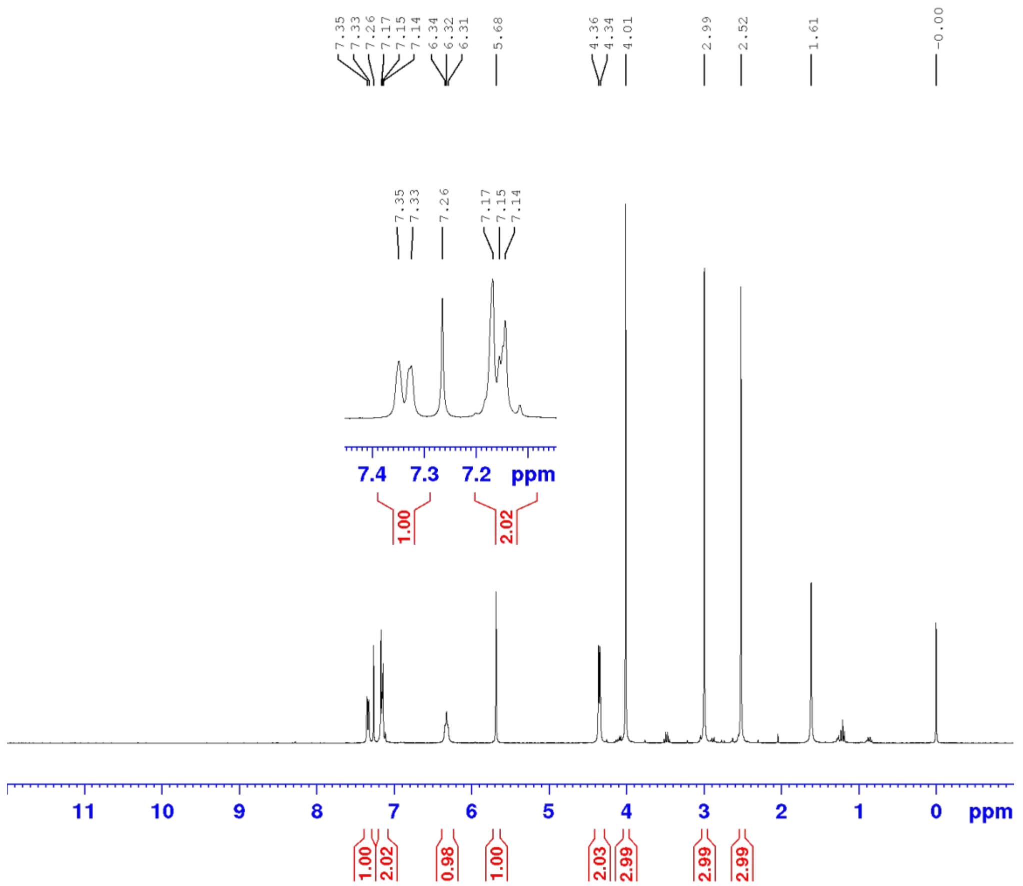

^{13}C NMR of **Compound 18**

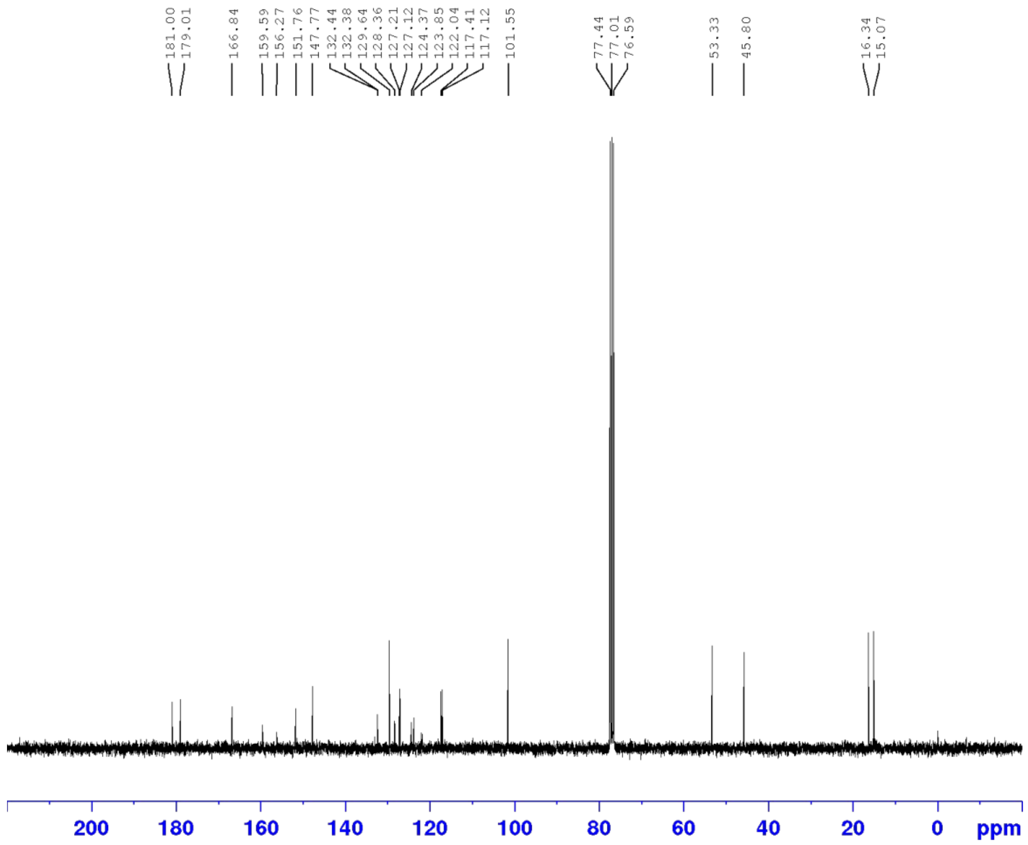

^{1}H NMR of **Compound 19**

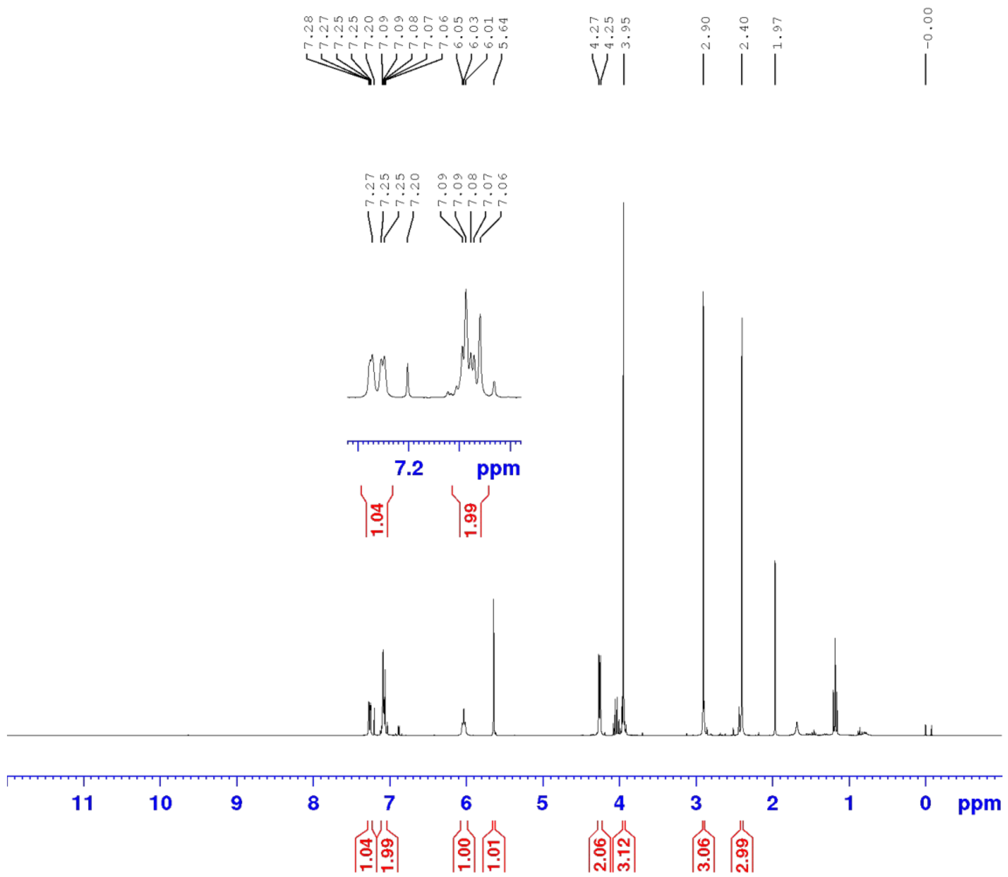

^{13}C NMR of **Compound 19**

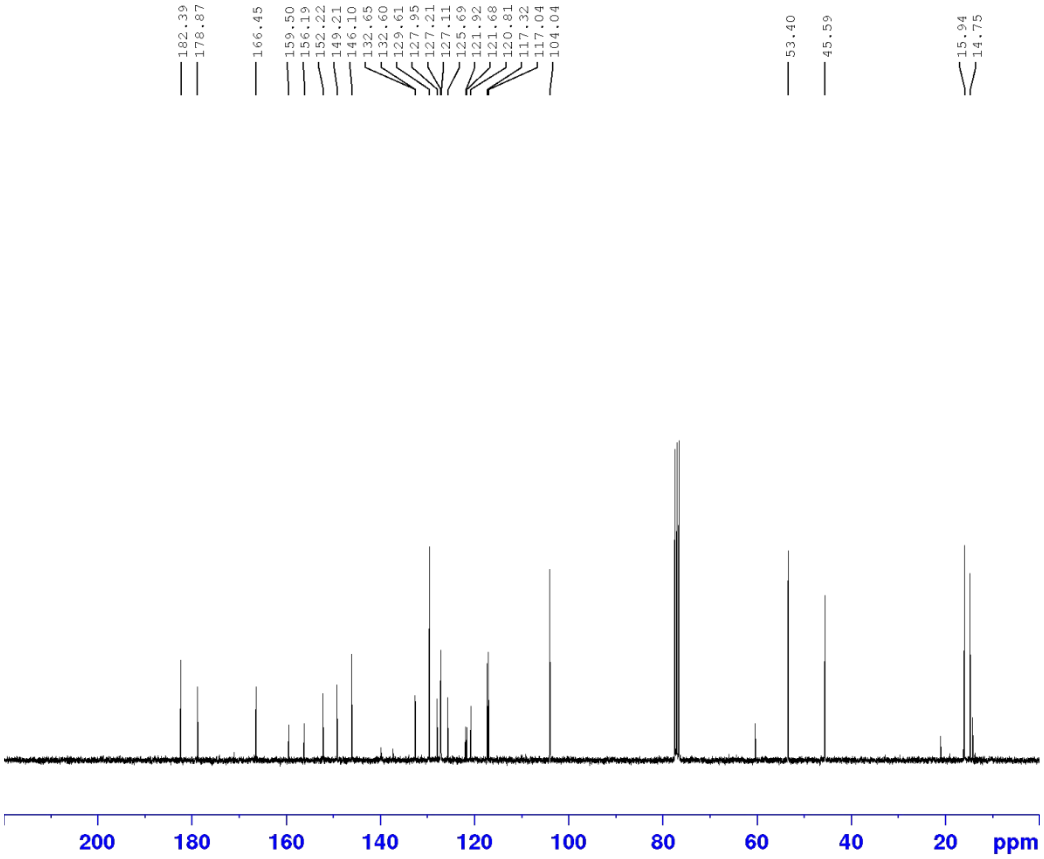

^{1}H NMR of **Compound 20**

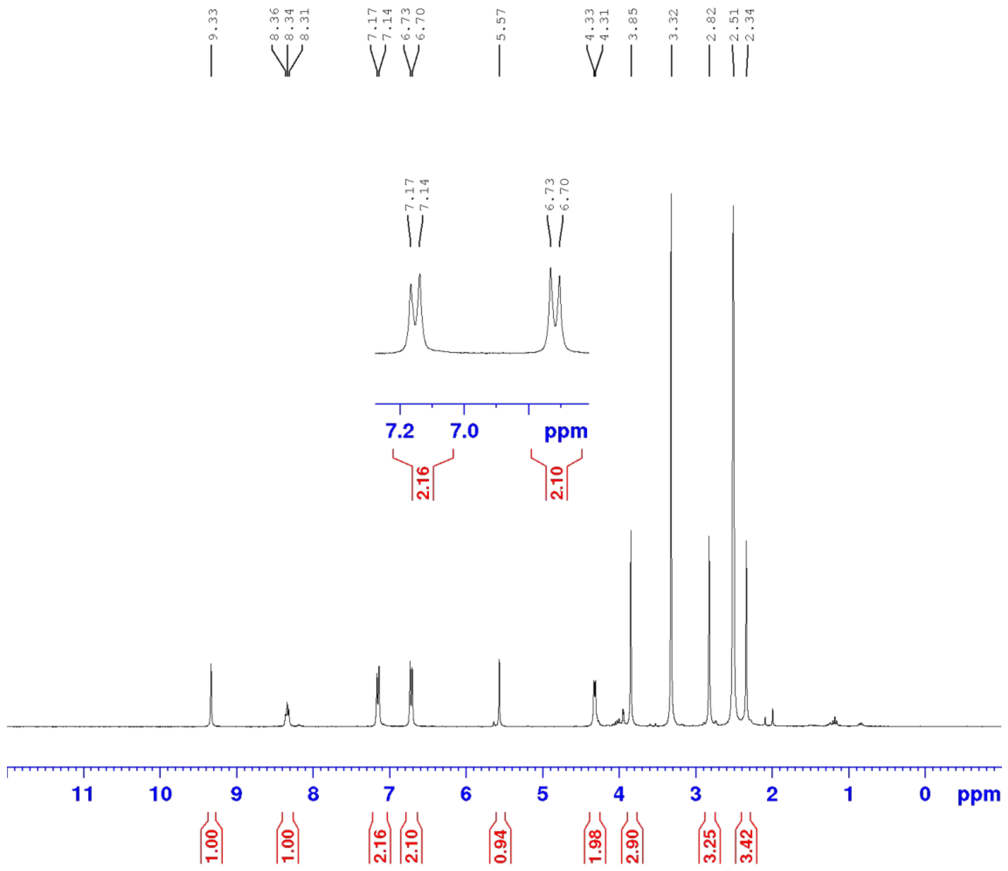

^{13}C NMR of **Compound 20**

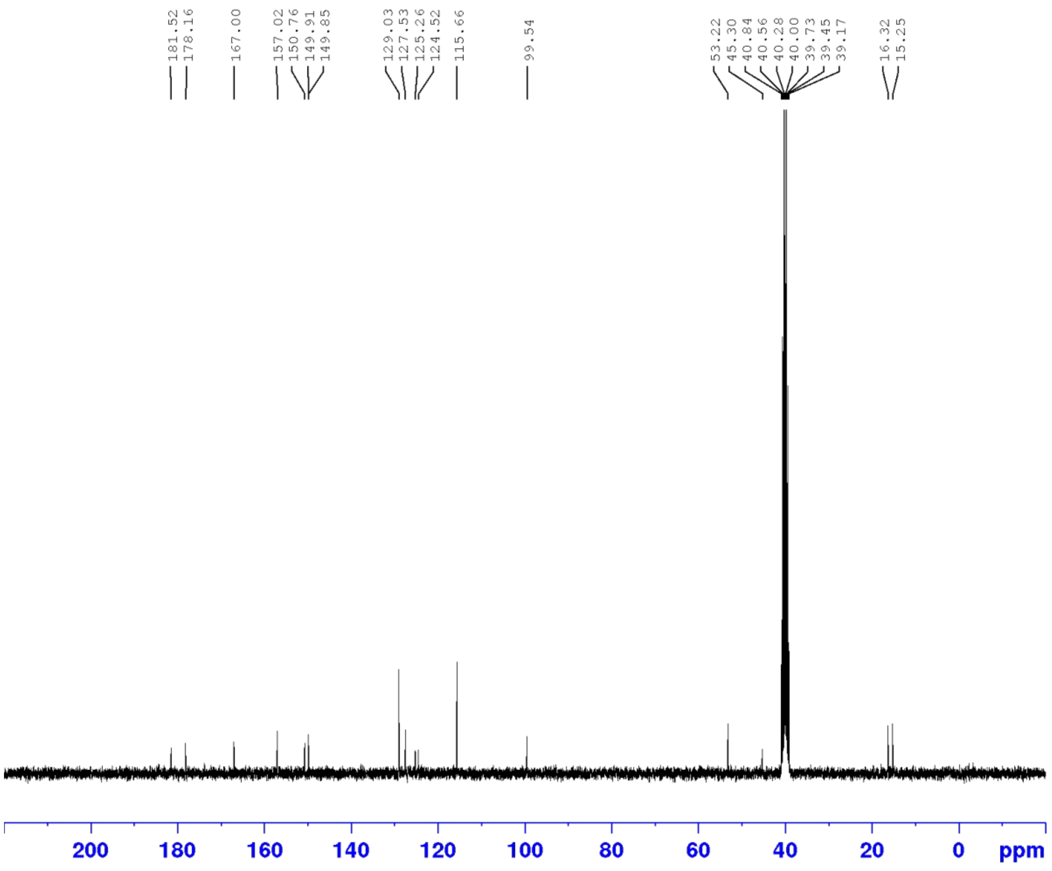

^{1}H NMR of **Compound 22**

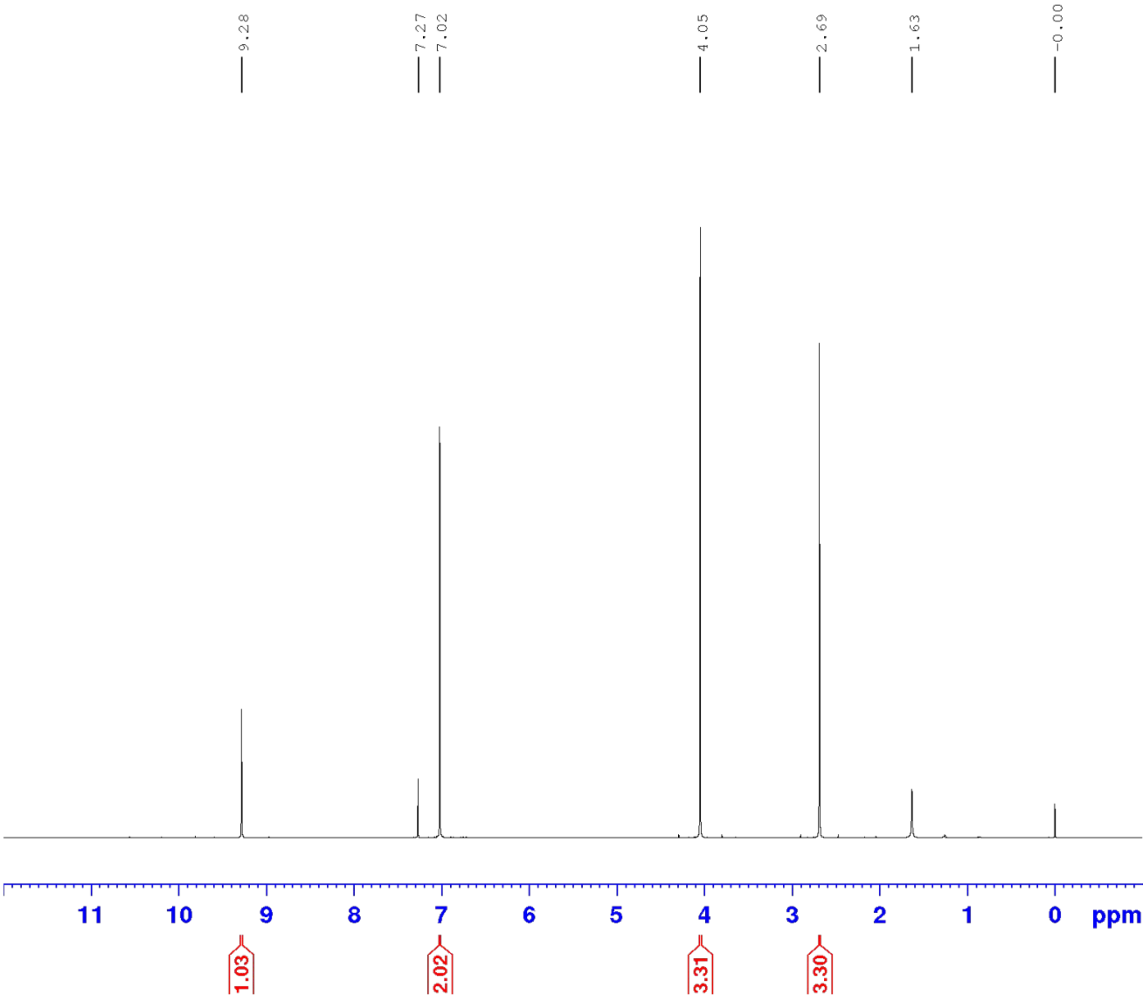

^{1}H NMR of **Compound 23**

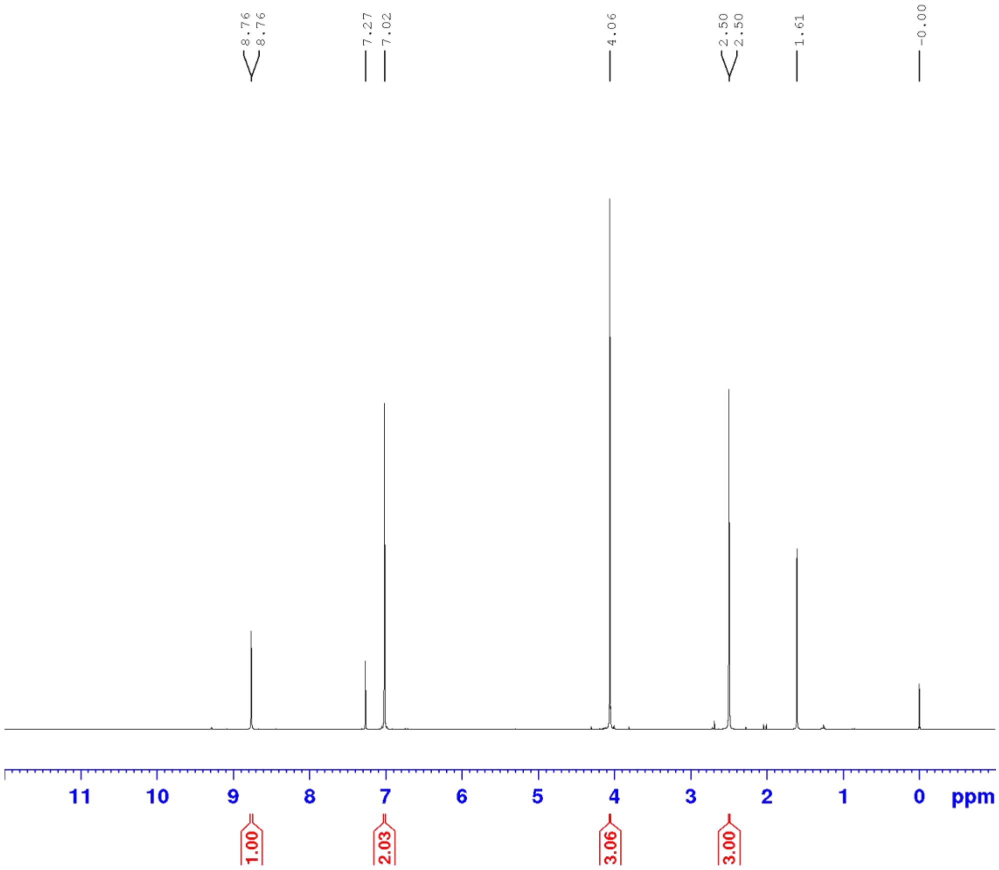

^{13}C NMR of **Compound 23**

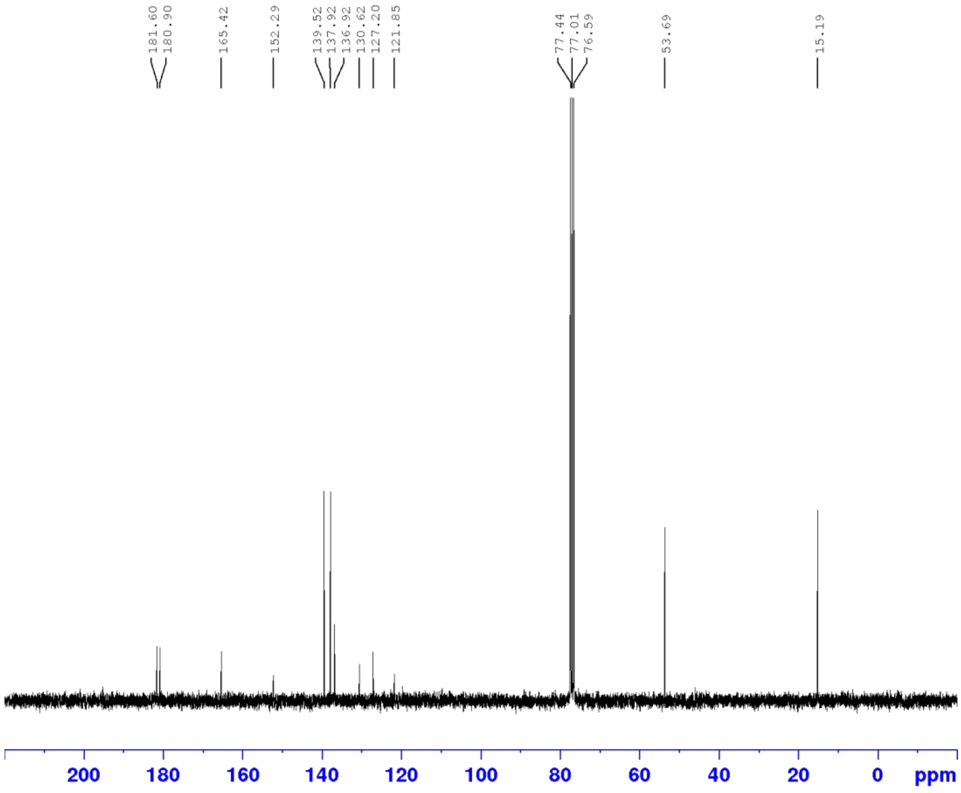

^{1}H NMR of **Compound 24**

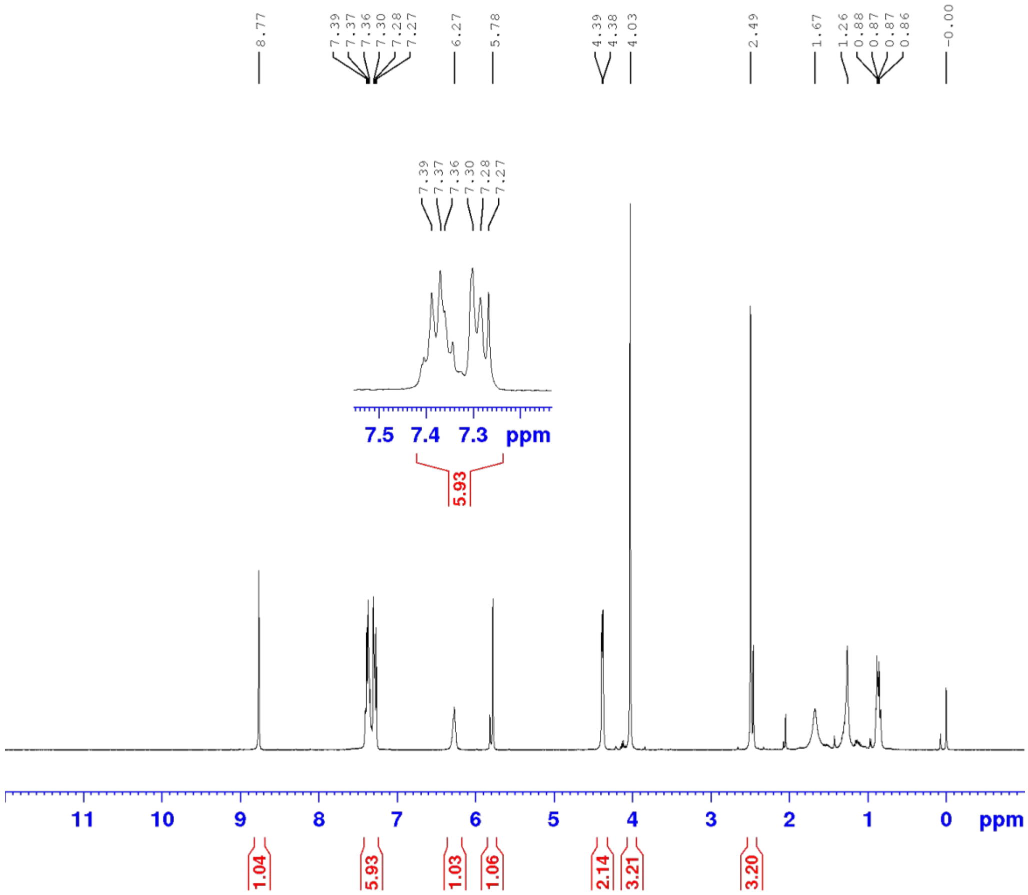

^{13}C NMR of **Compound 24**

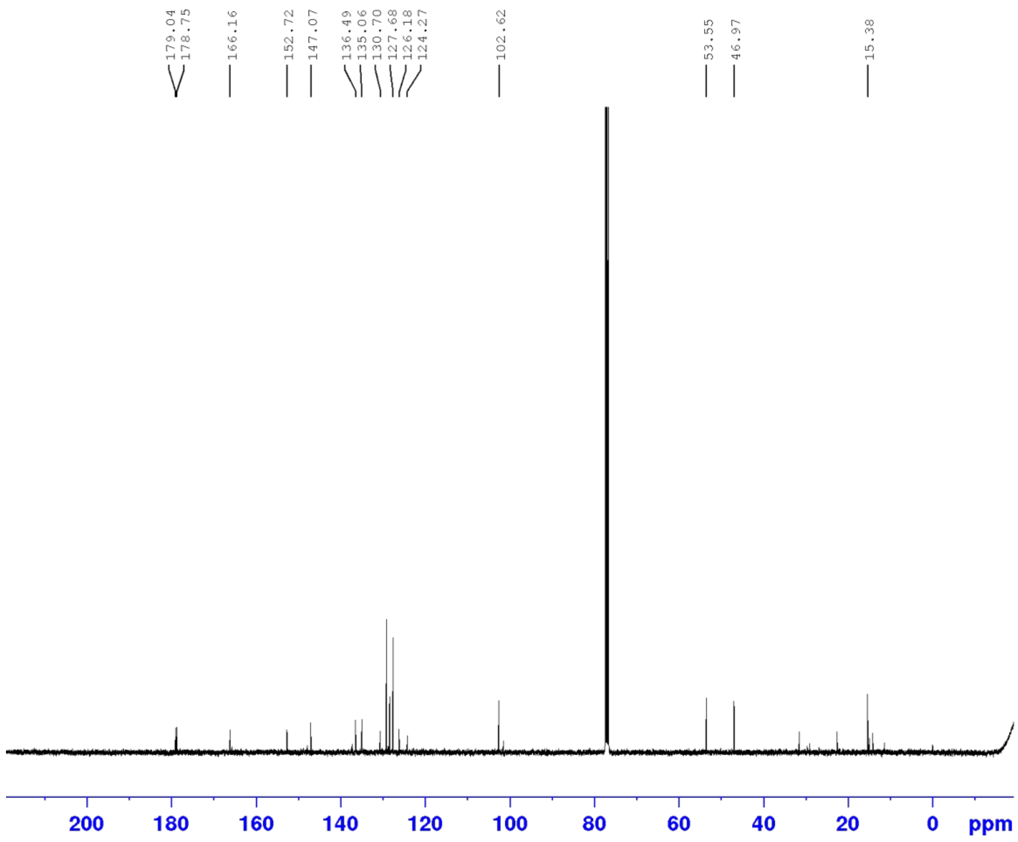

^{1}H NMR of **Compound 25**

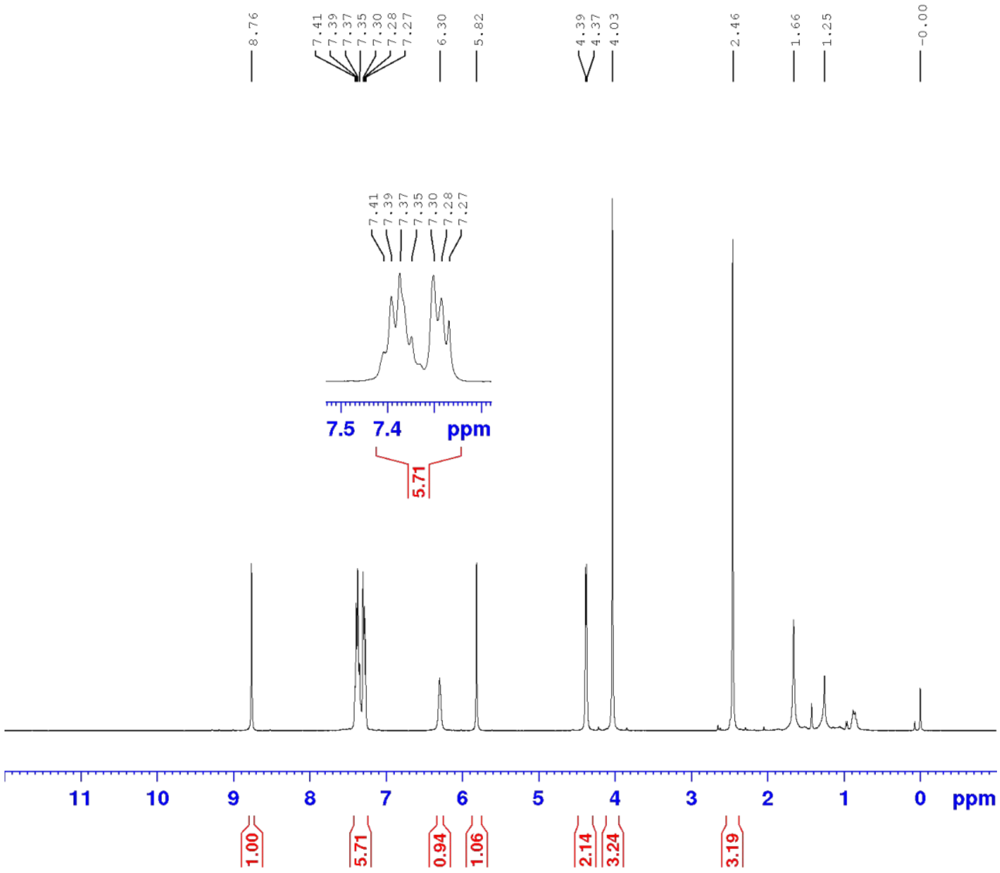

^{13}C NMR of **Compound 25**

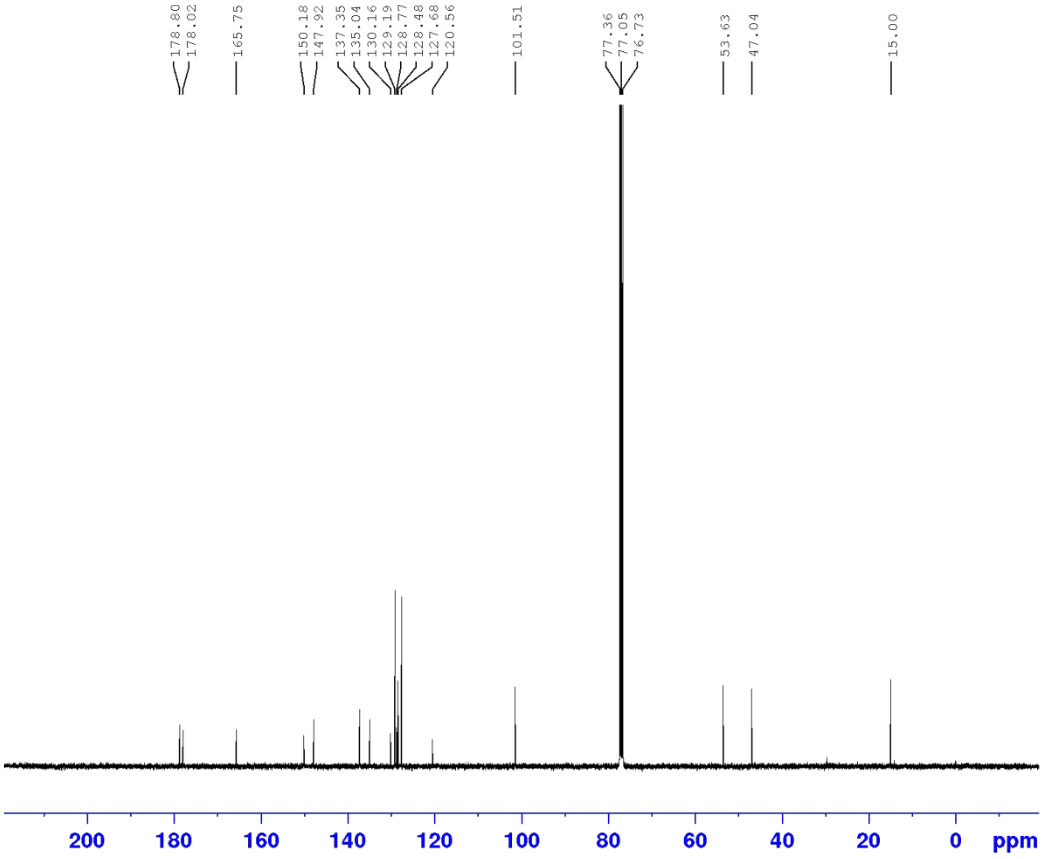

S2: NCI Data

S2.1 NCI Screening Assay Experimental details

NCI Screening Assay Experimental: Cancer cell growth assays (NCI60 screening) [5,6]

One-dose study: Tested compounds were initially solubilised in DMSO, diluted into RPMI 1640 and 5% fetal bovine serum/L-glutamine, and added to 96-well plates containing cell lines previously cultured for 24 h. After 48 h incubation, the media were removed, and the cells were fixed and stained with sulforhodamine B to determine overall percent growth/ total protein content. Unbound dye was removed with five washes of 1% acetic acid, and the plates were allowed to air dry. The dye was then resolubilised in Tris buffer, and the colorimetric absorbance was measured (515 nm). Growth inhibition was measured relative to the response generated from proliferating cells cultured under identical conditions for 48 h. Data from one-dose experiments pertains to the percentage growth at 10 μM.

Five-dose study: Serial 5 × 10-fold dilution from an initial DMSO stock solution was performed, prior to incubation at each individual concentration (10 nM, 100 nM, 1 μM, 10 μM and 100 μM). Using seven absorbance measurements (time zero (Tz), control growth (C), and test growth in the presence of drug at the five concentration levels (Ti)), the percentage growth was calculated at each of the drug concentrations levels. Percentage growth inhibition was calculated as: $[(Ti - Tz)/(C - Tz)] \times 100$ for concentrations for which $Ti \geq Tz$, $[(Ti - Tz)/Tz] \times 100$ for concentrations for which $Ti < Tz$. Three dose response parameters were calculated for each experimental agent. Growth inhibition of 50% (GI50) was calculated from $[(Ti - Tz)/(C - Tz)] \times 100 = 50$, which is the drug concentration resulting in a 50% reduction in the net protein increase (as measured by sulforhodamine B staining) in control cells during the drug incubation. The drug concentration resulting in total growth inhibition (TGI) was calculated from $Ti = Tz$. The LC50 (concentration of drug resulting in a 50% reduction in the measured protein at the end of the drug treatment as compared to that at the beginning) indicating a net loss of cells following treatment was calculated from $[(Ti - Tz)/Tz] \times 100 = -50$. Values were calculated for each of these three parameters if the level of activity was reached; however, if the effect was not reached or was exceeded, the value for that parameter was expressed as greater or less than the maximum or minimum concentration tested.

S2.2 One-dose and Five-dose data for compounds in sequence from 4 to 25

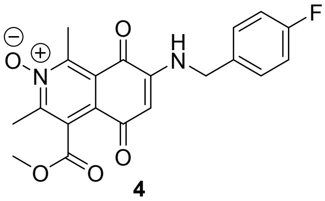

4

Developmental Therapeutics Program One Dose Mean Graph	NSC: D-810561 / 1	Conc: 1.00E-5 Molar	Test Date: Dec 03, 2018
	Experiment ID: 1812OS40		Report Date: Dec 20, 2018

Panel/Cell Line	Growth Percent
**Leukemia**	
HL-60(TB)	-51.54
K-562	9.12
MOLT-4	-19.63
RPMI-8226	26.86
SR	9.28
**Non-Small Cell Lung Cancer**	
A549/ATCC	4.34
EKVX	58.76
HOP-62	48.19
HOP-92	42.54
NCI-H226	81.62
NCI-H23	30.99
NCI-H322M	15.85
NCI-H460	11.51
NCI-H522	-11.59
**Colon Cancer**	
COLO 205	-49.78
HCC-2998	25.67
HCT-116	1.27
HCT-15	13.98
HT29	7.24
KM12	19.89
SW-620	6.37
**CNS Cancer**	
SF-268	22.97
SF-295	69.12
SF-539	0.76
SNB-19	3.72
SNB-75	128.08
U251	-13.93
**Melanoma**	
LOX IMVI	20.32
MALME-3M	-95.69
M14	2.89
MDA-MB-435	-72.94
SK-MEL-2	30.58
SK-MEL-28	72.37
SK-MEL-5	-47.89
UACC-257	-36.82
UACC-62	-35.51
**Ovarian Cancer**	
IGROV1	17.59
OVCAR-3	-21.00
OVCAR-4	-38.51
OVCAR-5	46.24
OVCAR-8	-5.16
NCI/ADR-RES	10.00
SK-OV-3	84.12
**Renal Cancer**	
786-0	48.68
A498	98.65
ACHN	48.28
CAKI-1	50.71
RXF 393	56.95
SN12C	-2.05
TK-10	81.42
UO-31	71.92
**Prostate Cancer**	
PC-3	3.57
DU-145	42.47
**Breast Cancer**	
MCF7	-0.80
MDA-MB-231/ATCC	1.94
HS 578T	82.63
BT-549	52.41
T-47D	6.75
MDA-MB-468	-9.79
Mean	17.90
Delta	113.59
Range	223.77

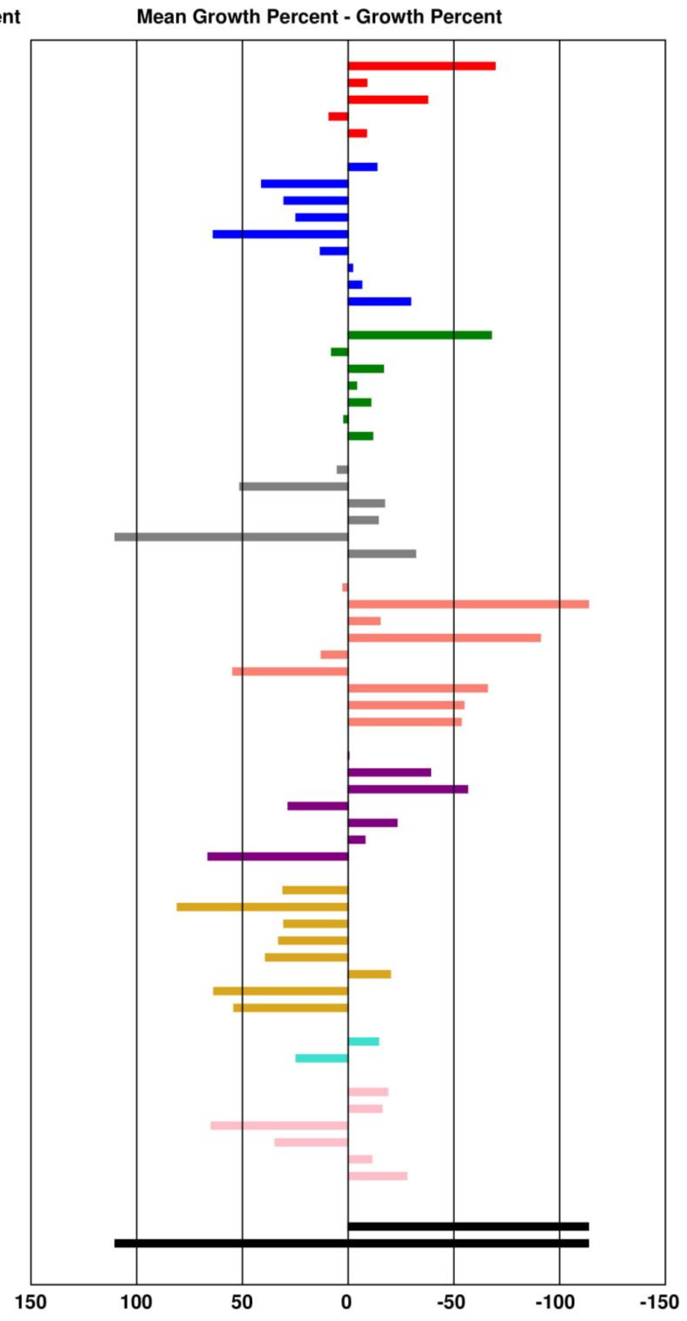

Figure S4 *NCI One Dose (10 µM) data for compound* ***4***

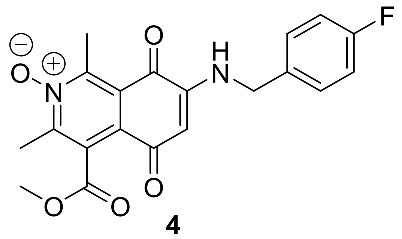

4

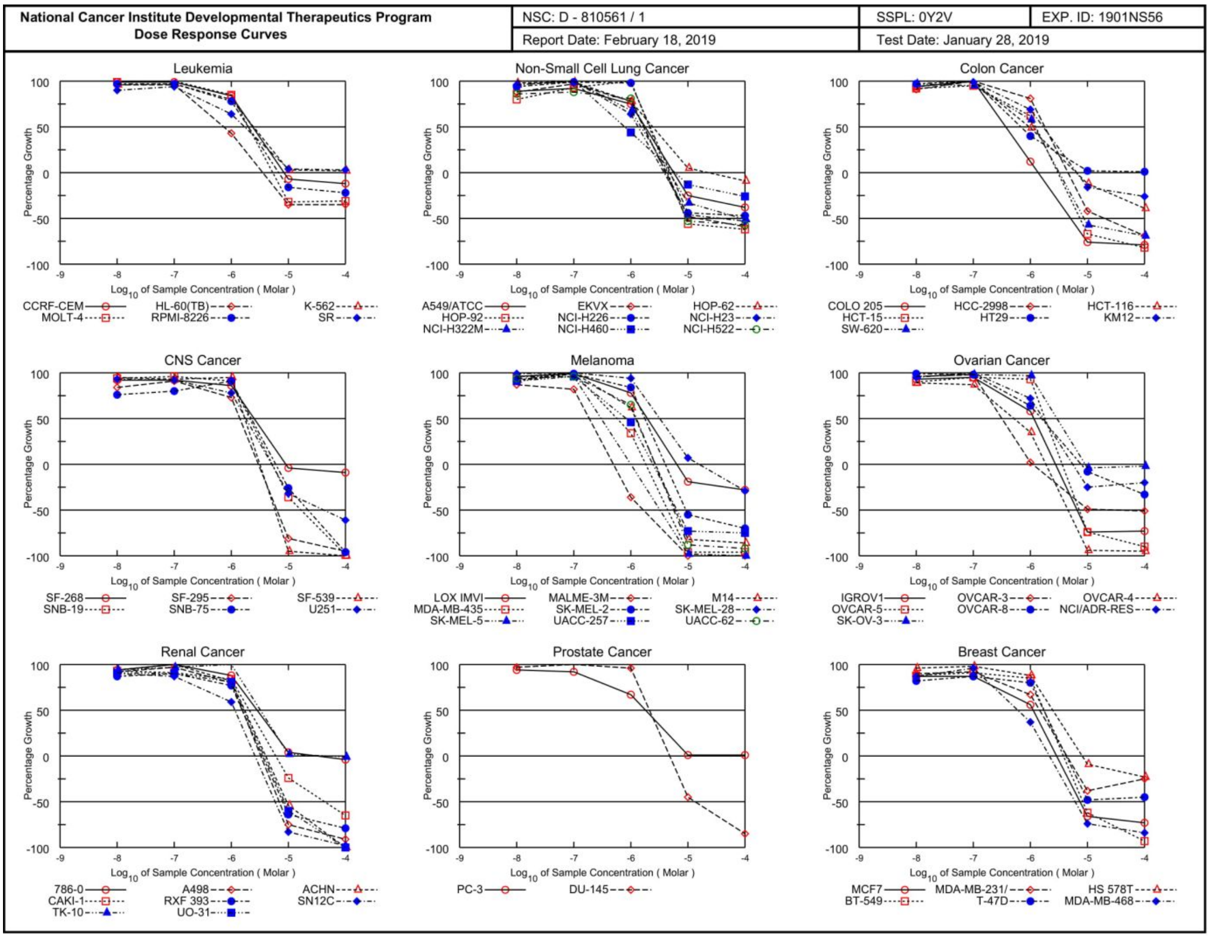

Figure S5** NCI Five Dose response summary data for compound **4

Table S2 *NCI Five Dose raw data for compound* ***4***

National Cancer Institute Developmental Therapeutics Program
In-Vitro Testing Results

NSC : D - 810561 / 1	Experiment ID : 1901NS56	Test Type : 08	Units : Molar
Report Date : February 18, 2019	Test Date : January 28, 2019	QNS :	MC :
COMI : RK 6.3.6	Stain Reagent : SRB Dual-Pass Related	SSPL : 0Y2V	

Log10 Concentration

Panel/Cell Line	Time Zero	Ctrl	Mean Optical Densities					Percent Growth					GI50	TGI	LC50
			-8.0	-7.0	-6.0	-5.0	-4.0	-8.0	-7.0	-6.0	-5.0	-4.0			
**Leukemia**															
CCRF-CEM	0.580	2.998	2.966	2.969	2.600	0.539	0.510	99	99	84	-7	-12	2.34E-6	8.36E-6	> 1.00E-4
HL-60(TB)	0.877	3.265	3.177	3.170	1.910	0.574	0.573	96	96	43	-35	-35	7.45E-7	3.60E-6	> 1.00E-4
K-562	0.214	2.586	2.495	2.516	2.103	0.284	0.256	96	97	80	3	2	2.43E-6	> 1.00E-4	> 1.00E-4
MOLT-4	0.550	2.892	2.861	2.885	2.532	0.375	0.380	99	100	85	-32	-31	1.98E-6	5.33E-6	> 1.00E-4
RPMI-8226	1.128	3.105	3.038	3.045	2.677	0.952	0.882	97	97	78	-16	-22	2.00E-6	6.82E-6	> 1.00E-4
SR	0.281	1.644	1.505	1.559	1.151	0.335	0.322	90	94	64	4	3	1.70E-6	> 1.00E-4	> 1.00E-4
**Non-Small Cell Lung Cancer**															
A549/ATCC	0.373	2.495	2.257	2.334	1.968	0.281	0.230	89	92	75	-25	-38	1.79E-6	5.66E-6	> 1.00E-4
EKVX	0.679	1.950	1.798	1.910	1.667	0.352	0.280	88	97	78	-48	-59	1.66E-6	4.14E-6	1.49E-5
HOP-62	0.451	1.964	1.936	1.950	1.627	0.525	0.411	98	99	78	5	-9	2.40E-6	2.25E-5	> 1.00E-4
HOP-92	1.205	2.032	1.866	1.973	2.122	0.528	0.462	80	93	111	-56	-62	2.31E-6	4.61E-6	9.18E-6
NCI-H226	1.465	3.063	2.966	3.044	3.023	0.817	0.775	94	99	98	-44	-47	2.16E-6	4.87E-6	> 1.00E-4
NCI-H23	0.538	1.654	1.610	1.651	1.250	0.289	0.253	96	100	64	-46	-53	1.33E-6	3.79E-6	3.54E-5
NCI-H322M	0.694	2.006	1.952	2.026	1.590	0.465	0.344	96	102	68	-33	-51	1.51E-6	4.72E-6	9.36E-5
NCI-H460	0.284	3.012	3.164	3.075	1.487	0.248	0.211	106	102	44	-13	-26	7.92E-7	5.98E-6	> 1.00E-4
NCI-H522	0.911	2.854	2.587	2.625	2.483	0.427	0.393	86	88	81	-53	-57	1.70E-6	4.01E-6	9.48E-6
**Colon Cancer**															
COLO 205	0.443	2.003	1.917	2.034	0.625	0.106	0.092	94	102	12	-76	-79	3.76E-7	1.36E-6	5.03E-6
HCC-2998	0.652	2.429	2.270	2.480	2.091	0.380	0.204	91	103	81	-42	-69	1.79E-6	4.57E-6	2.03E-5
HCT-116	0.242	2.514	2.386	2.517	1.356	0.212	0.148	94	100	49	-12	-39	9.57E-7	6.28E-6	> 1.00E-4
HCT-15	0.355	2.519	2.350	2.410	1.687	0.116	0.066	92	95	62	-67	-82	1.23E-6	3.00E-6	7.34E-6
HT29	0.240	2.130	2.070	2.268	0.997	0.284	0.263	97	107	40	2	1	7.11E-7	> 1.00E-4	> 1.00E-4
KM12	0.467	2.853	2.873	2.838	2.104	0.393	0.344	101	99	69	-16	-26	1.66E-6	6.49E-6	> 1.00E-4
SW-620	0.200	1.491	1.450	1.427	0.947	0.087	0.062	97	95	58	-57	-69	1.17E-6	3.21E-6	8.77E-6
**CNS Cancer**															
SF-268	0.759	2.556	2.414	2.421	2.302	0.731	0.690	92	92	86	-4	-9	2.51E-6	9.08E-6	> 1.00E-4
SF-295	0.484	1.701	1.507	1.594	1.378	0.094	0.025	84	91	73	-81	-95	1.42E-6	3.00E-6	6.33E-6
SF-539	0.884	2.739	2.641	2.615	2.637	0.044	0.002	95	93	95	-95	-100	1.72E-6	3.15E-6	5.79E-6
SNB-19	0.749	2.668	2.546	2.592	2.495	0.479	0.005	94	96	91	-36	-99	2.10E-6	5.20E-6	1.66E-5
SNB-75	0.890	1.760	1.553	1.588	1.680	0.656	0.040	76	80	91	-26	-96	2.23E-6	5.96E-6	2.20E-5
U251	0.256	1.723	1.621	1.600	1.404	0.175	0.099	93	92	78	-32	-61	1.81E-6	5.14E-6	4.13E-5
**Melanoma**															
LOX IMVI	0.406	2.977	2.880	2.964	2.414	0.327	0.294	96	99	78	-19	-28	1.94E-6	6.32E-6	> 1.00E-4
MALME-3M	0.679	1.751	1.615	1.559	0.432	-0.004	-0.014	87	82	-36	-100	-100	1.87E-7	4.93E-7	1.64E-6
M14	0.388	1.831	1.721	1.883	1.284	0.069	0.055	92	104	62	-82	-86	1.21E-6	2.69E-6	5.98E-6
MDA-MB-435	0.443	2.302	2.229	2.321	1.082	0.019	0.016	96	101	34	-96	-96	5.82E-7	1.84E-6	4.45E-6
SK-MEL-2	1.315	3.052	2.895	3.029	2.771	0.595	0.395	91	99	84	-55	-70	1.75E-6	4.03E-6	9.24E-6
SK-MEL-28	0.607	2.248	2.224	2.343	2.147	0.722	0.429	99	106	94	7	-29	3.20E-6	1.56E-5	> 1.00E-4
SK-MEL-5	0.752	3.199	3.103	3.094	0.753	0.013	0.004	96	96	.	-98	-100	3.00E-7	1.00E-6	3.23E-6
UACC-257	1.097	2.811	2.666	2.751	1.892	0.294	0.269	91	96	46	-73	-75	8.46E-7	2.44E-6	6.39E-6
UACC-62	1.057	3.125	3.003	3.040	2.393	0.127	0.081	94	96	65	-88	-92	1.25E-6	2.65E-6	5.63E-6
**Ovarian Cancer**															
IGROV1	0.358	1.942	1.836	1.856	1.277	0.094	0.095	93	95	58	-74	-73	1.15E-6	2.76E-6	6.60E-6
OVCAR-3	0.547	2.127	2.071	2.102	0.585	0.279	0.268	96	98	2	-49	-51	3.19E-7	1.11E-6	3.16E-5
OVCAR-4	0.555	1.324	1.239	1.225	0.826	0.031	0.028	89	87	35	-94	-95	5.19E-7	1.87E-6	4.54E-6
OVCAR-5	0.528	1.721	1.607	1.659	1.634	0.135	0.051	90	95	93	-74	-90	1.80E-6	3.59E-6	7.14E-6
OVCAR-8	0.418	2.376	2.348	2.333	1.681	0.385	0.280	99	98	64	-8	-33	1.59E-6	7.78E-6	> 1.00E-4
NCI/ADR-RES	0.621	2.243	2.165	2.282	1.784	0.467	0.497	95	102	72	-25	-20	1.68E-6	5.53E-6	> 1.00E-4
SK-OV-3	0.927	2.214	2.158	2.190	2.176	0.889	0.910	96	98	97	-4	-2	2.92E-6	9.11E-6	> 1.00E-4
**Renal Cancer**															
786-0	0.623	2.736	2.620	2.732	2.488	0.709	0.596	94	100	88	4	-4	2.85E-6	3.01E-5	> 1.00E-4
A498	1.828	2.878	2.791	2.846	2.673	0.452	0.163	92	97	80	-75	-91	1.57E-6	3.29E-6	6.88E-6
ACHN	0.320	1.628	1.548	1.661	1.399	0.149	-0.016	94	102	82	-54	-100	1.73E-6	4.04E-6	9.41E-6
CAKI-1	0.613	2.536	2.408	2.366	2.226	0.467	0.212	93	91	84	-24	-65	2.06E-6	6.00E-6	4.24E-5
RXF 393	1.197	1.740	1.672	1.685	1.616	0.429	0.250	87	90	77	-64	-79	1.56E-6	3.51E-6	7.94E-6
SN12C	0.664	2.693	2.500	2.426	1.871	0.115	0.013	90	87	59	-83	-98	1.17E-6	2.62E-6	5.88E-6
TK-10	0.733	2.290	2.198	2.237	2.327	0.761	0.723	94	97	102	2	-1	3.32E-6	3.67E-5	> 1.00E-4
UO-31	0.630	1.981	1.857	1.848	1.730	0.253	-0.010	91	90	81	-60	-100	1.67E-6	3.77E-6	8.51E-6
**Prostate Cancer**															
PC-3	0.511	2.361	2.250	2.205	1.742	0.528	0.525	94	92	67	1	1	1.79E-6	> 1.00E-4	> 1.00E-4
DU-145	0.346	1.759	1.721	1.795	1.697	0.189	0.051	97	102	96	-45	-85	2.11E-6	4.76E-6	1.30E-5
**Breast Cancer**															
MCF7	0.309	1.888	1.678	1.685	1.189	0.105	0.082	87	87	56	-66	-73	1.11E-6	2.86E-6	7.37E-6
MDA-MB-231/ATCC	0.656	1.662	1.561	1.583	1.332	0.407	0.492	90	92	67	-38	-25	1.46E-6	4.36E-6	> 1.00E-4
HS 578T	0.915	1.961	1.924	1.936	1.840	0.830	0.701	96	98	88	-9	-23	2.47E-6	8.03E-6	> 1.00E-4
BT-549	1.174	2.620	2.442	2.496	2.400	0.447	0.082	88	91	85	-62	-93	1.73E-6	3.78E-6	8.29E-6
T-47D	1.075	2.782	2.476	2.568	2.444	0.561	0.588	82	87	80	-48	-45	1.72E-6	4.23E-6	> 1.00E-4
MDA-MB-468	0.915	1.605	1.517	1.580	1.168	0.238	0.143	87	96	37	-74	-84	5.98E-7	2.15E-6	6.07E-6

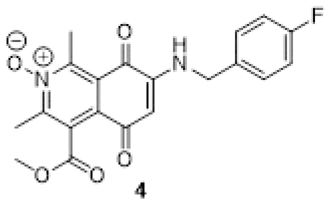

4

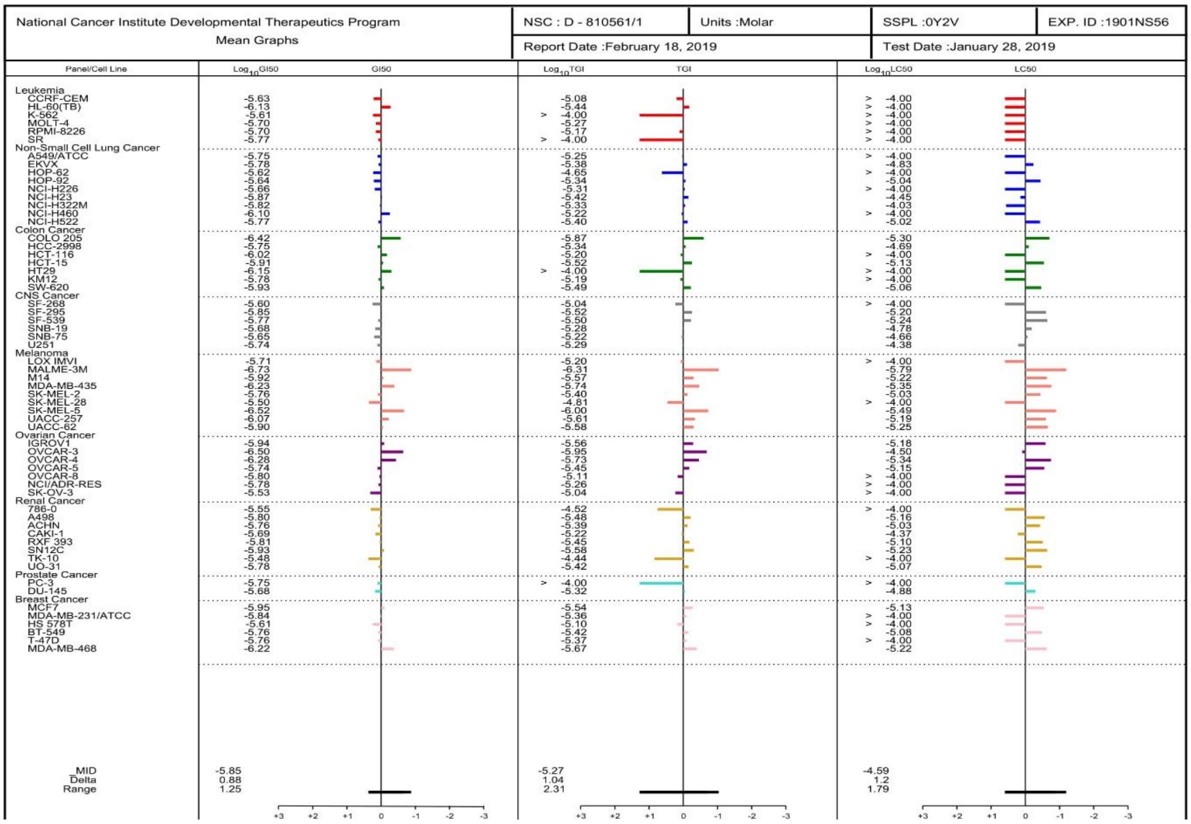

Mean of GI_{50} across 57 cell lines for compound **4** as Log_{10} Concentration (SD): -5.851 (±0.26)

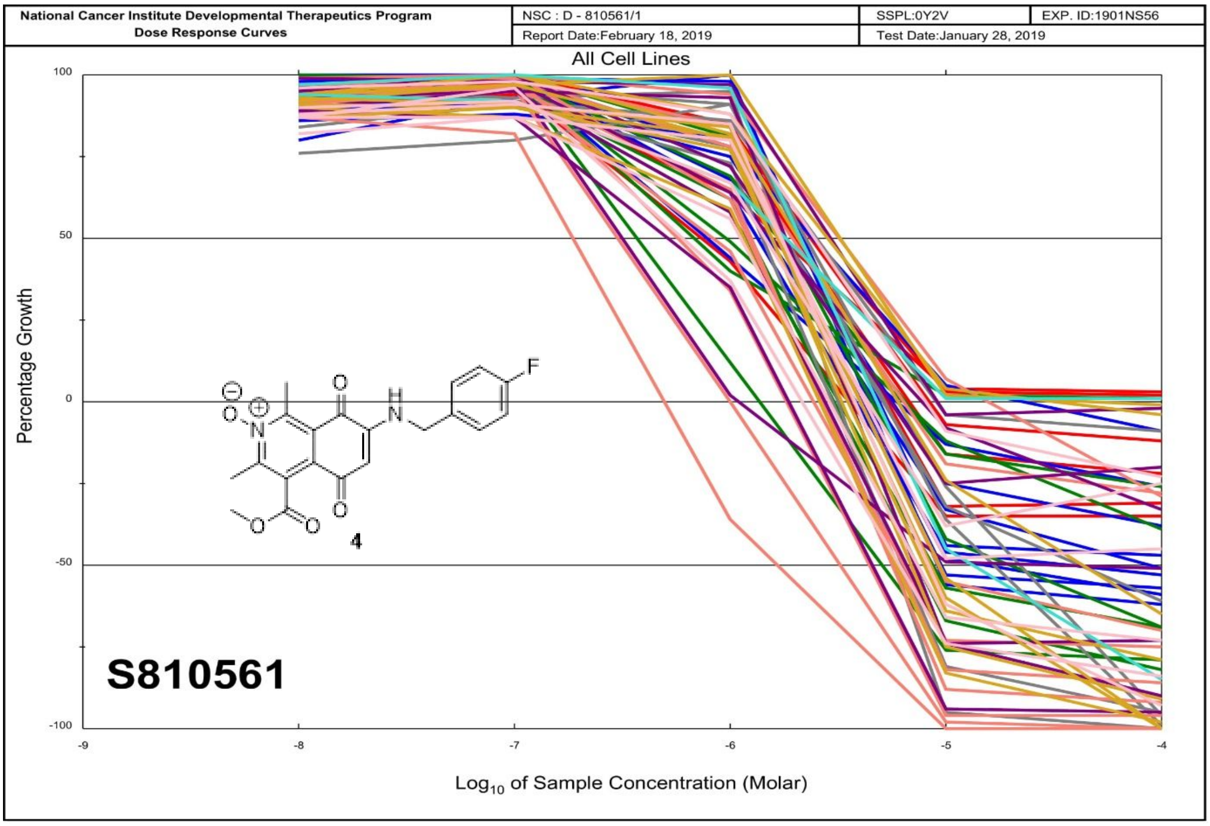

Figure S6 *NCI Five Dose data cell line comparison summary for compound* ***4***

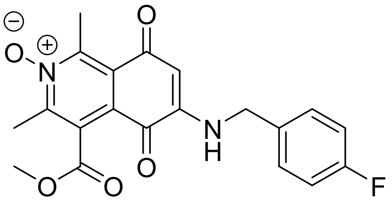

5

Developmental Therapeutics Program One Dose Mean Graph	NSC: D-815401 / 1	Conc: 1.00E-5 Molar	Test Date: May 20, 2019
	Experiment ID: 1905OS11		Report Date: Jun 10, 2019

Panel/Cell Line	Growth Percent
Leukemia	
CCRF-CEM	4.77
HL-60(TB)	-29.43
K-562	1.64
MOLT-4	-35.15
RPMI-8226	-20.91
SR	2.14
Non-Small Cell Lung Cancer	
A549/ATCC	-22.67
EKVX	-57.01
HOP-62	-6.11
HOP-92	-41.08
NCI-H23	-46.24
NCI-H322M	-6.43
NCI-H460	-0.65
NCI-H522	-15.15
Colon Cancer	
COLO 205	-59.22
HCC-2998	-40.21
HCT-116	0.36
HCT-15	-60.50
HT29	2.67
KM12	1.23
SW-620	-9.71
CNS Cancer	
SF-268	-1.99
SF-295	-80.04
SF-539	-91.16
SNB-19	-66.50
SNB-75	-91.98
U251	-23.07
Melanoma	
LOX IMVI	-47.07
MALME-3M	-97.80
M14	-69.27
MDA-MB-435	-93.06
SK-MEL-2	-31.29
SK-MEL-28	6.43
SK-MEL-5	-96.82
UACC-257	-87.15
UACC-62	-81.66
Ovarian Cancer	
IGROV1	-59.40
OVCAR-3	-35.92
OVCAR-5	-68.79
OVCAR-8	2.54
NCI/ADR-RES	-16.70
SK-OV-3	-5.79
Renal Cancer	
786-0	5.86
ACHN	-45.22
CAKI-1	-0.89
RXF 393	-22.16
SN12C	-88.84
TK-10	13.55
UO-31	-21.00
Prostate Cancer	
PC-3	2.62
DU-145	-90.64
Breast Cancer	
MCF7	-60.62
MDA-MB-231/ATCC	-29.34
HS 578T	-16.64
BT-549	-91.33
T-47D	-41.05
MDA-MB-468	-66.98
Mean	-37.31
Delta	60.49
Range	111.35

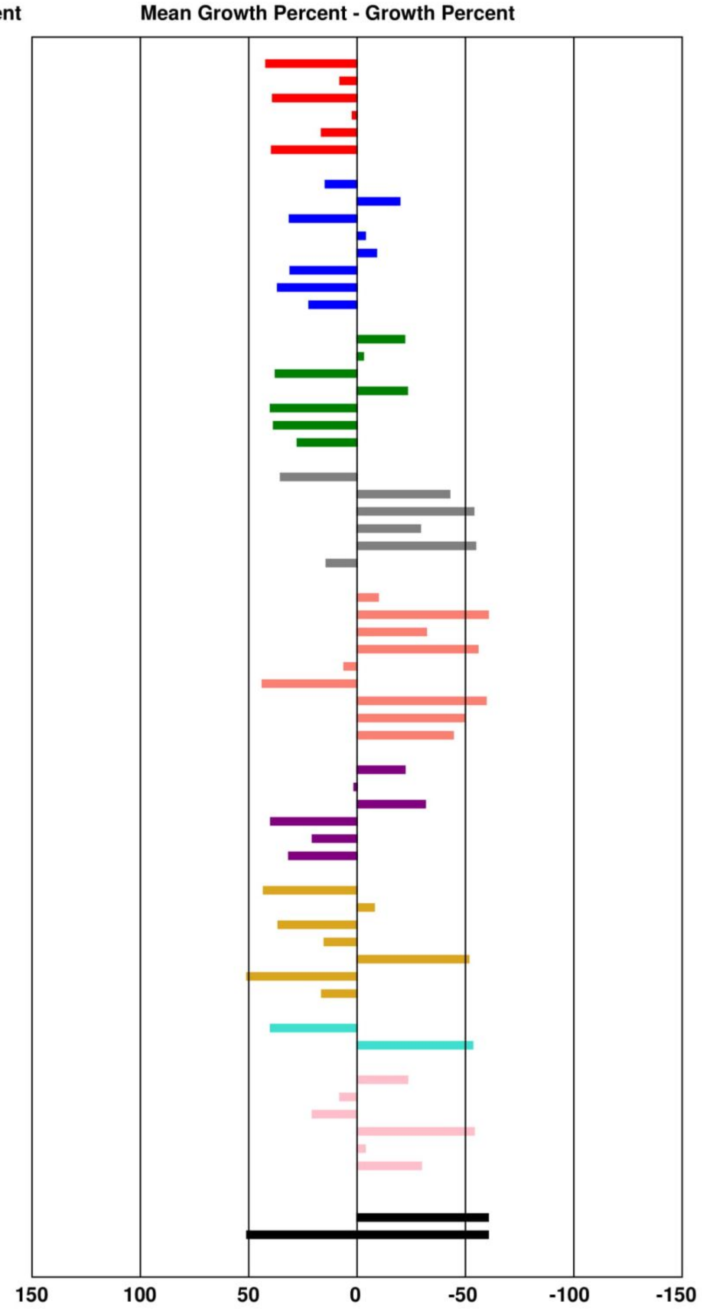

Figure S7 *NCI One Dose (10 µM) data for compound* ***5***

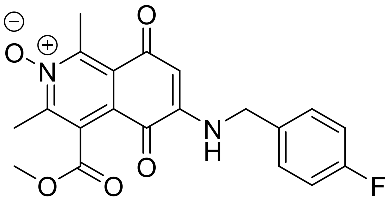

5

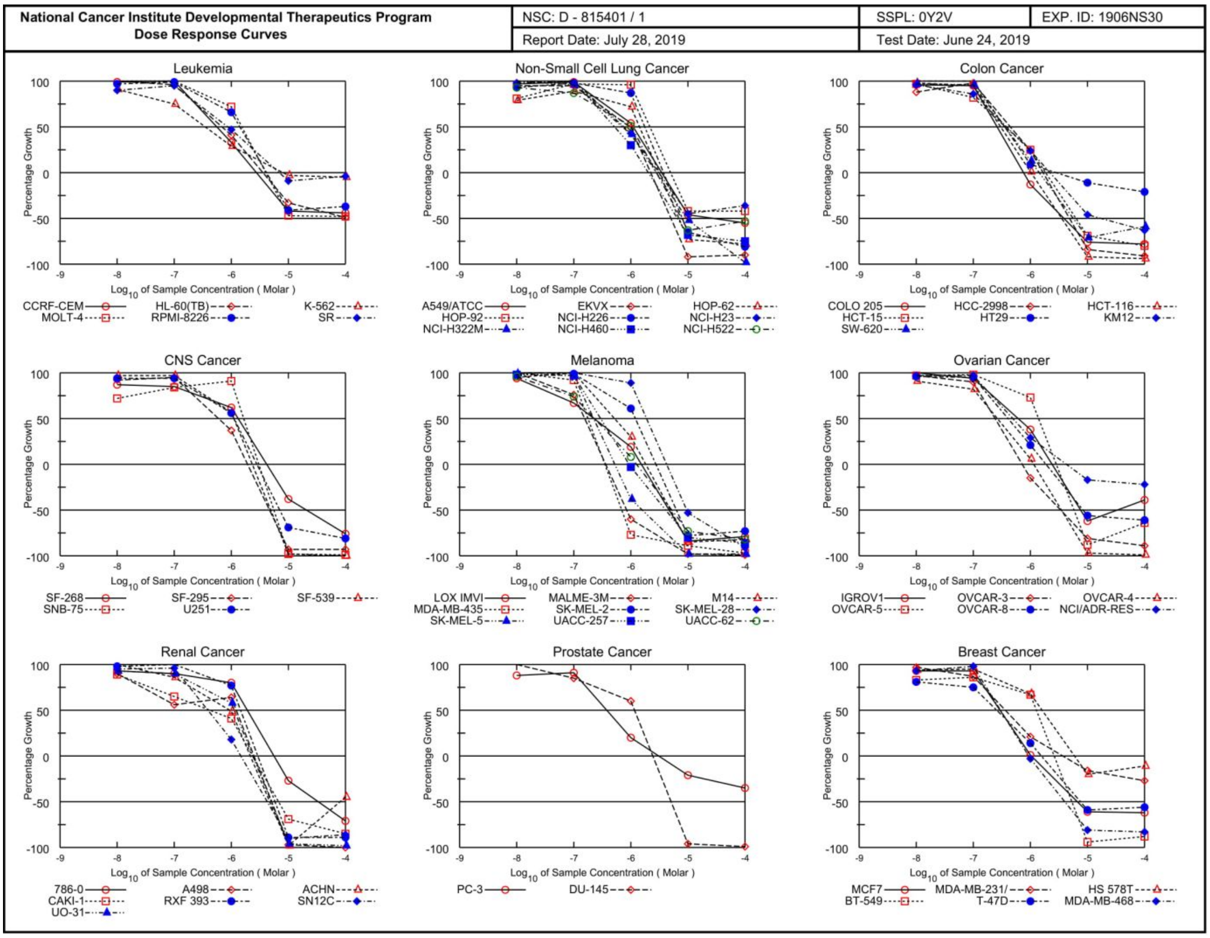

Figure S8** NCI Five Dose response summary data for compound **5

Table S3 *NCI Five Dose raw data for compound **5***

National Cancer Institute Developmental Therapeutics Program
In-Vitro Testing Results

NSC : D - 815401 / 1	Experiment ID : 1906NS30	Test Type : 08	Units : Molar
Report Date : July 28, 2019	Test Date : June 24, 2019	QNS :	MC :
COMI : RK 6.3.6.1	Stain Reagent : SRB Dual-Pass Related	SSPL : 0Y2V	

			Log10 Concentration													
	Time		Mean Optical Densities					Percent Growth								
Panel/Cell Line	Zero	Ctrl	-8.0	-7.0	-6.0	-5.0	-4.0	-8.0	-7.0	-6.0	-5.0	-4.0	GI50	TGI	LC50
Leukemia															
CCRF-CEM	0.555	2.248	2.233	2.273	1.107	0.323	0.311	99	101	33	-42	-44	5.58E-7	2.74E-6	> 1.00E-4
HL-60(TB)	0.970	3.208	3.228	3.116	1.880	0.653	0.496	101	96	41	-33	-49	6.77E-7	3.58E-6	> 1.00E-4
K-562	0.175	1.331	1.224	1.040	0.515	0.171	0.166	91	75	29	-3	-5	3.52E-7	8.31E-6	> 1.00E-4
MOLT-4	0.690	2.640	2.680	2.784	2.089	0.366	0.362	102	107	72	-47	-48	1.52E-6	4.02E-6	> 1.00E-4
RPMI-8226	0.896	2.641	2.585	2.625	2.039	0.527	0.565	97	99	66	-41	-37	1.40E-6	4.11E-6	> 1.00E-4
SR	0.401	1.202	1.119	1.161	0.774	0.365	0.384	90	95	47	-9	-4	8.50E-7	6.86E-6	> 1.00E-4
Non-Small Cell Lung Cancer															
A549/ATCC	0.290	1.823	1.775	1.807	1.112	0.156	0.132	97	99	54	-46	-55	1.09E-6	3.44E-6	2.74E-5
EKVX	0.554	1.791	1.729	1.725	1.163	0.045	0.054	95	95	49	-92	-90	9.63E-7	2.23E-6	5.04E-6
HOP-62	0.711	1.841	1.607	1.721	1.526	0.194	0.155	79	89	72	-73	-78	1.42E-6	3.14E-6	6.96E-6
HOP-92	1.036	1.563	1.464	1.548	1.543	0.602	0.600	81	97	96	-42	-42	2.16E-6	4.97E-6	> 1.00E-4
NCI-H226	1.126	2.430	2.339	2.400	2.259	0.395	0.216	93	98	87	-65	-81	1.75E-6	3.74E-6	7.98E-6
NCI-H23	0.613	1.958	1.927	1.978	1.195	0.338	0.390	98	101	43	-45	-36	7.66E-7	3.09E-6	> 1.00E-4
NCI-H322M	0.743	2.016	2.043	1.970	1.273	0.360	0.018	102	96	42	-52	-98	7.04E-7	2.80E-6	9.62E-6
NCI-H460	0.287	2.865	2.964	2.874	1.073	0.091	0.073	104	100	30	-68	-75	5.25E-7	2.03E-6	6.51E-6
NCI-H522	0.820	2.280	2.179	2.090	1.572	0.305	0.384	93	87	51	-63	-53	1.03E-6	2.82E-6	7.73E-6
Colon Cancer															
COLO 205	0.585	2.227	2.166	2.141	0.509	0.140	0.128	96	95	-13	-76	-78	2.60E-7	7.58E-7	3.86E-6
HCC-2998	0.932	2.615	2.413	2.590	1.329	0.151	0.084	88	98	24	-84	-91	4.44E-7	1.66E-6	4.84E-6
HCT-116	0.244	1.933	1.903	1.844	0.262	0.019	0.015	98	95	1	-92	-94	3.00E-7	1.03E-6	3.53E-6
HCT-15	0.278	2.091	2.040	1.768	0.725	0.086	0.055	97	82	25	-69	-80	3.63E-7	1.83E-6	6.24E-6
HT29	0.208	1.606	1.607	1.704	0.328	0.186	0.164	100	107	9	-11	-21	3.79E-7	2.76E-6	> 1.00E-4
KM12	0.579	2.943	2.842	2.615	1.144	0.311	0.213	96	86	24	-46	-63	3.81E-7	2.19E-6	1.64E-5
SW-620	0.258	2.021	1.964	1.947	0.491	0.075	0.107	97	96	13	-71	-59	3.58E-7	1.43E-6	5.62E-6
CNS Cancer															
SF-268	0.838	2.476	2.267	2.237	1.851	0.523	0.202	87	85	62	-38	-76	1.32E-6	4.18E-6	2.10E-5
SF-295	0.781	2.888	2.728	2.776	1.566	0.055	0.052	92	95	37	-93	-93	6.00E-7	1.93E-6	4.68E-6
SF-539	1.054	2.853	2.808	2.793	2.074	0.021	0.004	97	97	57	-98	-100	1.10E-6	2.32E-6	4.89E-6
SNB-75	1.027	1.815	1.597	1.686	1.745	0.017	0.008	72	84	91	-98	-99	1.65E-6	3.03E-6	5.56E-6
U251	0.272	1.505	1.434	1.430	0.963	0.085	0.051	94	94	56	-69	-81	1.12E-6	2.81E-6	7.05E-6
Melanoma															
LOX IMVI	0.496	2.718	2.580	1.990	0.912	0.080	0.107	94	67	19	-84	-79	2.26E-7	1.52E-6	4.67E-6
MALME-3M	0.763	1.652	1.648	1.439	0.304	0.016	0.008	100	76	-60	-98	-99	1.55E-7	3.62E-7	8.41E-7
M14	0.430	1.690	1.631	1.720	0.804	0.066	0.080	95	102	30	-85	-82	5.25E-7	1.82E-6	4.97E-6
MDA-MB-435	0.512	2.551	2.573	2.381	0.119	0.058	0.016	101	92	-77	-89	-97	1.77E-7	3.50E-7	6.94E-7
SK-MEL-2	1.127	2.488	2.525	2.471	1.960	0.247	0.299	103	99	61	-78	-73	1.20E-6	2.75E-6	6.28E-6
SK-MEL-28	0.772	1.999	2.119	2.210	1.866	0.362	0.067	110	117	89	-53	-91	1.88E-6	4.23E-6	9.51E-6
SK-MEL-5	0.784	3.077	3.050	2.999	0.485	0.015	0.016	99	97	-38	-98	-98	2.22E-7	5.21E-7	1.58E-6
UACC-257	0.739	1.852	1.826	1.824	0.721	0.146	0.101	98	97	-3	-80	-86	2.98E-7	9.44E-7	4.08E-6
UACC-62	0.975	2.664	2.588	2.220	1.108	0.260	0.176	96	74	8	-73	-82	2.29E-7	1.25E-6	5.16E-6
Ovarian Cancer															
IGROV1	0.547	2.238	2.247	2.130	1.197	0.207	0.331	100	94	38	-62	-39	6.17E-7	2.41E-6	.
OVCAR-3	0.469	1.575	1.537	1.463	0.400	0.089	0.050	97	90	-15	-81	-89	2.40E-7	7.22E-7	3.40E-6
OVCAR-4	0.560	1.511	1.427	1.344	0.617	0.018	0.008	91	82	6	-97	-99	2.65E-7	1.14E-6	3.50E-6
OVCAR-5	0.600	1.637	1.605	1.619	1.359	0.073	0.215	97	98	73	-88	-64	1.39E-6	2.85E-6	5.82E-6
OVCAR-8	0.444	2.285	2.208	2.193	0.832	0.195	0.174	96	95	21	-56	-61	4.06E-7	1.88E-6	8.34E-6
NCI/ADR-RES	0.563	2.045	1.994	1.998	0.989	0.465	0.437	97	97	29	-17	-22	4.87E-7	4.20E-6	> 1.00E-4
Renal Cancer															
786-0	0.921	2.683	2.560	2.508	2.323	0.670	0.267	93	90	80	-27	-71	1.89E-6	5.55E-6	3.31E-5
A498	1.553	2.131	2.073	1.876	1.922	0.052	0.006	90	56	64	-97	-100	1.22E-6	2.50E-6	5.12E-6
ACHN	0.354	1.665	1.682	1.487	1.001	0.008	0.196	101	86	49	-98	-45	9.58E-7	2.16E-6	.
CAKI-1	0.772	2.668	2.451	2.000	1.557	0.236	0.117	89	65	41	-69	-85	4.29E-7	2.36E-6	6.68E-6
RXF 393	0.883	1.647	1.630	1.706	1.469	0.099	0.093	98	108	77	-89	-89	1.45E-6	2.91E-6	5.83E-6
SN12C	0.558	1.985	1.913	1.931	0.819	0.057	0.081	95	96	18	-90	-86	3.92E-7	1.48E-6	4.28E-6
UO-31	0.542	1.686	1.609	1.572	1.202	0.023	0.012	93	90	58	-96	-98	1.12E-6	2.38E-6	5.03E-6
Prostate Cancer															
PC-3	0.573	1.579	1.455	1.489	0.771	0.452	0.371	88	91	20	-21	-35	3.76E-7	3.04E-6	> 1.00E-4
DU-145	0.410	1.733	1.727	1.537	1.199	0.017	0.005	100	85	60	-96	-99	1.15E-6	2.42E-6	5.07E-6
Breast Cancer															
MCF7	0.467	2.443	2.308	2.312	0.494	0.180	0.178	93	93	1	-61	-62	2.96E-7	1.05E-6	6.57E-6
MDA-MB-231/ATCC	0.775	1.875	1.845	1.737	1.004	0.653	0.569	97	87	21	-16	-27	3.64E-7	3.71E-6	> 1.00E-4
HS 578T	0.869	1.938	1.870	1.888	1.596	0.693	0.771	94	95	68	-20	-11	1.60E-6	5.89E-6	> 1.00E-4
BT-549	1.031	1.922	1.769	1.799	1.625	0.067	0.119	83	86	67	-94	-88	1.27E-6	2.61E-6	5.35E-6
T-47D	0.723	1.640	1.465	1.407	0.856	0.294	0.319	81	75	14	-59	-56	2.56E-7	1.57E-6	7.47E-6
MDA-MB-468	0.732	1.362	1.317	1.353	0.709	0.138	0.123	93	98	-3	-81	-83	3.00E-7	9.31E-7	3.99E-6

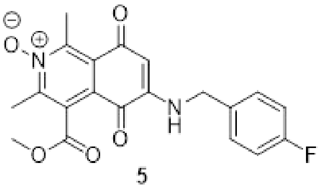

5

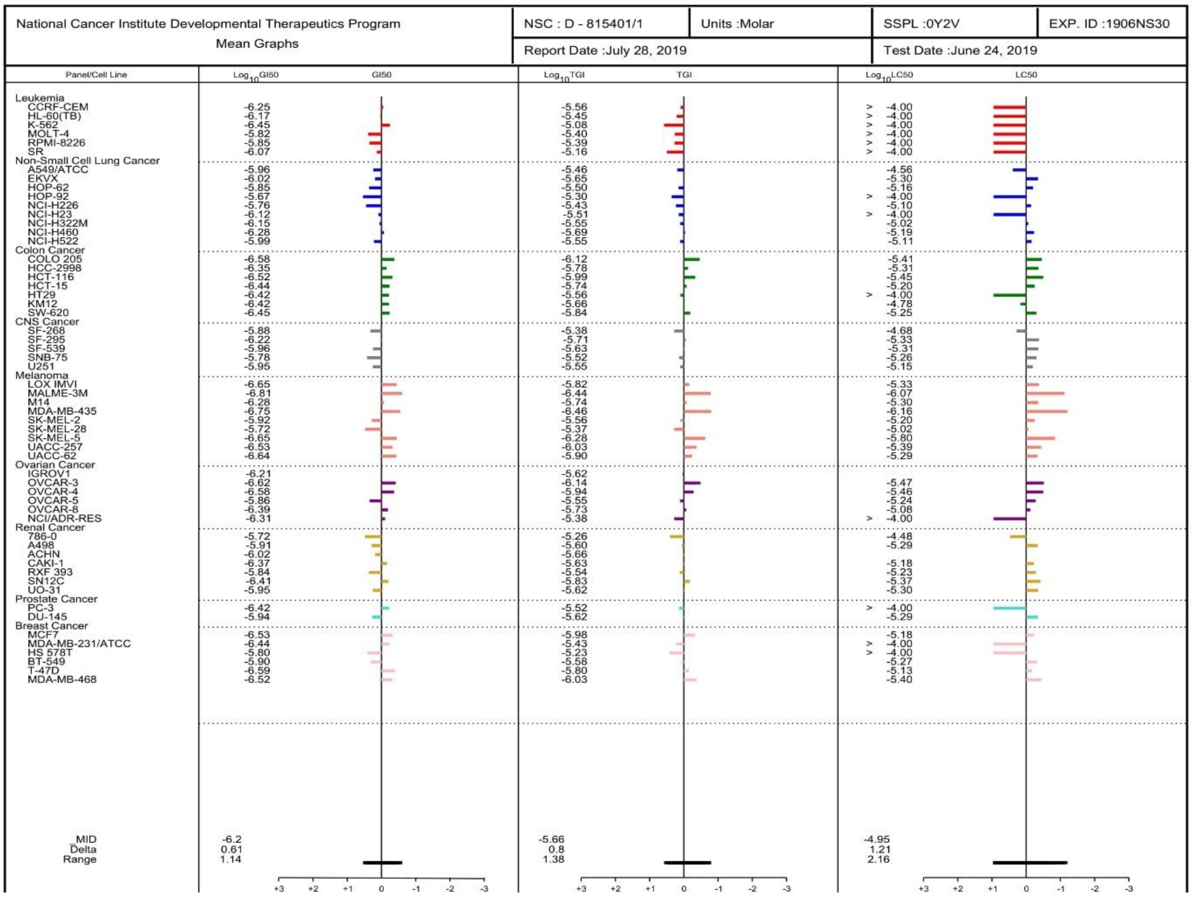

Mean of GI_{50} across 58 cell lines for compound **5** as Log_{10} Concentration (SD): -6.234 (±0.33)

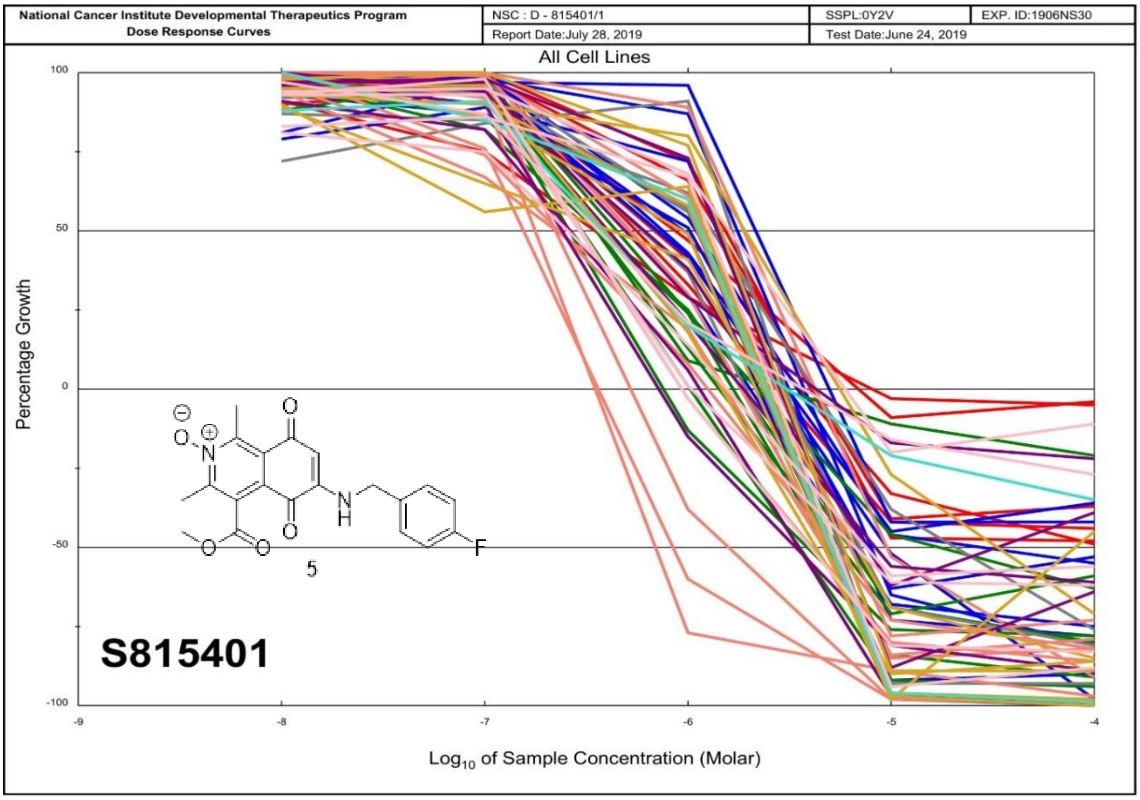

Figure S9** NCI Five Dose data cell line comparison summary for compound **5

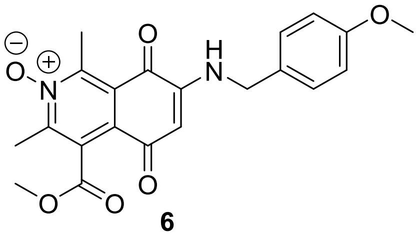

6

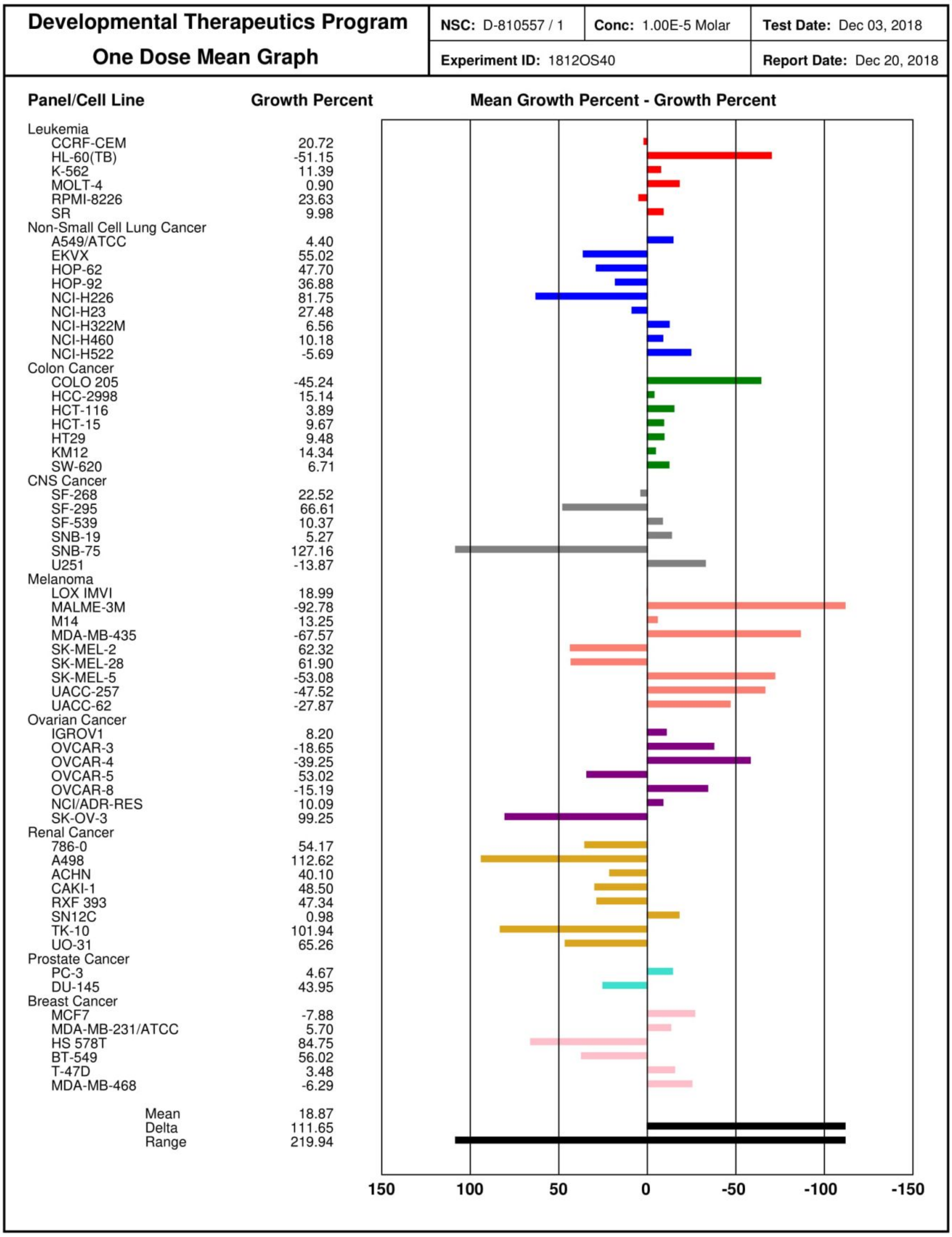

Developmental Therapeutics Program	NSC: D-810557 / 1	Conc: 1.00E-5 Molar	Test Date: Dec 03, 2018
One Dose Mean Graph	Experiment ID: 1812OS40		Report Date: Dec 20, 2018

Panel/Cell Line	Growth Percent
Leukemia	
CCRF-CEM	20.72
HL-60(TB)	-51.15
K-562	11.39
MOLT-4	0.90
RPMI-8226	23.63
SR	9.98
Non-Small Cell Lung Cancer	
A549/ATCC	4.40
EKVX	55.02
HOP-62	47.70
HOP-92	36.88
NCI-H226	81.75
NCI-H23	27.48
NCI-H322M	6.56
NCI-H460	10.18
NCI-H522	-5.69
Colon Cancer	
COLO 205	-45.24
HCC-2998	15.14
HCT-116	3.89
HCT-15	9.67
HT29	9.48
KM12	14.34
SW-620	6.71
CNS Cancer	
SF-268	22.52
SF-295	66.61
SF-539	10.37
SNB-19	5.27
SNB-75	127.16
U251	-13.87
Melanoma	
LOX IMVI	18.99
MALME-3M	-92.78
M14	13.25
MDA-MB-435	-67.57
SK-MEL-2	62.32
SK-MEL-28	61.90
SK-MEL-5	-53.08
UACC-257	-47.52
UACC-62	-27.87
Ovarian Cancer	
IGROV1	8.20
OVCAR-3	-18.65
OVCAR-4	-39.25
OVCAR-5	53.02
OVCAR-8	-15.19
NCI/ADR-RES	10.09
SK-OV-3	99.25
Renal Cancer	
786-0	54.17
A498	112.62
ACHN	40.10
CAKI-1	48.50
RXF 393	47.34
SN12C	0.98
TK-10	101.94
UO-31	65.26
Prostate Cancer	
PC-3	4.67
DU-145	43.95
Breast Cancer	
MCF7	-7.88
MDA-MB-231/ATCC	5.70
HS 578T	84.75
BT-549	56.02
T-47D	3.48
MDA-MB-468	-6.29
Mean	18.87
Delta	111.65
Range	219.94

Figure S10 *NCI One Dose (10 μM) data for compound* ***6***

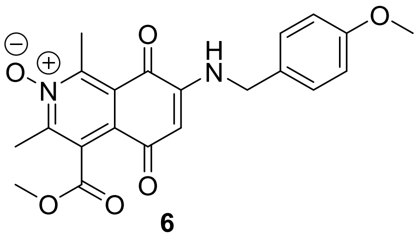

6

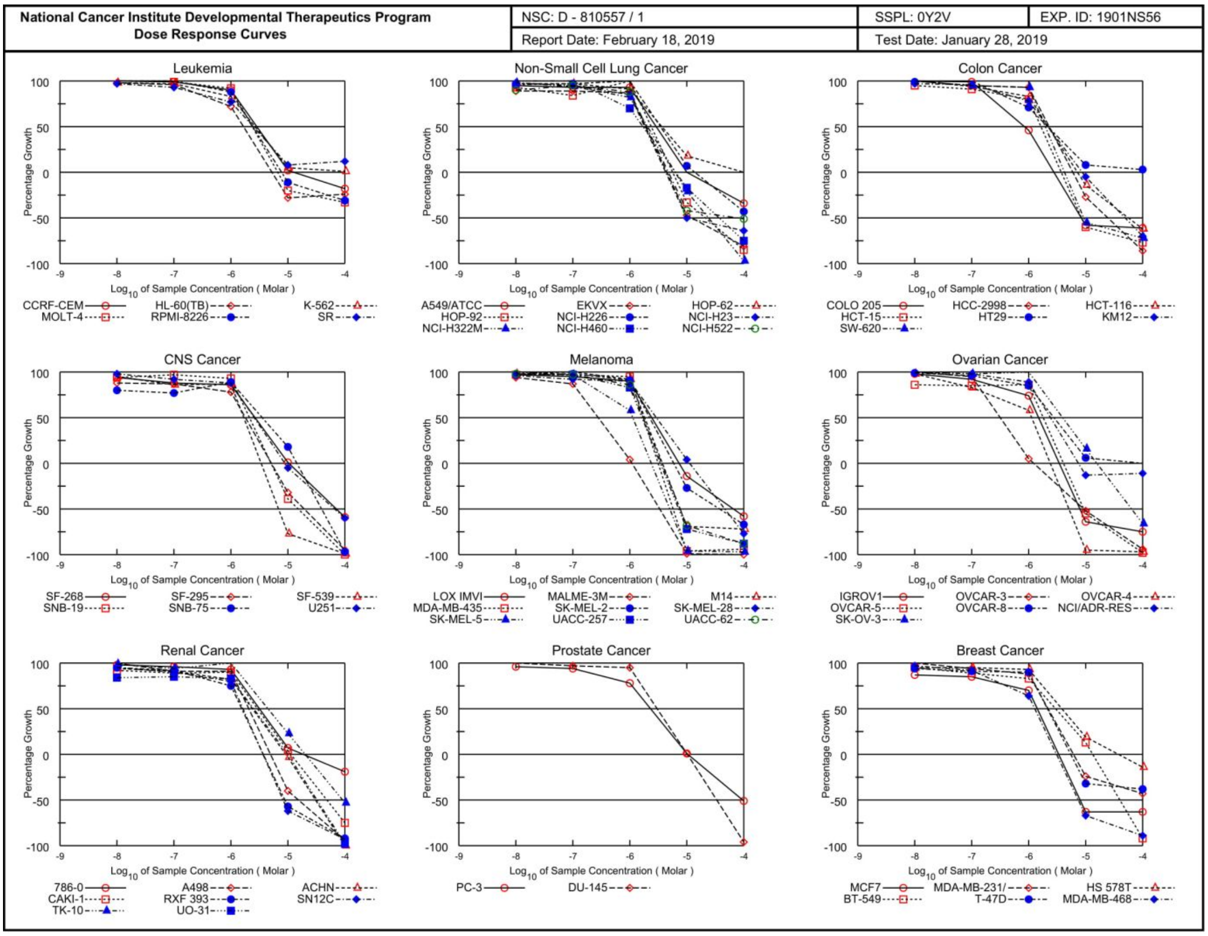

Figure S11 *NCI Five Dose response summary data for compound* ***6***

Table S4** NCI Five Dose raw data for compound **6

National Cancer Institute Developmental Therapeutics Program
In-Vitro Testing Results

NSC : D - 810557 / 1	Experiment ID : 1901NS56	Test Type : 08	Units : Molar
Report Date : February 18, 2019	Test Date : January 28, 2019	QNS :	MC :
COMI : RK 6.3.7	Stain Reagent : SRB Dual-Pass Related	SSPL : 0Y2V	

	Log10 Concentration															
	Time		Mean Optical Densities					Percent Growth								
Panel/Cell Line	Zero	Ctrl	-8.0	-7.0	-6.0	-5.0	-4.0	-8.0	-7.0	-6.0	-5.0	-4.0	GI50	TGI	LC50
**Leukemia**															
CCRF-CEM	0.580	2.988	2.996	2.960	2.751	0.633	0.476	100	99	90	2	-18	2.86E-6	1.28E-5	> 1.00E-4
HL-60(TB)	0.877	3.287	3.243	3.187	2.624	0.630	0.669	98	96	72	-28	-24	1.67E-6	5.25E-6	> 1.00E-4
K-562	0.214	2.718	2.680	2.640	2.303	0.341	0.248	98	97	83	5	1	2.67E-6	> 1.00E-4	> 1.00E-4
MOLT-4	0.550	2.961	2.993	2.942	2.757	0.441	0.370	101	99	92	-20	-33	2.36E-6	6.63E-6	> 1.00E-4
RPMI-8226	1.128	3.103	3.112	3.102	2.859	1.007	0.783	100	100	88	-11	-31	2.41E-6	7.78E-6	> 1.00E-4
SR	0.281	1.715	1.668	1.619	1.390	0.394	0.454	97	93	77	8	12	2.47E-6	> 1.00E-4	> 1.00E-4
**Non-Small Cell Lung Cancer**															
A549/ATCC	0.373	2.432	2.320	2.294	2.282	0.381	0.246	95	93	93	.	-34	2.90E-6	1.02E-5	> 1.00E-4
EKVX	0.679	1.996	1.855	1.845	1.820	0.358	0.127	89	89	87	-47	-81	1.88E-6	4.44E-6	1.20E-5
HOP-62	0.451	1.954	1.903	1.915	1.824	0.718	0.450	97	97	91	18	.	3.64E-6	9.72E-5	> 1.00E-4
HOP-92	1.205	1.968	1.913	1.846	2.046	0.813	0.176	93	84	110	-33	-85	2.64E-6	5.91E-6	2.14E-5
NCI-H226	1.465	3.116	3.049	3.065	3.126	1.573	0.831	96	97	101	7	-43	3.45E-6	1.35E-5	> 1.00E-4
NCI-H23	0.538	1.634	1.616	1.577	1.467	0.270	0.193	98	95	85	-50	-64	1.81E-6	4.26E-6	1.03E-5
NCI-H322M	0.694	2.032	2.003	1.947	1.789	0.551	0.019	98	94	82	-21	-97	2.04E-6	6.29E-6	2.42E-5
NCI-H460	0.284	2.989	3.070	3.109	2.176	0.236	0.071	103	104	70	-17	-75	1.70E-6	6.39E-6	3.71E-5
NCI-H522	0.911	2.816	2.632	2.716	2.583	0.526	0.445	90	95	88	-42	-51	1.95E-6	4.73E-6	7.41E-5
**Colon Cancer**															
COLO 205	0.443	2.044	2.051	2.035	1.179	0.184	0.174	100	99	46	-58	-61	8.39E-7	2.75E-6	8.30E-6
HCC-2998	0.652	2.493	2.549	2.406	2.356	0.476	0.095	103	95	93	-27	-86	2.27E-6	5.94E-6	2.47E-5
HCT-116	0.242	2.460	2.460	2.320	2.076	0.208	0.091	100	94	83	-14	-62	2.17E-6	7.13E-6	5.53E-5
HCT-15	0.355	2.648	2.543	2.449	2.189	0.141	0.083	95	91	80	-60	-77	1.64E-6	3.72E-6	8.45E-6
HT29	0.240	2.155	2.127	2.241	1.603	0.385	0.297	99	104	71	8	3	2.15E-6	> 1.00E-4	> 1.00E-4
KM12	0.467	2.917	2.843	2.827	2.388	0.443	0.140	97	96	78	-5	-70	2.19E-6	8.68E-6	4.90E-5
SW-620	0.200	1.460	1.488	1.393	1.368	0.091	0.057	102	95	93	-55	-72	1.95E-6	4.26E-6	9.32E-6
**CNS Cancer**															
SF-268	0.759	2.525	2.425	2.314	2.274	0.773	0.312	94	88	86	1	-59	2.64E-6	1.03E-5	7.08E-5
SF-295	0.484	1.786	1.634	1.614	1.500	0.327	0.026	88	87	78	-32	-95	1.79E-6	5.09E-6	1.91E-5
SF-539	0.884	2.838	2.740	2.556	2.581	0.205	0.005	95	86	87	-77	-99	1.68E-6	3.39E-6	6.86E-6
SNB-19	0.749	2.710	2.596	2.644	2.568	0.454	-0.006	94	97	93	-39	-100	2.11E-6	5.03E-6	1.49E-5
SNB-75	0.890	1.782	1.605	1.575	1.680	1.048	0.029	80	77	89	18	-97	3.50E-6	1.43E-5	3.91E-5
U251	0.256	1.770	1.745	1.653	1.593	0.243	0.102	98	92	88	-5	-60	2.57E-6	8.82E-6	6.50E-5
**Melanoma**															
LOX IMVI	0.406	3.089	3.007	2.962	2.811	0.349	0.172	97	95	90	-14	-58	2.41E-6	7.30E-6	6.67E-5
MALME-3M	0.679	1.790	1.722	1.646	0.727	0.006	0.001	94	87	4	-99	-100	2.80E-7	1.10E-6	3.35E-6
M14	0.388	1.815	1.788	1.890	1.695	0.119	0.110	98	105	92	-69	-72	1.81E-6	3.71E-6	7.58E-6
MDA-MB-435	0.443	2.290	2.228	2.230	2.202	0.019	0.028	97	97	95	-96	-94	1.73E-6	3.15E-6	5.76E-6
SK-MEL-2	1.315	3.049	3.005	3.014	2.898	0.956	0.439	97	98	91	-27	-67	2.23E-6	5.88E-6	3.78E-5
SK-MEL-28	0.607	2.304	2.232	2.170	2.142	0.681	0.139	96	92	90	4	-77	2.95E-6	1.13E-5	4.65E-5
SK-MEL-5	0.752	3.224	3.241	3.148	2.188	0.033	0.022	101	97	58	-96	-97	1.13E-6	2.39E-6	5.05E-6
UACC-257	1.097	2.761	2.788	2.705	2.475	0.305	0.135	102	97	83	-72	-88	1.63E-6	3.42E-6	7.19E-6
UACC-62	1.057	3.153	3.108	3.084	2.867	0.336	0.131	98	97	86	-68	-88	1.72E-6	3.62E-6	7.62E-6
**Ovarian Cancer**															
IGROV1	0.358	1.959	1.920	1.825	1.544	0.130	0.090	98	92	74	-64	-75	1.50E-6	3.45E-6	7.96E-6
OVCAR-3	0.547	2.074	2.106	1.999	0.617	0.261	0.034	102	95	5	-52	-94	3.15E-7	1.20E-6	9.08E-6
OVCAR-4	0.555	1.349	1.333	1.213	1.012	0.026	0.015	98	83	58	-95	-97	1.12E-6	2.38E-6	5.05E-6
OVCAR-5	0.528	1.766	1.596	1.581	1.596	0.235	0.013	86	85	86	-55	-98	1.80E-6	4.06E-6	9.15E-6
OVCAR-8	0.418	2.355	2.326	2.281	2.065	0.539	0.421	99	96	85	6	.	2.78E-6	> 1.00E-4	> 1.00E-4
NCI/ADR-RES	0.621	2.320	2.344	2.293	2.135	0.542	0.551	101	98	89	-13	-11	2.42E-6	7.49E-6	> 1.00E-4
SK-OV-3	0.927	2.350	2.366	2.325	2.400	1.160	0.319	101	98	103	16	-66	4.11E-6	1.58E-5	6.45E-5
**Renal Cancer**															
786-0	0.623	2.741	2.698	2.648	2.589	0.771	0.508	98	96	93	7	-19	3.15E-6	1.88E-5	> 1.00E-4
A498	1.828	2.817	2.868	2.732	2.727	1.103	0.115	105	91	91	-40	-94	2.06E-6	4.97E-6	1.55E-5
ACHN	0.320	1.687	1.606	1.562	1.436	0.309	0.001	94	91	82	-3	-100	2.35E-6	9.11E-6	3.04E-5
CAKI-1	0.613	2.528	2.366	2.315	2.328	0.690	0.156	92	89	90	4	-75	2.90E-6	1.12E-5	4.86E-5
RXF 393	1.197	1.650	1.628	1.612	1.538	0.511	0.098	95	92	75	-57	-92	1.55E-6	3.70E-6	8.81E-6
SN12C	0.664	2.684	2.585	2.486	2.273	0.252	0.045	95	90	80	-62	-93	1.62E-6	3.65E-6	8.21E-6
TK-10	0.733	2.329	2.307	2.234	2.381	1.103	0.342	99	94	103	23	-53	4.62E-6	2.01E-5	9.03E-5
UO-31	0.630	2.026	1.802	1.816	1.784	0.631	0.013	84	85	83	.	-98	2.48E-6	1.00E-5	3.24E-5
**Prostate Cancer**															
PC-3	0.511	2.297	2.231	2.193	1.905	0.526	0.250	96	94	78	1	-51	2.31E-6	1.04E-5	9.53E-5
DU-145	0.346	1.708	1.763	1.670	1.645	0.358	0.016	104	97	95	1	-96	3.02E-6	1.02E-5	3.37E-5
**Breast Cancer**															
MCF7	0.309	1.881	1.683	1.652	1.411	0.113	0.115	87	85	70	-63	-63	1.41E-6	3.35E-6	7.93E-6
MDA-MB-231/ATCC	0.656	1.643	1.671	1.588	1.528	0.501	0.376	103	94	88	-24	-43	2.20E-6	6.14E-6	> 1.00E-4
HS 578T	0.915	1.990	1.938	1.939	1.920	1.116	0.783	95	95	93	19	-14	3.81E-6	3.66E-5	> 1.00E-4
BT-549	1.174	2.627	2.557	2.470	2.377	1.365	0.095	95	89	83	13	-92	2.96E-6	1.33E-5	3.99E-5
T-47D	1.075	2.755	2.652	2.612	2.579	0.734	0.665	94	91	90	-32	-38	2.12E-6	5.47E-6	> 1.00E-4
MDA-MB-468	0.915	1.548	1.532	1.495	1.319	0.298	0.098	97	92	64	-67	-89	1.27E-6	3.06E-6	7.36E-6

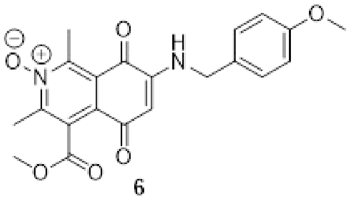

6

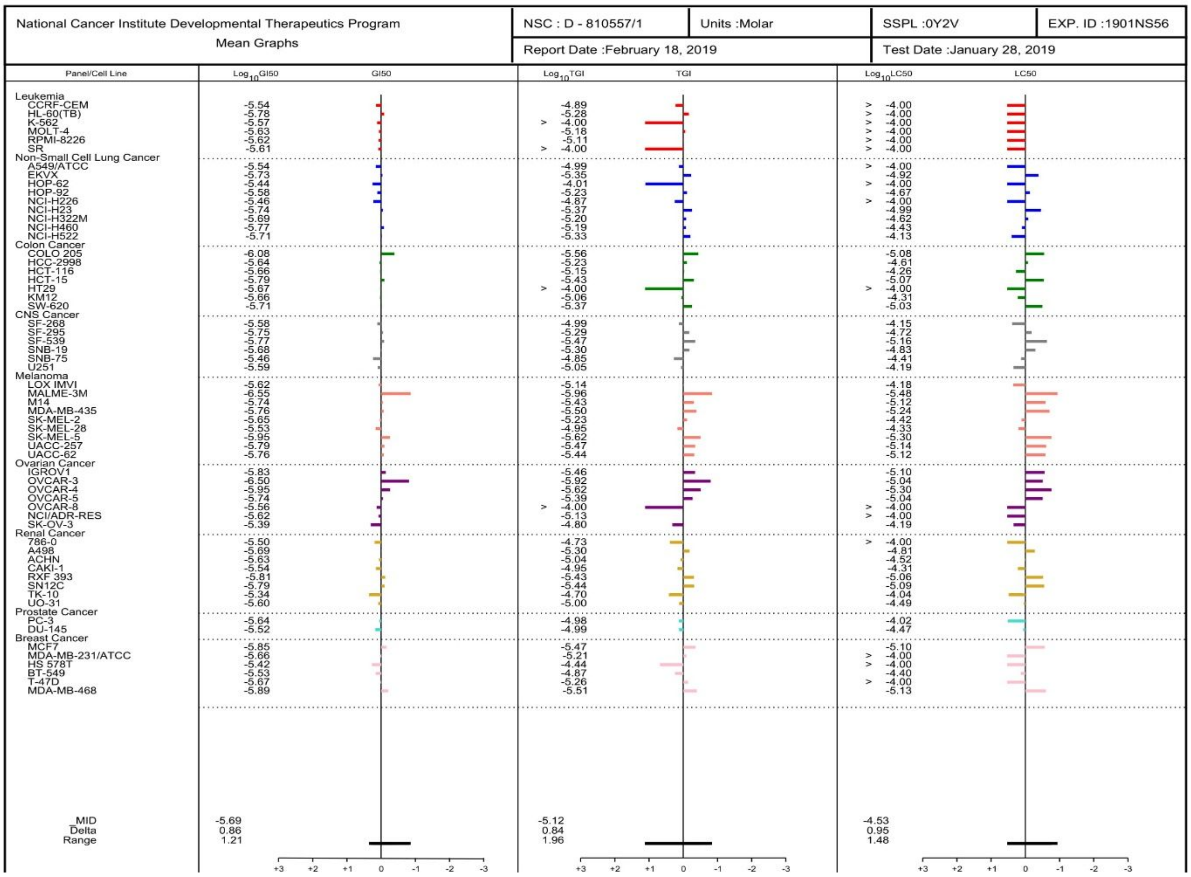

Mean of GI_{50} across 60 cell lines for compound **6** as Log_{10} Concentration (SD): -5.691 (±0.21)

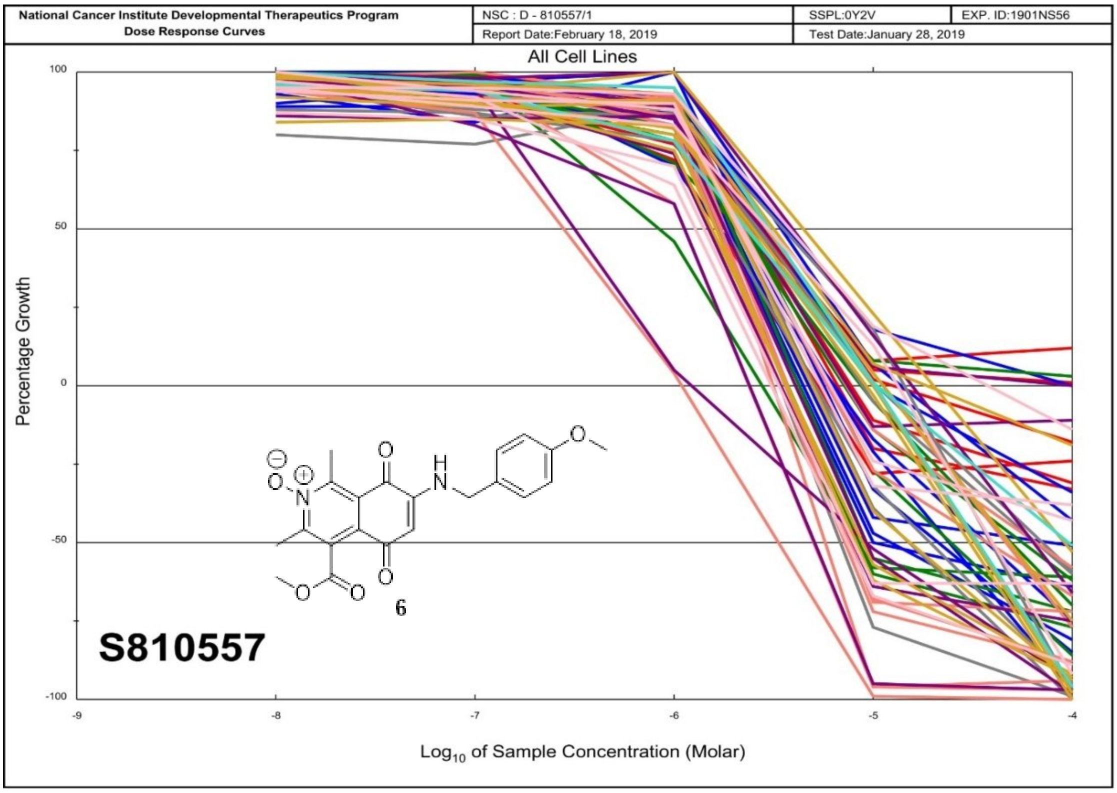

Figure S12 *NCI Five Dose data cell line comparison summary for compound* ***6***

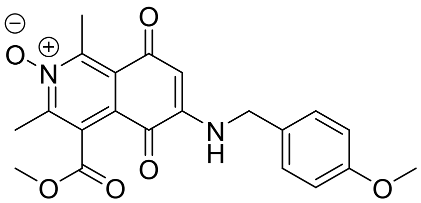

7

Developmental Therapeutics Program — **One Dose Mean Graph**

NSC: D-820593 / 1 | **Conc:** 1.00E-5 Molar | **Test Date:** Nov 18, 2019

Experiment ID: 1911OS93 | **Report Date:** Dec 19, 2019

Panel/Cell Line	Growth Percent
**Leukemia**	
CCRF-CEM	3.40
HL-60(TB)	-30.53
K-562	0.82
MOLT-4	-35.50
RPMI-8226	-16.48
SR	-0.64
**Non-Small Cell Lung Cancer**	
A549/ATCC	-18.42
EKVX	-57.64
HOP-62	3.79
HOP-92	-35.26
NCI-H226	-8.68
NCI-H23	-38.78
NCI-H322M	-6.26
NCI-H460	-3.91
NCI-H522	-19.52
**Colon Cancer**	
COLO 205	-54.01
HCT-116	-11.60
HCT-15	-43.94
HT29	3.50
KM12	-3.93
SW-620	0.25
**CNS Cancer**	
SF-268	-22.37
SF-295	-62.47
SF-539	-80.66
SNB-19	-73.80
SNB-75	-96.97
U251	-45.75
**Melanoma**	
LOX IMVI	-12.01
MALME-3M	-97.36
M14	-84.62
MDA-MB-435	-91.59
SK-MEL-2	-22.21
SK-MEL-28	-6.37
SK-MEL-5	-96.57
UACC-257	-77.00
UACC-62	-75.08
**Ovarian Cancer**	
IGROV1	-61.46
OVCAR-3	-40.49
OVCAR-4	-98.48
OVCAR-5	-54.60
OVCAR-8	1.17
NCI/ADR-RES	-7.81
SK-OV-3	8.27
**Renal Cancer**	
786-0	3.11
A498	-15.40
ACHN	-11.51
CAKI-1	0.87
RXF 393	-32.69
SN12C	-62.96
TK-10	28.36
UO-31	-9.55
**Prostate Cancer**	
PC-3	-6.90
DU-145	-83.66
**Breast Cancer**	
MCF7	-55.06
MDA-MB-231/ATCC	-6.30
HS 578T	-10.19
BT-549	-88.75
T-47D	-30.98
MDA-MB-468	-61.05
Mean	-34.14
Delta	64.34
Range	126.84

Mean Growth Percent - Growth Percent

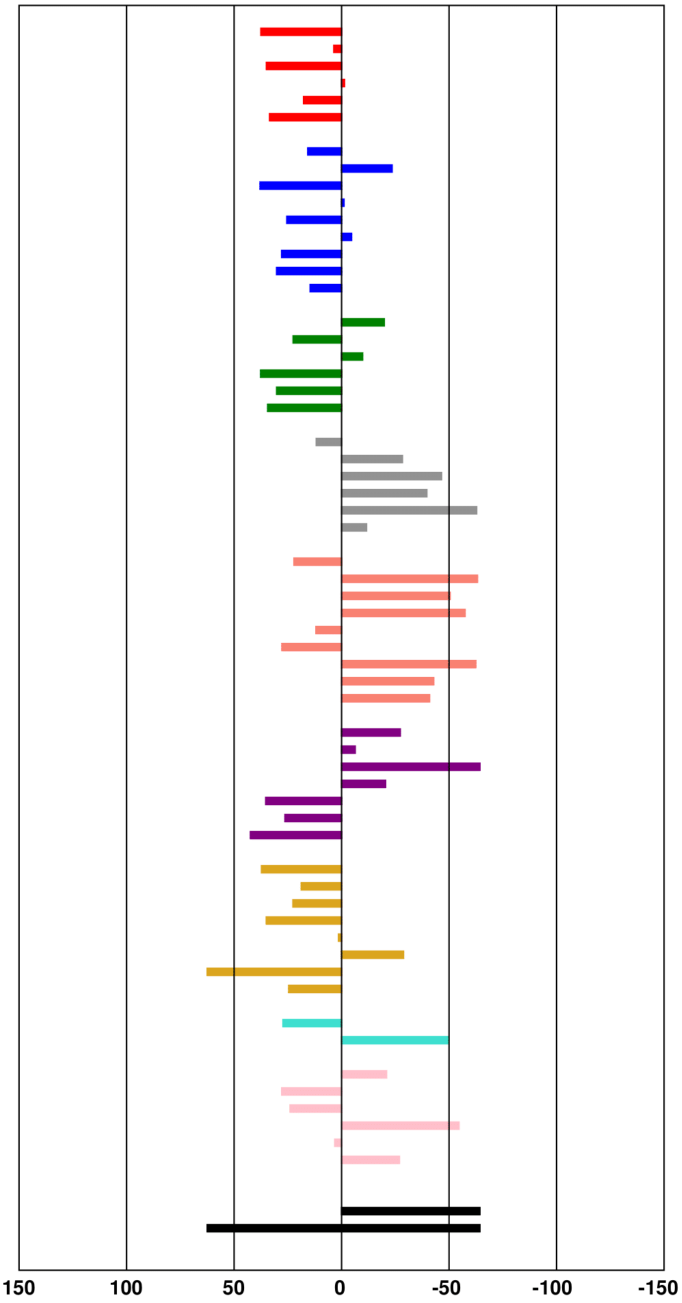

Figure S13 *NCI One Dose (10 µM) data for compound* ***7***

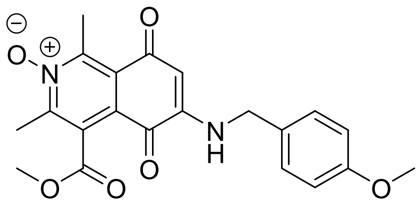

7

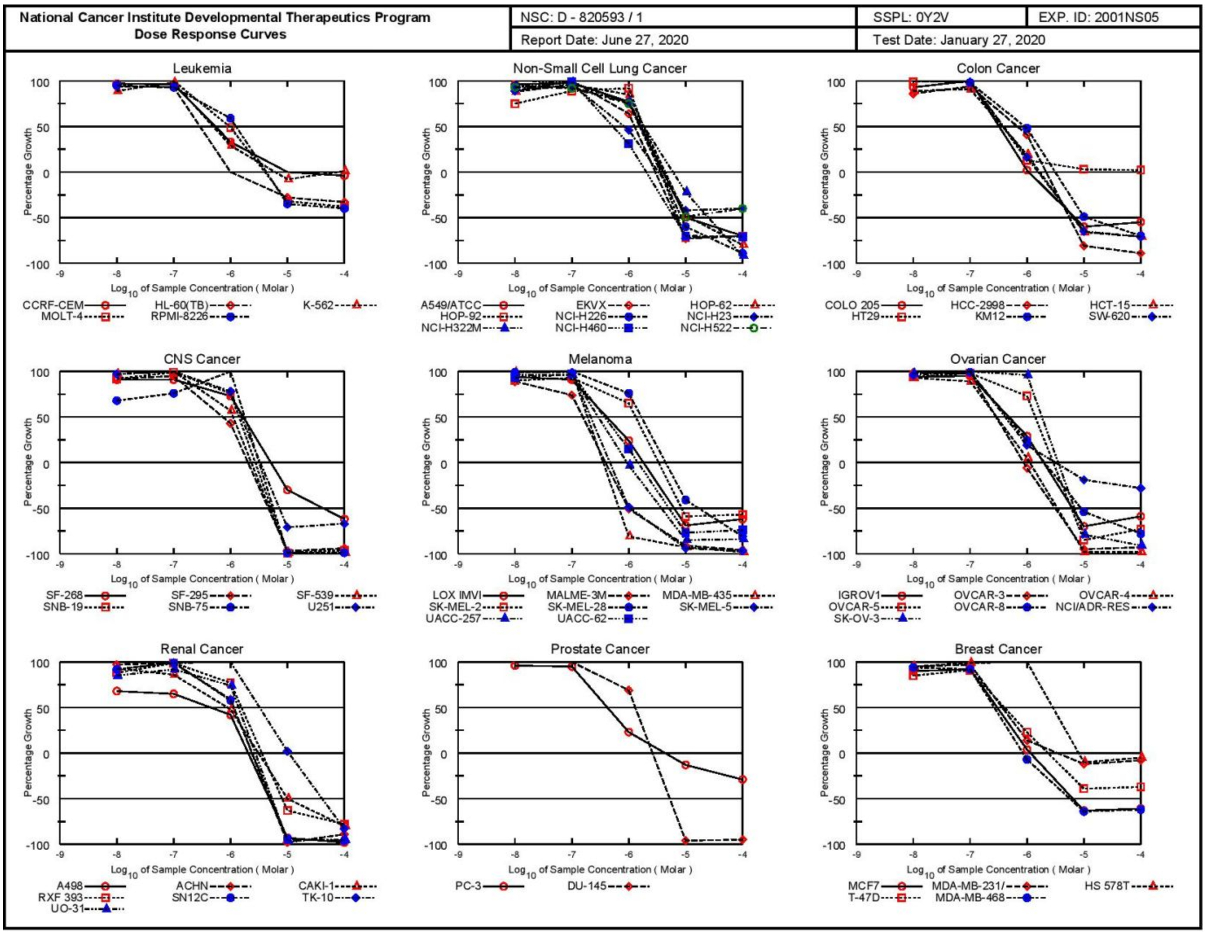

Figure S14 *NCI Five Dose response summary data for compound **7***

Table S5 *NCI Five Dose raw data for compound **7***

National Cancer Institute Developmental Therapeutics Program
In-Vitro Testing Results

NSC : D - 820593 / 1	Experiment ID : 2001NS05	Test Type : 08	Units : Molar
Report Date : June 27, 2020	Test Date : January 27, 2020	QNS :	MC :
COMI : RK 6.3.7.1	Stain Reagent : SRB Dual-Pass Related	SSPL : 0Y2V	

Log10 Concentration

Panel/Cell Line	Time Zero	Ctrl	Mean Optical Densities					Percent Growth					GI50	TGI	LC50
			-8.0	-7.0	-6.0	-5.0	-4.0	-8.0	-7.0	-6.0	-5.0	-4.0			
**Leukemia**															
CCRF-CEM	0.435	2.132	2.081	2.064	0.992	0.443	0.418	97	96	33	.	-4	5.35E-7	1.27E-5	> 1.00E-4
HL-60(TB)	0.661	2.924	2.794	2.759	0.656	0.478	0.443	94	93	.	-28	-33	2.86E-7	9.80E-7	> 1.00E-4
K-562	0.196	1.728	1.563	1.696	0.634	0.181	0.213	89	98	29	-8	1	4.91E-7	.	> 1.00E-4
MOLT-4	0.835	3.198	3.228	3.208	1.983	0.566	0.520	101	100	49	-32	-38	9.38E-7	3.99E-6	> 1.00E-4
RPMI-8226	0.857	2.320	2.247	2.223	1.725	0.557	0.511	95	93	59	-35	-40	1.25E-6	4.25E-6	> 1.00E-4
**Non-Small Cell Lung Cancer**															
A549/ATCC	0.949	3.053	2.968	3.008	2.575	0.478	0.288	96	98	77	-50	-70	1.64E-6	4.06E-6	1.04E-5
EKVX	0.899	2.124	1.992	2.147	1.687	0.241	0.273	89	102	64	-73	-70	1.27E-6	2.94E-6	6.78E-6
HOP-62	0.823	2.641	2.427	2.565	2.376	0.428	0.162	88	96	85	-48	-80	1.84E-6	4.36E-6	1.15E-5
HOP-92	1.421	2.105	1.935	2.027	2.048	0.392	0.422	75	89	92	-72	-70	1.79E-6	3.62E-6	7.30E-6
NCI-H226	0.750	1.748	1.686	1.677	1.512	0.300	0.080	94	93	76	-60	-89	1.56E-6	3.63E-6	8.45E-6
NCI-H23	0.833	2.325	2.169	2.262	1.526	0.482	0.502	89	96	46	-42	-40	8.46E-7	3.34E-6	> 1.00E-4
NCI-H322M	0.923	2.462	2.351	2.437	2.100	0.724	0.078	93	98	76	-22	-92	1.86E-6	6.03E-6	2.55E-5
NCI-H460	0.258	2.757	2.786	2.742	1.032	0.078	0.075	101	99	31	-70	-71	5.27E-7	2.03E-6	6.34E-6
NCI-H522	1.079	2.893	2.763	2.752	2.449	0.554	0.652	93	92	75	-49	-40	1.60E-6	4.05E-6	> 1.00E-4
**Colon Cancer**															
COLO 205	0.655	2.924	2.775	2.934	0.700	0.260	0.294	93	100	2	-60	-55	3.25E-7	1.08E-6	6.82E-6
HCC-2998	0.527	2.184	1.959	2.086	1.213	0.100	0.058	86	94	41	-81	-89	6.87E-7	2.18E-6	5.58E-6
HCT-15	0.373	2.552	2.311	2.352	0.778	0.126	0.108	89	91	19	-66	-71	3.67E-7	1.66E-6	6.44E-6
HT29	0.439	2.830	2.809	3.007	0.742	0.510	0.487	99	107	13	3	2	4.04E-7	> 1.00E-4	> 1.00E-4
KM12	0.853	3.338	3.331	3.300	2.037	0.436	0.260	100	98	48	-49	-70	8.98E-7	3.11E-6	1.13E-5
SW-620	0.336	2.273	2.269	2.262	0.645	0.118	0.098	100	99	16	-65	-71	3.91E-7	1.57E-6	6.54E-6
**CNS Cancer**															
SF-268	1.051	2.863	2.706	2.695	2.382	0.741	0.404	91	91	73	-30	-62	1.69E-6	5.17E-6	4.35E-5
SF-295	0.766	3.135	2.953	3.027	1.792	0.024	0.044	92	95	43	-97	-94	7.44E-7	2.04E-6	4.63E-6
SF-539	0.914	2.771	2.717	2.799	1.979	0.009	0.012	97	102	57	-99	-99	1.11E-6	2.33E-6	4.86E-6
SNB-19	0.621	2.193	2.066	2.176	1.819	0.004	0.023	92	99	76	-99	-96	1.41E-6	2.72E-6	5.23E-6
SNB-75	1.678	2.589	2.297	2.373	2.604	0.021	0.024	68	76	102	-99	-99	1.81E-6	3.21E-6	5.71E-6
U251	0.659	2.751	2.687	2.763	2.287	0.193	0.217	97	101	78	-71	-67	1.54E-6	3.34E-6	7.25E-6
**Melanoma**															
LOX IMVI	0.438	3.078	2.921	2.836	1.072	0.135	0.167	94	91	24	-69	-62	4.08E-7	1.81E-6	6.23E-6
MALME-3M	0.917	2.239	2.094	1.893	0.448	0.085	0.041	89	74	-51	-91	-96	1.55E-7	3.89E-7	9.78E-7
MDA-MB-435	0.874	3.316	3.302	3.324	0.168	0.057	0.017	99	100	-81	-93	-98	1.90E-7	3.58E-7	6.76E-7
SK-MEL-2	1.256	2.848	2.689	2.741	2.283	0.512	0.534	90	93	65	-59	-57	1.31E-6	3.32E-6	8.42E-6
SK-MEL-28	0.831	2.589	2.563	2.708	2.167	0.492	0.159	99	107	76	-41	-81	1.67E-6	4.47E-6	1.69E-5
SK-MEL-5	0.771	3.301	3.270	3.269	0.392	0.046	0.021	99	99	-49	-94	-97	2.13E-7	4.65E-7	1.04E-6
UACC-257	1.224	2.794	2.726	2.729	1.187	0.188	0.202	96	96	-3	-85	-84	2.91E-7	9.31E-7	3.76E-6
UACC-62	0.988	2.942	2.811	2.880	1.282	0.230	0.260	93	97	15	-77	-74	3.74E-7	1.46E-6	5.11E-6
**Ovarian Cancer**															
IGROV1	0.506	2.261	2.150	2.177	1.017	0.150	0.210	94	95	29	-70	-59	4.83E-7	1.96E-6	6.24E-6
OVCAR-3	0.877	2.543	2.519	2.491	0.820	0.048	0.063	99	97	-6	-95	-93	2.84E-7	8.65E-7	3.12E-6
OVCAR-4	1.006	2.210	2.127	2.074	1.069	0.019	0.017	93	89	5	-98	-98	2.91E-7	1.12E-6	3.42E-6
OVCAR-5	0.431	1.473	1.405	1.450	1.196	0.063	0.116	94	98	73	-85	-73	1.40E-6	2.90E-6	5.99E-6
OVCAR-8	0.600	2.615	2.619	2.595	1.088	0.279	0.131	100	99	24	-54	-78	4.52E-7	2.05E-6	9.02E-6
NCI/ADR-RES	0.576	2.148	2.089	2.164	0.875	0.466	0.417	96	101	19	-19	-28	4.19E-7	3.15E-6	> 1.00E-4
SK-OV-3	0.951	2.615	2.581	2.689	2.551	0.202	0.082	98	104	96	-79	-91	1.84E-6	3.55E-6	6.85E-6
**Renal Cancer**															
A498	2.141	2.900	2.657	2.632	2.462	0.156	0.039	68	65	42	-93	-98	4.52E-7	2.06E-6	4.83E-6
ACHN	0.327	1.669	1.626	1.658	1.117	0.006	0.035	97	99	59	-98	-89	1.14E-6	2.37E-6	4.93E-6
CAKI-1	1.085	2.989	2.855	2.717	1.996	0.544	0.218	93	86	48	-50	-80	8.76E-7	3.09E-6	1.01E-5
RXF 393	1.369	2.292	2.186	2.282	2.083	0.503	0.307	88	99	77	-63	-78	1.56E-6	3.55E-6	8.04E-6
SN12C	0.489	2.263	2.125	2.238	1.522	0.029	0.019	92	99	58	-94	-96	1.13E-6	2.41E-6	5.14E-6
TK-10	0.899	2.207	2.104	2.194	2.268	0.922	0.149	92	99	105	2	-83	3.40E-6	1.05E-5	4.05E-5
UO-31	0.612	1.843	1.658	1.739	1.518	0.024	0.034	85	92	74	-96	-95	1.38E-6	2.72E-6	5.35E-6
**Prostate Cancer**															
PC-3	0.753	2.605	2.523	2.514	1.181	0.656	0.531	96	95	23	-13	-29	4.23E-7	4.39E-6	> 1.00E-4
DU-145	0.418	1.831	1.881	1.965	1.395	0.017	0.020	104	109	69	-96	-95	1.31E-6	2.62E-6	5.26E-6
**Breast Cancer**															
MCF7	0.732	2.733	2.541	2.581	0.803	0.274	0.284	90	92	4	-63	-61	3.00E-7	1.13E-6	6.44E-6
MDA-MB-231/ATCC	0.607	1.650	1.594	1.623	0.757	0.536	0.557	95	97	14	-12	-8	3.72E-7	3.55E-6	> 1.00E-4
HS 578T	1.654	2.792	2.735	2.784	2.805	1.496	1.579	95	99	101	-10	-5	2.90E-6	8.19E-6	> 1.00E-4
T-47D	0.914	2.501	2.260	2.366	1.276	0.557	0.573	85	91	23	-39	-37	4.02E-7	2.34E-6	> 1.00E-4
MDA-MB-468	0.960	2.172	2.095	2.081	0.894	0.348	0.362	94	92	-7	-64	-62	2.68E-7	8.53E-7	5.72E-6

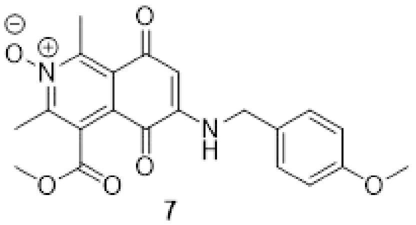

7

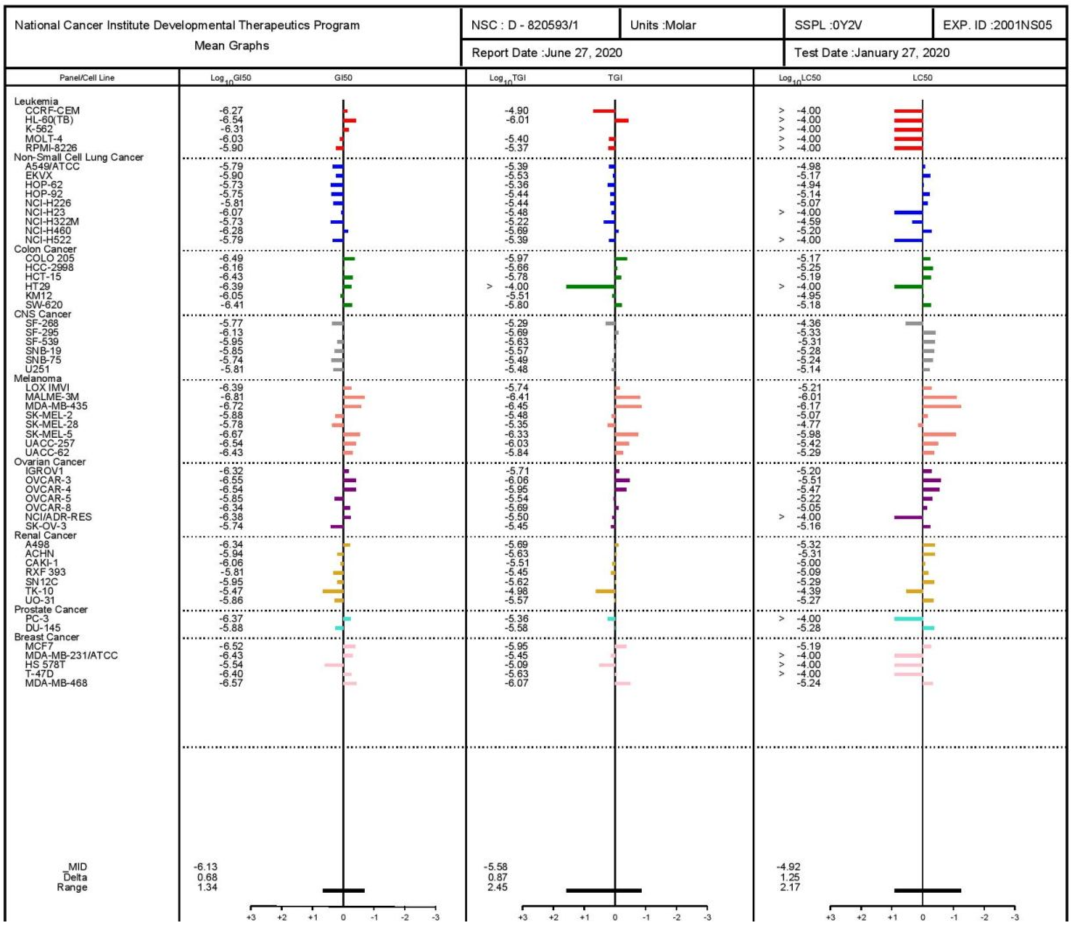

Mean of GI_{50} across 58 cell lines for compound **7** as Log_{10} Concentration (SD): -5.816 (±0.41)

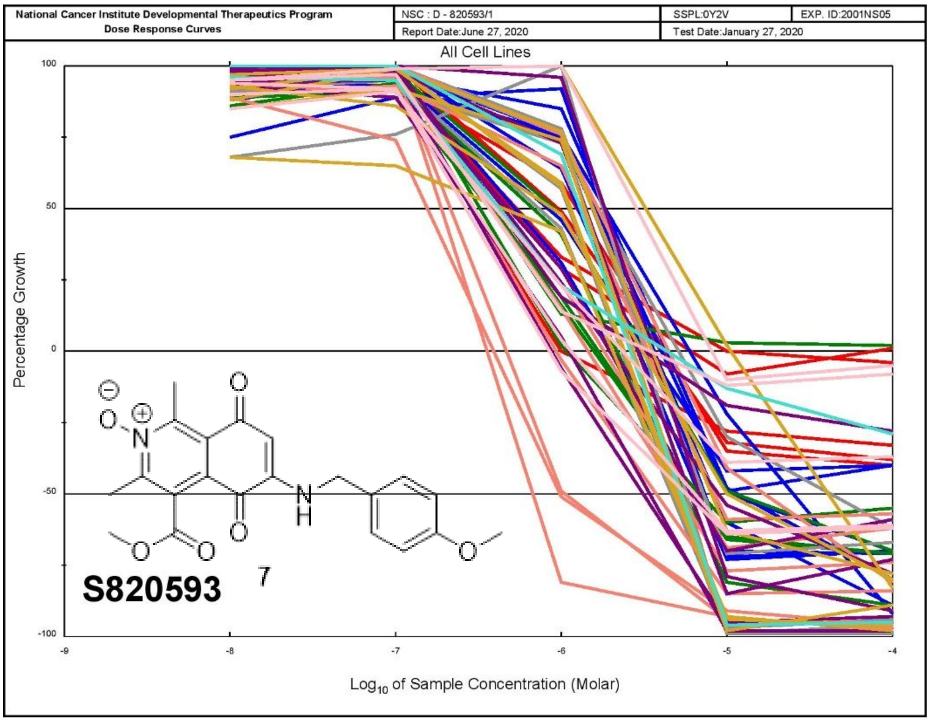

Figure S15 *NCI Five Dose data cell line comparison summary for compound* ***7***

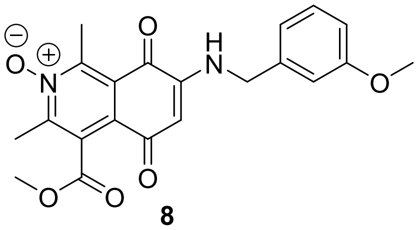

8

Developmental Therapeutics Program One Dose Mean Graph	NSC: D-810558 / 1	Conc: 1.00E-5 Molar	Test Date: Dec 03, 2018
	Experiment ID: 1812OS40		Report Date: Dec 20, 2018

Panel/Cell Line	Growth Percent
**Leukemia**	
CCRF-CEM	16.26
HL-60(TB)	-52.23
K-562	13.66
MOLT-4	3.80
RPMI-8226	33.07
SR	17.56
**Non-Small Cell Lung Cancer**	
A549/ATCC	12.91
EKVX	57.23
HOP-62	53.18
HOP-92	54.52
NCI-H226	83.21
NCI-H23	25.37
NCI-H322M	22.63
NCI-H460	14.60
NCI-H522	10.75
**Colon Cancer**	
COLO 205	-63.36
HCC-2998	22.33
HCT-116	1.61
HCT-15	11.92
HT29	11.44
KM12	21.97
SW-620	7.85
**CNS Cancer**	
SF-268	36.79
SF-295	69.92
SF-539	11.45
SNB-19	19.23
SNB-75	132.21
U251	-1.83
**Melanoma**	
LOX IMVI	22.02
MALME-3M	-89.88
M14	12.88
MDA-MB-435	-70.63
SK-MEL-2	64.28
SK-MEL-28	86.64
SK-MEL-5	-54.84
UACC-257	-25.55
UACC-62	-13.25
**Ovarian Cancer**	
IGROV1	12.34
OVCAR-3	-12.10
OVCAR-4	-28.61
OVCAR-5	67.57
OVCAR-8	-4.96
NCI/ADR-RES	8.50
SK-OV-3	89.56
**Renal Cancer**	
786-0	54.25
A498	107.91
ACHN	47.76
CAKI-1	55.69
RXF 393	38.72
SN12C	2.90
TK-10	103.63
UO-31	76.79
**Prostate Cancer**	
PC-3	6.62
DU-145	54.83
**Breast Cancer**	
MCF7	0.25
MDA-MB-231/ATCC	5.97
HS 578T	82.72
BT-549	57.51
T-47D	11.52
MDA-MB-468	-21.27
Mean	23.30
Delta	113.18
Range	222.09

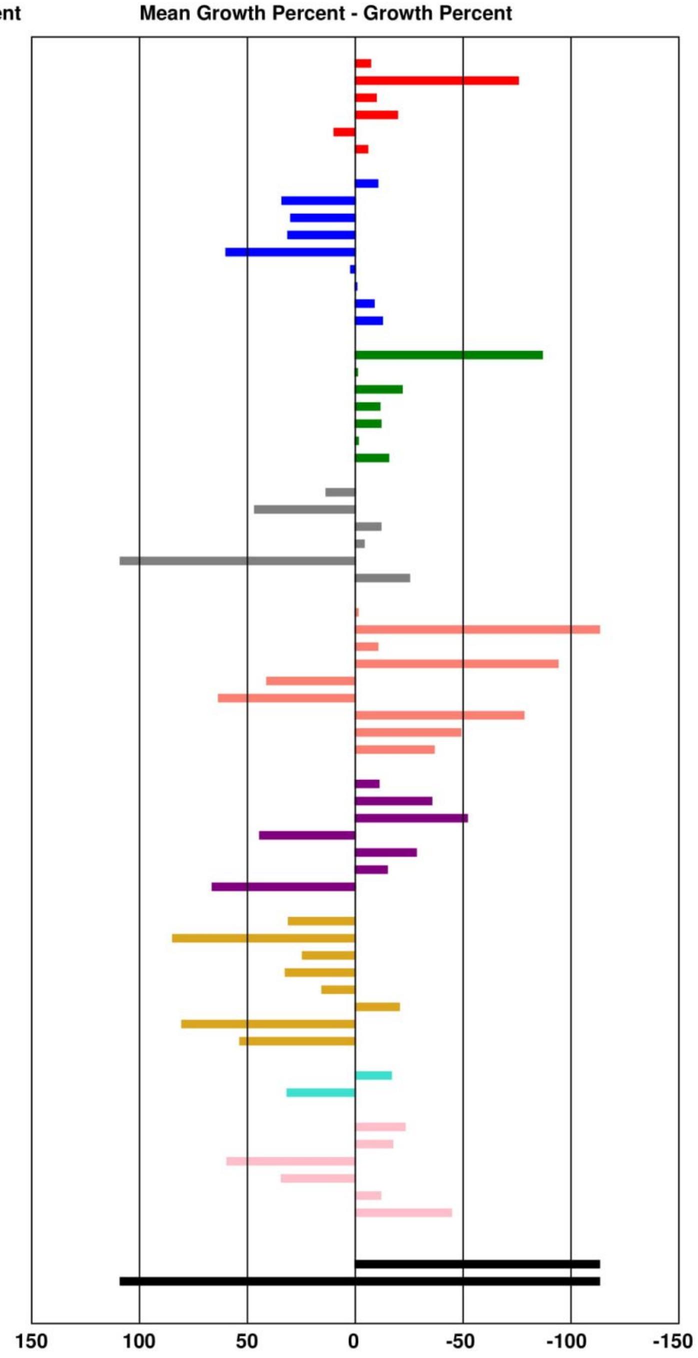

Figure S16 *NCI One Dose (10 µM) data for compound* ***8***

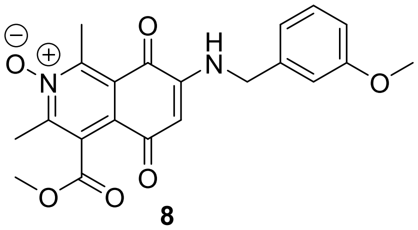

8

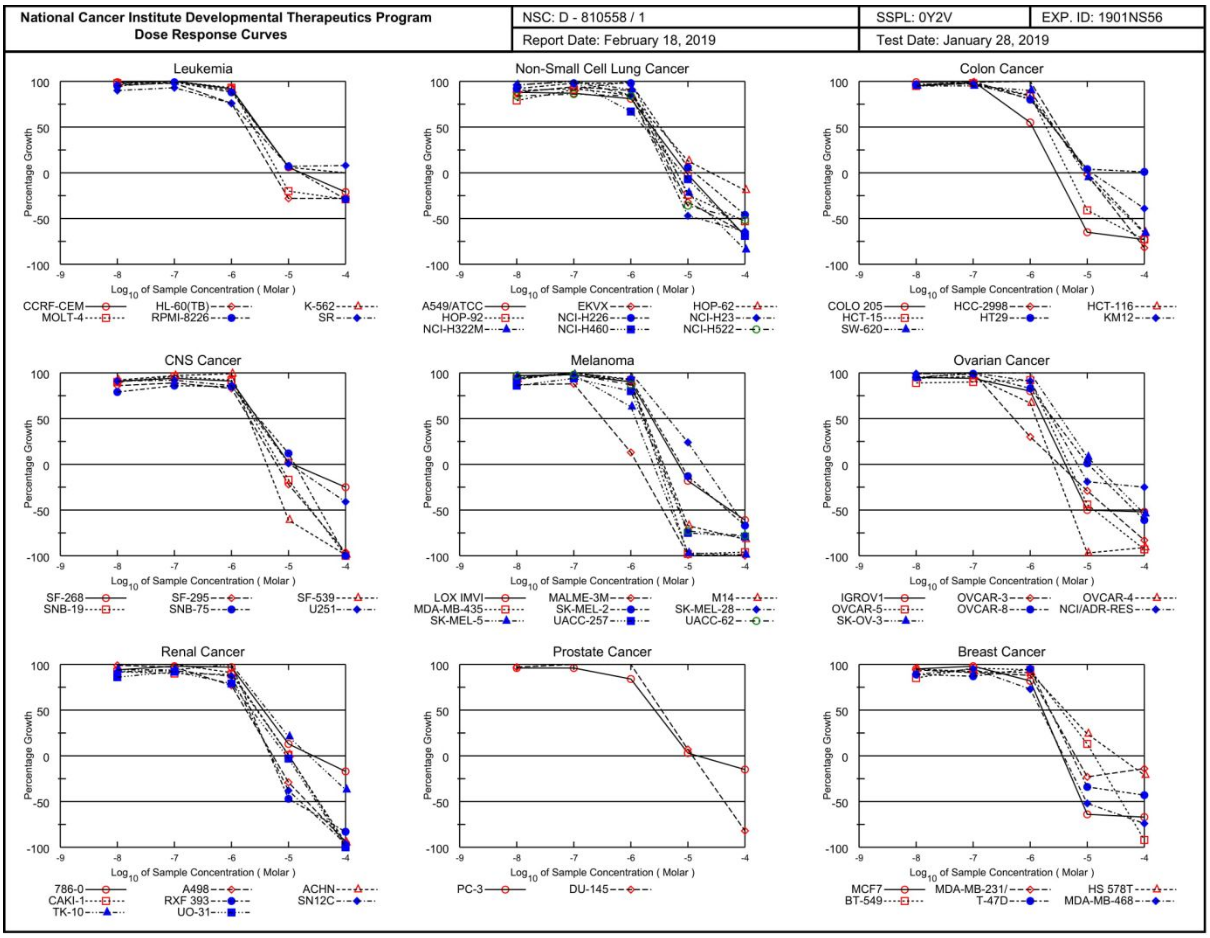

Figure S17 *NCI Five Dose response summary data for compound **8***

Table S6** NCI Five Dose raw data for compound **8

National Cancer Institute Developmental Therapeutics Program
In-Vitro Testing Results

NSC : D - 810558 / 1	Experiment ID : 1901NS56	Test Type : 08	Units : Molar
Report Date : February 18, 2019	Test Date : January 28, 2019	QNS :	MC :
COMI : RK 6.3.8	Stain Reagent : SRB Dual-Pass Related	SSPL : 0Y2V	

			Log10 Concentration														
	Time		Mean Optical Densities					Percent Growth									
Panel/Cell Line	Zero	Ctrl	-8.0	-7.0	-6.0	-5.0	-4.0	-8.0	-7.0	-6.0	-5.0	-4.0		GI50		TGI	LC50
Leukemia																	
CCRF-CEM	0.580	3.025	3.010	3.013	2.827	0.735	0.459	99	100	92	6	-21		3.09E-6		1.71E-5	> 1.00E-4
HL-60(TB)	0.877	3.263	3.177	3.223	2.689	0.633	0.630	96	98	76	-28	-28		1.78E-6		5.39E-6	> 1.00E-4
K-562	0.214	2.584	2.516	2.585	2.339	0.359	0.219	97	100	90	6	.		2.98E-6		> 1.00E-4	> 1.00E-4
MOLT-4	0.550	2.896	2.857	2.925	2.721	0.442	0.389	98	101	93	-20	-29		2.39E-6		6.67E-6	> 1.00E-4
RPMI-8226	1.128	3.118	3.015	3.101	2.870	1.272	0.804	95	99	88	7	-29		2.93E-6		1.59E-5	> 1.00E-4
SR	0.281	1.647	1.509	1.551	1.317	0.371	0.397	90	93	76	7	8		2.36E-6		> 1.00E-4	> 1.00E-4
Non-Small Cell Lung Cancer																	
A549/ATCC	0.373	2.457	2.200	2.193	2.061	0.364	0.117	88	87	81	-2	-69		2.35E-6		9.36E-6	5.21E-5
EKVX	0.679	2.007	1.852	1.931	1.869	0.461	0.232	88	94	90	-32	-66		2.11E-6		5.44E-6	3.38E-5
HOP-62	0.451	2.034	1.872	1.908	1.799	0.657	0.365	90	92	85	13	-19		3.07E-6		2.53E-5	> 1.00E-4
HOP-92	1.205	1.972	1.807	1.906	2.033	0.915	0.564	79	91	108	-24	-53		2.75E-6		6.57E-6	7.77E-5
NCI-H226	1.465	3.101	2.967	3.074	3.068	1.562	0.796	92	98	98	6	-46		3.32E-6		1.30E-5	> 1.00E-4
NCI-H23	0.538	1.664	1.616	1.664	1.494	0.286	0.191	96	100	85	-47	-64		1.84E-6		4.41E-6	1.51E-5
NCI-H322M	0.694	2.018	1.960	2.047	1.898	0.539	0.111	96	102	91	-22	-84		2.30E-6		6.34E-6	2.80E-5
NCI-H460	0.284	2.974	3.116	3.025	2.094	0.264	0.089	105	102	67	-7	-69		1.71E-6		8.00E-6	4.97E-5
NCI-H522	0.911	2.836	2.529	2.569	2.528	0.584	0.436	84	86	84	-36	-52		1.92E-6		5.02E-6	7.33E-5
Colon Cancer																	
COLO 205	0.443	2.075	2.055	2.080	1.340	0.153	0.119	99	100	55	-65	-73		1.10E-6		2.86E-6	7.44E-6
HCC-2998	0.652	2.525	2.431	2.498	2.531	0.642	0.116	95	99	100	-2	-82		3.12E-6		9.64E-6	3.98E-5
HCT-116	0.242	2.422	2.313	2.376	2.066	0.245	0.086	95	98	84	.	-65		2.53E-6		1.00E-5	5.94E-5
HCT-15	0.355	2.654	2.549	2.611	2.301	0.211	0.098	95	98	85	-41	-73		1.89E-6		4.73E-6	1.96E-5
HT29	0.240	2.091	2.015	2.119	1.725	0.323	0.258	96	102	80	4	1		2.51E-6		> 1.00E-4	> 1.00E-4
KM12	0.467	3.002	2.920	2.921	2.517	0.536	0.285	97	97	81	3	-39		2.48E-6		1.16E-5	> 1.00E-4
SW-620	0.200	1.511	1.441	1.452	1.379	0.191	0.068	95	95	90	-5	-66		2.65E-6		8.96E-6	5.49E-5
CNS Cancer																	
SF-268	0.759	2.591	2.434	2.480	2.418	0.792	0.571	91	94	91	2	-25		2.86E-6		1.17E-5	> 1.00E-4
SF-295	0.484	1.792	1.605	1.645	1.566	0.376	0.021	86	89	83	-22	-96		2.05E-6		6.13E-6	2.38E-5
SF-539	0.884	2.800	2.649	2.750	2.775	0.346	0.010	92	97	99	-61	-99		2.02E-6		4.15E-6	8.54E-6
SNB-19	0.749	2.713	2.518	2.635	2.546	0.624	-0.006	90	96	92	-17	-100		2.42E-6		7.00E-6	2.51E-5
SNB-75	0.890	1.736	1.556	1.615	1.609	0.995	0.004	79	86	85	12	-100		3.03E-6		1.29E-5	3.61E-5
U251	0.256	1.717	1.589	1.604	1.512	0.273	0.151	91	92	86	1	-41		2.65E-6		1.06E-5	> 1.00E-4
Melanoma																	
LOX IMVI	0.406	3.048	2.977	2.987	2.787	0.335	0.157	97	98	90	-18	-61		2.36E-6		6.86E-6	5.51E-5
MALME-3M	0.679	1.767	1.622	1.635	0.815	0.010	-0.002	87	88	13	-99	-100		3.18E-7		1.30E-6	3.66E-6
M14	0.388	1.782	1.678	1.835	1.636	0.127	0.068	93	104	90	-67	-82		1.79E-6		3.72E-6	7.75E-6
MDA-MB-435	0.443	2.239	2.132	2.237	2.104	0.008	0.020	94	100	92	-98	-96		1.67E-6		3.05E-6	5.59E-6
SK-MEL-2	1.315	3.012	2.900	3.008	2.899	1.139	0.429	93	100	93	-13	-67		2.55E-6		7.49E-6	4.76E-5
SK-MEL-28	0.607	2.349	2.258	2.381	2.386	1.017	0.203	95	102	102	24	-67		4.60E-6		1.82E-5	6.55E-5
SK-MEL-5	0.752	3.259	3.158	3.219	2.325	0.024	0.009	96	98	63	-97	-99		1.20E-6		2.47E-6	5.09E-6
UACC-257	1.097	2.828	2.593	2.716	2.476	0.271	0.235	86	94	80	-75	-79		1.55E-6		3.27E-6	6.87E-6
UACC-62	1.057	3.157	3.086	3.106	2.882	0.281	0.232	97	98	87	-73	-78		1.70E-6		3.48E-6	7.14E-6
Ovarian Cancer																	
IGROV1	0.358	1.921	1.837	1.830	1.604	0.179	0.171	95	94	80	-50	-52		1.69E-6		4.11E-6	9.98E-6
OVCAR-3	0.547	2.171	2.160	2.169	1.027	0.390	0.096	99	100	30	-29	-83		5.12E-7		3.21E-6	2.48E-5
OVCAR-4	0.555	1.319	1.270	1.287	1.065	0.015	0.051	94	96	67	-97	-91		1.27E-6		2.55E-6	5.15E-6
OVCAR-5	0.528	1.766	1.624	1.647	1.671	0.297	0.039	89	90	92	-44	-93		2.05E-6		4.76E-6	1.34E-5
OVCAR-8	0.418	2.349	2.259	2.323	2.022	0.431	0.163	95	99	83	1	-61		2.52E-6		1.03E-5	6.61E-5
NCI/ADR-RES	0.621	2.263	2.244	2.315	2.114	0.504	0.467	99	103	91	-19	-25		2.36E-6		6.74E-6	> 1.00E-4
SK-OV-3	0.927	2.389	2.317	2.391	2.400	1.047	0.431	95	100	101	8	-54		3.53E-6		1.36E-5	8.76E-5
Renal Cancer																	
786-0	0.623	2.690	2.565	2.654	2.623	0.887	0.519	94	98	97	13	-17		3.60E-6		2.71E-5	> 1.00E-4
A498	1.828	2.812	2.798	2.788	2.585	1.293	0.102	99	97	77	-29	-94		1.79E-6		5.30E-6	2.08E-5
ACHN	0.320	1.693	1.693	1.739	1.566	0.332	0.015	100	103	91	1	-95		2.84E-6		1.02E-5	3.38E-5
CAKI-1	0.613	2.481	2.332	2.301	2.275	0.641	0.012	92	90	89	1	-98		2.79E-6		1.03E-5	3.29E-5
RXF 393	1.197	1.469	1.512	1.551	1.517	0.639	0.209	116	130	117	-47	-83		2.58E-6		5.20E-6	1.24E-5
SN12C	0.664	2.655	2.487	2.508	2.395	0.410	0.026	92	93	87	-38	-96		1.97E-6		4.94E-6	1.59E-5
TK-10	0.733	2.248	2.164	2.162	2.350	1.045	0.460	94	94	107	21	-37		4.55E-6		2.27E-5	> 1.00E-4
UO-31	0.630	2.030	1.829	1.917	1.739	0.613	-0.006	86	92	79	-3	-100		2.27E-6		9.25E-6	3.06E-5
Prostate Cancer																	
PC-3	0.511	2.292	2.213	2.224	2.005	0.568	0.432	96	96	84	3	-15		2.63E-6		1.48E-5	> 1.00E-4
DU-145	0.346	1.770	1.732	1.834	1.763	0.447	0.061	97	104	100	7	-82		3.43E-6		1.20E-5	4.35E-5
Breast Cancer																	
MCF7	0.309	1.813	1.731	1.781	1.539	0.110	0.103	95	98	82	-64	-67		1.65E-6		3.63E-6	7.97E-6
MDA-MB-231/ATCC	0.656	1.658	1.594	1.567	1.541	0.507	0.566	94	91	88	-23	-14		2.21E-6		6.24E-6	> 1.00E-4
HS 578T	0.915	2.037	1.983	1.951	1.937	1.186	0.727	95	92	91	24	-21		4.10E-6		3.47E-5	> 1.00E-4
BT-549	1.174	2.572	2.366	2.519	2.483	1.360	0.099	85	96	94	13	-92		3.49E-6		1.34E-5	4.01E-5
T-47D	1.075	2.781	2.591	2.558	2.694	0.713	0.614	89	87	95	-34	-43		2.23E-6		5.47E-6	> 1.00E-4
MDA-MB-468	0.915	1.680	1.595	1.643	1.471	0.442	0.240	89	95	73	-52	-74		1.52E-6		3.84E-6	9.68E-6

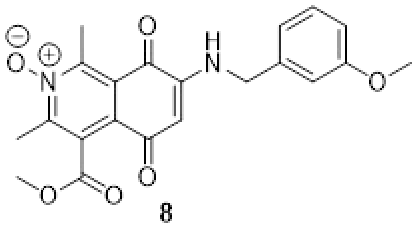

8

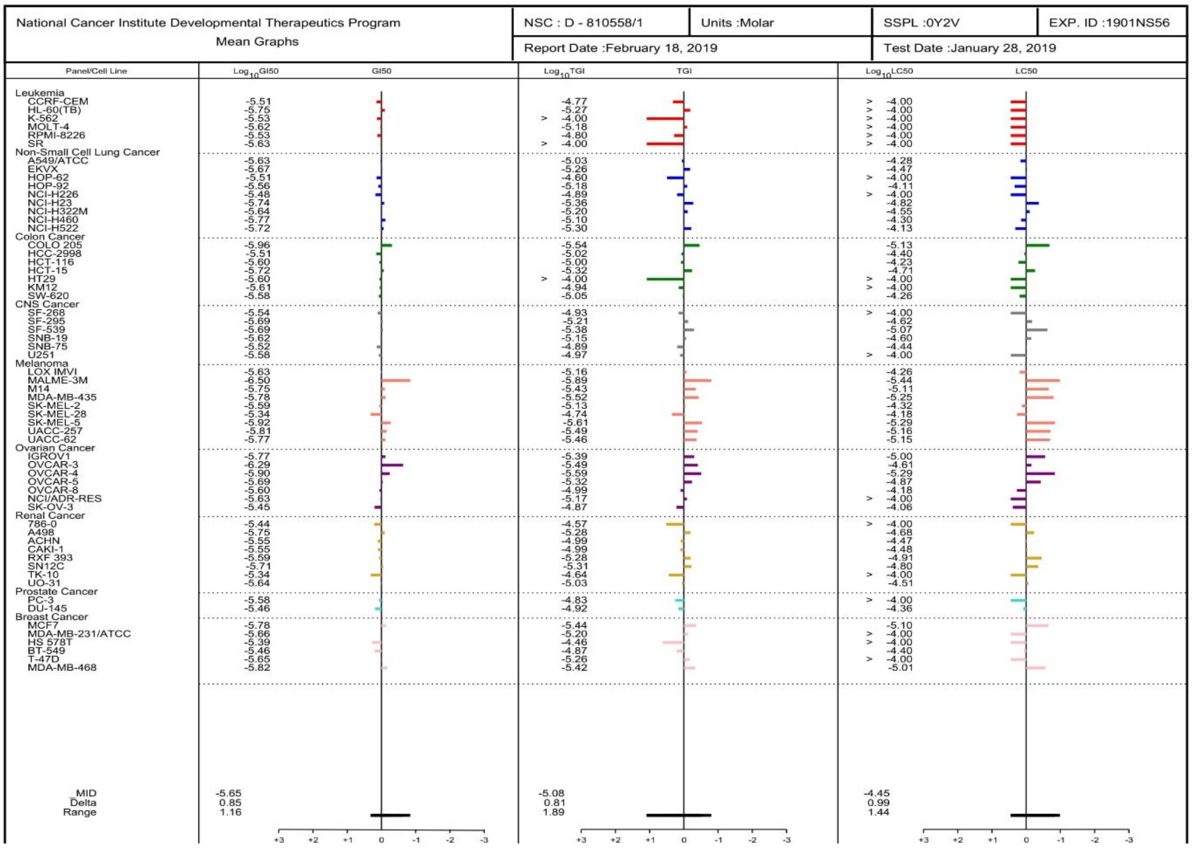

Mean of GI_{50} across 60 cell lines for compound **8** as Log_{10} Concentration (SD): -5.655 (±0.19)

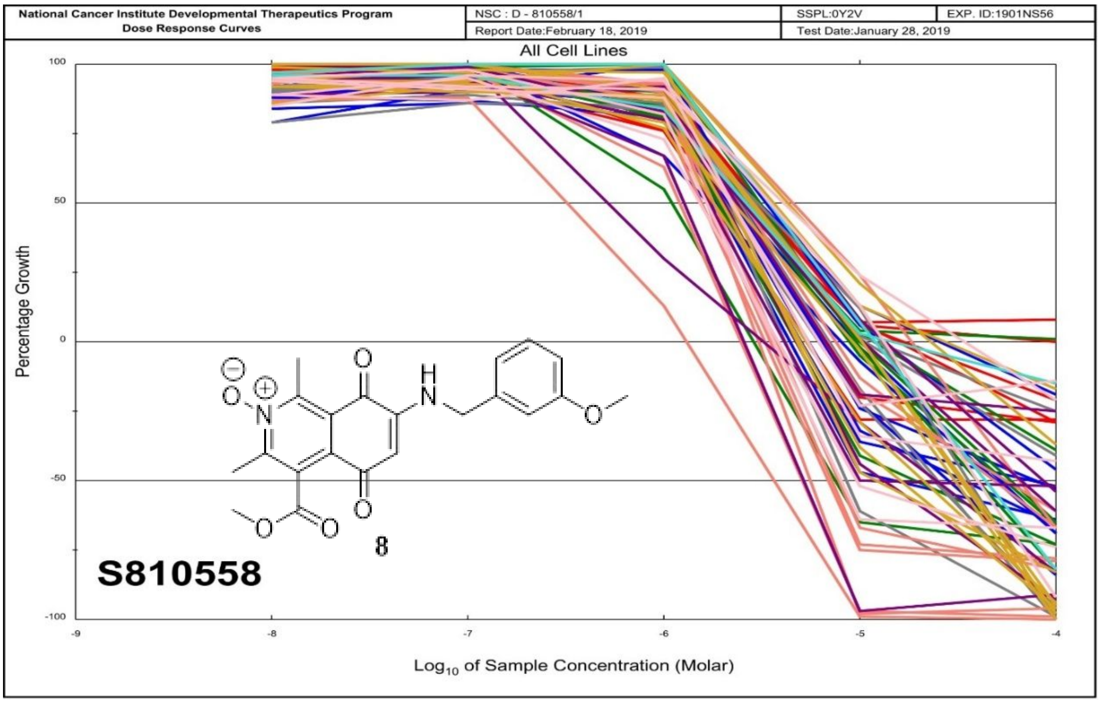

Figure S18** NCI Five Dose data cell line comparison summary for compound **8

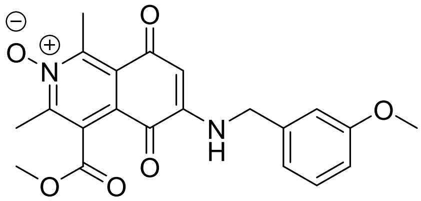

9

Developmental Therapeutics Program
One Dose Mean Graph

NSC: D-815405 / 1	Conc: 1.00E-5 Molar	Test Date: May 20, 2019
Experiment ID: 1905OS11		Report Date: Jun 10, 2019

Panel/Cell Line	Growth Percent
Leukemia	
CCRF-CEM	-17.07
HL-60(TB)	-37.28
K-562	-23.56
MOLT-4	-45.86
RPMI-8226	-35.52
SR	-8.20
Non-Small Cell Lung Cancer	
A549/ATCC	-52.10
EKVX	-68.70
HOP-62	-15.13
HOP-92	-43.56
NCI-H23	-69.17
NCI-H322M	-19.58
NCI-H460	-41.56
NCI-H522	-54.72
Colon Cancer	
COLO 205	-61.51
HCC-2998	-64.68
HCT-116	-60.07
HCT-15	-74.83
HT29	2.57
KM12	-21.15
SW-620	-41.22
CNS Cancer	
SF-268	-16.05
SF-295	-97.53
SF-539	-92.28
SNB-19	-88.42
SNB-75	-97.73
U251	-39.26
Melanoma	
LOX IMVI	-79.69
MALME-3M	-98.93
M14	-83.43
MDA-MB-435	-94.68
SK-MEL-2	-64.61
SK-MEL-28	-22.34
SK-MEL-5	-97.15
UACC-257	-87.06
UACC-62	-68.09
Ovarian Cancer	
IGROV1	-64.02
OVCAR-3	-50.96
OVCAR-5	-90.27
OVCAR-8	-23.70
NCI/ADR-RES	-25.59
SK-OV-3	-11.11
Renal Cancer	
786-0	-3.41
ACHN	-81.51
CAKI-1	-32.63
RXF 393	-46.67
SN12C	-93.44
TK-10	4.50
UO-31	-62.47
Prostate Cancer	
PC-3	-2.04
DU-145	-99.07
Breast Cancer	
MCF7	-65.36
MDA-MB-231/ATCC	-17.41
HS 578T	-22.15
BT-549	-95.01
T-47D	-43.95
MDA-MB-468	-67.84
Mean	-52.18
Delta	46.89
Range	103.57

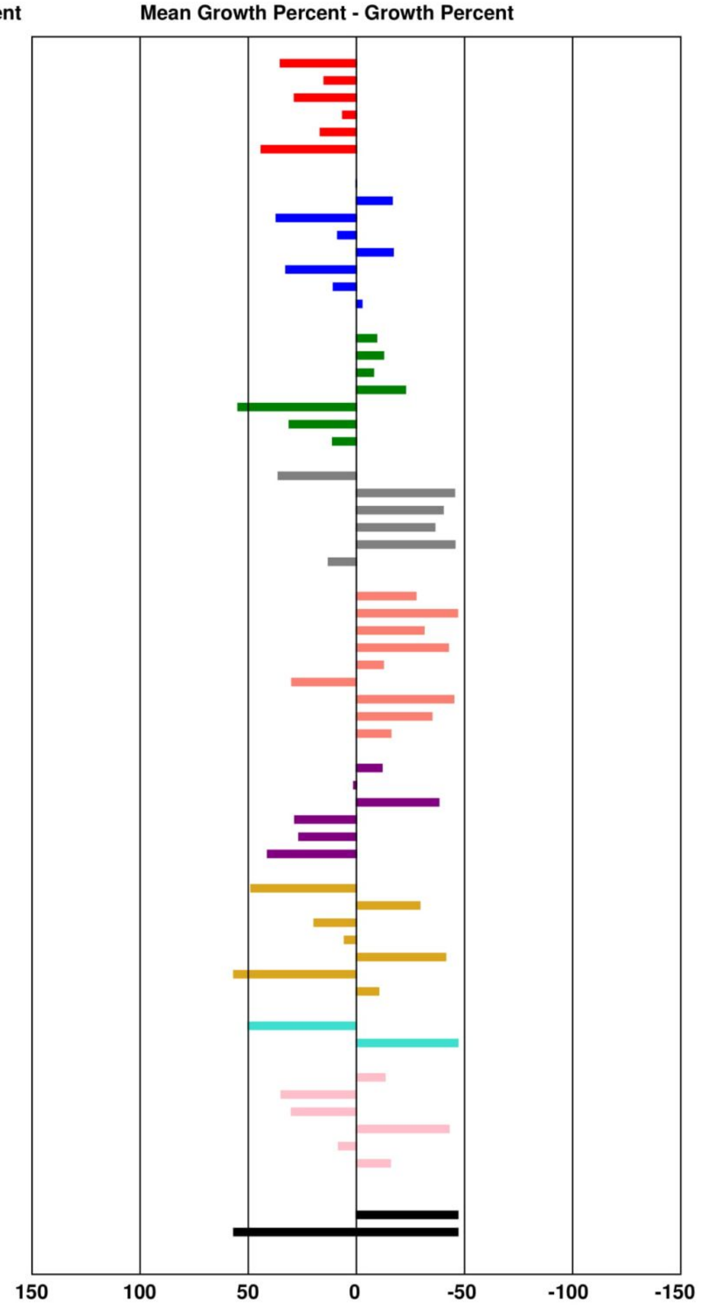

Figure S19 *NCI One Dose (10 μM) data for compound **9***

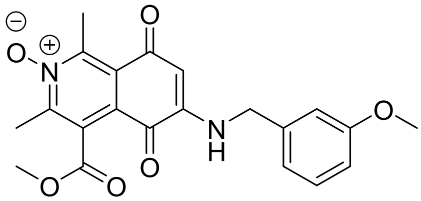

9

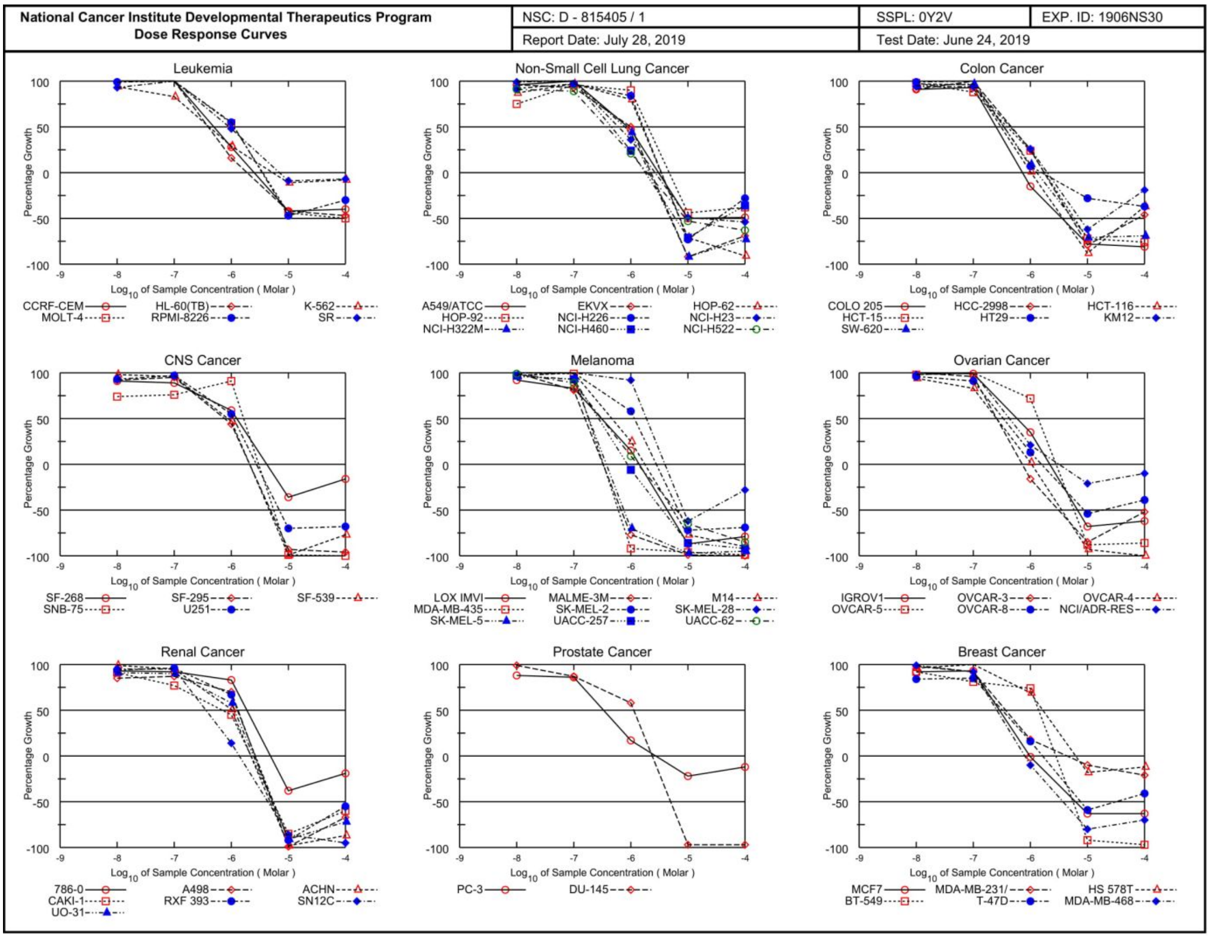

Figure S20 *NCI Five Dose response summary data for compound* ***9***

Table S7 *NCI Five Dose raw data for compound* ***9***

National Cancer Institute Developmental Therapeutics Program
In-Vitro Testing Results

NSC : D - 815405 / 1	Experiment ID : 1906NS30	Test Type : 08	Units : Molar
Report Date : July 28, 2019	Test Date : June 24, 2019	QNS :	MC :
COMI : RK 6.3.8.1	Stain Reagent : SRB Dual-Pass Related	SSPL : 0Y2V	

			Log10 Concentration														
	Time		Mean Optical Densities					Percent Growth									
Panel/Cell Line	Zero	Ctrl	-8.0	-7.0	-6.0	-5.0	-4.0	-8.0	-7.0	-6.0	-5.0	-4.0		GI50	TGI	LC50
Leukemia																
CCRF-CEM	0.555	2.186	2.261	2.292	1.014	0.322	0.335	105	106	28	-42	-40		5.26E-7	2.52E-6	> 1.00E-4
HL-60(TB)	0.970	3.173	3.232	3.215	1.319	0.565	0.512	103	102	16	-42	-47		4.01E-7	1.88E-6	> 1.00E-4
K-562	0.175	1.356	1.286	1.161	0.522	0.155	0.162	94	83	29	-11	-8		4.16E-7	5.25E-6	> 1.00E-4
MOLT-4	0.690	2.754	2.817	2.913	1.795	0.386	0.345	103	108	54	-44	-50		1.09E-6	3.54E-6	9.73E-5
RPMI-8226	0.896	2.596	2.585	2.663	1.837	0.477	0.631	99	104	55	-47	-30		1.13E-6	3.48E-6	> 1.00E-4
SR	0.401	1.174	1.124	1.197	0.776	0.367	0.374	93	103	48	-9	-7		9.38E-7	7.07E-6	> 1.00E-4
Non-Small Cell Lung Cancer																
A549/ATCC	0.290	1.791	1.732	1.788	1.002	0.145	0.147	96	100	47	-50	-49		8.92E-7	3.06E-6	.
EKVX	0.554	1.815	1.760	1.728	1.191	0.046	0.173	96	93	50	-92	-69		1.01E-6	2.26E-6	5.08E-6
HOP-62	0.711	1.786	1.650	1.756	1.573	0.207	0.066	87	97	80	-71	-91		1.58E-6	3.39E-6	7.27E-6
HOP-92	1.036	1.546	1.419	1.526	1.496	0.581	0.645	75	96	90	-44	-38		1.99E-6	4.70E-6	> 1.00E-4
NCI-H226	1.126	2.425	2.324	2.391	2.215	0.304	0.807	92	97	84	-73	-28		1.64E-6	3.42E-6	.
NCI-H23	0.613	1.974	1.966	2.011	1.109	0.311	0.281	99	103	36	-49	-54		6.24E-7	2.66E-6	1.40E-5
NCI-H322M	0.743	2.003	2.022	2.020	1.296	0.060	0.200	101	101	44	-92	-73		7.82E-7	2.10E-6	4.91E-6
NCI-H460	0.287	2.867	2.884	2.919	0.894	0.082	0.185	101	102	24	-71	-36		4.60E-7	1.77E-6	.
NCI-H522	0.820	2.274	2.137	2.110	1.127	0.384	0.301	91	89	21	-53	-63		3.74E-7	1.92E-6	9.06E-6
Colon Cancer																
COLO 205	0.585	2.266	2.106	2.152	0.499	0.129	0.114	91	93	-15	-78	-81		2.52E-7	7.31E-7	3.61E-6
HCC-2998	0.932	2.470	2.324	2.605	1.318	0.200	0.506	91	109	25	-79	-46		5.04E-7	1.75E-6	.
HCT-116	0.244	1.999	1.948	1.924	0.255	0.029	0.154	97	96	1	-88	-37		3.02E-7	1.02E-6	.
HCT-15	0.278	2.133	2.103	1.910	0.729	0.077	0.066	98	88	24	-72	-76		3.95E-7	1.78E-6	5.86E-6
HT29	0.208	1.584	1.569	1.684	0.299	0.149	0.131	99	107	7	-28	-37		3.70E-7	1.54E-6	> 1.00E-4
KM12	0.579	2.905	2.770	2.776	1.176	0.221	0.471	94	94	26	-62	-19		4.43E-7	1.96E-6	.
SW-620	0.258	2.017	1.936	1.962	0.418	0.076	0.079	95	97	9	-71	-69		3.42E-7	1.30E-6	5.50E-6
CNS Cancer																
SF-268	0.838	2.465	2.319	2.285	1.805	0.538	0.704	91	89	59	-36	-16		1.26E-6	4.21E-6	> 1.00E-4
SF-295	0.781	2.881	2.713	2.778	1.711	0.053	0.031	92	95	44	-93	-96		7.72E-7	2.10E-6	4.85E-6
SF-539	1.054	2.873	2.841	2.804	1.914	0.015	0.246	98	96	47	-99	-77		8.79E-7	2.11E-6	4.64E-6
SNB-75	1.027	1.774	1.577	1.592	1.706	0.010	0.004	74	76	91	-99	-100		1.64E-6	3.01E-6	5.52E-6
U251	0.272	1.491	1.403	1.458	0.939	0.083	0.088	93	97	55	-70	-68		1.09E-6	2.75E-6	6.95E-6
Melanoma																
LOX IMVI	0.496	2.712	2.535	2.326	0.820	0.065	0.104	92	83	15	-87	-79		3.02E-7	1.39E-6	4.32E-6
MALME-3M	0.763	1.612	1.626	1.447	0.174	0.007	-0.003	102	81	-77	-99	-100		1.56E-7	3.24E-7	6.72E-7
M14	0.430	1.699	1.660	1.834	0.745	0.097	0.045	97	111	25	-77	-90		5.08E-7	1.75E-6	5.39E-6
MDA-MB-435	0.512	2.483	2.482	2.466	0.042	0.023	0.007	100	99	-92	-96	-99		1.81E-7	3.31E-7	6.04E-7
SK-MEL-2	1.127	2.537	2.491	2.536	1.940	0.315	0.353	97	100	58	-72	-69		1.15E-6	2.78E-6	6.76E-6
SK-MEL-28	0.772	1.962	2.087	2.172	1.871	0.295	0.558	110	118	92	-62	-28		1.88E-6	3.97E-6	.
SK-MEL-5	0.784	3.090	2.998	2.920	0.234	0.026	0.042	96	93	-70	-97	-95		1.83E-7	3.71E-7	7.52E-7
UACC-257	0.739	1.821	1.783	1.747	0.693	0.106	0.056	97	93	-6	-86	-92		2.72E-7	8.64E-7	3.55E-6
UACC-62	0.975	2.665	2.652	2.478	1.124	0.339	0.145	99	89	9	-65	-85		3.06E-7	1.31E-6	6.23E-6
Ovarian Cancer																
IGROV1	0.547	2.182	2.190	2.164	1.116	0.177	0.207	100	99	35	-68	-62		5.79E-7	2.18E-6	6.71E-6
OVCAR-3	0.469	1.560	1.571	1.520	0.395	0.070	0.226	101	96	-16	-85	-52		2.59E-7	7.23E-7	3.11E-6
OVCAR-4	0.560	1.468	1.415	1.310	0.576	0.040	0.002	94	83	2	-93	-100		2.53E-7	1.04E-6	3.52E-6
OVCAR-5	0.600	1.577	1.562	1.618	1.300	0.071	0.087	98	104	72	-88	-86		1.37E-6	2.81E-6	5.76E-6
OVCAR-8	0.444	2.253	2.185	2.099	0.685	0.205	0.273	96	91	13	-54	-39		3.39E-7	1.58E-6	.
NCI/ADR-RES	0.563	2.038	2.003	2.066	0.879	0.442	0.506	98	102	21	-21	-10		4.41E-7	3.15E-6	> 1.00E-4
Renal Cancer																
786-0	0.921	2.695	2.566	2.554	2.400	0.569	0.747	93	92	83	-38	-19		1.88E-6	4.85E-6	> 1.00E-4
A498	1.553	2.117	2.035	2.046	1.947	0.017	0.517	85	87	70	-99	-67		1.31E-6	2.59E-6	5.13E-6
ACHN	0.354	1.667	1.657	1.600	1.019	0.007	0.045	99	95	51	-98	-87		1.01E-6	2.19E-6	4.75E-6
CAKI-1	0.772	2.588	2.422	2.170	1.589	0.119	0.312	91	77	45	-85	-60		6.96E-7	2.22E-6	5.41E-6
RXF 393	0.883	1.657	1.610	1.628	1.400	0.067	0.396	94	96	67	-92	-55		1.27E-6	2.63E-6	5.41E-6
SN12C	0.558	2.071	1.990	2.001	0.763	0.077	0.028	95	95	14	-86	-95		3.59E-7	1.37E-6	4.34E-6
UO-31	0.542	1.687	1.584	1.575	1.202	0.049	0.152	91	90	58	-91	-72		1.13E-6	2.44E-6	5.30E-6
Prostate Cancer																
PC-3	0.573	1.601	1.480	1.459	0.746	0.448	0.503	88	86	17	-22	-12		3.33E-7	2.73E-6	> 1.00E-4
DU-145	0.410	1.744	1.728	1.576	1.181	0.012	0.011	99	87	58	-97	-97		1.12E-6	2.36E-6	4.97E-6
Breast Cancer																
MCF7	0.467	2.495	2.339	2.351	0.462	0.173	0.174	92	93	-1	-63	-63		2.86E-7	9.72E-7	6.17E-6
MDA-MB-231/ATCC	0.775	1.916	1.889	1.829	0.981	0.697	0.609	98	92	18	-10	-21		3.71E-7	4.37E-6	> 1.00E-4
HS 578T	0.869	1.950	1.904	1.983	1.611	0.716	0.761	96	103	69	-18	-12		1.64E-6	6.25E-6	> 1.00E-4
BT-549	1.031	1.944	1.867	1.771	1.709	0.085	0.033	92	81	74	-92	-97		1.40E-6	2.80E-6	5.60E-6
T-47D	0.723	1.661	1.507	1.520	0.874	0.297	0.425	84	85	16	-59	-41		3.22E-7	1.64E-6	.
MDA-MB-468	0.732	1.342	1.337	1.291	0.659	0.144	0.219	99	92	-10	-80	-70		2.57E-7	7.98E-7	3.71E-6

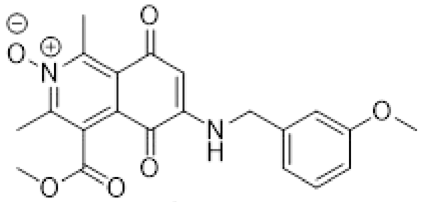

9

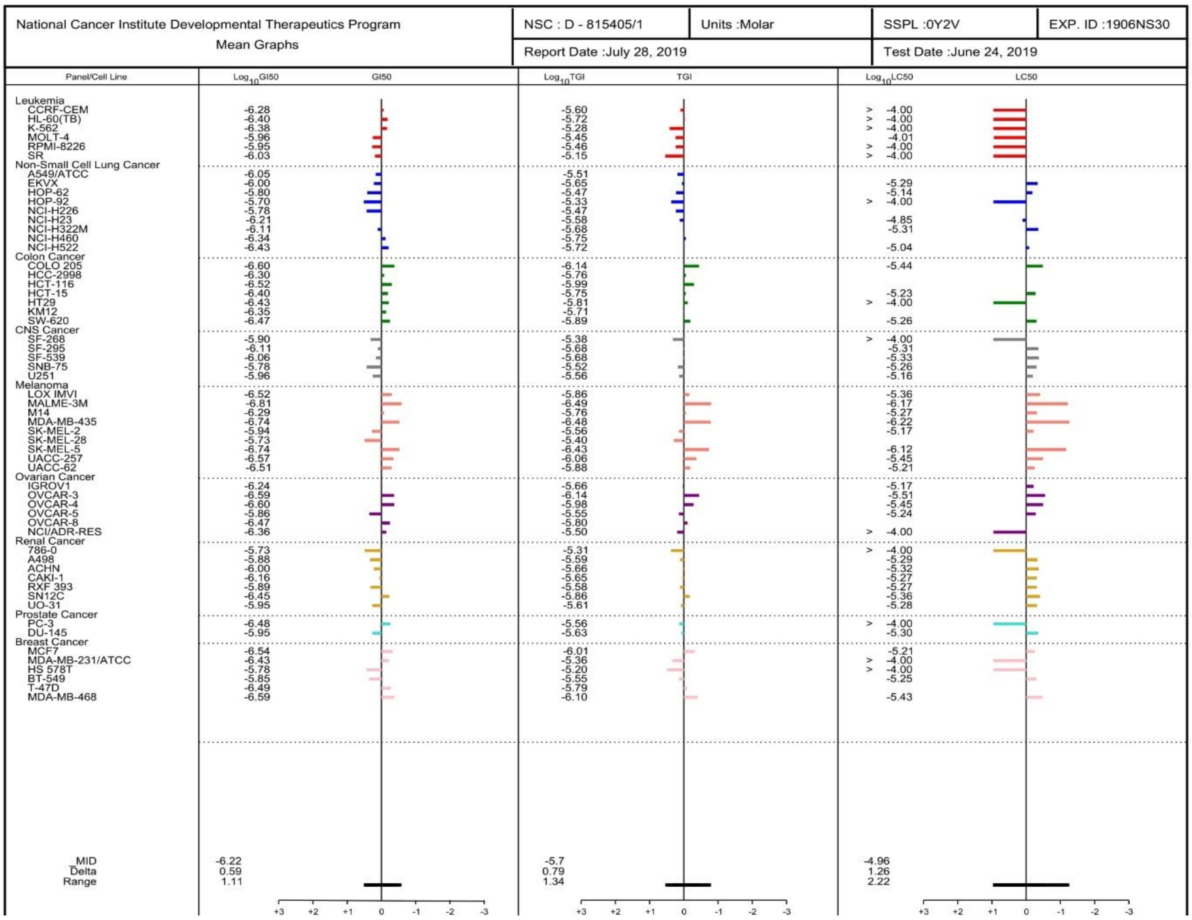

Mean of GI_{50} across 58 cell lines for compound **9** as Log_{10} Concentration (SD): -6.217 (±0.66)

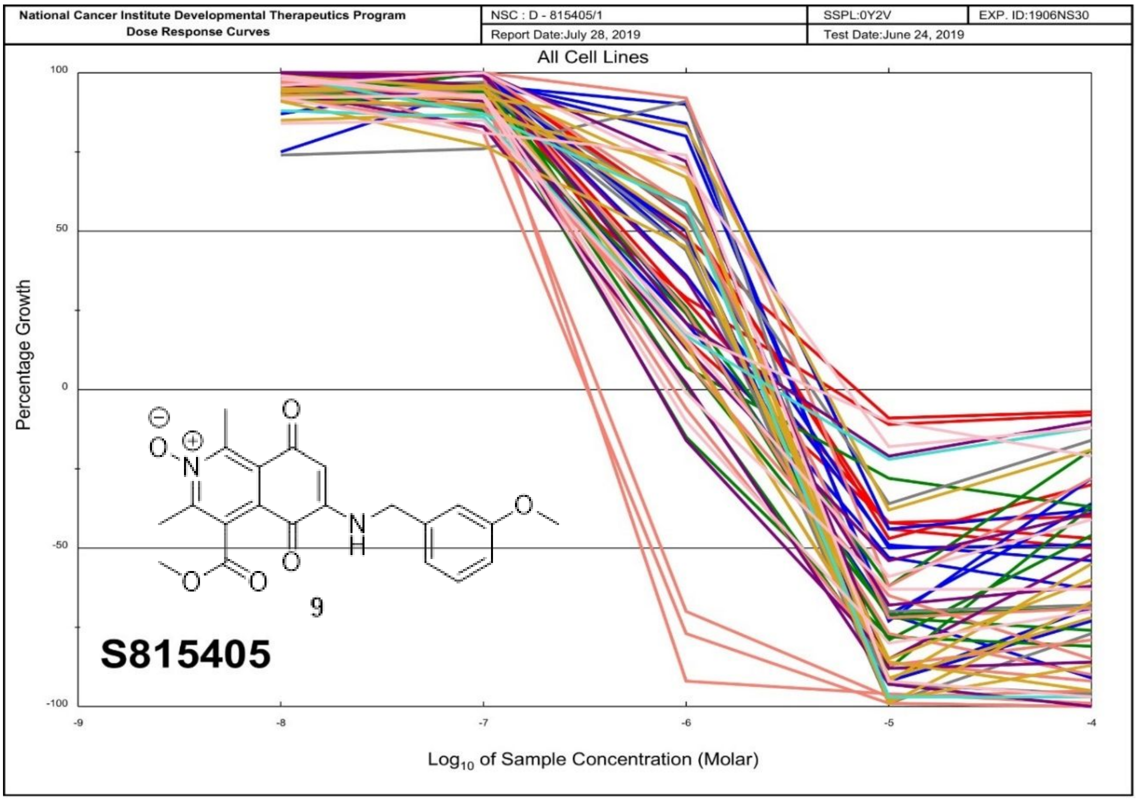

Figure S21 *NCI Five Dose data cell line comparison summary for compound* ***9***

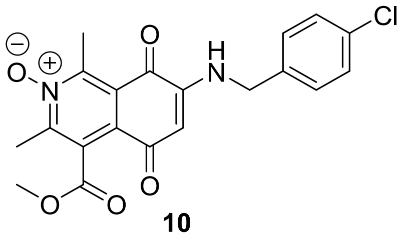

10

Developmental Therapeutics Program One Dose Mean Graph	NSC: D-810559 / 1	Conc: 1.00E-5 Molar	Test Date: Dec 03, 2018
	Experiment ID: 1812OS40		Report Date: Dec 20, 2018

Panel/Cell Line	Growth Percent
**Leukemia**	
CCRF-CEM	57.96
HL-60(TB)	80.28
K-562	28.71
MOLT-4	72.77
RPMI-8226	69.77
SR	48.63
**Non-Small Cell Lung Cancer**	
A549/ATCC	66.86
EKVX	70.92
HOP-62	78.21
HOP-92	107.42
NCI-H226	92.62
NCI-H23	59.00
NCI-H322M	64.27
NCI-H460	51.76
NCI-H522	41.96
**Colon Cancer**	
COLO 205	9.79
HCC-2998	59.62
HCT-116	11.46
HCT-15	39.65
HT29	9.34
KM12	48.81
SW-620	45.94
**CNS Cancer**	
SF-268	76.84
SF-295	87.55
SF-539	85.28
SNB-19	84.44
SNB-75	131.13
U251	57.20
**Melanoma**	
LOX IMVI	45.83
MALME-3M	-75.37
M14	39.63
MDA-MB-435	9.83
SK-MEL-2	83.36
SK-MEL-28	95.68
SK-MEL-5	29.95
UACC-257	17.82
UACC-62	6.15
**Ovarian Cancer**	
IGROV1	61.19
OVCAR-3	-7.64
OVCAR-4	36.63
OVCAR-5	97.34
OVCAR-8	33.12
NCI/ADR-RES	54.84
SK-OV-3	95.83
**Renal Cancer**	
786-0	74.05
A498	88.22
ACHN	67.46
CAKI-1	76.31
RXF 393	86.58
SN12C	37.97
TK-10	111.25
UO-31	78.96
**Prostate Cancer**	
PC-3	33.55
DU-145	86.63
**Breast Cancer**	
MCF7	35.90
MDA-MB-231/ATCC	48.70
HS 578T	92.22
BT-549	77.87
T-47D	37.55
MDA-MB-468	39.81
Mean	57.26
Delta	132.63
Range	206.50

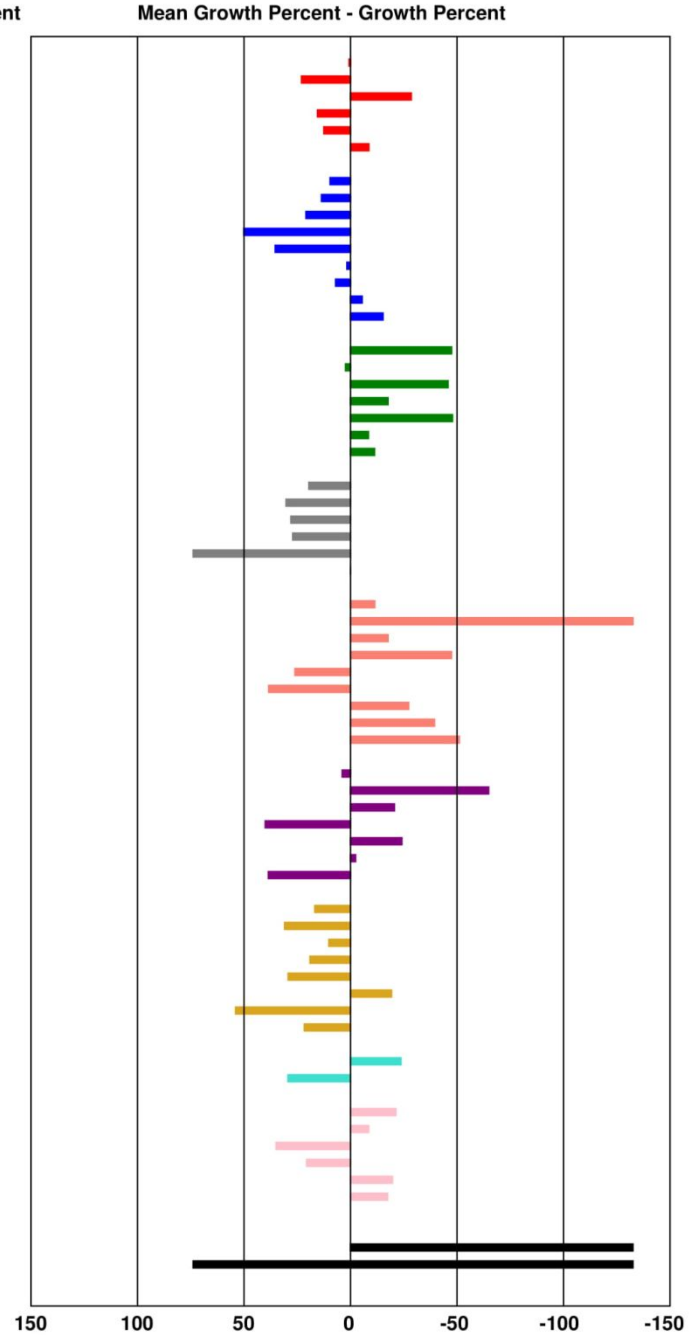

Figure S22 *NCI One Dose (10 µM) data for compound* ***10***

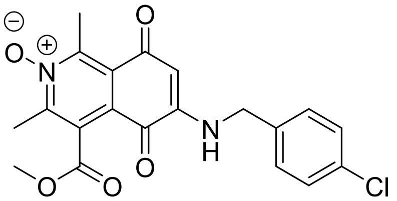

11

Developmental Therapeutics Program One Dose Mean Graph	NSC: D-815402 / 1	Conc: 1.00E-5 Molar	Test Date: May 20, 2019
	Experiment ID: 1905OS11		Report Date: Jun 10, 2019

Panel/Cell Line	Growth Percent
Leukemia	
CCRF-CEM	-6.94
HL-60(TB)	-30.53
K-562	1.55
MOLT-4	-32.17
RPMI-8226	-16.19
SR	3.91
Non-Small Cell Lung Cancer	
A549/ATCC	-16.53
EKVX	-50.00
HOP-62	2.25
HOP-92	-27.93
NCI-H23	-35.70
NCI-H322M	-7.11
NCI-H460	0.38
NCI-H522	-15.12
Colon Cancer	
COLO 205	-57.85
HCC-2998	-34.58
HCT-116	-19.92
HCT-15	-54.83
HT29	2.63
KM12	-4.32
SW-620	-17.28
CNS Cancer	
SF-268	0.31
SF-295	-59.74
SF-539	-89.83
SNB-19	-23.52
SNB-75	-94.75
U251	-7.46
Melanoma	
LOX IMVI	-23.24
MALME-3M	-95.05
M14	-85.52
MDA-MB-435	-96.08
SK-MEL-2	-30.93
SK-MEL-28	10.58
SK-MEL-5	-98.75
UACC-257	-84.74
UACC-62	-65.49
Ovarian Cancer	
IGROV1	-50.88
OVCAR-3	-28.56
OVCAR-5	-68.99
OVCAR-8	2.34
NCI/ADR-RES	-15.14
SK-OV-3	4.93
Renal Cancer	
786-0	7.94
ACHN	-19.13
CAKI-1	-3.15
RXF 393	-31.67
SN12C	-84.92
TK-10	21.67
UO-31	-9.07
Prostate Cancer	
PC-3	1.07
DU-145	-83.66
Breast Cancer	
MCF7	-63.23
MDA-MB-231/ATCC	-20.69
HS 578T	-18.98
BT-549	-23.77
T-47D	-33.76
MDA-MB-468	-66.95
Mean	-32.37
Delta	66.38
Range	120.42

Mean Growth Percent - Growth Percent

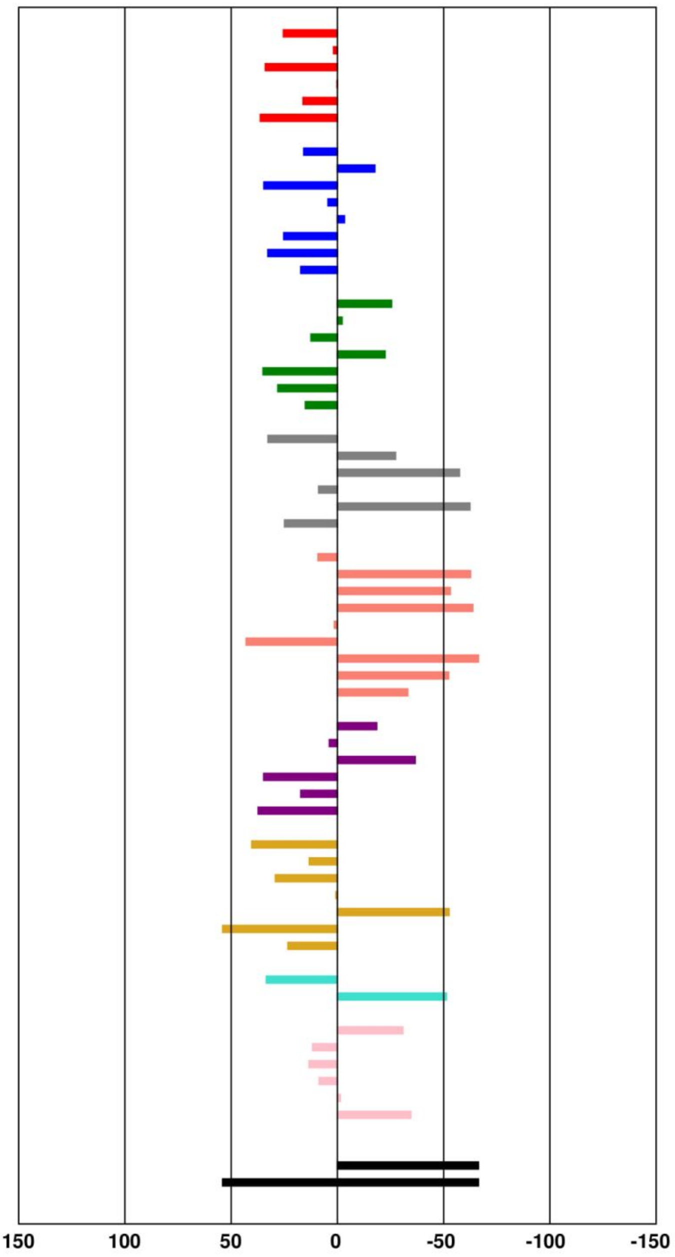

Figure S23 *NCI One Dose (10 µM) data for compound* ***11***

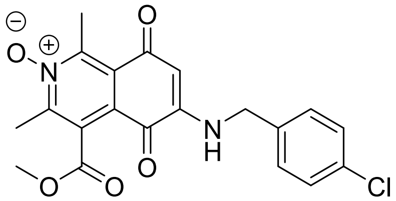

11

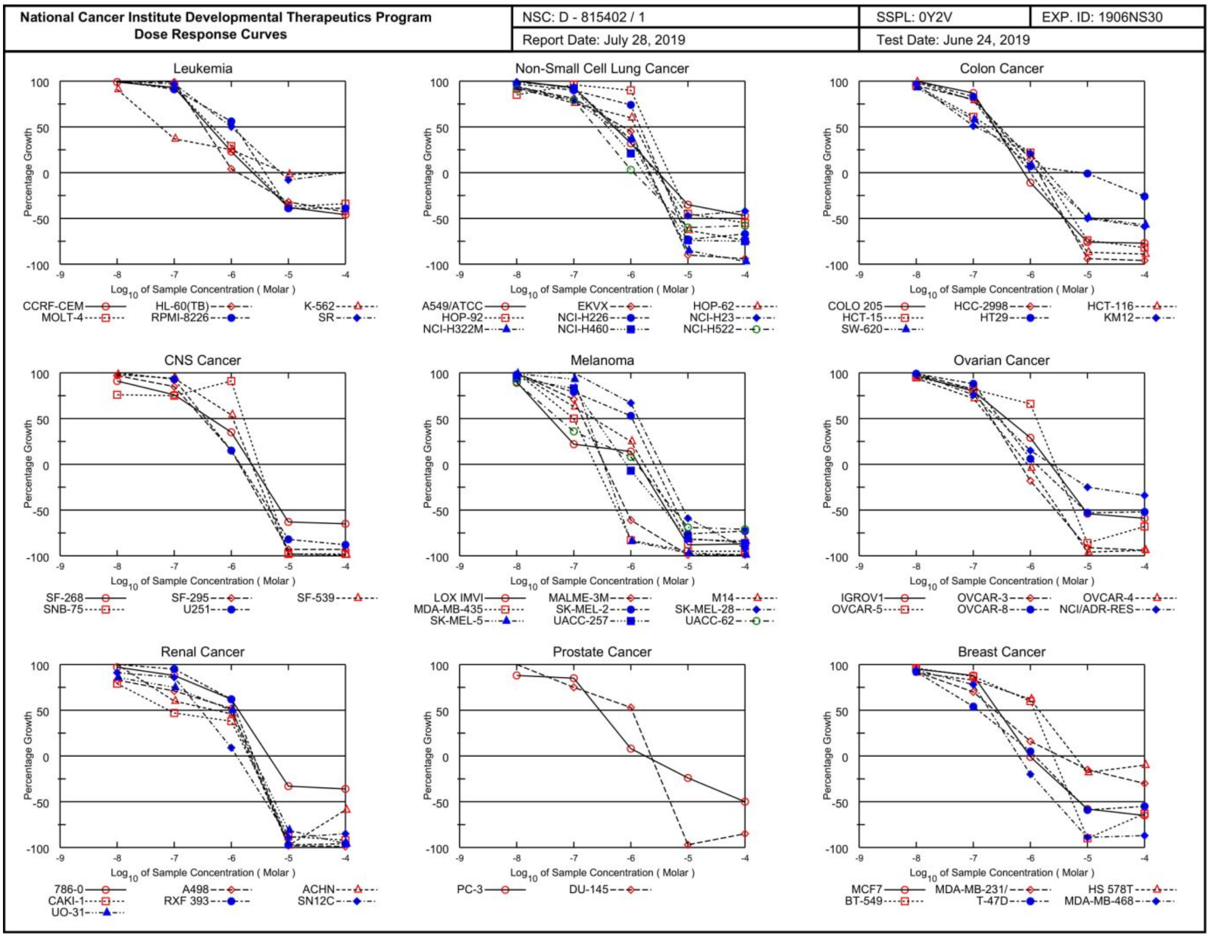

Figure S24 *NCI Five Dose response summary data for compound* ***11***

Table S8 *NCI Five Dose raw data for compound* ***11***

National Cancer Institute Developmental Therapeutics Program
In-Vitro Testing Results

NSC : D - 815402 / 1	Experiment ID : 1906NS30	Test Type : 08	Units : Molar
Report Date : July 28, 2019	Test Date : June 24, 2019	QNS :	MC :
COMI : RK 6.4.0.1	Stain Reagent : SRB Dual-Pass Related	SSPL : 0Y2V	

			Log10 Concentration												
	Time		Mean Optical Densities					Percent Growth							
Panel/Cell Line	Zero	Ctrl	-8.0	-7.0	-6.0	-5.0	-4.0	-8.0	-7.0	-6.0	-5.0	-4.0	GI50	TGI	LC50
Leukemia															
CCRF-CEM	0.555	2.248	2.229	2.126	0.945	0.346	0.298	99	93	23	-38	-46	4.11E-7	2.40E-6	> 1.00E-4
HL-60(TB)	0.970	3.208	3.199	3.177	1.059	0.656	0.555	100	99	4	-32	-43	3.26E-7	1.28E-6	> 1.00E-4
K-562	0.175	1.331	1.231	0.607	0.463	0.171	0.178	91	37	25	-2	.	5.82E-8	.	> 1.00E-4
MOLT-4	0.690	2.640	2.666	2.503	1.254	0.432	0.452	101	93	29	-37	-34	4.69E-7	2.73E-6	> 1.00E-4
RPMI-8226	0.896	2.641	2.645	2.489	1.878	0.546	0.546	100	91	56	-39	-39	1.16E-6	3.89E-6	> 1.00E-4
SR	0.401	1.202	1.204	1.176	0.805	0.371	0.403	100	97	50	-8	.	1.02E-6	.	> 1.00E-4
Non-Small Cell Lung Cancer															
A549/ATCC	0.290	1.823	1.840	1.710	0.787	0.188	0.154	101	93	32	-35	-47	5.11E-7	3.02E-6	> 1.00E-4
EKVX	0.554	1.791	1.718	1.556	1.115	0.056	0.032	94	81	45	-90	-94	7.41E-7	2.16E-6	5.07E-6
HOP-62	0.711	1.841	1.762	1.568	1.385	0.262	0.186	93	76	60	-63	-74	1.20E-6	3.06E-6	7.81E-6
HOP-92	1.036	1.563	1.486	1.544	1.510	0.569	0.471	85	96	90	-45	-55	1.97E-6	4.63E-6	3.29E-5
NCI-H226	1.126	2.430	2.384	2.297	2.088	0.310	0.376	97	90	74	-73	-67	1.45E-6	3.19E-6	7.02E-6
NCI-H23	0.613	1.958	1.947	1.870	1.093	0.322	0.359	99	93	36	-47	-42	5.64E-7	2.68E-6	> 1.00E-4
NCI-H322M	0.743	2.016	1.934	1.751	1.210	0.115	0.022	94	79	37	-85	-97	4.87E-7	2.01E-6	5.19E-6
NCI-H460	0.287	2.865	2.909	2.659	0.820	0.075	0.073	102	92	21	-74	-75	3.88E-7	1.65E-6	5.59E-6
NCI-H522	0.820	2.280	2.148	1.979	0.868	0.326	0.345	91	79	3	-60	-58	2.43E-7	1.13E-6	6.90E-6
Colon Cancer															
COLO 205	0.585	2.227	2.227	2.009	0.521	0.142	0.137	100	87	-11	-76	-77	2.38E-7	7.73E-7	4.00E-6
HCC-2998	0.932	2.615	2.538	2.285	1.180	0.053	0.041	95	80	15	-94	-96	2.90E-7	1.36E-6	3.92E-6
HCT-116	0.244	1.933	1.921	1.576	0.248	0.032	0.028	99	79	.	-87	-89	2.33E-7	1.01E-6	3.76E-6
HCT-15	0.278	2.091	2.004	1.378	0.680	0.072	0.050	95	61	22	-74	-82	1.89E-7	1.70E-6	5.62E-6
HT29	0.208	1.606	1.654	1.367	0.304	0.206	0.154	103	83	7	-1	-26	2.71E-7	7.09E-6	> 1.00E-4
KM12	0.579	2.943	2.883	1.774	1.064	0.287	0.235	97	51	21	-50	-59	1.04E-7	1.95E-6	9.86E-6
SW-620	0.258	2.021	1.899	1.275	0.395	0.131	0.111	93	58	8	-49	-57	1.43E-7	1.37E-6	1.26E-5
CNS Cancer															
SF-268	0.838	2.476	2.327	2.078	1.414	0.314	0.292	91	76	35	-63	-65	4.30E-7	2.29E-6	7.43E-6
SF-295	0.781	2.888	2.835	2.564	1.103	0.058	0.056	97	85	15	-93	-93	3.16E-7	1.39E-6	4.03E-6
SF-539	1.054	2.853	2.819	2.749	2.031	0.019	0.006	98	94	54	-98	-99	1.07E-6	2.27E-6	4.83E-6
SNB-75	1.027	1.815	1.626	1.617	1.744	0.022	0.020	76	75	91	-98	-98	1.65E-6	3.03E-6	5.58E-6
U251	0.272	1.505	1.519	1.420	0.460	0.049	0.032	101	93	15	-82	-88	3.57E-7	1.43E-6	4.69E-6
Melanoma															
LOX IMVI	0.496	2.718	2.491	0.980	0.798	0.060	0.063	90	22	14	-88	-87	3.85E-8	1.36E-6	4.23E-6
MALME-3M	0.763	1.652	1.671	1.395	0.296	0.010	0.011	102	71	-61	-99	-99	1.44E-7	3.45E-7	8.23E-7
M14	0.430	1.690	1.729	1.227	0.742	0.079	0.070	103	63	25	-82	-84	2.20E-7	1.71E-6	5.04E-6
MDA-MB-435	0.512	2.551	2.596	1.527	0.085	0.027	0.027	102	50	-83	-95	-95	9.90E-8	2.36E-7	5.61E-7
SK-MEL-2	1.127	2.488	2.456	2.203	1.854	0.273	0.300	98	79	53	-76	-73	1.06E-6	2.59E-6	6.31E-6
SK-MEL-28	0.772	1.999	2.066	2.054	1.600	0.313	0.061	105	104	67	-59	-92	1.37E-6	3.40E-6	8.42E-6
SK-MEL-5	0.784	3.077	3.047	2.911	0.123	0.024	0.008	99	93	-84	-97	-99	1.74E-7	3.34E-7	6.40E-7
UACC-257	0.739	1.852	1.788	1.664	0.684	0.138	0.103	94	83	-7	-81	-86	2.32E-7	8.28E-7	3.76E-6
UACC-62	0.975	2.664	2.485	1.582	1.103	0.302	0.285	89	36	8	-69	-71	5.45E-8	1.25E-6	5.64E-6
Ovarian Cancer															
IGROV1	0.547	2.238	2.187	1.903	1.040	0.251	0.227	97	80	29	-54	-59	3.90E-7	2.24E-6	8.92E-6
OVCAR-3	0.469	1.575	1.560	1.372	0.384	0.040	0.028	99	82	-18	-91	-94	2.08E-7	6.58E-7	2.72E-6
OVCAR-4	0.560	1.511	1.451	1.241	0.539	0.022	0.035	94	72	-4	-96	-94	1.93E-7	8.89E-7	3.16E-6
OVCAR-5	0.600	1.637	1.583	1.454	1.285	0.082	0.195	95	82	66	-86	-68	1.27E-6	2.71E-6	5.77E-6
OVCAR-8	0.444	2.285	2.257	2.069	0.557	0.209	0.211	99	88	6	-53	-52	2.92E-7	1.27E-6	8.92E-6
NCI/ADR-RES	0.563	2.045	2.027	1.690	0.786	0.423	0.373	99	76	15	-25	-34	2.67E-7	2.38E-6	> 1.00E-4
Renal Cancer															
786-0	0.921	2.683	2.637	2.463	2.009	0.620	0.588	97	88	62	-33	-36	1.33E-6	4.51E-6	> 1.00E-4
A498	1.553	2.131	2.033	1.966	1.856	0.025	0.020	83	71	52	-98	-99	1.04E-6	2.22E-6	4.77E-6
ACHN	0.354	1.665	1.660	1.144	0.961	0.007	0.144	100	60	46	-98	-59	5.40E-7	2.09E-6	4.65E-6
CAKI-1	0.772	2.668	2.277	1.656	1.502	0.090	0.059	79	47	38	-88	-92	7.88E-8	2.01E-6	4.98E-6
RXF 393	0.883	1.647	1.676	1.609	1.360	0.030	0.039	104	95	62	-97	-96	1.20E-6	2.47E-6	5.09E-6
SN12C	0.558	1.985	1.856	1.780	0.680	0.062	0.082	91	86	9	-89	-85	2.90E-7	1.22E-6	3.98E-6
UO-31	0.542	1.686	1.527	1.397	1.113	0.105	0.021	86	75	50	-81	-96	9.89E-7	2.41E-6	5.82E-6
Prostate Cancer															
PC-3	0.573	1.579	1.457	1.429	0.658	0.438	0.289	88	85	8	-24	-50	2.87E-7	1.83E-6	> 1.00E-4
DU-145	0.410	1.733	1.762	1.408	1.111	0.014	0.064	102	75	53	-97	-85	1.05E-6	2.26E-6	4.88E-6
Breast Cancer															
MCF7	0.467	2.443	2.351	2.207	0.462	0.195	0.164	95	88	-1	-58	-65	2.67E-7	9.70E-7	7.17E-6
MDA-MB-231/ATCC	0.775	1.875	1.787	1.550	0.946	0.655	0.546	92	70	16	-15	-30	2.36E-7	3.16E-6	> 1.00E-4
HS 578T	0.869	1.938	1.839	1.756	1.532	0.714	0.784	91	83	62	-18	-10	1.41E-6	5.97E-6	> 1.00E-4
BT-549	1.031	1.922	1.889	1.802	1.564	0.103	0.382	96	87	60	-90	-63	1.16E-6	2.51E-6	5.41E-6
T-47D	0.723	1.640	1.565	1.215	0.766	0.299	0.328	92	54	5	-59	-55	1.19E-7	1.19E-6	7.30E-6
MDA-MB-468	0.732	1.362	1.324	1.221	0.589	0.081	0.099	94	78	-20	-89	-87	1.92E-7	6.29E-7	2.75E-6

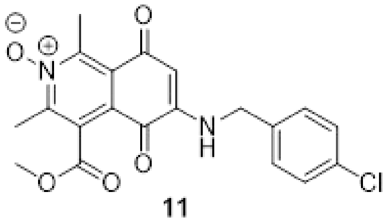

11

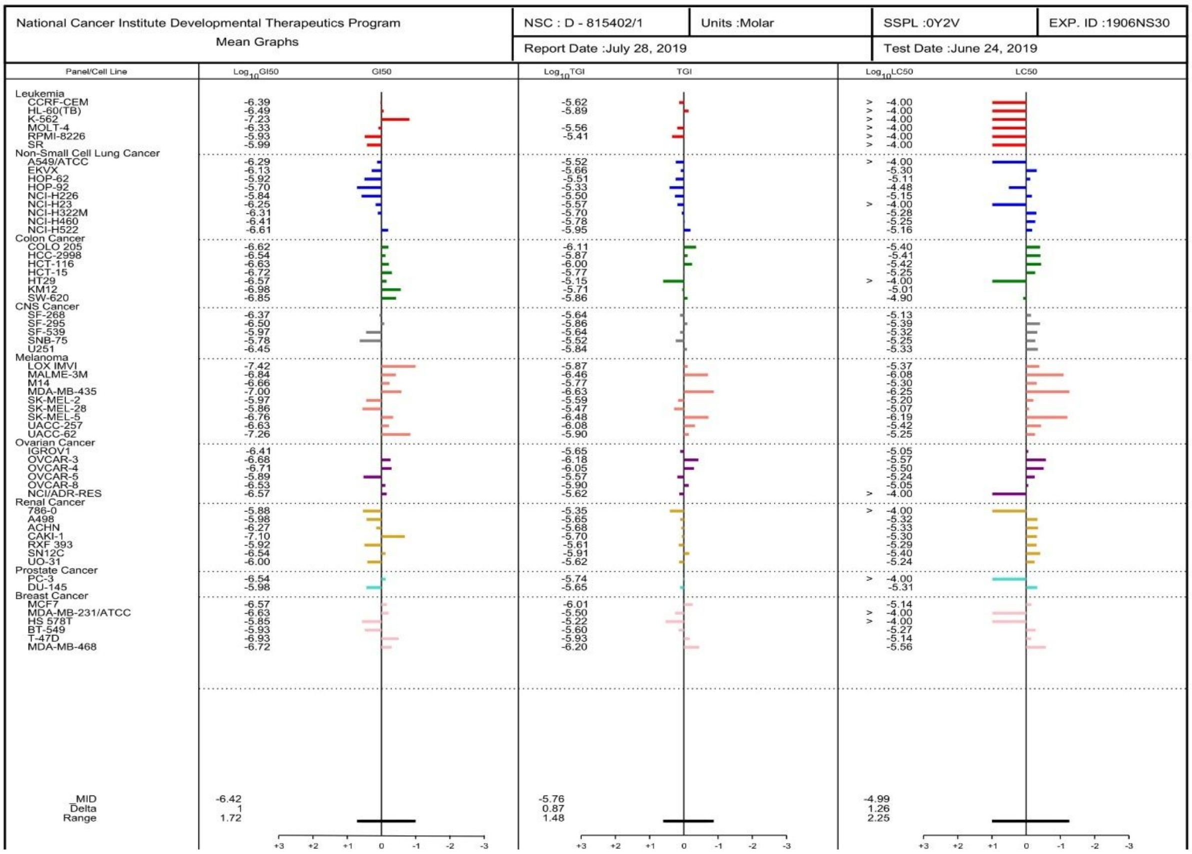

Mean of GI_{50} across 58 cell lines for compound **11** as Log_{10} Concentration (SD): -6.385 (±0.42)

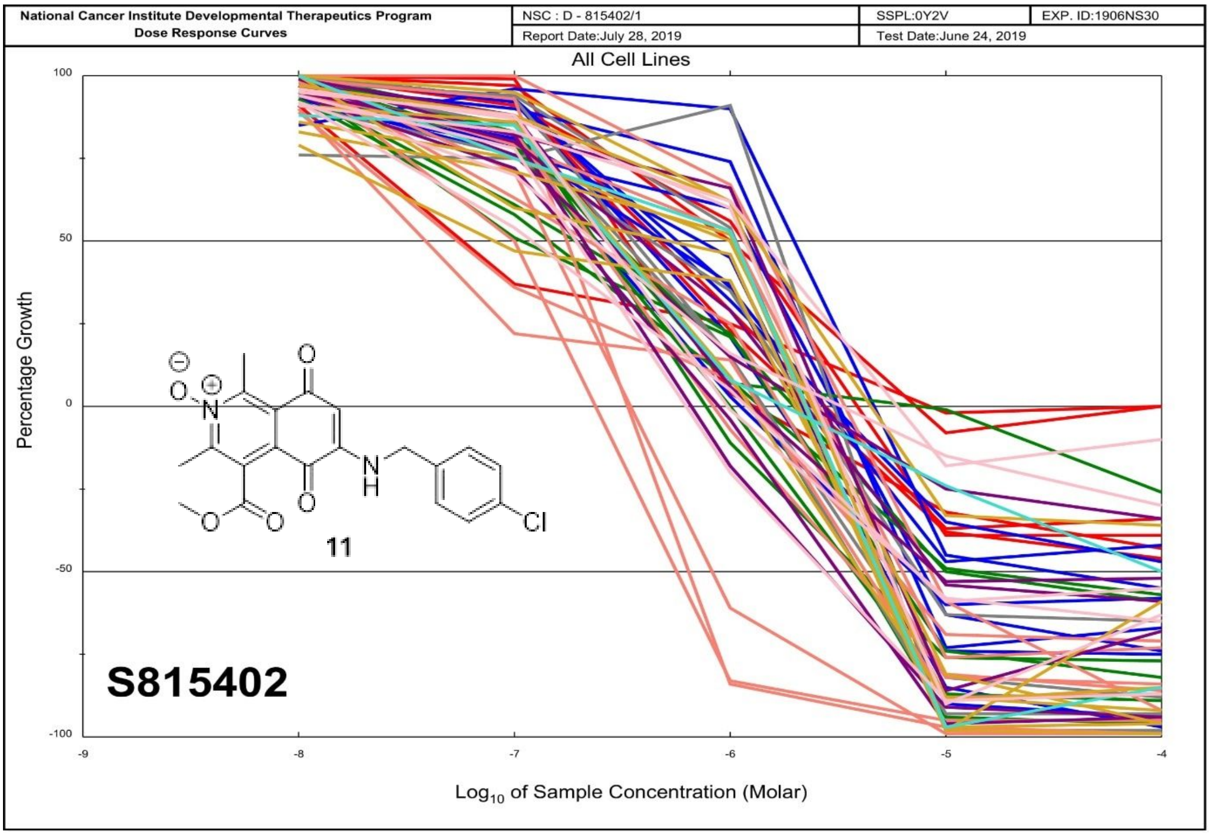

Figure S25 *NCI Five Dose data cell line comparison summary for compound* ***11***

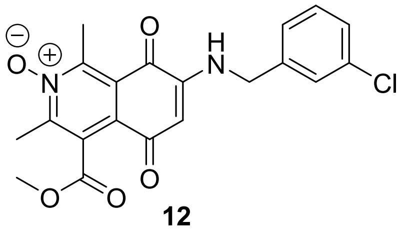

12

Developmental Therapeutics Program One Dose Mean Graph	NSC: D-810560 / 1	Conc: 1.00E-5 Molar	Test Date: Dec 03, 2018
	Experiment ID: 1812OS40		Report Date: Dec 20, 2018

Panel/Cell Line	Growth Percent
Leukemia	
CCRF-CEM	17.55
HL-60(TB)	-56.15
K-562	6.86
MOLT-4	-12.56
RPMI-8226	26.86
SR	9.98
Non-Small Cell Lung Cancer	
A549/ATCC	6.34
EKVX	46.19
HOP-62	41.61
HOP-92	38.00
NCI-H226	79.84
NCI-H23	14.71
NCI-H322M	1.86
NCI-H460	10.12
NCI-H522	-10.36
Colon Cancer	
COLO 205	-50.29
HCC-2998	15.34
HCT-116	1.18
HCT-15	7.55
HT29	5.87
KM12	17.67
SW-620	7.10
CNS Cancer	
SF-268	16.16
SF-295	42.49
SF-539	4.07
SNB-19	9.37
SNB-75	126.89
U251	-6.00
Melanoma	
LOX IMVI	11.69
MALME-3M	-96.21
M14	1.44
MDA-MB-435	-68.50
SK-MEL-2	33.66
SK-MEL-28	63.03
SK-MEL-5	-77.71
UACC-257	-37.53
UACC-62	-42.62
Ovarian Cancer	
IGROV1	2.76
OVCAR-3	-23.91
OVCAR-4	-75.98
OVCAR-5	40.62
OVCAR-8	-5.76
NCI/ADR-RES	0.85
SK-OV-3	81.35
Renal Cancer	
786-0	39.51
A498	105.85
ACHN	26.59
CAKI-1	42.19
RXF 393	34.27
SN12C	0.80
TK-10	73.54
UO-31	50.57
Prostate Cancer	
PC-3	4.17
DU-145	38.29
Breast Cancer	
MCF7	-35.96
MDA-MB-231/ATCC	3.99
HS 578T	79.12
BT-549	49.35
T-47D	6.00
MDA-MB-468	-26.29
Mean	11.96
Delta	108.17
Range	223.10

Mean Growth Percent - Growth Percent

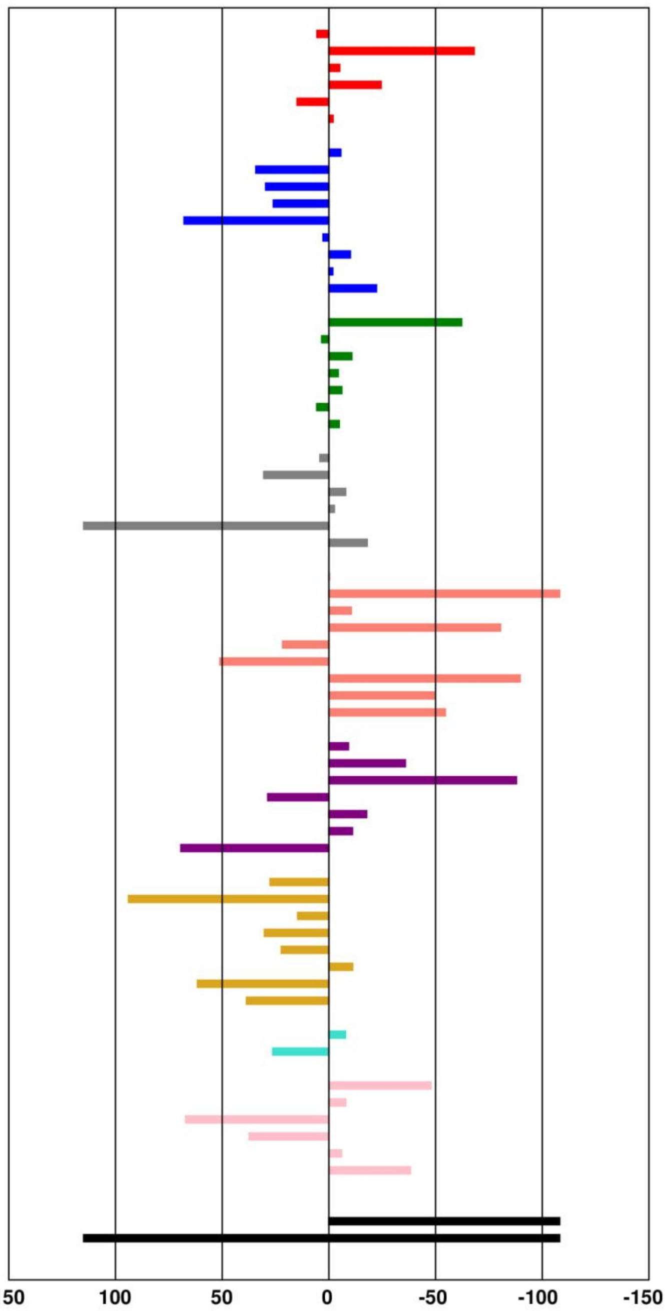

Figure S26 *NCI One Dose (10 µM) data for compound* ***12***

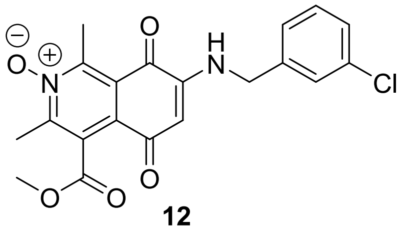

12

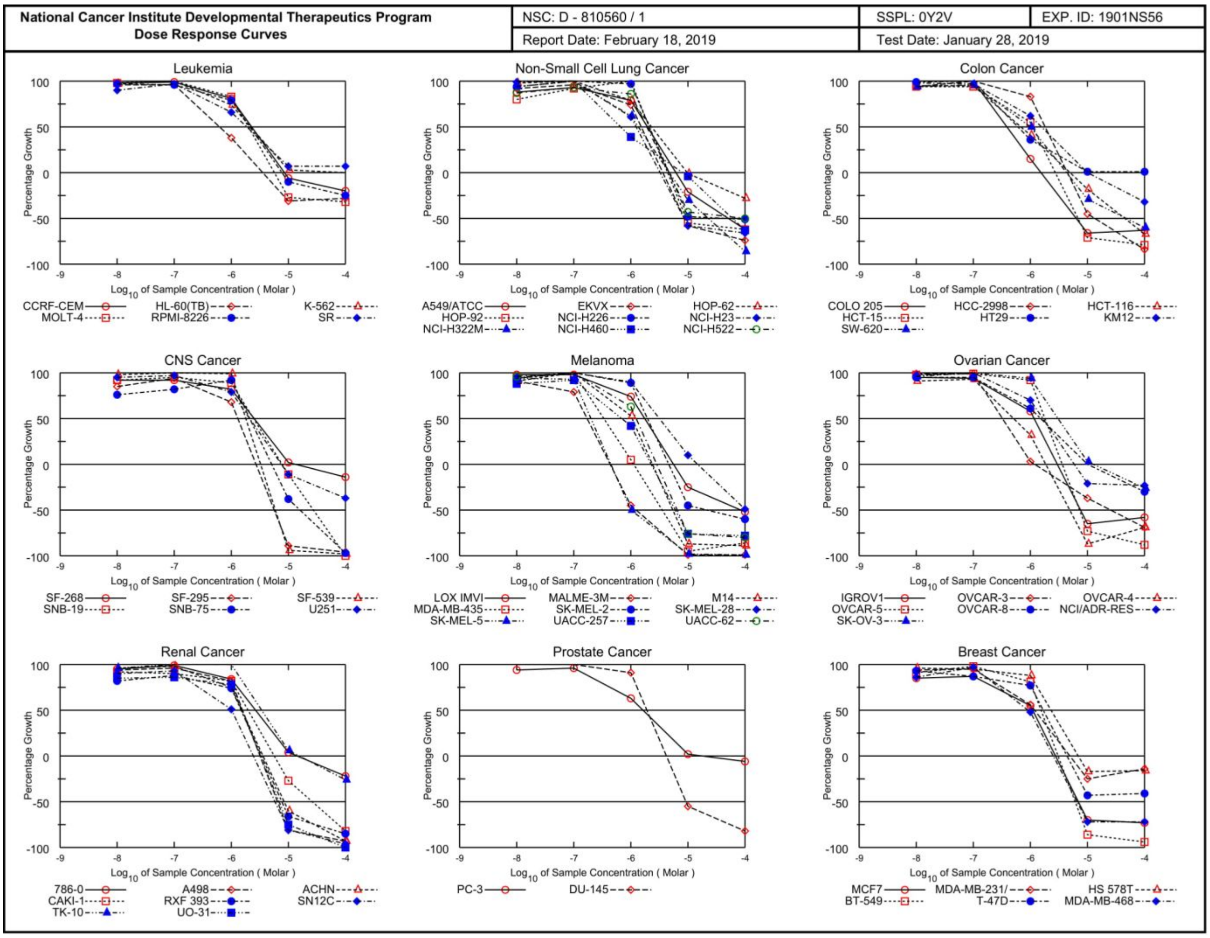

Figure S27 *NCI Five Dose response summary data for compound* ***12***

Table S9** NCI Five Dose raw data for compound **12

National Cancer Institute Developmental Therapeutics Program
In-Vitro Testing Results

NSC : D - 810560 / 1	Experiment ID : 1901NS56	Test Type : 08	Units : Molar
Report Date : February 18, 2019	Test Date : January 28, 2019	QNS :	MC :
COMI : RK 6.4.1	Stain Reagent : SRB Dual-Pass Related	SSPL : 0Y2V	

			Log10 Concentration												
	Time		Mean Optical Densities					Percent Growth							
Panel/Cell Line	Zero	Ctrl	-8.0	-7.0	-6.0	-5.0	-4.0	-8.0	-7.0	-6.0	-5.0	-4.0	GI50	TGI	LC50
Leukemia															
CCRF-CEM	0.580	3.014	2.970	2.979	2.564	0.545	0.464	98	99	81	-6	-20	2.29E-6	8.53E-6	> 1.00E-4
HL-60(TB)	0.877	3.230	3.147	3.140	1.779	0.603	0.631	96	96	38	-31	-28	6.28E-7	3.55E-6	> 1.00E-4
K-562	0.214	2.514	2.438	2.589	1.906	0.293	0.216	97	103	74	3	.	2.17E-6	> 1.00E-4	> 1.00E-4
MOLT-4	0.550	2.839	2.795	2.874	2.461	0.401	0.374	98	102	83	-27	-32	2.01E-6	5.68E-6	> 1.00E-4
RPMI-8226	1.128	3.107	3.042	3.029	2.700	1.021	0.841	97	96	79	-10	-25	2.14E-6	7.81E-6	> 1.00E-4
SR	0.281	1.607	1.468	1.564	1.150	0.375	0.370	90	97	66	7	7	1.84E-6	> 1.00E-4	> 1.00E-4
Non-Small Cell Lung Cancer															
A549/ATCC	0.373	2.454	2.212	2.309	2.012	0.295	0.141	88	93	79	-21	-62	1.94E-6	6.17E-6	5.06E-5
EKVX	0.679	1.948	1.844	1.901	1.616	0.282	0.174	92	96	74	-58	-74	1.51E-6	3.61E-6	8.63E-6
HOP-62	0.451	1.989	1.956	2.001	1.666	0.445	0.324	98	101	79	-1	-28	2.30E-6	9.63E-6	> 1.00E-4
HOP-92	1.205	2.032	1.866	1.969	2.078	0.541	0.457	80	92	106	-55	-62	2.22E-6	4.54E-6	9.29E-6
NCI-H226	1.465	3.027	2.927	3.030	2.973	0.767	0.721	94	100	97	-48	-51	2.10E-6	4.67E-6	5.62E-5
NCI-H23	0.538	1.654	1.645	1.657	1.216	0.226	0.182	99	100	61	-58	-66	1.23E-6	3.25E-6	8.55E-6
NCI-H322M	0.694	2.013	1.934	2.015	1.511	0.487	0.101	94	100	62	-30	-86	1.35E-6	4.73E-6	2.30E-5
NCI-H460	0.284	2.964	2.992	2.955	1.329	0.272	0.105	101	100	39	-4	-63	6.59E-7	7.92E-6	5.99E-5
NCI-H522	0.911	2.772	2.524	2.647	2.518	0.515	0.457	87	93	86	-43	-50	1.91E-6	4.63E-6	> 1.00E-4
Colon Cancer															
COLO 205	0.443	2.093	2.098	2.087	0.696	0.153	0.164	100	100	15	-66	-63	3.88E-7	1.55E-6	6.42E-6
HCC-2998	0.652	2.372	2.265	2.394	2.085	0.359	0.103	94	101	83	-45	-84	1.82E-6	4.46E-6	1.34E-5
HCT-116	0.242	2.474	2.352	2.365	1.155	0.198	0.081	95	95	41	-18	-67	6.79E-7	4.90E-6	4.54E-5
HCT-15	0.355	2.554	2.413	2.433	1.572	0.104	0.076	94	94	55	-71	-79	1.10E-6	2.75E-6	6.85E-6
HT29	0.240	2.030	2.019	2.182	0.877	0.265	0.258	99	108	36	1	1	6.34E-7	> 1.00E-4	> 1.00E-4
KM12	0.467	2.935	2.886	2.864	1.999	0.464	0.316	98	97	62	.	-32	1.56E-6	9.73E-6	> 1.00E-4
SW-620	0.200	1.547	1.464	1.494	0.878	0.142	0.081	94	96	50	-29	-60	1.01E-6	4.31E-6	4.88E-5
CNS Cancer															
SF-268	0.759	2.584	2.438	2.444	2.264	0.792	0.652	92	92	82	2	-14	2.53E-6	1.29E-5	> 1.00E-4
SF-295	0.484	1.713	1.524	1.634	1.322	0.054	0.021	85	94	68	-89	-96	1.31E-6	2.72E-6	5.66E-6
SF-539	0.884	2.724	2.682	2.795	2.711	0.050	0.021	98	104	99	-94	-98	1.80E-6	3.26E-6	5.90E-6
SNB-19	0.749	2.699	2.540	2.600	2.479	0.669	-0.001	92	95	89	-11	-100	2.45E-6	7.80E-6	2.75E-5
SNB-75	0.890	1.797	1.576	1.631	1.725	0.548	0.026	76	82	92	-38	-97	2.10E-6	5.07E-6	1.57E-5
U251	0.256	1.782	1.710	1.731	1.461	0.228	0.161	95	97	79	-11	-37	2.10E-6	7.52E-6	> 1.00E-4
Melanoma															
LOX IMVI	0.406	2.968	2.916	2.909	2.313	0.304	0.195	98	98	74	-25	-52	1.76E-6	5.58E-6	8.36E-5
MALME-3M	0.679	1.771	1.673	1.541	0.372	0.004	0.005	91	79	-45	-99	-99	1.71E-7	4.32E-7	1.22E-6
M14	0.388	1.798	1.664	1.833	1.140	0.051	0.044	91	102	53	-87	-89	1.06E-6	2.40E-6	5.46E-6
MDA-MB-435	0.443	2.306	2.202	2.335	0.544	0.022	0.061	94	102	5	-95	-86	3.44E-7	1.13E-6	3.56E-6
SK-MEL-2	1.315	3.024	2.928	3.021	2.836	0.720	0.529	94	100	89	-45	-60	1.95E-6	4.60E-6	2.11E-5
SK-MEL-28	0.607	2.349	2.262	2.398	2.171	0.790	0.308	95	103	90	10	-49	3.17E-6	1.50E-5	> 1.00E-4
SK-MEL-5	0.752	3.113	2.976	2.944	0.378	0.018	0.007	94	93	-50	-98	-99	2.00E-7	4.48E-7	1.01E-6
UACC-257	1.097	2.795	2.594	2.666	1.815	0.268	0.238	88	92	42	-76	-78	7.01E-7	2.28E-6	6.06E-6
UACC-62	1.057	3.174	3.098	3.173	2.396	0.249	0.209	96	100	63	-76	-80	1.24E-6	2.84E-6	6.47E-6
Ovarian Cancer															
IGROV1	0.358	1.917	1.837	1.821	1.269	0.127	0.150	95	94	58	-65	-58	1.17E-6	2.98E-6	7.60E-6
OVCAR-3	0.547	2.152	2.133	2.132	0.590	0.345	0.172	99	99	3	-37	-69	3.22E-7	1.17E-6	2.58E-5
OVCAR-4	0.555	1.341	1.271	1.290	0.806	0.070	0.174	91	93	32	-87	-69	5.07E-7	1.85E-6	4.86E-6
OVCAR-5	0.528	1.713	1.686	1.703	1.623	0.145	0.066	98	99	92	-73	-88	1.81E-6	3.63E-6	7.30E-6
OVCAR-8	0.418	2.393	2.288	2.293	1.626	0.416	0.293	95	95	61	.	-30	1.52E-6	9.78E-6	> 1.00E-4
NCI/ADR-RES	0.621	2.227	2.181	2.250	1.741	0.488	0.480	97	101	70	-21	-23	1.64E-6	5.81E-6	> 1.00E-4
SK-OV-3	0.927	2.395	2.333	2.404	2.308	0.973	0.688	96	101	94	3	-26	3.05E-6	1.28E-5	> 1.00E-4
Renal Cancer															
786-0	0.623	2.706	2.610	2.675	2.373	0.702	0.483	95	99	84	4	-22	2.65E-6	1.39E-5	> 1.00E-4
A498	1.828	2.877	2.814	2.837	2.683	0.353	0.121	94	96	82	-81	-93	1.56E-6	3.18E-6	6.47E-6
ACHN	0.320	1.710	1.648	1.681	1.356	0.128	0.023	96	98	75	-60	-93	1.52E-6	3.58E-6	8.43E-6
CAKI-1	0.613	2.525	2.373	2.342	2.187	0.445	0.111	92	90	82	-27	-82	1.97E-6	5.63E-6	2.59E-5
RXF 393	1.197	1.759	1.658	1.690	1.612	0.406	0.185	82	88	74	-66	-85	1.48E-6	3.37E-6	7.67E-6
SN12C	0.664	2.694	2.489	2.561	1.707	0.126	0.028	90	93	51	-81	-96	1.02E-6	2.44E-6	5.83E-6
TK-10	0.733	2.215	2.154	2.220	2.328	0.821	0.543	96	100	108	6	-26	3.69E-6	1.53E-5	> 1.00E-4
UO-31	0.630	1.978	1.779	1.784	1.686	0.156	-0.010	85	86	78	-75	-100	1.53E-6	3.23E-6	6.84E-6
Prostate Cancer															
PC-3	0.511	2.393	2.277	2.315	1.690	0.552	0.478	94	96	63	2	-6	1.62E-6	1.79E-5	> 1.00E-4
DU-145	0.346	1.756	1.764	1.817	1.632	0.155	0.061	101	104	91	-55	-82	1.91E-6	4.20E-6	9.21E-6
Breast Cancer															
MCF7	0.309	1.783	1.562	1.597	1.126	0.093	0.083	85	87	55	-70	-73	1.10E-6	2.77E-6	6.94E-6
MDA-MB-231/ATCC	0.656	1.647	1.586	1.595	1.208	0.491	0.563	94	95	56	-25	-14	1.18E-6	4.88E-6	> 1.00E-4
HS 578T	0.915	2.004	1.964	1.948	1.878	0.761	0.769	96	95	88	-17	-16	2.32E-6	6.91E-6	> 1.00E-4
BT-549	1.174	2.578	2.447	2.543	2.313	0.170	0.068	91	98	81	-86	-94	1.54E-6	3.07E-6	6.12E-6
T-47D	1.075	2.835	2.720	2.615	2.438	0.617	0.635	93	87	77	-43	-41	1.69E-6	4.41E-6	> 1.00E-4
MDA-MB-468	0.915	1.614	1.514	1.593	1.248	0.257	0.259	86	97	48	-72	-72	8.96E-7	2.50E-6	6.56E-6

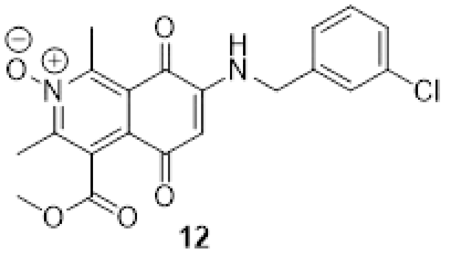

12

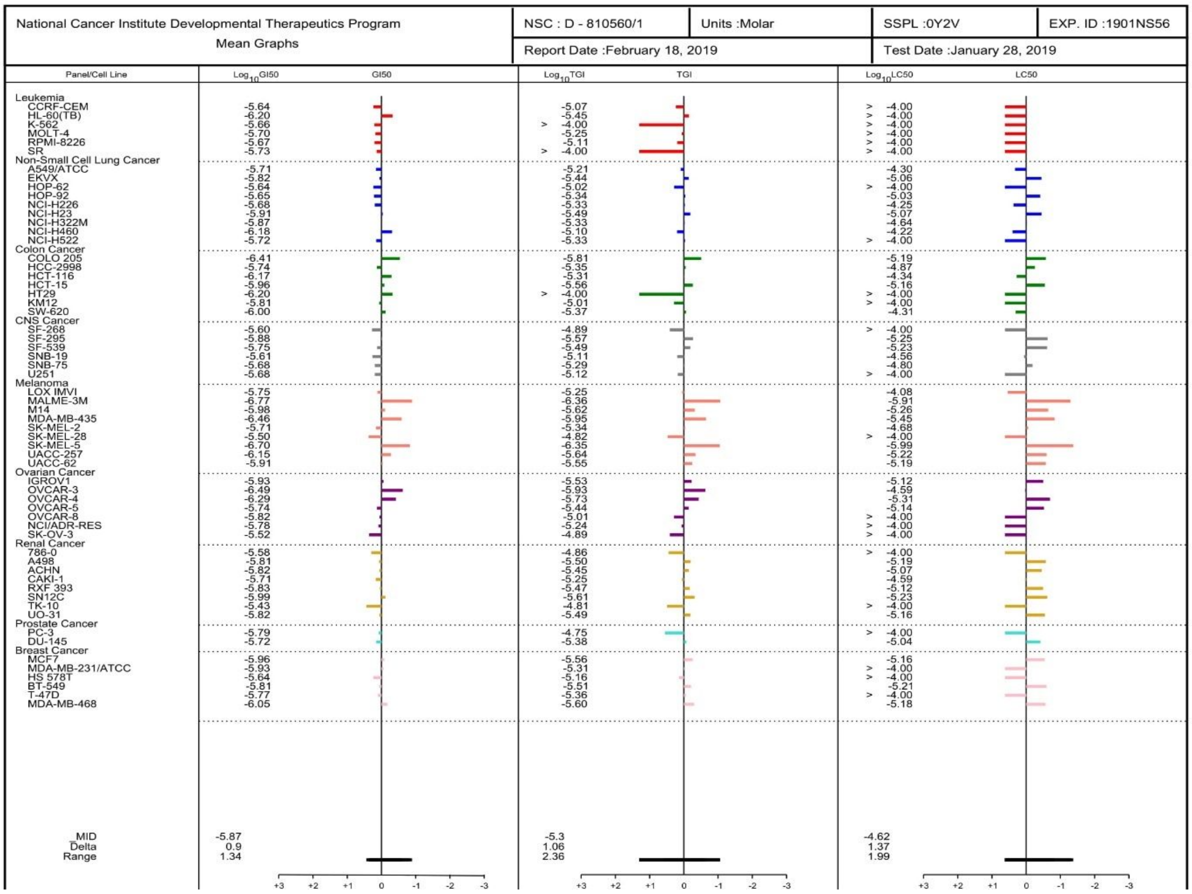

Mean of GI_{50} across 60 cell lines for compound **12** as Log_{10} Concentration (SD): -5.874 (±0.28)

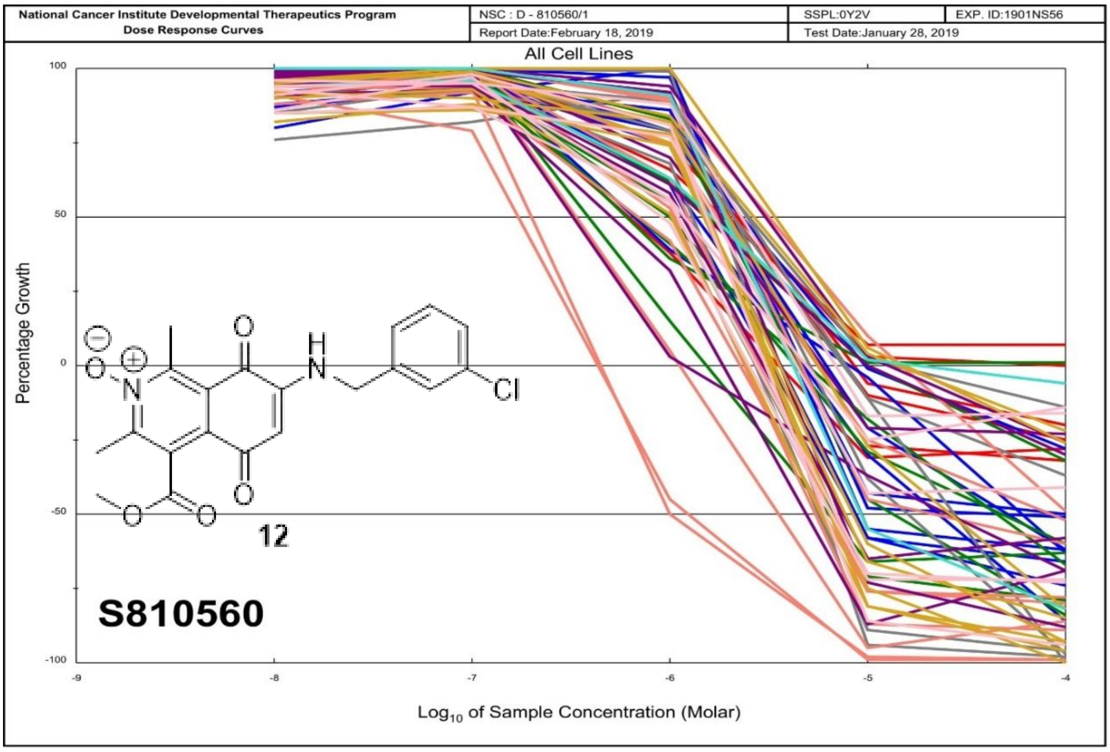

Figure S28 *NCI Five Dose data cell line comparison summary for compound* ***12***

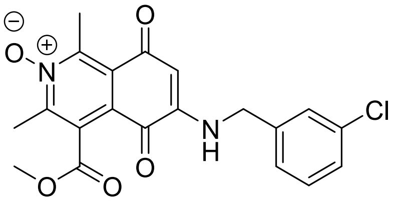

13

Developmental Therapeutics Program One Dose Mean Graph	NSC: D-815403 / 1	Conc: 1.00E-5 Molar	Test Date: May 20, 2019
	Experiment ID: 1905OS11		Report Date: Jun 10, 2019

Panel/Cell Line	Growth Percent
Leukemia	
CCRF-CEM	-11.17
HL-60(TB)	-31.78
K-562	-1.12
MOLT-4	-34.78
RPMI-8226	-31.90
SR	1.95
Non-Small Cell Lung Cancer	
A549/ATCC	-49.46
EKVX	-57.30
HOP-62	-19.77
HOP-92	-32.17
NCI-H23	-62.99
NCI-H322M	-18.33
NCI-H460	-3.61
NCI-H522	-63.48
Colon Cancer	
COLO 205	-69.94
HCC-2998	-38.32
HCT-116	-13.70
HCT-15	-66.94
HT29	3.61
KM12	0.50
SW-620	-31.37
CNS Cancer	
SF-268	-0.35
SF-295	-96.02
SF-539	-91.70
SNB-19	-53.41
SNB-75	-95.41
U251	-20.70
Melanoma	
LOX IMVI	-57.55
MALME-3M	-96.90
M14	-79.55
MDA-MB-435	-92.09
SK-MEL-2	-32.77
SK-MEL-28	-1.27
SK-MEL-5	-97.06
UACC-257	-88.46
UACC-62	-67.97
Ovarian Cancer	
IGROV1	-53.86
OVCAR-3	-35.69
OVCAR-5	-87.19
OVCAR-8	-14.42
NCI/ADR-RES	-12.67
SK-OV-3	-0.95
Renal Cancer	
786-0	6.87
ACHN	-44.79
CAKI-1	-14.33
RXF 393	-32.08
SN12C	-90.67
TK-10	14.48
UO-31	-18.46
Prostate Cancer	
PC-3	-1.70
DU-145	-98.33
Breast Cancer	
MCF7	-61.30
MDA-MB-231/ATCC	-27.26
HS 578T	-23.16
BT-549	-82.25
T-47D	-37.59
MDA-MB-468	-66.28
Mean	-41.84
Delta	56.49
Range	112.81

Mean Growth Percent - Growth Percent

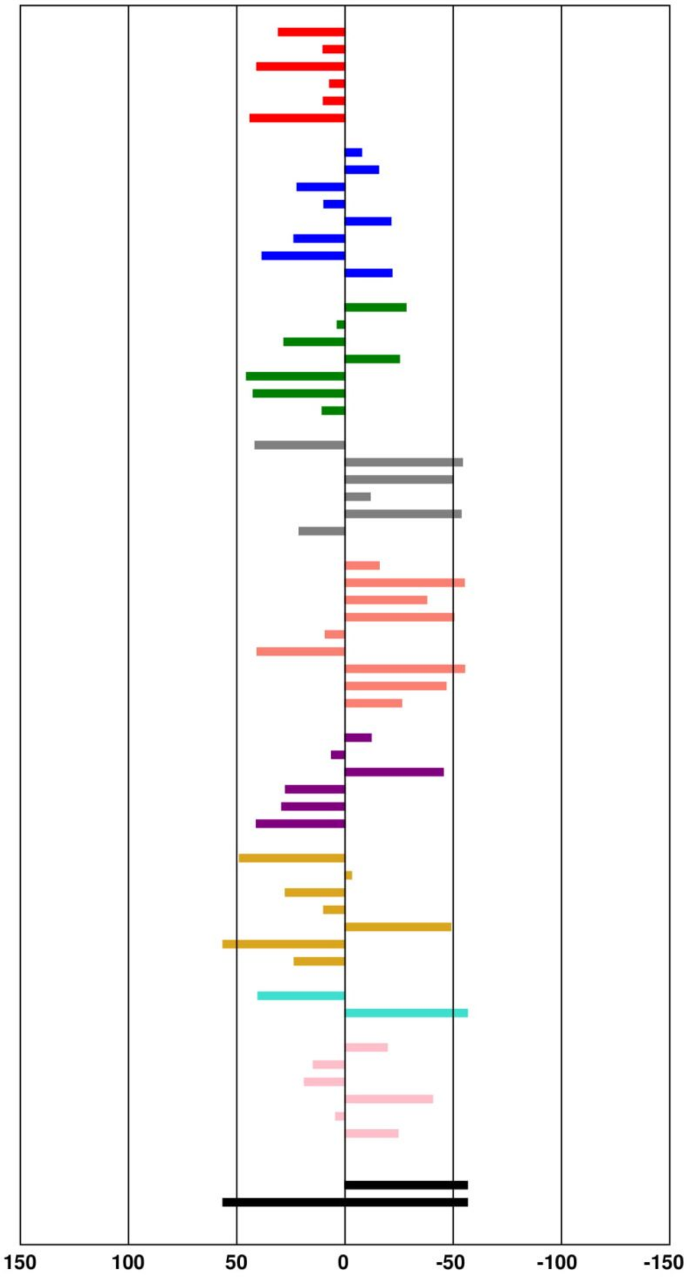

Figure S29** NCI One Dose (10 μM) data for compound **13

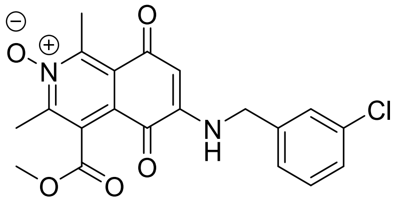

13

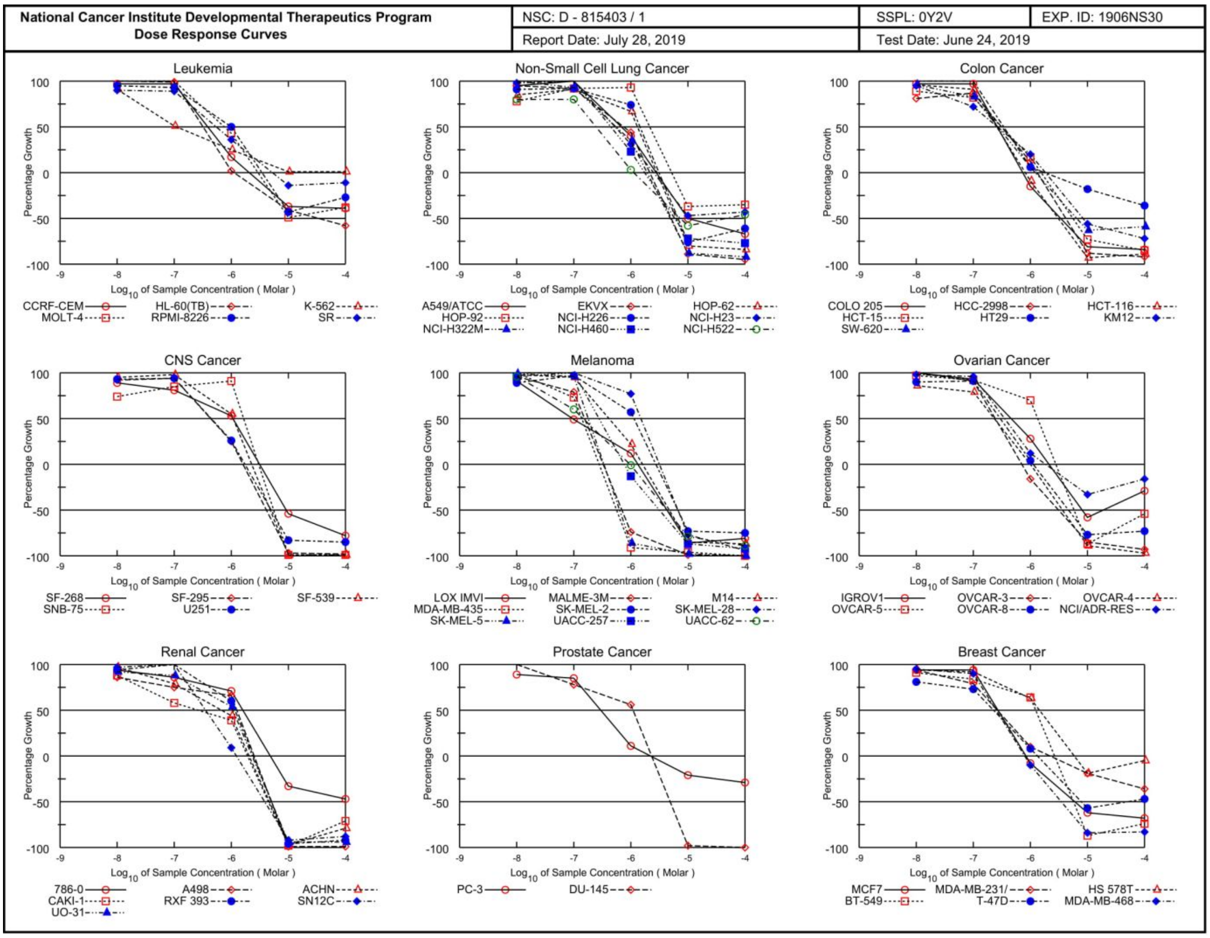

Figure S30 *NCI Five Dose response summary data for compound* ***13***

Table S10 *NCI Five Dose raw data for compound **13***

National Cancer Institute Developmental Therapeutics Program
In-Vitro Testing Results

NSC : D - 815403 / 1	Experiment ID : 1906NS30	Test Type : 08	Units : Molar
Report Date : July 28, 2019	Test Date : June 24, 2019	QNS :	MC :
COMI : RK 6.4.1.1	Stain Reagent : SRB Dual-Pass Related	SSPL : 0Y2V	

Log10 Concentration

	Time		Mean Optical Densities					Percent Growth							
Panel/Cell Line	Zero	Ctrl	-8.0	-7.0	-6.0	-5.0	-4.0	-8.0	-7.0	-6.0	-5.0	-4.0	GI50	TGI	LC50
Leukemia															
CCRF-CEM	0.555	2.292	2.247	2.244	0.852	0.352	0.339	97	97	17	-37	-39	3.88E-7	2.08E-6	> 1.00E-4
HL-60(TB)	0.970	3.231	3.220	3.212	1.018	0.577	0.406	100	99	2	-41	-58	3.21E-7	1.12E-6	3.43E-5
K-562	0.175	1.302	1.206	0.750	0.454	0.185	0.184	91	51	25	1	1	1.09E-7	> 1.00E-4	> 1.00E-4
MOLT-4	0.690	2.644	2.833	2.957	1.539	0.355	0.427	110	116	43	-49	-38	8.13E-7	2.97E-6	> 1.00E-4
RPMI-8226	0.896	2.668	2.573	2.537	1.782	0.514	0.651	95	93	50	-43	-27	9.99E-7	3.46E-6	> 1.00E-4
SR	0.401	1.217	1.131	1.131	0.694	0.346	0.358	90	89	36	-14	-11	5.45E-7	5.27E-6	> 1.00E-4
Non-Small Cell Lung Cancer															
A549/ATCC	0.290	1.839	1.756	1.852	0.911	0.145	0.095	95	101	40	-50	-67	6.86E-7	2.78E-6	1.00E-5
EKVX	0.554	1.801	1.738	1.696	1.108	0.061	0.026	95	92	44	-89	-95	7.62E-7	2.15E-6	5.10E-6
HOP-62	0.711	1.825	1.657	1.720	1.453	0.142	0.117	85	91	67	-80	-84	1.30E-6	2.85E-6	6.24E-6
HOP-92	1.036	1.570	1.452	1.527	1.533	0.654	0.671	78	92	93	-37	-35	2.15E-6	5.20E-6	> 1.00E-4
NCI-H226	1.126	2.489	2.372	2.381	2.140	0.275	0.441	91	92	74	-76	-61	1.45E-6	3.13E-6	6.75E-6
NCI-H23	0.613	1.938	1.907	1.973	1.023	0.328	0.347	98	103	31	-47	-43	5.42E-7	2.51E-6	> 1.00E-4
NCI-H322M	0.743	2.017	2.052	1.924	1.190	0.093	0.059	103	93	35	-88	-92	5.50E-7	1.93E-6	4.94E-6
NCI-H460	0.287	2.898	2.952	2.891	0.894	0.081	0.066	102	100	23	-72	-77	4.47E-7	1.75E-6	5.88E-6
NCI-H522	0.820	2.327	2.023	2.020	0.872	0.342	0.444	80	80	3	-58	-46	2.45E-7	1.14E-6	.
Colon Cancer															
COLO 205	0.585	2.207	2.152	2.164	0.496	0.110	0.094	97	97	-15	-81	-84	2.63E-7	7.33E-7	3.37E-6
HCC-2998	0.932	2.634	2.308	2.412	1.129	0.115	0.071	81	87	12	-88	-92	3.09E-7	1.31E-6	4.17E-6
HCT-116	0.244	1.914	1.856	1.762	0.223	0.017	0.028	96	91	-9	-93	-89	2.58E-7	8.19E-7	3.09E-6
HCT-15	0.278	2.075	1.886	1.756	0.586	0.075	0.041	89	82	17	-73	-85	3.13E-7	1.55E-6	5.55E-6
HT29	0.208	1.618	1.649	1.647	0.292	0.170	0.133	102	102	6	-18	-36	3.48E-7	1.76E-6	> 1.00E-4
KM12	0.579	2.940	2.820	2.287	1.040	0.255	0.160	95	72	20	-56	-72	2.65E-7	1.81E-6	8.32E-6
SW-620	0.258	2.054	1.979	1.746	0.372	0.096	0.105	96	83	6	-63	-59	2.69E-7	1.24E-6	6.53E-6
CNS Cancer															
SF-268	0.838	2.470	2.292	2.160	1.695	0.387	0.188	89	81	53	-54	-78	1.06E-6	3.12E-6	9.19E-6
SF-295	0.781	2.841	2.674	2.719	1.292	0.026	0.016	92	94	25	-97	-98	4.33E-7	1.60E-6	4.13E-6
SF-539	1.054	2.820	2.725	2.780	2.025	0.010	0.007	95	98	55	-99	-99	1.08E-6	2.28E-6	4.80E-6
SNB-75	1.027	1.755	1.566	1.647	1.688	0.011	0.011	74	85	91	-99	-99	1.64E-6	3.01E-6	5.52E-6
U251	0.272	1.521	1.436	1.450	0.596	0.047	0.040	93	94	26	-83	-85	4.45E-7	1.73E-6	5.00E-6
Melanoma															
LOX IMVI	0.496	2.745	2.548	1.594	0.767	0.069	0.097	91	49	12	-86	-81	9.37E-8	1.33E-6	4.29E-6
MALME-3M	0.763	1.612	1.583	1.430	0.197	0.009	0.002	96	79	-74	-99	-100	1.54E-7	3.27E-7	6.94E-7
M14	0.430	1.655	1.630	1.598	0.702	0.063	0.054	98	95	22	-85	-87	4.17E-7	1.61E-6	4.69E-6
MDA-MB-435	0.512	2.494	2.513	1.959	0.045	0.019	0.002	101	73	-91	-96	-100	1.38E-7	2.78E-7	5.61E-7
SK-MEL-2	1.127	2.574	2.417	2.537	1.954	0.303	0.281	89	97	57	-73	-75	1.13E-6	2.75E-6	6.64E-6
SK-MEL-28	0.772	1.994	2.076	2.134	1.713	0.177	0.043	107	111	77	-77	-94	1.50E-6	3.16E-6	6.67E-6
SK-MEL-5	0.784	3.133	3.099	3.028	0.114	0.012	0.004	99	96	-86	-98	-100	1.78E-7	3.37E-7	6.37E-7
UACC-257	0.739	1.807	1.759	1.803	0.646	0.094	0.061	96	100	-13	-87	-92	2.77E-7	7.72E-7	3.16E-6
UACC-62	0.975	2.644	2.585	1.971	0.961	0.201	0.112	96	60	-1	-79	-89	1.44E-7	9.46E-7	4.20E-6
Ovarian Cancer															
IGROV1	0.547	2.188	2.185	2.080	1.004	0.232	0.389	100	93	28	-58	-29	4.60E-7	2.12E-6	.
OVCAR-3	0.469	1.572	1.568	1.477	0.394	0.071	0.034	100	91	-16	-85	-93	2.43E-7	7.08E-7	3.11E-6
OVCAR-4	0.560	1.552	1.411	1.342	0.558	0.060	0.016	86	79	.	-89	-97	2.31E-7	9.90E-7	3.61E-6
OVCAR-5	0.600	1.647	1.612	1.564	1.337	0.077	0.277	97	92	70	-87	-54	1.35E-6	2.80E-6	5.81E-6
OVCAR-8	0.444	2.325	2.133	2.165	0.516	0.102	0.119	90	91	4	-77	-73	2.97E-7	1.11E-6	4.62E-6
NCI/ADR-RES	0.563	2.064	2.031	2.005	0.740	0.380	0.471	98	96	12	-33	-16	3.52E-7	1.84E-6	> 1.00E-4
Renal Cancer															
786-0	0.921	2.660	2.555	2.419	2.161	0.622	0.488	94	86	71	-33	-47	1.60E-6	4.86E-6	> 1.00E-4
A498	1.553	2.116	2.039	1.977	1.925	0.012	0.018	86	75	66	-99	-99	1.25E-6	2.51E-6	5.03E-6
ACHN	0.354	1.698	1.661	1.415	0.941	0.011	0.076	97	79	44	-97	-79	6.61E-7	2.04E-6	4.63E-6
CAKI-1	0.772	2.663	2.441	1.862	1.518	0.017	0.224	88	58	39	-98	-71	2.63E-7	1.94E-6	4.48E-6
RXF 393	0.883	1.651	1.605	1.661	1.347	0.038	0.068	94	101	60	-96	-92	1.17E-6	2.44E-6	5.09E-6
SN12C	0.558	1.950	1.903	1.979	0.679	0.047	0.068	97	102	9	-92	-88	3.61E-7	1.22E-6	3.84E-6
UO-31	0.542	1.659	1.575	1.523	1.142	0.033	0.033	92	88	54	-94	-94	1.06E-6	2.31E-6	5.04E-6
Prostate Cancer															
PC-3	0.573	1.595	1.479	1.440	0.683	0.454	0.407	89	85	11	-21	-29	2.95E-7	2.19E-6	> 1.00E-4
DU-145	0.410	1.745	1.763	1.447	1.156	0.009	-0.002	101	78	56	-98	-100	1.09E-6	2.31E-6	4.89E-6
Breast Cancer															
MCF7	0.467	2.408	2.283	2.297	0.428	0.178	0.149	94	94	-8	-62	-68	2.70E-7	8.29E-7	6.00E-6
MDA-MB-231/ATCC	0.775	1.830	1.769	1.605	0.877	0.627	0.495	94	79	10	-19	-36	2.60E-7	2.16E-6	> 1.00E-4
HS 578T	0.869	1.985	1.922	1.893	1.576	0.704	0.823	94	92	63	-19	-5	1.45E-6	5.87E-6	> 1.00E-4
BT-549	1.031	1.911	1.834	1.769	1.597	0.137	0.266	91	84	64	-87	-74	1.24E-6	2.67E-6	5.71E-6
T-47D	0.723	1.620	1.452	1.376	0.798	0.313	0.381	81	73	8	-57	-47	2.25E-7	1.34E-6	.
MDA-MB-468	0.732	1.340	1.310	1.276	0.661	0.114	0.126	95	90	-10	-84	-83	2.50E-7	7.97E-7	3.46E-6

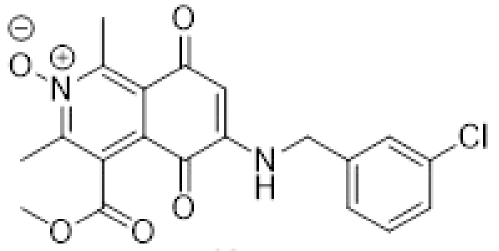

13

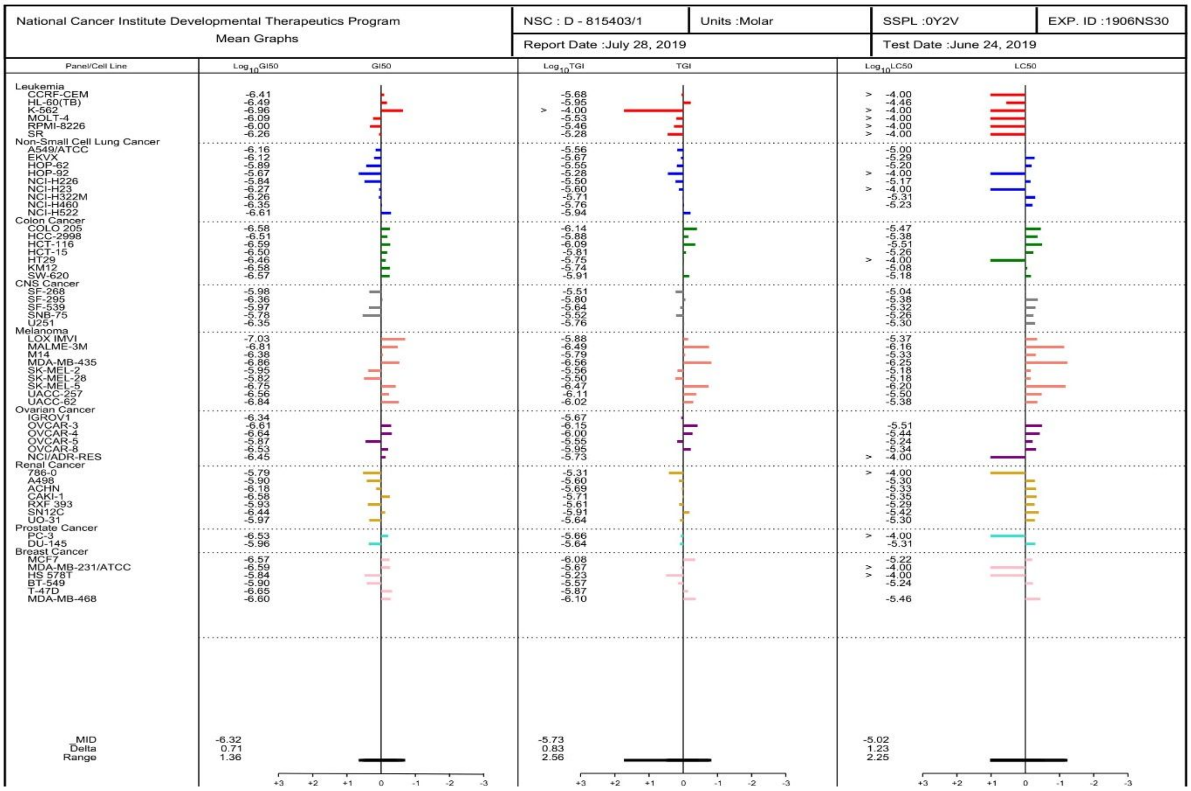

Mean of GI_{50} across 58 cell lines for compound **13** as Log_{10} Concentration (SD): -6.393 (±0.38)

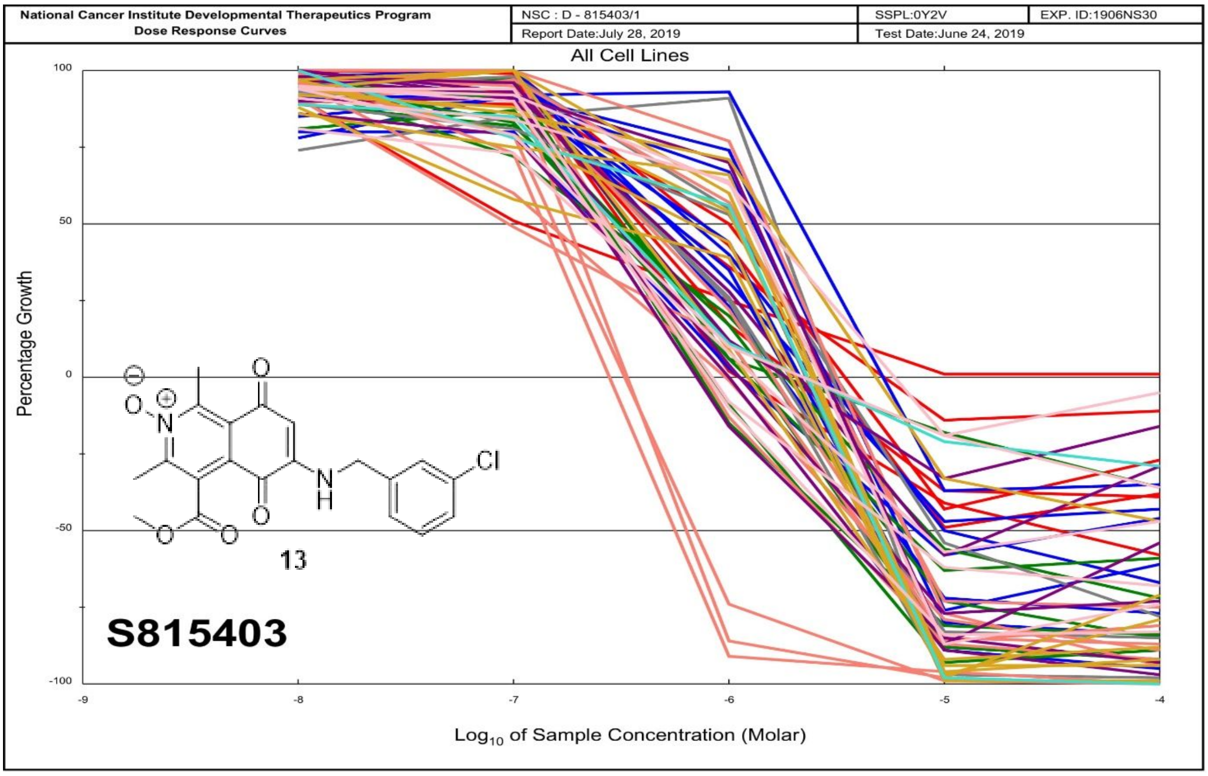

Figure S31** NCI Five Dose data cell line comparison summary for compound **13

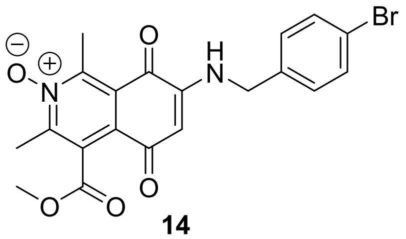

14

Developmental Therapeutics Program One Dose Mean Graph	NSC: D-815369 / 1	Conc: 1.00E-5 Molar	Test Date: May 20, 2019
	Experiment ID: 1905OS11		Report Date: Jun 10, 2019

Panel/Cell Line	Growth Percent
Leukemia	
CCRF-CEM	34.29
HL-60(TB)	-19.89
K-562	32.02
MOLT-4	35.01
RPMI-8226	47.59
SR	30.16
Non-Small Cell Lung Cancer	
A549/ATCC	18.20
EKVX	31.79
HOP-62	31.41
HOP-92	78.14
NCI-H23	11.01
NCI-H322M	28.73
NCI-H460	3.51
NCI-H522	42.84
Colon Cancer	
COLO 205	-5.42
HCC-2998	14.41
HCT-116	1.37
HCT-15	7.94
HT29	9.96
KM12	15.16
SW-620	5.24
CNS Cancer	
SF-268	16.20
SF-295	19.31
SF-539	8.36
SNB-19	19.03
SNB-75	84.51
U251	9.45
Melanoma	
LOX IMVI	16.89
MALME-3M	-70.08
M14	4.19
MDA-MB-435	-77.38
SK-MEL-2	47.06
SK-MEL-28	65.29
SK-MEL-5	-93.07
UACC-257	-52.35
UACC-62	-16.17
Ovarian Cancer	
IGROV1	5.51
OVCAR-3	-21.49
OVCAR-4	-26.96
OVCAR-5	69.08
OVCAR-8	4.52
NCI/ADR-RES	-3.23
SK-OV-3	67.25
Renal Cancer	
786-0	48.79
ACHN	32.61
CAKI-1	46.44
RXF 393	41.38
SN12C	4.82
TK-10	84.36
UO-31	60.88
Prostate Cancer	
PC-3	27.59
DU-145	53.32
Breast Cancer	
MCF7	-20.08
MDA-MB-231/ATCC	4.21
HS 578T	82.37
BT-549	62.27
T-47D	20.48
MDA-MB-468	-16.58
Mean	18.31
Delta	111.38
Range	177.58

Mean Growth Percent - Growth Percent

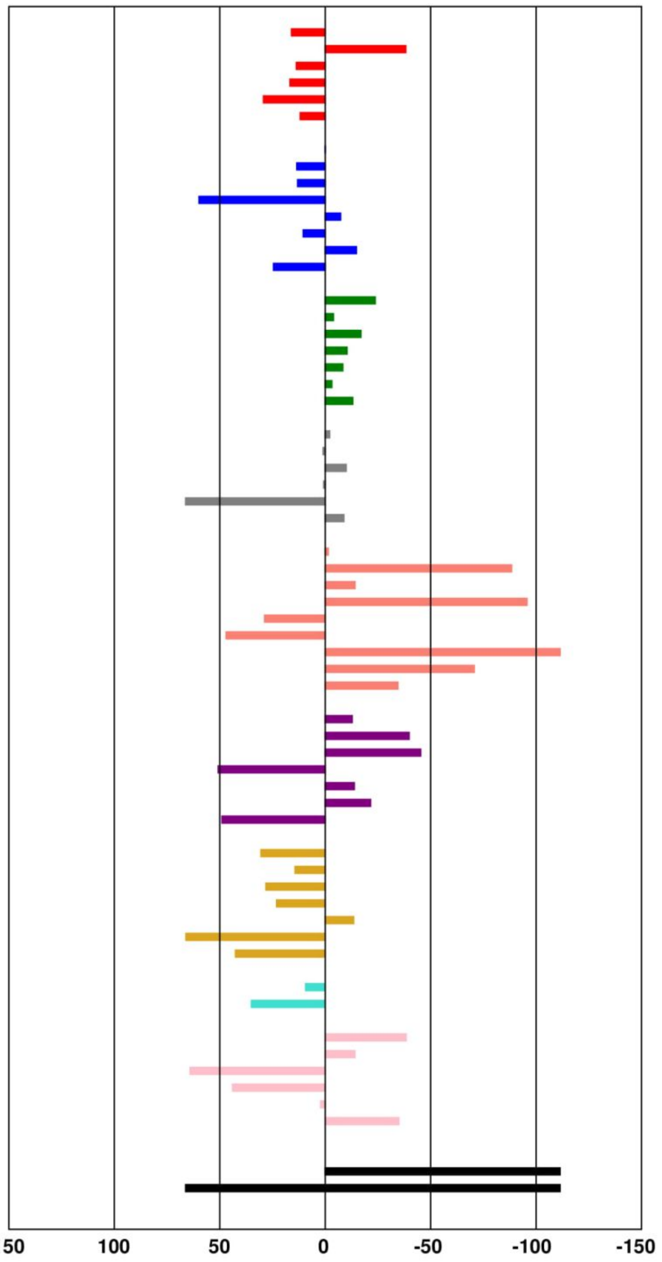

Figure S32 *NCI One Dose (10 µM) data for compound* ***14***

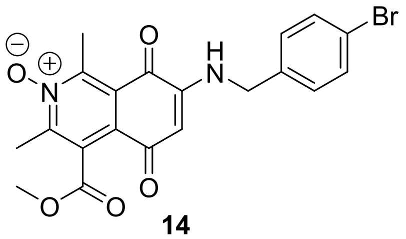

14

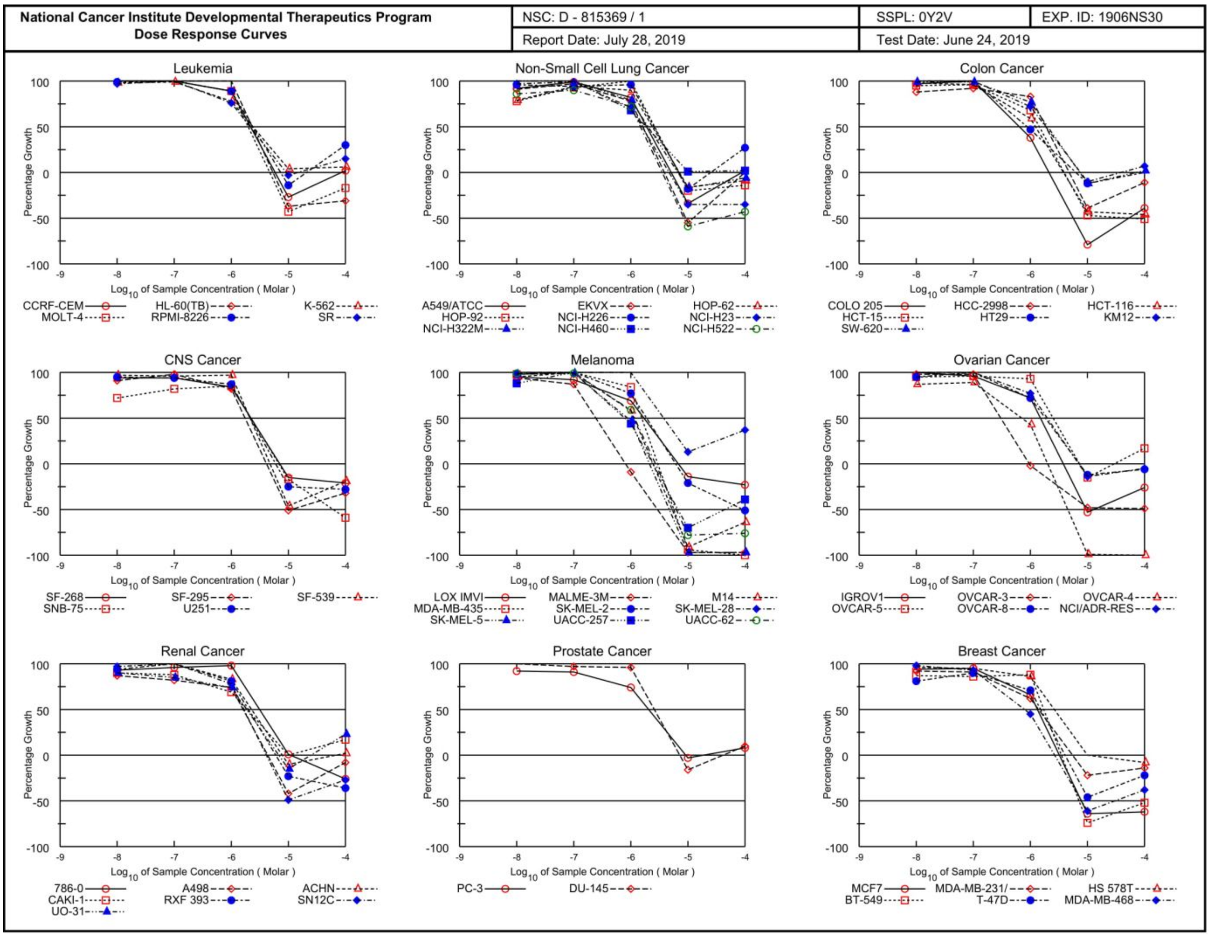

Figure S33 *NCI Five Dose response summary data for compound* ***14***

Table S11 *NCI Five Dose raw data for compound* ***14***

National Cancer Institute Developmental Therapeutics Program
In-Vitro Testing Results

NSC : D - 815369 / 1	Experiment ID : 1906NS30	Test Type : 08	Units : Molar
Report Date : July 28, 2019	Test Date : June 24, 2019	QNS :	MC :
COMI : RK-CC-001	Stain Reagent : SRB Dual-Pass Related	SSPL : 0Y2V	

			Log10 Concentration													
	Time		Mean Optical Densities					Percent Growth							
Panel/Cell Line	Zero	Ctrl	-8.0	-7.0	-6.0	-5.0	-4.0	-8.0	-7.0	-6.0	-5.0	-4.0	GI50	TGI	LC50
Leukemia															
CCRF-CEM	0.555	2.264	2.366	2.450	2.079	0.406	0.595	106	111	89	-27	2	2.18E-6	.	> 1.00E-4
HL-60(TB)	0.970	3.180	3.199	3.180	3.175	0.612	0.672	101	100	100	-37	-31	2.31E-6	5.37E-6	> 1.00E-4
K-562	0.175	1.269	1.248	1.257	1.029	0.216	0.239	98	99	78	4	6	2.38E-6	> 1.00E-4	> 1.00E-4
MOLT-4	0.690	2.569	2.697	2.767	2.358	0.397	0.571	107	111	89	-43	-17	1.97E-6	4.74E-6	> 1.00E-4
RPMI-8226	0.896	2.757	2.740	2.851	2.552	0.774	1.460	99	105	89	-14	30	2.40E-6	.	> 1.00E-4
SR	0.401	1.153	1.133	1.223	0.976	0.387	0.517	97	109	76	-3	15	2.14E-6	.	> 1.00E-4
Non-Small Cell Lung Cancer															
A549/ATCC	0.290	1.761	1.643	1.753	1.503	0.193	0.316	92	99	82	-34	2	1.90E-6	.	> 1.00E-4
EKVX	0.554	1.864	1.747	1.829	1.580	0.247	0.553	91	97	78	-55	.	1.63E-6	3.85E-6	.
HOP-62	0.711	1.759	1.539	1.698	1.653	0.598	0.648	79	94	90	-16	-9	2.38E-6	7.07E-6	> 1.00E-4
HOP-92	1.036	1.574	1.455	1.535	1.662	0.830	0.887	78	93	116	-20	-14	3.07E-6	7.15E-6	> 1.00E-4
NCI-H226	1.126	2.425	2.373	2.342	2.375	0.927	1.474	96	94	96	-18	27	2.54E-6	.	> 1.00E-4
NCI-H23	0.613	1.985	1.947	1.966	1.581	0.400	0.399	97	99	71	-35	-35	1.57E-6	4.68E-6	> 1.00E-4
NCI-H322M	0.743	1.914	1.944	1.928	1.670	0.618	0.700	103	101	79	-17	-6	2.01E-6	6.67E-6	> 1.00E-4
NCI-H460	0.287	2.712	2.779	2.801	1.940	0.301	0.341	103	104	68	1	2	1.86E-6	> 1.00E-4	> 1.00E-4
NCI-H522	0.820	2.178	1.990	2.036	1.798	0.334	0.470	86	90	72	-59	-43	1.47E-6	3.54E-6	.
Colon Cancer															
COLO 205	0.585	2.174	2.126	2.215	1.195	0.122	0.358	97	103	38	-79	-39	6.59E-7	2.12E-6	.
HCC-2998	0.932	2.575	2.382	2.444	2.301	0.573	0.831	88	92	83	-39	-11	1.88E-6	4.83E-6	> 1.00E-4
HCT-116	0.244	2.057	2.020	1.987	1.315	0.138	0.132	98	96	59	-43	-46	1.23E-6	3.77E-6	> 1.00E-4
HCT-15	0.278	2.200	2.097	2.116	1.589	0.148	0.138	95	96	68	-47	-51	1.44E-6	3.91E-6	7.08E-5
HT29	0.208	1.596	1.605	1.730	0.865	0.183	0.212	101	110	47	-12	.	9.06E-7	.	> 1.00E-4
KM12	0.579	2.899	2.895	2.914	2.257	0.522	0.732	100	101	72	-10	7	1.87E-6	.	> 1.00E-4
SW-620	0.258	1.863	1.845	1.841	1.498	0.227	0.283	99	99	77	-12	2	2.02E-6	.	> 1.00E-4
CNS Cancer															
SF-268	0.838	2.348	2.251	2.256	2.105	0.716	0.663	94	94	84	-15	-21	2.21E-6	7.11E-6	> 1.00E-4
SF-295	0.781	2.972	2.784	2.927	2.574	0.385	0.531	91	98	82	-51	-32	1.74E-6	4.14E-6	.
SF-539	1.054	2.816	2.767	2.747	2.764	0.566	0.857	97	96	97	-46	-19	2.13E-6	4.75E-6	> 1.00E-4
SNB-75	1.027	1.724	1.526	1.599	1.619	0.851	0.420	72	82	85	-17	-59	2.20E-6	6.79E-6	6.05E-5
U251	0.272	1.462	1.403	1.387	1.306	0.205	0.196	95	94	87	-25	-28	2.14E-6	6.01E-6	> 1.00E-4
Melanoma															
LOX IMVI	0.496	2.755	2.651	2.577	2.045	0.425	0.381	95	92	69	-14	-23	1.67E-6	6.70E-6	> 1.00E-4
MALME-3M	0.763	1.652	1.602	1.540	0.692	0.023	0.020	94	87	-9	-97	-97	2.43E-7	8.00E-7	2.91E-6
M14	0.430	1.624	1.567	1.711	1.128	0.038	0.155	95	107	58	-91	-64	1.14E-6	2.46E-6	5.31E-6
MDA-MB-435	0.512	2.598	2.565	2.640	2.265	0.029	-0.005	98	102	84	-94	-100	1.55E-6	2.96E-6	5.64E-6
SK-MEL-2	1.127	2.430	2.400	2.489	2.128	0.893	0.549	98	105	77	-21	-51	1.88E-6	6.12E-6	9.07E-5
SK-MEL-28	0.772	1.917	2.044	2.045	2.019	0.925	1.191	111	111	109	13	37	4.13E-6	> 1.00E-4	> 1.00E-4
SK-MEL-5	0.784	3.177	3.140	3.144	1.904	0.022	0.021	98	99	47	-97	-97	8.67E-7	2.11E-6	4.70E-6
UACC-257	0.739	1.811	1.684	1.812	1.209	0.221	0.453	88	100	44	-70	-39	7.77E-7	2.43E-6	.
UACC-62	0.975	2.556	2.533	2.542	1.912	0.210	0.238	99	99	59	-78	-76	1.17E-6	2.69E-6	6.21E-6
Ovarian Cancer															
IGROV1	0.547	2.126	2.130	2.066	1.685	0.257	0.405	100	96	72	-53	-26	1.50E-6	3.76E-6	.
OVCAR-3	0.469	1.539	1.514	1.514	0.460	0.244	0.237	98	98	-2	-48	-49	3.01E-7	9.57E-7	> 1.00E-4
OVCAR-4	0.560	1.447	1.333	1.353	0.941	0.005	-0.015	87	89	43	-99	-100	7.04E-7	2.01E-6	4.51E-6
OVCAR-5	0.600	1.563	1.517	1.522	1.494	0.508	0.767	95	96	93	-15	17	2.49E-6	.	> 1.00E-4
OVCAR-8	0.444	2.236	2.143	2.263	1.737	0.391	0.417	95	101	72	-12	-6	1.83E-6	7.19E-6	> 1.00E-4
NCI/ADR-RES	0.563	2.074	2.076	2.125	1.722	0.485	0.533	100	103	77	-14	-5	1.97E-6	7.03E-6	> 1.00E-4
Renal Cancer															
786-0	0.921	2.701	2.577	2.624	2.661	0.945	0.680	93	96	98	1	-26	3.13E-6	1.12E-5	> 1.00E-4
A498	1.553	2.035	1.971	1.950	1.911	0.900	1.436	87	82	74	-42	-8	1.62E-6	4.35E-6	> 1.00E-4
ACHN	0.354	1.577	1.586	1.611	1.371	0.320	0.382	101	103	83	-10	2	2.27E-6	.	> 1.00E-4
CAKI-1	0.772	2.630	2.438	2.402	2.045	0.768	1.088	90	88	69	.	17	1.85E-6	.	> 1.00E-4
RXF 393	0.883	1.690	1.646	1.713	1.539	0.676	0.570	94	103	81	-23	-36	1.99E-6	5.96E-6	> 1.00E-4
SN12C	0.558	1.886	1.847	1.901	1.595	0.282	0.410	97	101	78	-49	-27	1.66E-6	4.09E-6	> 1.00E-4
UO-31	0.542	1.557	1.450	1.408	1.295	0.461	0.772	90	85	74	-15	23	1.87E-6	.	> 1.00E-4
Prostate Cancer															
PC-3	0.573	1.599	1.512	1.504	1.336	0.556	0.658	92	91	74	-3	8	2.06E-6	.	> 1.00E-4
DU-145	0.410	1.662	1.685	1.626	1.608	0.343	0.532	102	97	96	-16	10	2.55E-6	.	> 1.00E-4
Breast Cancer															
MCF7	0.467	2.480	2.350	2.377	1.794	0.167	0.179	94	95	66	-64	-62	1.33E-6	3.21E-6	7.76E-6
MDA-MB-231/ATCC	0.775	1.785	1.708	1.697	1.406	0.606	0.666	92	91	62	-22	-14	1.41E-6	5.51E-6	> 1.00E-4
HS 578T	0.869	1.906	1.866	1.858	1.766	0.867	0.798	96	95	86	.	-8	2.64E-6	9.94E-6	> 1.00E-4
BT-549	1.031	1.877	1.767	1.755	1.780	0.273	0.499	87	86	88	-74	-52	1.73E-6	3.52E-6	7.15E-6
T-47D	0.723	1.693	1.506	1.593	1.413	0.393	0.563	81	90	71	-46	-22	1.52E-6	4.06E-6	> 1.00E-4
MDA-MB-468	0.732	1.317	1.304	1.274	0.997	0.284	0.456	98	93	45	-61	-38	7.97E-7	2.66E-6	.

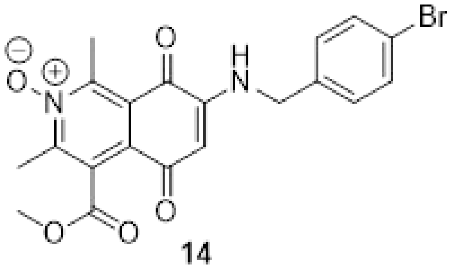

14

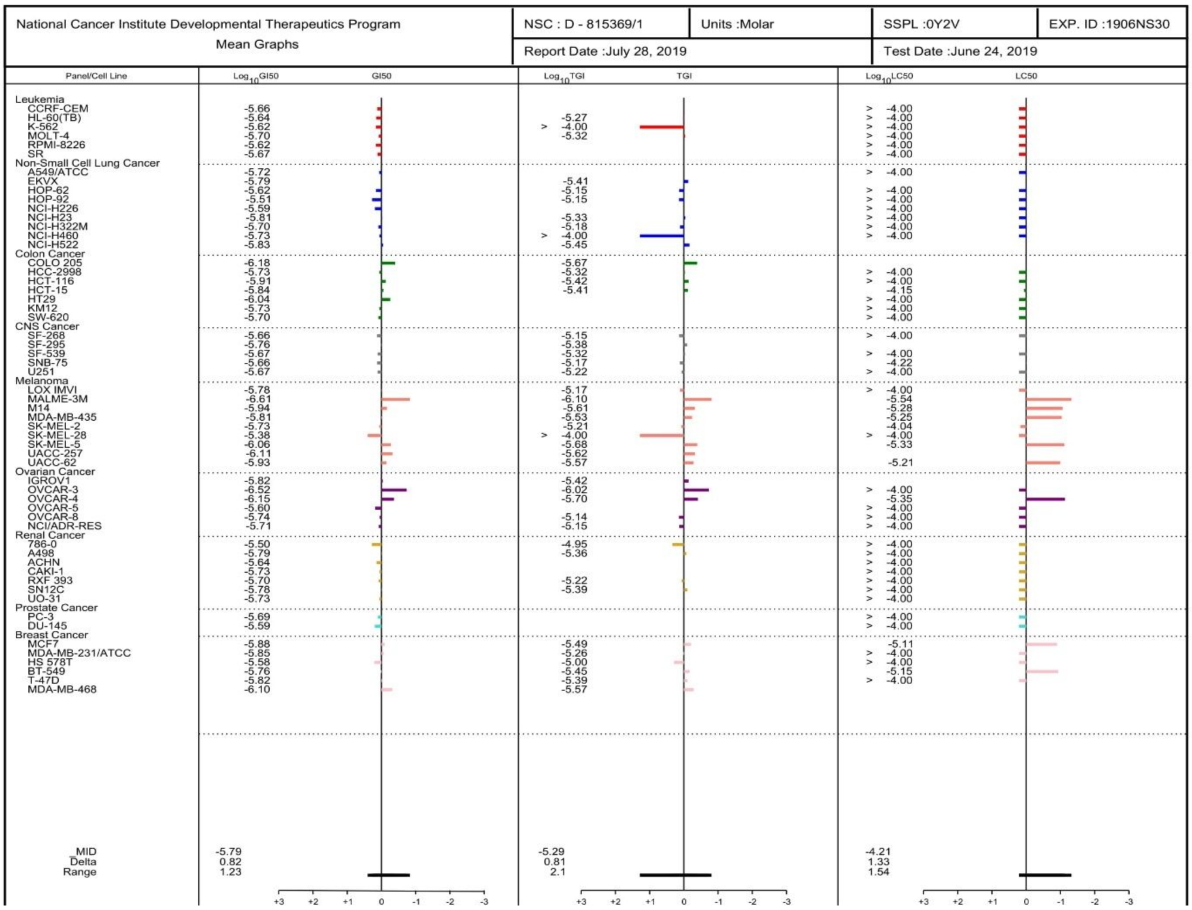

Mean of GI_{50} across 57 cell lines for compound **14** as Log_{10} Concentration (SD): -5.786 (±0.22)

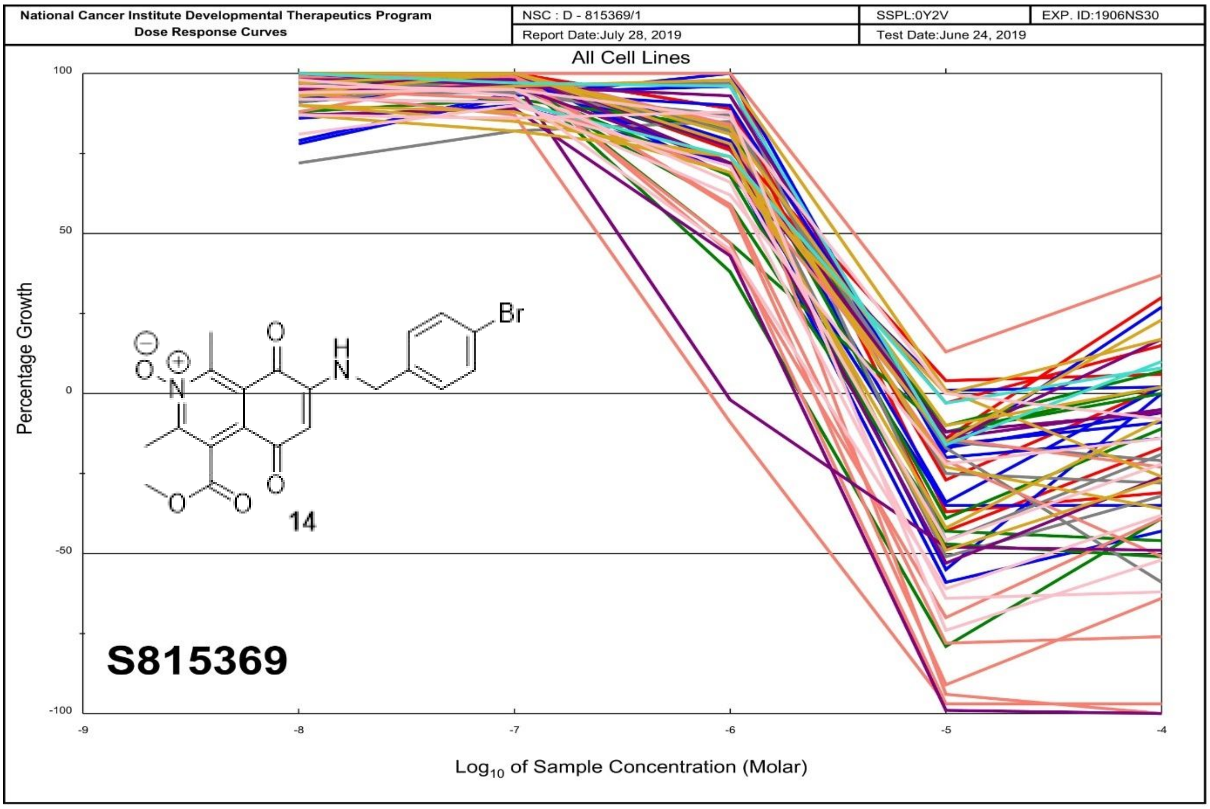

Figure S34 *NCI Five Dose data cell line comparison summary for compound* ***14***

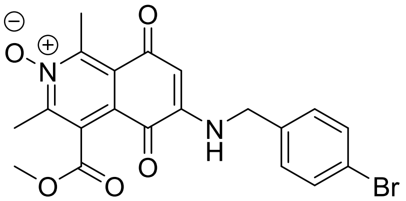

15

Developmental Therapeutics Program
One Dose Mean Graph

NSC: D-820592 / 1 | **Conc:** 1.00E-5 Molar | **Test Date:** Nov 18, 2019

Experiment ID: 1911OS93 | **Report Date:** Dec 19, 2019

Panel/Cell Line	Growth Percent
**Leukemia**	
CCRF-CEM	-12.65
HL-60(TB)	-19.37
K-562	2.51
MOLT-4	-21.69
RPMI-8226	-28.33
SR	1.30
**Non-Small Cell Lung Cancer**	
A549/ATCC	-28.21
EKVX	-85.28
HOP-62	-77.22
HOP-92	-53.85
NCI-H226	-42.88
NCI-H23	-44.82
NCI-H322M	-14.87
NCI-H460	-53.76
NCI-H522	-44.80
**Colon Cancer**	
COLO 205	-69.77
HCT-116	-17.24
HCT-15	-63.01
HT29	-5.46
KM12	-11.31
SW-620	-17.18
**CNS Cancer**	
SF-268	-43.70
SF-295	-97.89
SF-539	-86.81
SNB-19	-87.21
SNB-75	-98.61
U251	-41.00
**Melanoma**	
LOX IMVI	-75.67
MALME-3M	-97.86
M14	-83.87
MDA-MB-435	-92.80
SK-MEL-2	-54.01
SK-MEL-28	-39.61
SK-MEL-5	-97.53
UACC-257	-80.62
UACC-62	-59.45
**Ovarian Cancer**	
IGROV1	-53.40
OVCAR-3	-73.40
OVCAR-4	-92.77
OVCAR-5	-82.79
OVCAR-8	1.05
NCI/ADR-RES	-8.51
SK-OV-3	-10.69
**Renal Cancer**	
786-0	-8.26
A498	-86.37
ACHN	-73.17
CAKI-1	-25.66
RXF 393	-46.11
SN12C	-77.68
TK-10	2.50
UO-31	-85.79
**Prostate Cancer**	
PC-3	-11.06
DU-145	-87.66
**Breast Cancer**	
MCF7	-56.88
MDA-MB-231/ATCC	-4.67
HS 578T	-7.62
BT-549	-93.46
T-47D	-42.80
MDA-MB-468	-60.08
Mean	-49.66
Delta	48.95
Range	101.12

Mean Growth Percent - Growth Percent

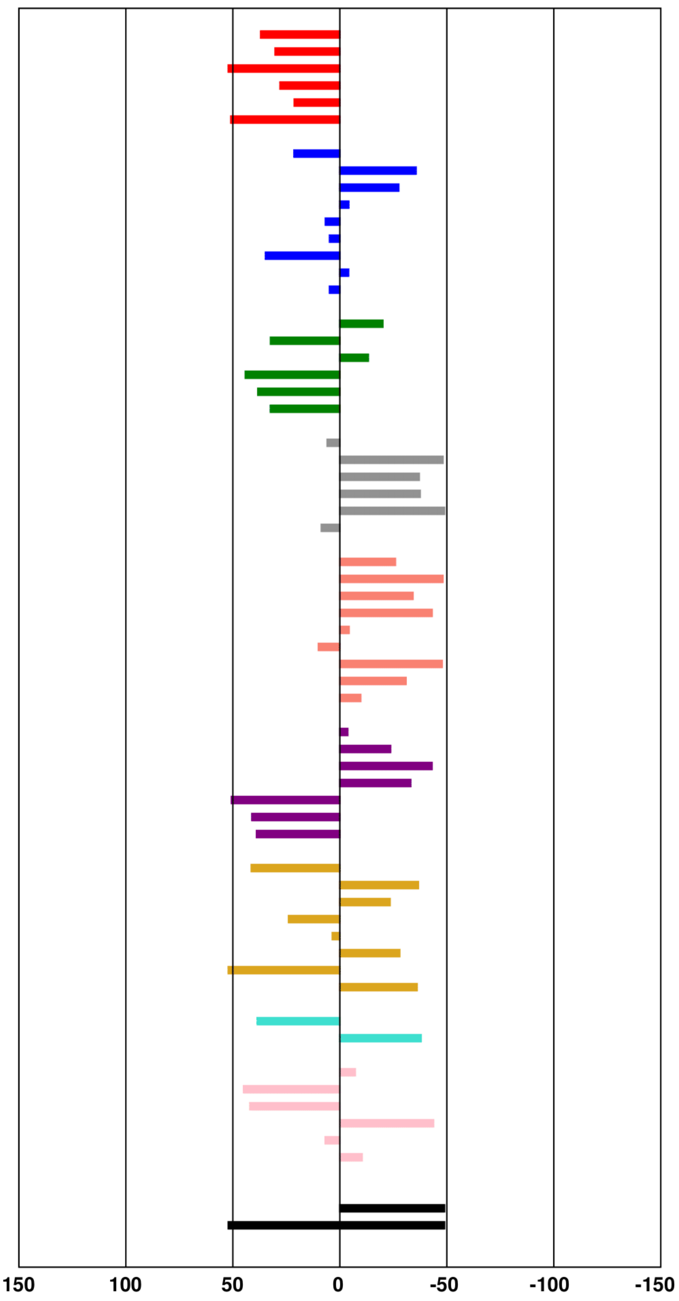

Figure S35 *NCI One Dose (10 µM) data for compound* ***15***

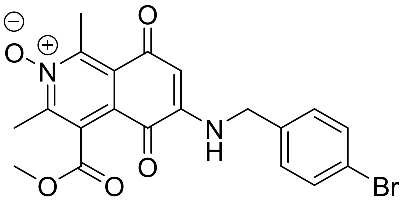

15

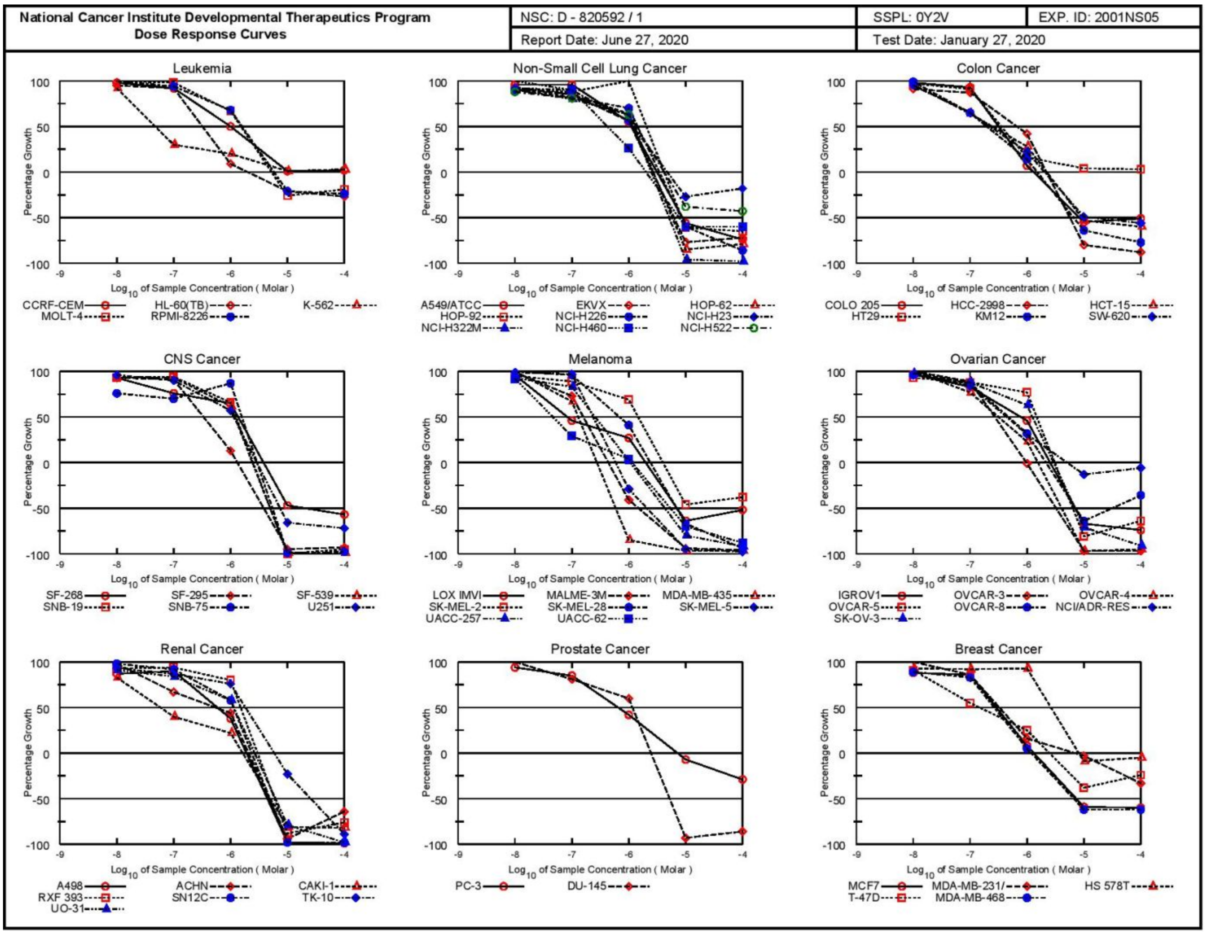

Figure S36 *NCI Five Dose response summary data for compound* ***15***

Table S12** NCI Five Dose raw data for compound **15

National Cancer Institute Developmental Therapeutics Program
In-Vitro Testing Results

NSC : D - 820592 / 1	Experiment ID : 2001NS05	Test Type : 08	Units : Molar
Report Date : June 27, 2020	Test Date : January 27, 2020	QNS :	MC :
COMI : RK 6.8.3.1	Stain Reagent : SRB Dual-Pass Related	SSPL : 0Y2V	

			Log10 Concentration													
	Time		Mean Optical Densities					Percent Growth								
Panel/Cell Line	Zero	Ctrl	-8.0	-7.0	-6.0	-5.0	-4.0	-8.0	-7.0	-6.0	-5.0	-4.0	GI50		TGI	LC50
Leukemia																
CCRF-CEM	0.435	2.141	2.107	2.009	1.287	0.461	0.472	98	92	50	1	2	9.95E-7		> 1.00E-4	> 1.00E-4
HL-60(TB)	0.661	2.906	2.783	2.737	0.865	0.520	0.482	95	92	9	-21	-27	3.23E-7		1.99E-6	> 1.00E-4
K-562	0.196	1.756	1.626	0.662	0.512	0.207	0.236	92	30	20	1	3	4.72E-8		> 1.00E-4	> 1.00E-4
MOLT-4	0.835	3.203	3.232	3.157	2.423	0.619	0.681	101	98	67	-26	-19	1.53E-6		5.27E-6	> 1.00E-4
RPMI-8226	0.857	2.375	2.377	2.284	1.894	0.680	0.653	100	94	68	-21	-24	1.61E-6		5.86E-6	> 1.00E-4
Non-Small Cell Lung Cancer																
A549/ATCC	0.949	3.074	2.980	2.981	2.114	0.421	0.248	96	96	55	-56	-74	1.11E-6		3.14E-6	8.89E-6
EKVX	0.899	2.107	2.024	1.929	1.571	0.203	0.248	93	85	56	-77	-72	1.10E-6		2.62E-6	6.22E-6
HOP-62	0.823	2.671	2.500	2.396	2.005	0.124	0.173	91	85	64	-85	-79	1.24E-6		2.69E-6	5.83E-6
HOP-92	1.421	2.131	2.059	2.046	2.174	0.553	0.499	90	88	106	-61	-65	2.17E-6		4.31E-6	8.58E-6
NCI-H226	0.750	1.807	1.700	1.613	1.487	0.301	0.102	90	82	70	-60	-86	1.42E-6		3.45E-6	8.39E-6
NCI-H23	0.833	2.329	2.207	2.179	1.668	0.612	0.686	92	90	56	-27	-18	1.18E-6		4.76E-6	> 1.00E-4
NCI-H322M	0.923	2.453	2.303	2.150	1.894	0.035	0.017	90	80	63	-96	-98	1.21E-6		2.50E-6	5.14E-6
NCI-H460	0.258	2.844	2.849	2.589	0.928	0.103	0.103	100	90	26	-60	-60	4.22E-7		2.00E-6	7.63E-6
NCI-H522	1.079	2.978	2.741	2.614	2.271	0.668	0.618	88	81	63	-38	-43	1.34E-6		4.19E-6	> 1.00E-4
Colon Cancer																
COLO 205	0.655	2.961	2.907	2.795	0.805	0.298	0.324	98	93	7	-55	-51	3.13E-7		1.28E-6	8.41E-6
HCC-2998	0.527	2.146	1.996	1.931	1.199	0.107	0.065	91	87	42	-80	-88	6.49E-7		2.20E-6	5.68E-6
HCT-15	0.373	2.524	2.433	1.755	0.986	0.175	0.149	96	64	28	-53	-60	2.50E-7		2.23E-6	9.13E-6
HT29	0.439	2.842	2.760	2.636	0.819	0.526	0.508	97	91	16	4	3	3.53E-7		> 1.00E-4	> 1.00E-4
KM12	0.853	3.338	3.301	2.460	1.189	0.304	0.198	99	65	14	-64	-77	1.93E-7		1.49E-6	6.53E-6
SW-620	0.336	2.353	2.252	1.655	0.778	0.172	0.147	95	65	22	-49	-56	2.26E-7		2.04E-6	1.38E-5
CNS Cancer																
SF-268	1.051	2.870	2.740	2.431	2.230	0.559	0.452	93	76	65	-47	-57	1.36E-6		3.81E-6	2.05E-5
SF-295	0.766	3.131	2.976	2.926	1.078	0.042	0.050	93	91	13	-95	-93	3.38E-7		1.33E-6	3.86E-6
SF-539	0.914	2.748	2.644	2.611	2.044	0.007	0.009	94	93	62	-99	-99	1.18E-6		2.42E-6	4.94E-6
SNB-19	0.621	2.205	2.096	2.108	1.659	-0.001	0.031	93	94	66	-100	-95	1.24E-6		2.49E-6	4.99E-6
SNB-75	1.678	2.535	2.329	2.278	2.425	0.021	0.027	76	70	87	-99	-98	1.58E-6		2.94E-6	5.47E-6
U251	0.659	2.835	2.745	2.607	1.895	0.222	0.187	96	90	57	-66	-72	1.14E-6		2.89E-6	7.36E-6
Melanoma																
LOX IMVI	0.438	3.103	2.997	1.669	1.156	0.157	0.210	96	46	27	-64	-52	8.38E-8		1.97E-6	6.97E-6
MALME-3M	0.917	2.165	2.156	1.833	0.542	0.055	0.038	99	73	-41	-94	-96	1.60E-7		4.39E-7	1.48E-6
MDA-MB-435	0.874	3.312	3.327	2.518	0.133	0.026	0.028	101	67	-85	-97	-97	1.30E-7		2.77E-7	5.91E-7
SK-MEL-2	1.256	2.950	2.857	2.757	2.418	0.678	0.773	95	89	69	-46	-38	1.45E-6		3.97E-6	> 1.00E-4
SK-MEL-28	0.831	2.575	2.572	2.507	1.544	0.277	0.054	100	96	41	-67	-94	6.84E-7		2.40E-6	6.99E-6
SK-MEL-5	0.771	3.316	3.303	3.248	0.549	0.040	0.013	99	97	-29	-95	-98	2.37E-7		5.91E-7	2.09E-6
UACC-257	1.224	2.827	2.710	2.555	1.261	0.240	0.098	93	83	2	-80	-92	2.56E-7		1.07E-6	4.29E-6
UACC-62	0.988	2.862	2.718	1.529	1.072	0.301	0.119	92	29	4	-70	-88	4.64E-8		1.15E-6	5.44E-6
Ovarian Cancer																
IGROV1	0.506	2.188	2.130	1.910	1.275	0.170	0.131	97	83	46	-67	-74	7.68E-7		2.55E-6	7.13E-6
OVCAR-3	0.877	2.577	2.575	2.397	0.868	0.024	0.028	100	89	-1	-97	-97	2.73E-7		9.73E-7	3.22E-6
OVCAR-4	1.006	2.211	2.225	1.938	1.281	0.031	0.049	101	77	23	-97	-95	3.17E-7		1.55E-6	4.05E-6
OVCAR-5	0.431	1.421	1.356	1.298	1.189	0.083	0.157	93	88	77	-81	-64	1.48E-6		3.07E-6	6.38E-6
OVCAR-8	0.600	2.657	2.568	2.388	1.251	0.214	0.384	96	87	32	-64	-36	4.65E-7		2.13E-6	.
NCI/ADR-RES	0.576	2.180	2.194	1.922	1.076	0.502	0.540	101	84	31	-13	-6	4.39E-7		5.09E-6	> 1.00E-4
SK-OV-3	0.951	2.668	2.627	2.462	2.041	0.281	0.085	98	88	63	-71	-91	1.26E-6		2.98E-6	7.03E-6
Renal Cancer																
A498	2.141	2.911	2.813	2.836	2.436	0.052	0.026	87	90	38	-98	-99	5.95E-7		1.91E-6	4.46E-6
ACHN	0.327	1.644	1.617	1.215	0.913	0.021	0.118	98	67	44	-94	-64	5.75E-7		2.10E-6	4.83E-6
CAKI-1	1.085	2.994	2.674	1.857	1.507	0.206	0.195	83	40	22	-81	-82	5.97E-8		1.64E-6	5.00E-6
RXF 393	1.369	2.282	2.214	2.227	2.101	0.169	0.332	93	94	80	-88	-76	1.51E-6		3.00E-6	5.96E-6
SN12C	0.489	2.289	2.248	2.147	1.537	0.009	0.010	98	92	58	-98	-98	1.13E-6		2.36E-6	4.92E-6
TK-10	0.899	2.252	2.161	2.074	1.923	0.696	0.100	93	87	76	-23	-89	1.82E-6		5.89E-6	2.59E-5
UO-31	0.612	1.823	1.718	1.634	1.320	0.126	0.010	91	84	58	-79	-98	1.15E-6		2.65E-6	6.12E-6
Prostate Cancer																
PC-3	0.753	2.635	2.525	2.348	1.548	0.701	0.537	94	85	42	-7	-29	6.57E-7		7.22E-6	> 1.00E-4
DU-145	0.418	1.813	1.837	1.551	1.257	0.030	0.059	102	81	60	-93	-86	1.16E-6		2.47E-6	5.25E-6
Breast Cancer																
MCF7	0.732	2.704	2.476	2.423	0.894	0.303	0.296	88	86	8	-59	-60	2.89E-7		1.33E-6	7.43E-6
MDA-MB-231/ATCC	0.607	1.657	1.659	1.514	0.779	0.591	0.410	100	86	16	-3	-33	3.31E-7		7.27E-6	> 1.00E-4
HS 578T	1.654	2.825	2.744	2.737	2.740	1.513	1.567	93	92	93	-9	-5	2.64E-6		8.23E-6	> 1.00E-4
T-47D	0.914	2.508	2.360	1.795	1.313	0.565	0.695	91	55	25	-38	-24	1.49E-7		2.49E-6	> 1.00E-4
MDA-MB-468	0.960	2.228	2.086	2.016	1.019	0.361	0.369	89	83	5	-62	-62	2.65E-7		1.17E-6	6.53E-6

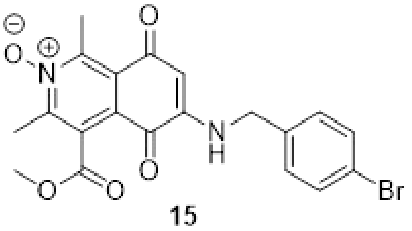

15

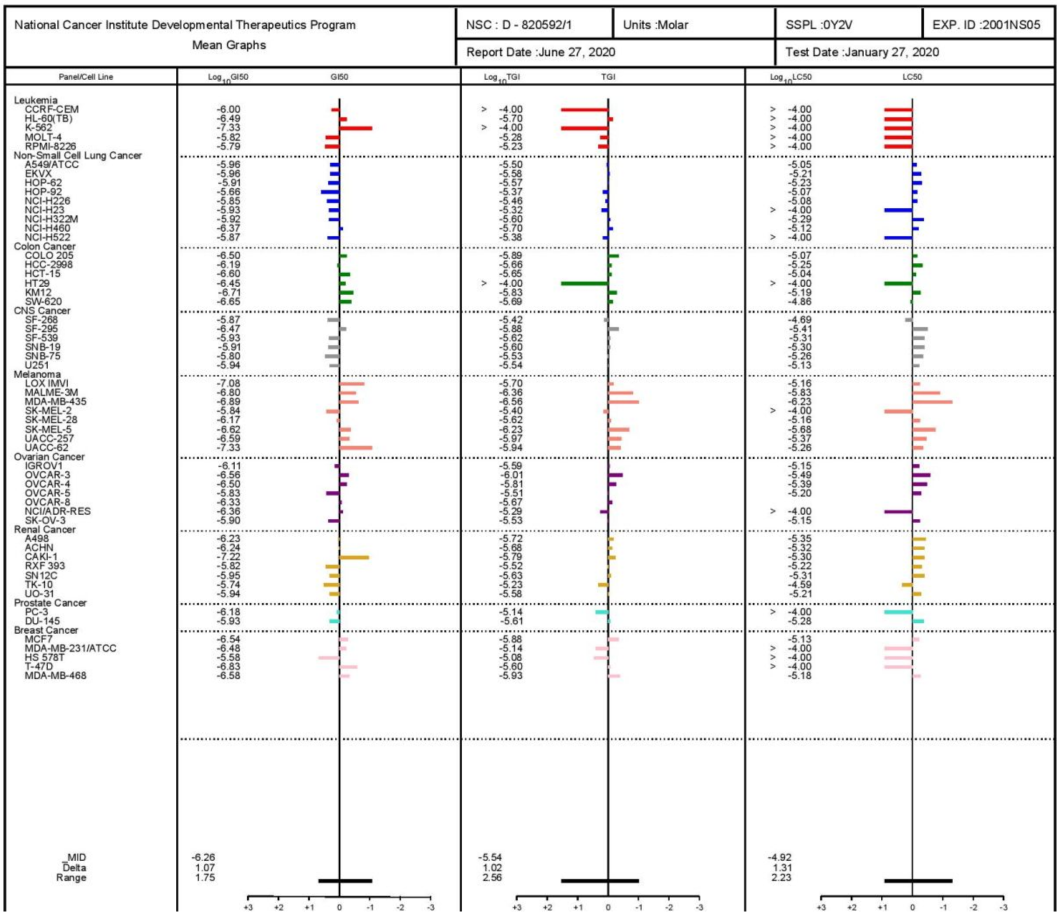

Mean of GI_{50} across 58 cell lines for compound **15** as Log_{10} Concentration (SD): -6.288 (±0.75)

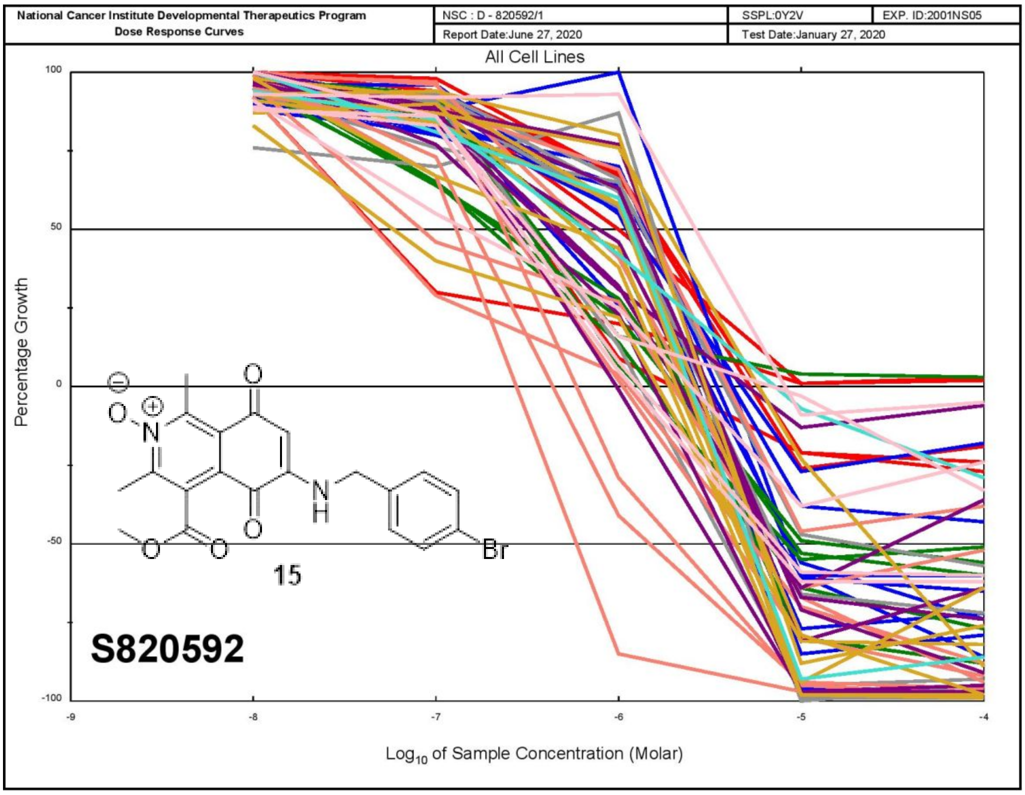

Figure S37** NCI Five Dose data cell line comparison summary for compound **15

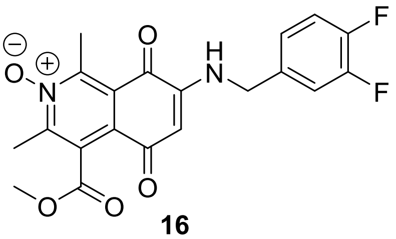

16

Developmental Therapeutics Program
One Dose Mean Graph

NSC: D-815370 / 1 | **Conc:** 1.00E-5 Molar | **Test Date:** May 20, 2019

Experiment ID: 1905OS11 | **Report Date:** Jun 10, 2019

Panel/Cell Line	Growth Percent
Leukemia	
CCRF-CEM	21.49
HL-60(TB)	-23.65
K-562	18.44
MOLT-4	8.41
RPMI-8226	29.91
SR	20.43
Non-Small Cell Lung Cancer	
A549/ATCC	8.81
EKVX	13.03
HOP-62	33.08
HOP-92	51.10
NCI-H23	-5.80
NCI-H322M	13.36
NCI-H460	4.70
NCI-H522	22.01
Colon Cancer	
COLO 205	-5.47
HCC-2998	9.25
HCT-116	1.74
HCT-15	6.07
HT29	11.59
KM12	10.37
SW-620	3.60
CNS Cancer	
SF-268	12.03
SF-295	18.20
SF-539	2.45
SNB-19	8.33
SNB-75	83.58
U251	2.99
Melanoma	
LOX IMVI	5.30
MALME-3M	-70.77
M14	4.33
MDA-MB-435	-88.42
SK-MEL-2	43.10
SK-MEL-28	50.21
SK-MEL-5	-97.05
UACC-257	-30.03
UACC-62	-15.69
Ovarian Cancer	
IGROV1	0.32
OVCAR-3	-19.04
OVCAR-4	-93.42
OVCAR-5	41.36
OVCAR-8	4.63
NCI/ADR-RES	-6.62
SK-OV-3	69.12
Renal Cancer	
786-0	48.23
ACHN	22.14
CAKI-1	43.01
RXF 393	32.18
SN12C	1.10
TK-10	85.50
UO-31	58.75
Prostate Cancer	
PC-3	15.60
DU-145	41.98
Breast Cancer	
MCF7	-43.33
MDA-MB-231/ATCC	-2.52
HS 578T	80.19
BT-549	66.36
T-47D	10.89
MDA-MB-468	-26.14
Mean	10.54
Delta	107.59
Range	182.55

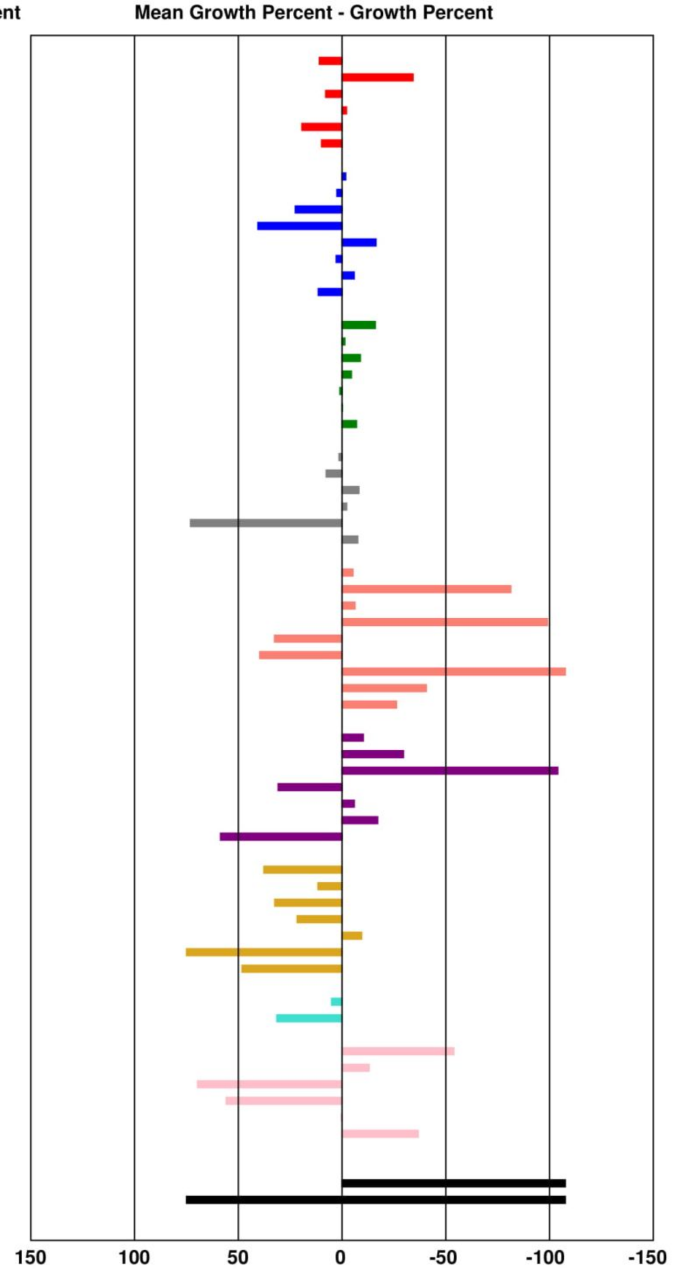

Figure S38 *NCI One Dose (10 µM) data for compound **16***

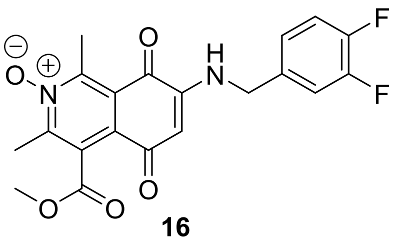

16

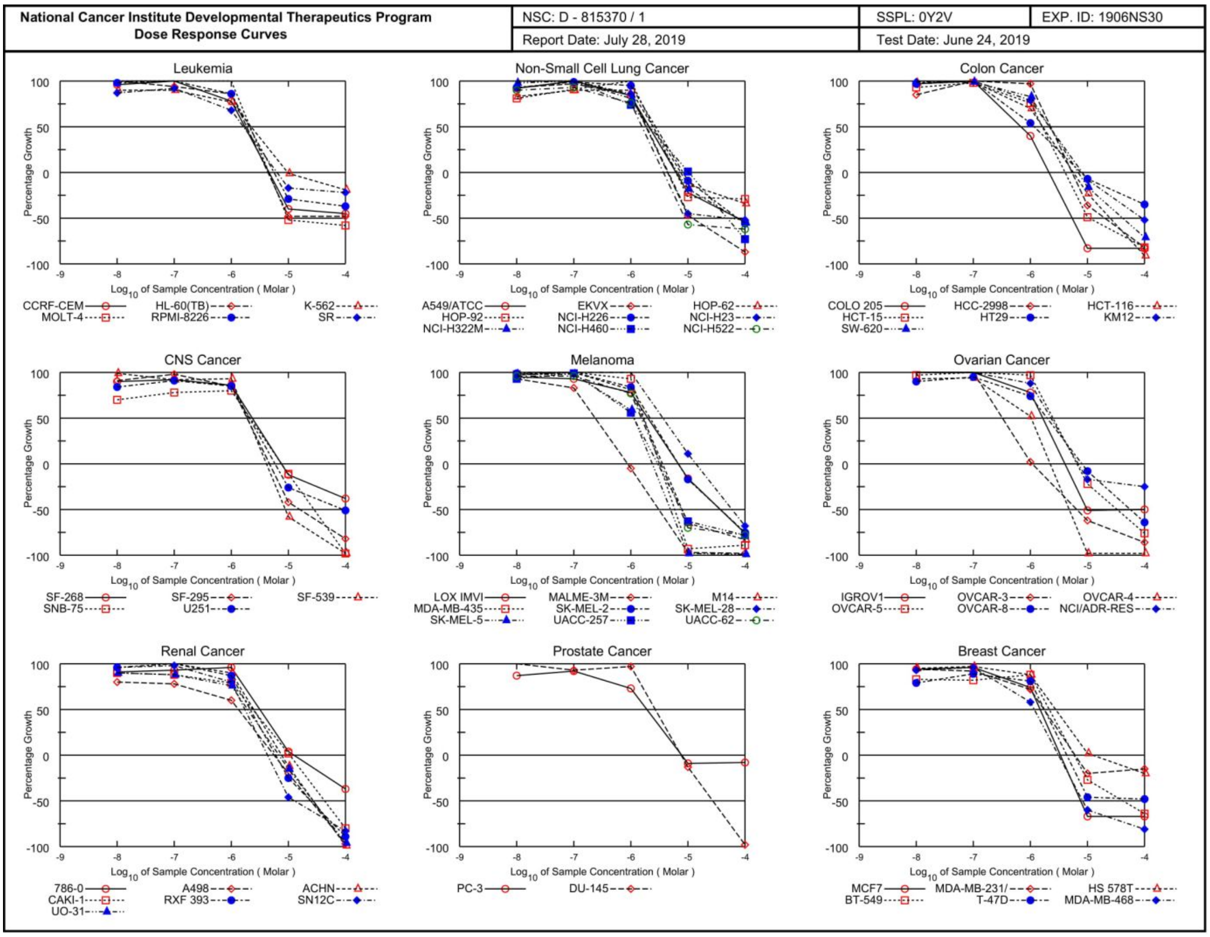

Figure S39** NCI Five Dose response summary data for compound **16

Table S13** NCI Five Dose raw data for compound **16

National Cancer Institute Developmental Therapeutics Program In-Vitro Testing Results			
NSC : D - 815370 / 1	Experiment ID : 1906NS30	Test Type : 08	Units : Molar
Report Date : July 28, 2019	Test Date : June 24, 2019	QNS :	MC :
COMI : RK-CC-003	Stain Reagent : SRB Dual-Pass Related	SSPL : 0Y2V	

			Log10 Concentration												
	Time		Mean Optical Densities					Percent Growth							
Panel/Cell Line	Zero	Ctrl	-8.0	-7.0	-6.0	-5.0	-4.0	-8.0	-7.0	-6.0	-5.0	-4.0	GI50	TGI	LC50
**Leukemia**															
CCRF-CEM	0.555	2.338	2.260	2.370	1.941	0.333	0.307	96	102	78	-40	-45	1.72E-6	4.57E-6	> 1.00E-4
HL-60(TB)	0.970	3.133	3.176	3.001	2.835	0.507	0.501	102	94	86	-48	-48	1.86E-6	4.40E-6	> 1.00E-4
K-562	0.175	1.369	1.247	1.255	1.099	0.173	0.142	90	90	77	-1	-19	2.23E-6	9.59E-6	> 1.00E-4
MOLT-4	0.690	2.474	2.584	2.969	2.739	0.328	0.287	106	128	115	-52	-58	2.44E-6	4.86E-6	9.67E-6
RPMI-8226	0.896	2.775	2.731	2.796	2.505	0.638	0.562	98	101	86	-29	-37	2.05E-6	5.60E-6	> 1.00E-4
SR	0.401	1.135	1.042	1.073	0.899	0.331	0.313	87	92	68	-17	-22	1.62E-6	6.24E-6	> 1.00E-4
**Non-Small Cell Lung Cancer**															
A549/ATCC	0.290	1.919	1.791	1.920	1.665	0.225	0.135	92	100	84	-22	-54	2.10E-6	6.17E-6	7.66E-5
EKVX	0.554	1.867	1.772	1.823	1.667	0.293	0.074	93	97	85	-47	-87	1.84E-6	4.39E-6	1.18E-5
HOP-62	0.711	1.813	1.623	1.699	1.704	0.618	0.468	83	90	90	-13	-34	2.45E-6	7.47E-6	> 1.00E-4
HOP-92	1.036	1.609	1.499	1.561	1.680	0.761	0.731	81	91	112	-27	-29	2.81E-6	6.43E-6	> 1.00E-4
NCI-H226	1.126	2.385	2.300	2.367	2.318	1.022	0.491	93	99	95	-9	-56	2.69E-6	8.14E-6	7.30E-5
NCI-H23	0.613	1.868	1.883	1.962	1.687	0.338	0.297	101	107	86	-45	-52	1.87E-6	4.52E-6	5.83E-5
NCI-H322M	0.743	1.960	1.940	2.016	1.734	0.610	0.335	98	105	81	-18	-55	2.07E-6	6.59E-6	7.34E-5
NCI-H460	0.287	2.764	2.858	2.891	2.119	0.307	0.077	104	105	74	1	-73	2.13E-6	1.02E-5	4.84E-5
NCI-H522	0.820	2.272	2.128	2.175	1.924	0.357	0.310	90	93	76	-57	-62	1.57E-6	3.75E-6	8.93E-6
**Colon Cancer**															
COLO 205	0.585	2.203	2.152	2.207	1.225	0.098	0.102	97	100	40	-83	-83	6.73E-7	2.10E-6	5.36E-6
HCC-2998	0.932	2.543	2.302	2.545	2.499	0.594	0.171	85	100	97	-36	-82	2.26E-6	5.35E-6	2.00E-5
HCT-116	0.244	2.059	2.017	2.037	1.517	0.189	0.022	98	99	70	-23	-91	1.65E-6	5.71E-6	2.52E-5
HCT-15	0.278	2.113	1.980	2.071	1.666	0.141	0.051	93	98	76	-49	-82	1.60E-6	4.02E-6	1.04E-5
HT29	0.208	1.712	1.669	1.801	1.015	0.193	0.136	97	106	54	-7	-35	1.15E-6	7.61E-6	> 1.00E-4
KM12	0.579	2.965	2.945	3.000	2.462	0.535	0.277	99	101	79	-8	-52	2.16E-6	8.15E-6	8.90E-5
SW-620	0.258	1.891	1.899	1.875	1.619	0.217	0.074	100	99	83	-16	-71	2.17E-6	6.89E-6	4.11E-5
**CNS Cancer**															
SF-268	0.838	2.441	2.287	2.311	2.211	0.734	0.522	90	92	86	-12	-38	2.31E-6	7.47E-6	> 1.00E-4
SF-295	0.781	2.926	2.744	2.877	2.604	0.450	0.144	91	98	85	-42	-82	1.88E-6	4.64E-6	1.56E-5
SF-539	1.054	2.819	2.804	2.679	2.695	0.440	0.018	99	92	93	-58	-98	1.92E-6	4.12E-6	8.81E-6
SNB-75	1.027	1.811	1.578	1.639	1.656	0.915	0.023	70	78	80	-11	-98	2.15E-6	7.58E-6	2.82E-5
U251	0.272	1.582	1.369	1.461	1.381	0.202	0.134	84	91	85	-26	-51	2.06E-6	5.85E-6	9.35E-5
**Melanoma**															
LOX IMVI	0.496	2.775	2.664	2.623	2.270	0.417	0.119	95	93	78	-16	-76	1.98E-6	6.76E-6	3.69E-5
MALME-3M	0.763	1.675	1.608	1.522	0.724	0.021	0.018	93	83	-5	-97	-98	2.38E-7	8.74E-7	3.07E-6
M14	0.430	1.807	1.761	1.911	1.550	0.152	0.072	97	108	81	-65	-83	1.64E-6	3.60E-6	7.92E-6
MDA-MB-435	0.512	2.664	2.620	2.688	2.504	0.036	0.056	98	101	93	-93	-89	1.70E-6	3.15E-6	5.87E-6
SK-MEL-2	1.127	2.562	2.552	2.610	2.329	0.931	0.282	99	103	84	-17	-75	2.16E-6	6.73E-6	3.68E-5
SK-MEL-28	0.772	1.914	2.042	2.156	2.047	0.900	0.246	111	121	112	11	-68	4.11E-6	1.38E-5	5.90E-5
SK-MEL-5	0.784	3.159	3.115	3.101	2.188	0.020	0.004	98	98	59	-98	-99	1.14E-6	2.38E-6	4.97E-6
UACC-257	0.739	1.905	1.821	1.890	1.394	0.275	0.161	93	99	56	-63	-78	1.13E-6	2.96E-6	7.80E-6
UACC-62	0.975	2.652	2.582	2.591	2.268	0.296	0.218	96	96	77	-70	-78	1.53E-6	3.35E-6	7.35E-6
**Ovarian Cancer**															
IGROV1	0.547	2.134	2.128	2.130	1.788	0.270	0.274	100	100	78	-51	-50	1.66E-6	4.05E-6	.
OVCAR-3	0.469	1.531	1.546	1.553	0.495	0.179	0.067	101	102	2	-62	-86	3.33E-7	1.09E-6	6.54E-6
OVCAR-4	0.560	1.500	1.435	1.447	1.047	0.013	0.009	93	94	52	-98	-98	1.03E-6	2.22E-6	4.80E-6
OVCAR-5	0.600	1.584	1.556	1.588	1.559	0.471	0.147	97	100	97	-22	-76	2.50E-6	6.59E-6	3.36E-5
OVCAR-8	0.444	2.368	2.167	2.277	1.868	0.408	0.160	90	95	74	-8	-64	1.96E-6	7.97E-6	5.60E-5
NCI/ADR-RES	0.563	2.074	2.076	2.118	1.896	0.466	0.420	100	103	88	-17	-25	2.30E-6	6.86E-6	> 1.00E-4
**Renal Cancer**															
786-0	0.921	2.757	2.588	2.634	2.680	1.000	0.583	91	93	96	4	-37	3.17E-6	1.27E-5	> 1.00E-4
A498	1.553	2.098	1.988	1.976	1.881	1.245	0.052	80	78	60	-20	-97	1.34E-6	5.65E-6	2.47E-5
ACHN	0.354	1.569	1.591	1.618	1.451	0.313	0.003	102	104	90	-12	-99	2.48E-6	7.68E-6	2.74E-5
CAKI-1	0.772	2.673	2.473	2.447	2.278	0.806	0.152	90	88	79	2	-80	2.39E-6	1.05E-5	4.27E-5
RXF 393	0.883	1.677	1.642	1.678	1.577	0.660	0.096	96	100	87	-25	-89	2.15E-6	5.96E-6	2.44E-5
SN12C	0.558	1.964	1.902	1.942	1.679	0.303	0.097	96	98	80	-46	-83	1.73E-6	4.32E-6	1.31E-5
UO-31	0.542	1.595	1.492	1.474	1.345	0.463	0.023	90	88	76	-15	-96	1.94E-6	6.90E-6	2.73E-5
**Prostate Cancer**															
PC-3	0.573	1.664	1.523	1.577	1.370	0.521	0.525	87	92	73	-9	-8	1.91E-6	7.75E-6	> 1.00E-4
DU-145	0.410	1.710	1.739	1.613	1.667	0.357	0.007	102	93	97	-13	-98	2.66E-6	7.60E-6	2.71E-5
**Breast Cancer**															
MCF7	0.467	2.487	2.370	2.402	1.962	0.156	0.154	94	96	74	-67	-67	1.48E-6	3.36E-6	7.62E-6
MDA-MB-231/ATCC	0.775	1.855	1.792	1.769	1.556	0.621	0.660	94	92	72	-20	-15	1.75E-6	6.09E-6	> 1.00E-4
HS 578T	0.869	1.941	1.884	1.910	1.815	0.886	0.700	95	97	88	2	-20	2.76E-6	1.19E-5	> 1.00E-4
BT-549	1.031	2.013	1.848	1.838	1.895	0.757	0.375	83	82	88	-27	-64	2.14E-6	5.86E-6	4.28E-5
T-47D	0.723	1.736	1.528	1.622	1.541	0.390	0.376	79	89	81	-46	-48	1.75E-6	4.33E-6	> 1.00E-4
MDA-MB-468	0.732	1.442	1.394	1.416	1.140	0.290	0.141	93	96	58	-60	-81	1.16E-6	3.07E-6	8.15E-6

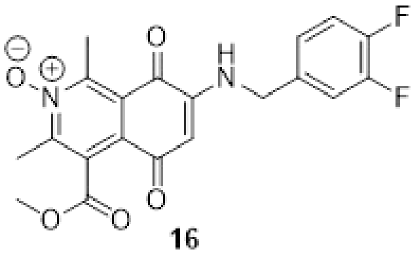

16

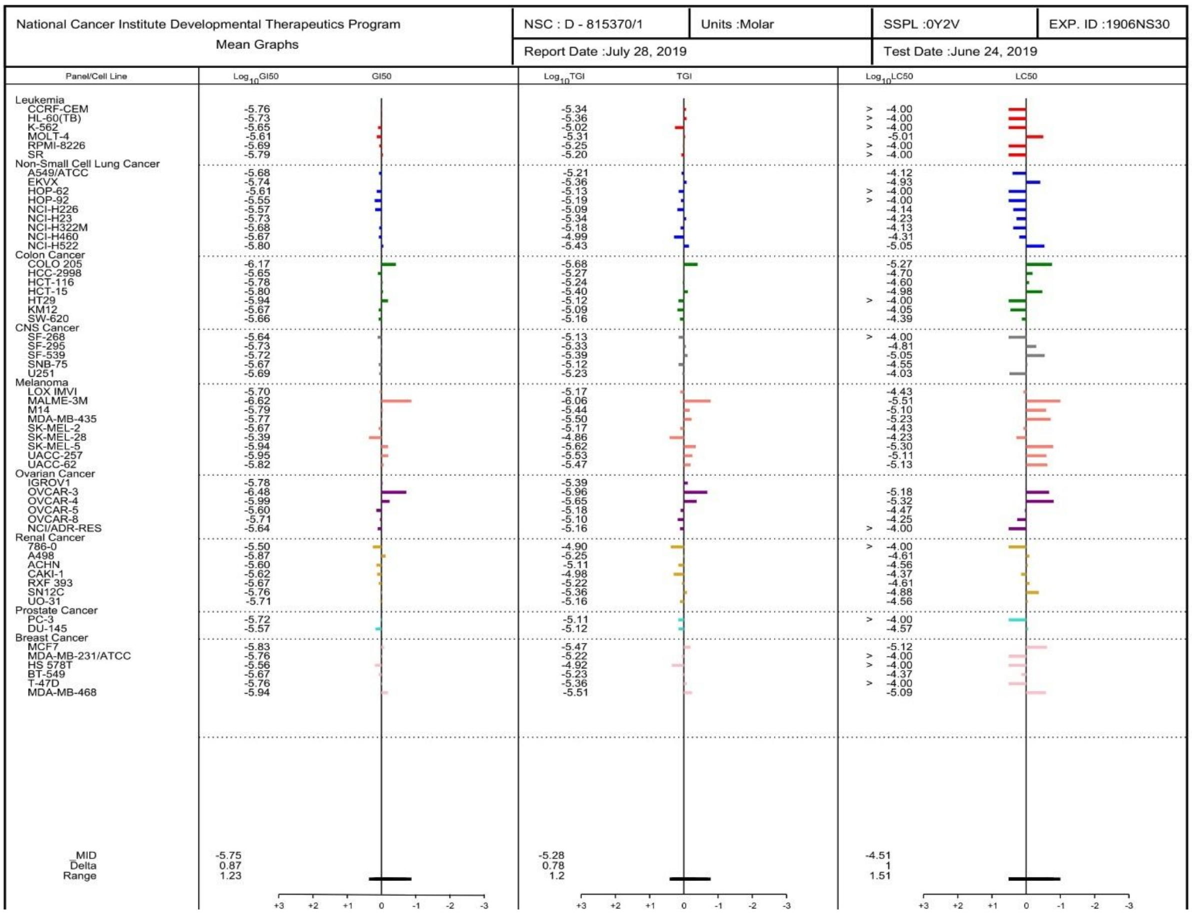

Mean of GI_{50} across 57 cell lines for compound **16** as Log_{10} Concentration (SD): -5.750 (±0.20)

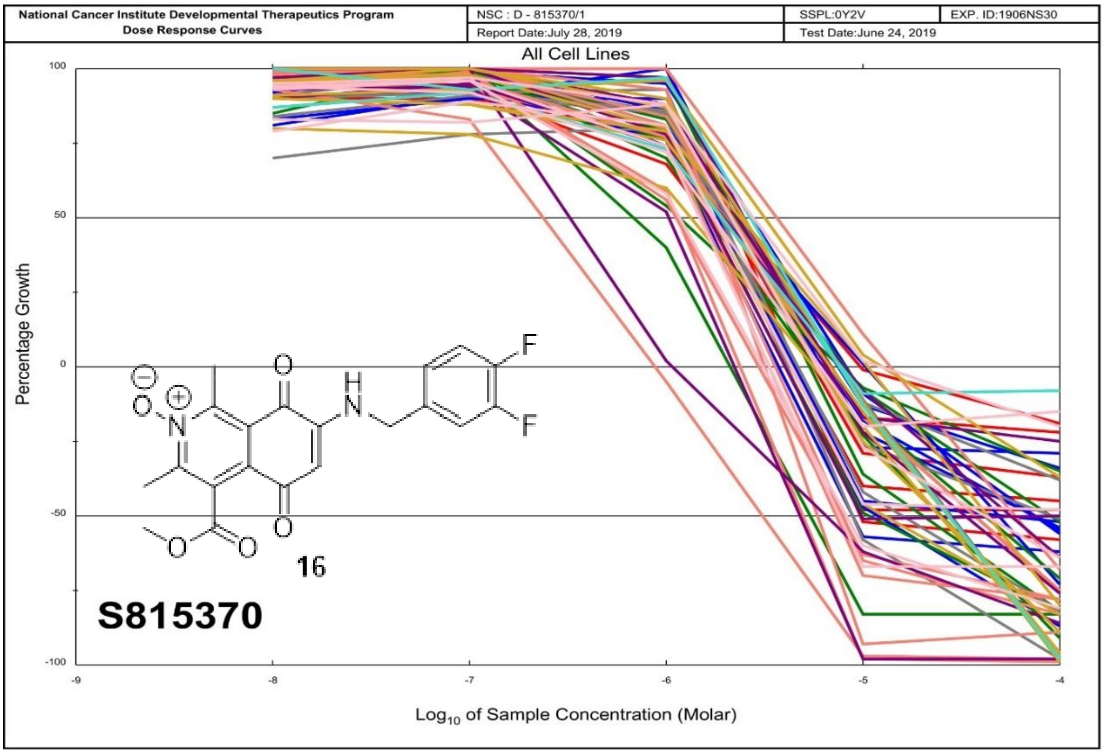

Figure S40 *NCI Five Dose data cell line comparison summary for compound* ***16***

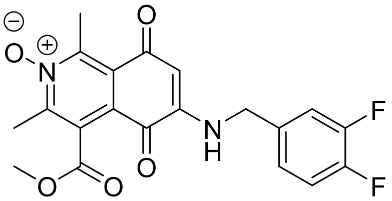

17

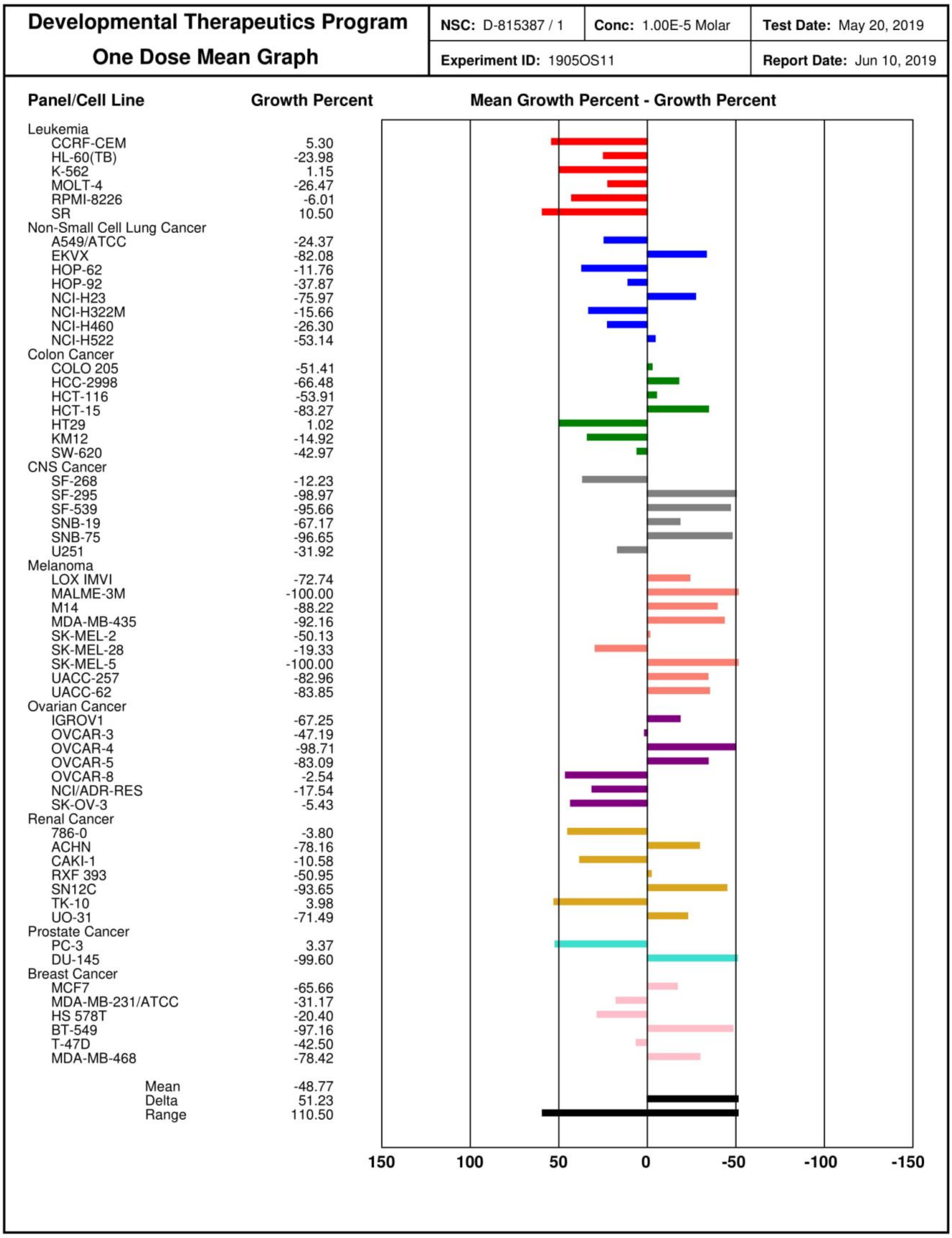

Developmental Therapeutics Program
One Dose Mean Graph

NSC: D-815387 / 1 | **Conc:** 1.00E-5 Molar | **Test Date:** May 20, 2019
Experiment ID: 1905OS11 | **Report Date:** Jun 10, 2019

Panel/Cell Line	Growth Percent
Leukemia	
CCRF-CEM	5.30
HL-60(TB)	-23.98
K-562	1.15
MOLT-4	-26.47
RPMI-8226	-6.01
SR	10.50
Non-Small Cell Lung Cancer	
A549/ATCC	-24.37
EKVX	-82.08
HOP-62	-11.76
HOP-92	-37.87
NCI-H23	-75.97
NCI-H322M	-15.66
NCI-H460	-26.30
NCI-H522	-53.14
Colon Cancer	
COLO 205	-51.41
HCC-2998	-66.48
HCT-116	-53.91
HCT-15	-83.27
HT29	1.02
KM12	-14.92
SW-620	-42.97
CNS Cancer	
SF-268	-12.23
SF-295	-98.97
SF-539	-95.66
SNB-19	-67.17
SNB-75	-96.65
U251	-31.92
Melanoma	
LOX IMVI	-72.74
MALME-3M	-100.00
M14	-88.22
MDA-MB-435	-92.16
SK-MEL-2	-50.13
SK-MEL-28	-19.33
SK-MEL-5	-100.00
UACC-257	-82.96
UACC-62	-83.85
Ovarian Cancer	
IGROV1	-67.25
OVCAR-3	-47.19
OVCAR-4	-98.71
OVCAR-5	-83.09
OVCAR-8	-2.54
NCI/ADR-RES	-17.54
SK-OV-3	-5.43
Renal Cancer	
786-0	-3.80
ACHN	-78.16
CAKI-1	-10.58
RXF 393	-50.95
SN12C	-93.65
TK-10	3.98
UO-31	-71.49
Prostate Cancer	
PC-3	3.37
DU-145	-99.60
Breast Cancer	
MCF7	-65.66
MDA-MB-231/ATCC	-31.17
HS 578T	-20.40
BT-549	-97.16
T-47D	-42.50
MDA-MB-468	-78.42
Mean	-48.77
Delta	51.23
Range	110.50

Figure S41 *NCI One Dose (10 μM) data for compound **17***

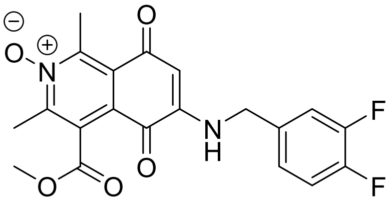

17

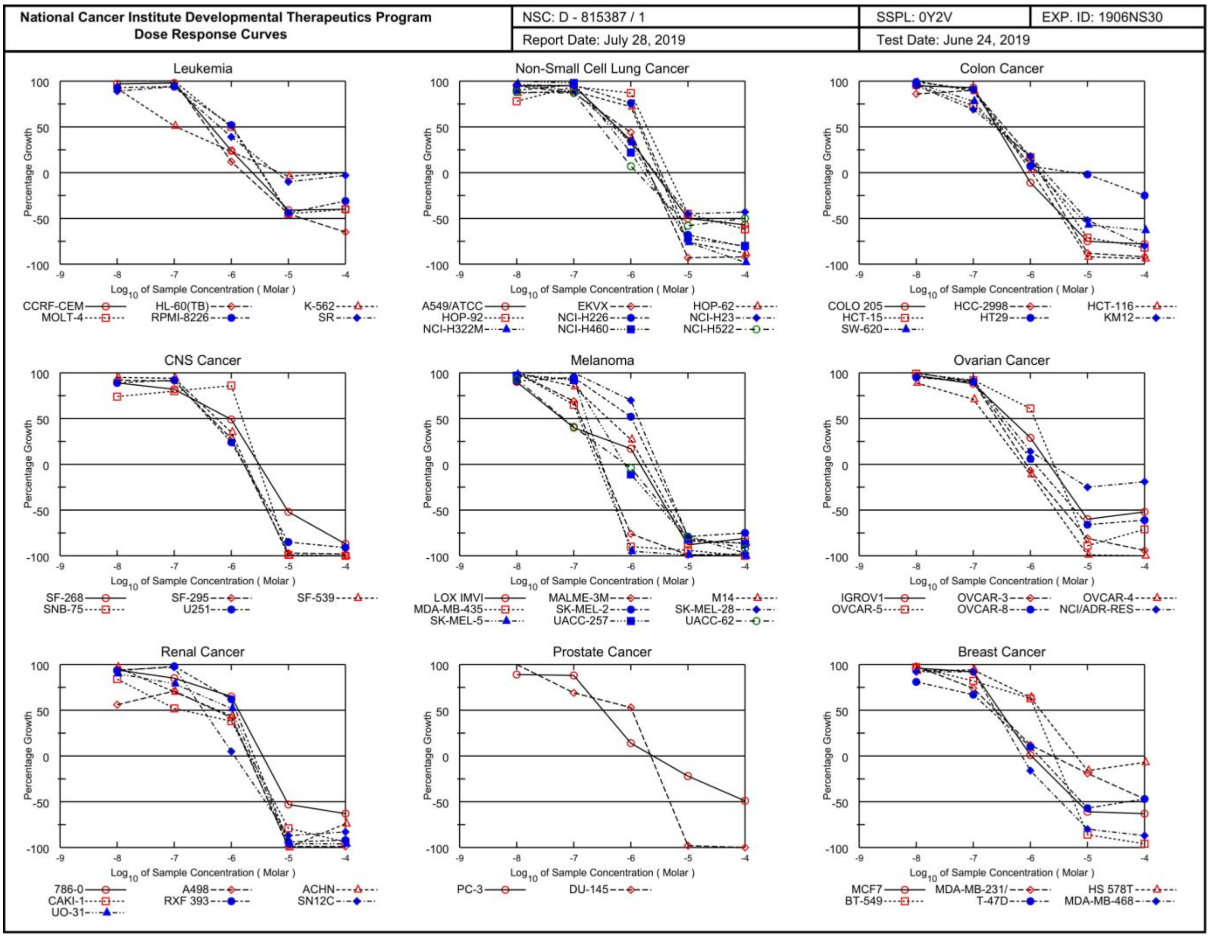

Figure S42 *NCI Five Dose response summary data for compound* ***17***

Table S14** NCI Five Dose raw data for compound **17

National Cancer Institute Developmental Therapeutics Program
In-Vitro Testing Results

NSC : D - 815387 / 1	Experiment ID : 1906NS30	Test Type : 08	Units : Molar
Report Date : July 28, 2019	Test Date : June 24, 2019	QNS :	MC :
COMI : RK-CC-0031	Stain Reagent : SRB Dual-Pass Related	SSPL : 0Y2V	

			Log10 Concentration													
	Time		Mean Optical Densities					Percent Growth								
Panel/Cell Line	Zero	Ctrl	-8.0	-7.0	-6.0	-5.0	-4.0	-8.0	-7.0	-6.0	-5.0	-4.0	GI50	TGI	LC50
**Leukemia**															
CCRF-CEM	0.555	2.295	2.235	2.252	0.981	0.329	0.335	97	98	24	-41	-40	4.47E-7	2.37E-6	> 1.00E-4
HL-60(TB)	0.970	3.171	3.211	3.165	1.225	0.537	0.344	102	100	12	-45	-65	3.67E-7	1.61E-6	1.86E-5
K-562	0.175	1.359	1.265	0.785	0.448	0.168	0.175	92	51	23	-4	.	1.13E-7	1.00E-4	> 1.00E-4
MOLT-4	0.690	2.593	2.644	2.836	1.642	0.380	0.416	103	113	50	-45	-40	1.00E-6	3.36E-6	> 1.00E-4
RPMI-8226	0.896	2.732	2.603	2.614	1.856	0.506	0.619	93	94	52	-44	-31	1.06E-6	3.51E-6	> 1.00E-4
SR	0.401	1.151	1.070	1.107	0.691	0.361	0.389	89	94	39	-10	-3	6.23E-7	6.20E-6	> 1.00E-4
**Non-Small Cell Lung Cancer**															
A549/ATCC	0.290	1.888	1.813	1.816	0.853	0.146	0.124	95	95	35	-50	-57	5.68E-7	2.60E-6	1.11E-5
EKVX	0.554	1.872	1.801	1.696	1.139	0.037	0.044	95	87	44	-93	-92	7.36E-7	2.10E-6	4.84E-6
HOP-62	0.711	1.774	1.639	1.656	1.479	0.169	0.084	87	89	72	-76	-88	1.41E-6	3.07E-6	6.66E-6
HOP-92	1.036	1.593	1.472	1.561	1.518	0.572	0.399	78	94	87	-45	-62	1.90E-6	4.56E-6	2.05E-5
NCI-H226	1.126	2.438	2.310	2.389	2.124	0.356	0.215	90	96	76	-68	-81	1.51E-6	3.36E-6	7.46E-6
NCI-H23	0.613	1.957	1.909	1.895	1.066	0.337	0.351	96	95	34	-45	-43	5.44E-7	2.68E-6	> 1.00E-4
NCI-H322M	0.743	2.036	1.993	1.892	1.167	0.176	0.019	97	89	33	-76	-98	4.93E-7	2.00E-6	5.73E-6
NCI-H460	0.287	2.999	3.051	2.940	0.890	0.082	0.058	102	98	22	-72	-80	4.29E-7	1.73E-6	5.88E-6
NCI-H522	0.820	2.375	2.194	2.169	0.926	0.348	0.412	88	87	7	-58	-50	2.88E-7	1.27E-6	.
**Colon Cancer**															
COLO 205	0.585	2.250	2.174	2.131	0.524	0.148	0.131	95	93	-11	-75	-78	2.60E-7	7.91E-7	4.11E-6
HCC-2998	0.932	2.726	2.468	2.548	1.238	0.114	0.072	86	90	17	-88	-92	3.54E-7	1.45E-6	4.36E-6
HCT-116	0.244	2.172	2.168	1.954	0.295	0.021	0.015	100	89	3	-92	-94	2.81E-7	1.07E-6	3.62E-6
HCT-15	0.278	2.242	2.166	1.722	0.620	0.081	0.049	96	73	17	-71	-82	2.62E-7	1.57E-6	5.80E-6
HT29	0.208	1.740	1.722	1.597	0.317	0.203	0.156	99	91	7	-2	-25	3.07E-7	5.58E-6	> 1.00E-4
KM12	0.579	3.014	2.886	2.262	1.028	0.276	0.115	95	69	18	-52	-80	2.38E-7	1.82E-6	9.24E-6
SW-620	0.258	2.146	2.105	1.732	0.424	0.111	0.095	98	78	9	-57	-63	2.54E-7	1.36E-6	7.78E-6
**CNS Cancer**															
SF-268	0.838	2.476	2.295	2.184	1.633	0.405	0.105	89	82	49	-52	-87	9.04E-7	3.05E-6	9.62E-6
SF-295	0.781	3.020	2.846	2.828	1.407	0.027	0.018	92	91	28	-97	-98	4.49E-7	1.68E-6	4.22E-6
SF-539	1.054	2.867	2.777	2.767	1.690	-0.002	-0.002	95	94	35	-100	-100	5.60E-7	1.82E-6	4.26E-6
SNB-75	1.027	1.758	1.566	1.611	1.658	0.006	.	74	80	86	-99	-100	1.57E-6	2.92E-6	5.42E-6
U251	0.272	1.575	1.437	1.467	0.581	0.041	0.026	89	92	24	-85	-91	4.10E-7	1.65E-6	4.77E-6
**Melanoma**															
LOX IMVI	0.496	2.743	2.517	1.400	0.884	0.060	0.096	90	40	17	-88	-81	6.36E-8	1.46E-6	4.35E-6
MALME-3M	0.763	1.655	1.653	1.378	0.180	0.010	0.014	100	69	-76	-99	-98	1.35E-7	2.98E-7	6.58E-7
M14	0.430	1.817	1.836	1.608	0.806	0.070	0.059	101	85	27	-84	-86	4.02E-7	1.76E-6	4.96E-6
MDA-MB-435	0.512	2.614	2.603	1.880	0.053	0.030	0.002	100	65	-90	-94	-100	1.25E-7	2.63E-7	5.54E-7
SK-MEL-2	1.127	2.600	2.470	2.529	1.886	0.242	0.281	91	95	52	-79	-75	1.03E-6	2.49E-6	6.03E-6
SK-MEL-28	0.772	2.033	2.136	2.196	1.655	0.159	0.026	108	113	70	-79	-97	1.36E-6	2.94E-6	6.36E-6
SK-MEL-5	0.784	3.171	3.121	3.007	0.037	0.007	-0.002	98	93	-95	-99	-100	1.69E-7	3.12E-7	5.75E-7
UACC-257	0.739	1.881	1.850	1.791	0.660	0.135	0.094	97	92	-11	-82	-87	2.57E-7	7.86E-7	3.57E-6
UACC-62	0.975	2.590	2.472	1.643	0.937	0.207	0.082	93	41	-4	-79	-92	6.78E-8	8.20E-7	4.12E-6
**Ovarian Cancer**															
IGROV1	0.547	2.237	2.185	2.042	1.043	0.222	0.261	97	88	29	-60	-52	4.47E-7	2.14E-6	7.82E-6
OVCAR-3	0.469	1.627	1.666	1.513	0.435	0.090	0.027	103	90	-7	-81	-94	2.58E-7	8.40E-7	3.81E-6
OVCAR-4	0.560	1.473	1.372	1.207	0.501	0.004	-0.003	89	71	-11	-99	-100	1.80E-7	7.42E-7	2.78E-6
OVCAR-5	0.600	1.597	1.586	1.518	1.209	0.067	0.175	99	92	61	-89	-71	1.18E-6	2.55E-6	5.51E-6
OVCAR-8	0.444	2.328	2.237	2.139	0.556	0.150	0.173	95	90	6	-66	-61	2.99E-7	1.21E-6	5.96E-6
NCI/ADR-RES	0.563	2.103	2.033	1.975	0.780	0.420	0.455	95	92	14	-25	-19	3.45E-7	2.27E-6	> 1.00E-4
**Renal Cancer**															
786-0	0.921	2.799	2.687	2.524	2.151	0.435	0.341	94	85	65	-53	-63	1.35E-6	3.58E-6	9.48E-6
A498	1.553	2.097	1.859	1.940	1.783	0.013	0.013	56	71	42	-99	-99	5.41E-7	1.99E-6	4.49E-6
ACHN	0.354	1.674	1.635	1.281	0.929	-0.002	0.092	97	70	44	-100	-74	5.72E-7	2.01E-6	4.48E-6
CAKI-1	0.772	2.615	2.313	1.740	1.465	0.162	0.046	84	52	38	-79	-94	1.47E-7	2.10E-6	5.64E-6
RXF 393	0.883	1.668	1.616	1.650	1.369	0.057	0.072	93	98	62	-94	-92	1.19E-6	2.50E-6	5.25E-6
SN12C	0.558	2.002	1.912	1.963	0.636	0.072	0.096	94	97	5	-87	-83	3.27E-7	1.14E-6	3.97E-6
UO-31	0.542	1.698	1.586	1.457	1.142	0.024	0.024	90	79	52	-96	-96	1.03E-6	2.25E-6	4.91E-6
**Prostate Cancer**															
PC-3	0.573	1.625	1.513	1.497	0.720	0.447	0.290	89	88	14	-22	-49	3.25E-7	2.44E-6	> 1.00E-4
DU-145	0.410	1.780	1.788	1.355	1.135	0.010	-0.002	101	69	53	-98	-100	1.05E-6	2.25E-6	4.83E-6
**Breast Cancer**															
MCF7	0.467	2.467	2.383	2.310	0.489	0.181	0.172	96	92	1	-61	-63	2.90E-7	1.04E-6	6.58E-6
MDA-MB-231/ATCC	0.775	1.770	1.751	1.515	0.890	0.632	0.414	98	74	12	-19	-47	2.44E-7	2.42E-6	> 1.00E-4
HS 578T	0.869	2.011	1.928	1.944	1.600	0.729	0.808	93	94	64	-16	-7	1.50E-6	6.29E-6	> 1.00E-4
BT-549	1.031	2.050	2.016	1.867	1.678	0.147	0.046	97	82	63	-86	-96	1.23E-6	2.66E-6	5.76E-6
T-47D	0.723	1.684	1.497	1.371	0.821	0.311	0.384	81	67	10	-57	-47	2.02E-7	1.42E-6	.
MDA-MB-468	0.732	1.372	1.322	1.319	0.619	0.149	0.095	92	92	-16	-80	-87	2.45E-7	7.17E-7	3.45E-6

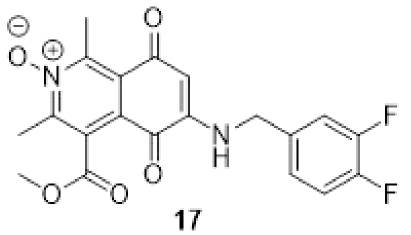

17

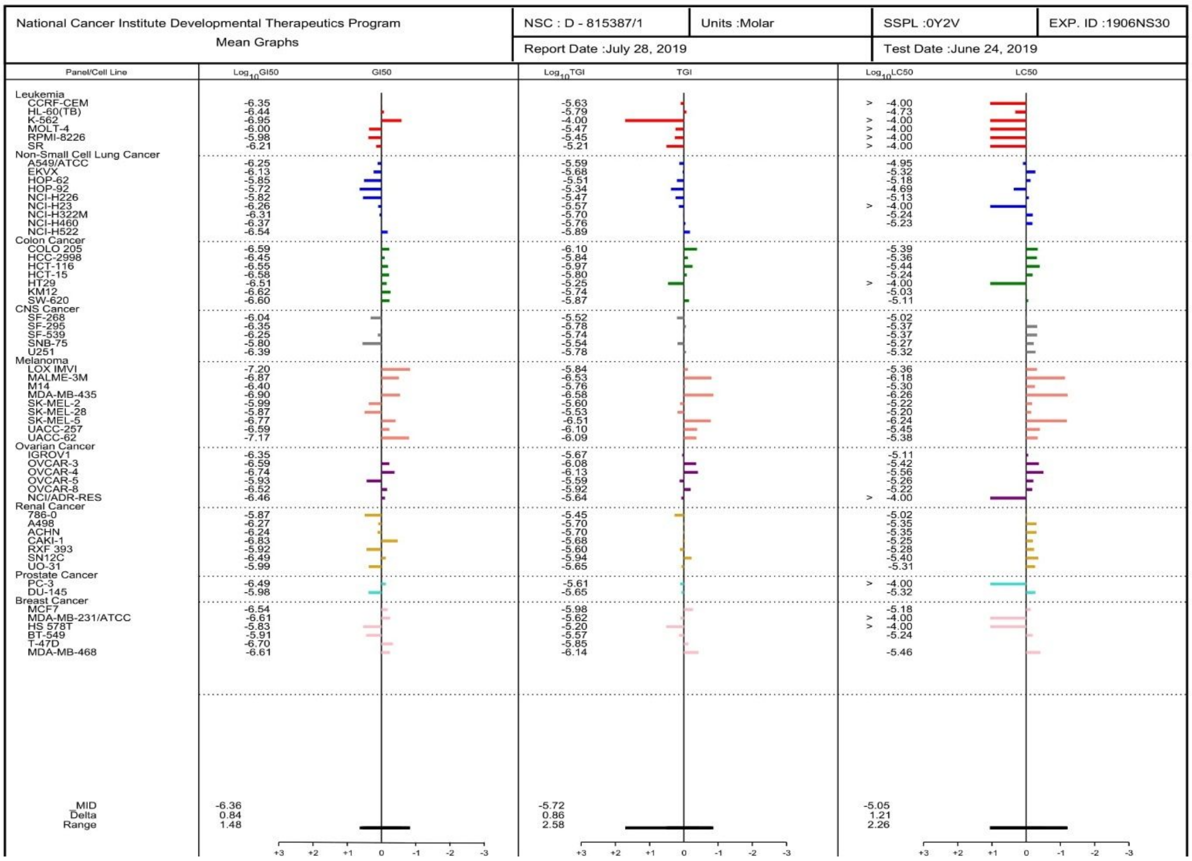

Mean of GI_{50} across 58 cell lines for compound **17** as Log_{10} Concentration (SD): -6.376 (±0.37)

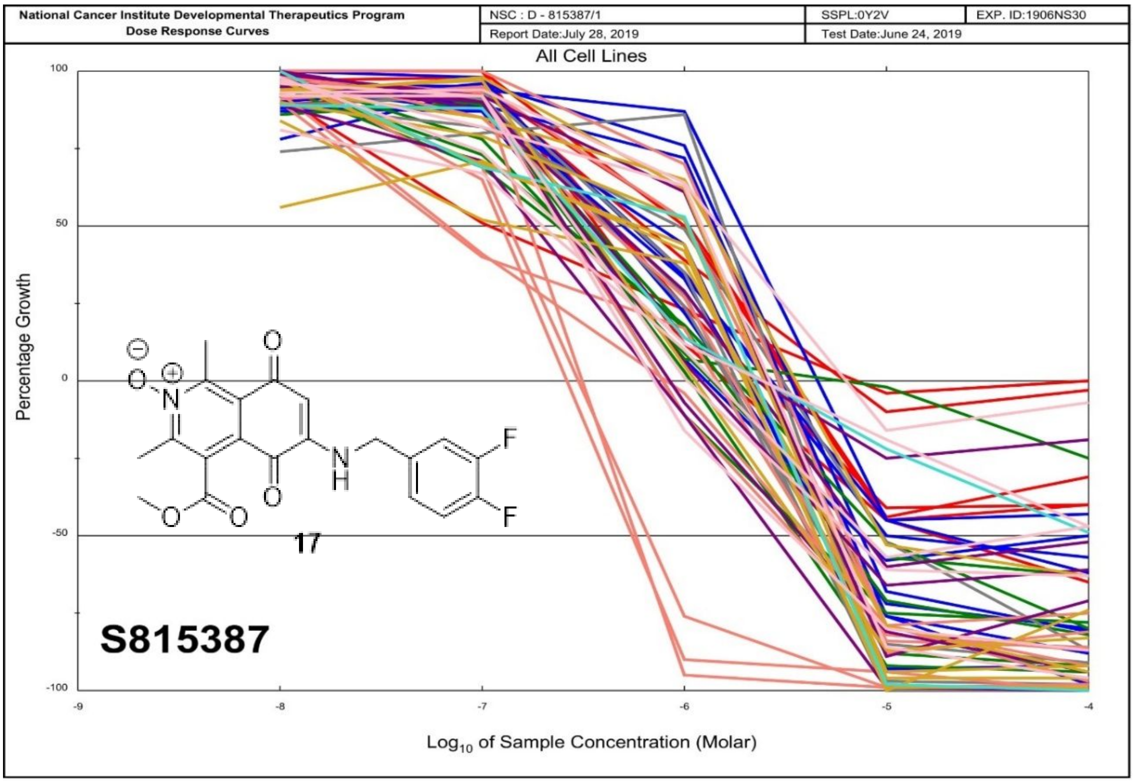

Figure S43 *NCI Five Dose data cell line comparison summary for compound* ***17***

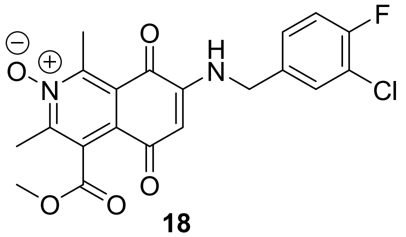

18

Developmental Therapeutics Program

One Dose Mean Graph

**NSC:** D-810562 / 1	**Conc:** 1.00E-5 Molar	**Test Date:** Dec 03, 2018
**Experiment ID:** 1812OS40		**Report Date:** Dec 20, 2018

Panel/Cell Line	Growth Percent
Leukemia	
HL-60(TB)	-54.63
K-562	5.87
MOLT-4	-19.21
RPMI-8226	20.55
Non-Small Cell Lung Cancer	
A549/ATCC	1.30
EKVX	37.77
HOP-62	28.31
HOP-92	19.03
NCI-H226	84.27
NCI-H23	16.33
NCI-H322M	3.24
NCI-H460	7.33
NCI-H522	-26.57
Colon Cancer	
COLO 205	-75.50
HCC-2998	13.34
HCT-116	0.43
HCT-15	8.29
HT29	5.00
KM12	11.47
SW-620	5.47
CNS Cancer	
SF-268	9.33
SF-295	38.96
SF-539	3.31
SNB-19	7.17
SNB-75	109.35
U251	-20.40
Melanoma	
LOX IMVI	9.57
MALME-3M	-93.48
M14	-19.57
MDA-MB-435	-82.43
SK-MEL-2	29.36
SK-MEL-28	57.92
SK-MEL-5	-71.61
UACC-257	-69.30
UACC-62	-42.00
Ovarian Cancer	
IGROV1	3.18
OVCAR-3	-21.28
OVCAR-4	-96.37
OVCAR-5	38.24
OVCAR-8	-14.52
NCI/ADR-RES	0.13
SK-OV-3	87.20
Renal Cancer	
786-0	-93.47
A498	78.03
ACHN	30.88
CAKI-1	33.98
RXF 393	36.17
SN12C	-0.15
TK-10	58.84
UO-31	41.71
Prostate Cancer	
PC-3	5.89
DU-145	36.23
Breast Cancer	
MCF7	-32.76
MDA-MB-231/ATCC	2.68
HS 578T	68.01
BT-549	43.74
T-47D	-5.44
MDA-MB-468	-18.05
Mean	4.16
Delta	100.53
Range	205.72

Mean Growth Percent - Growth Percent

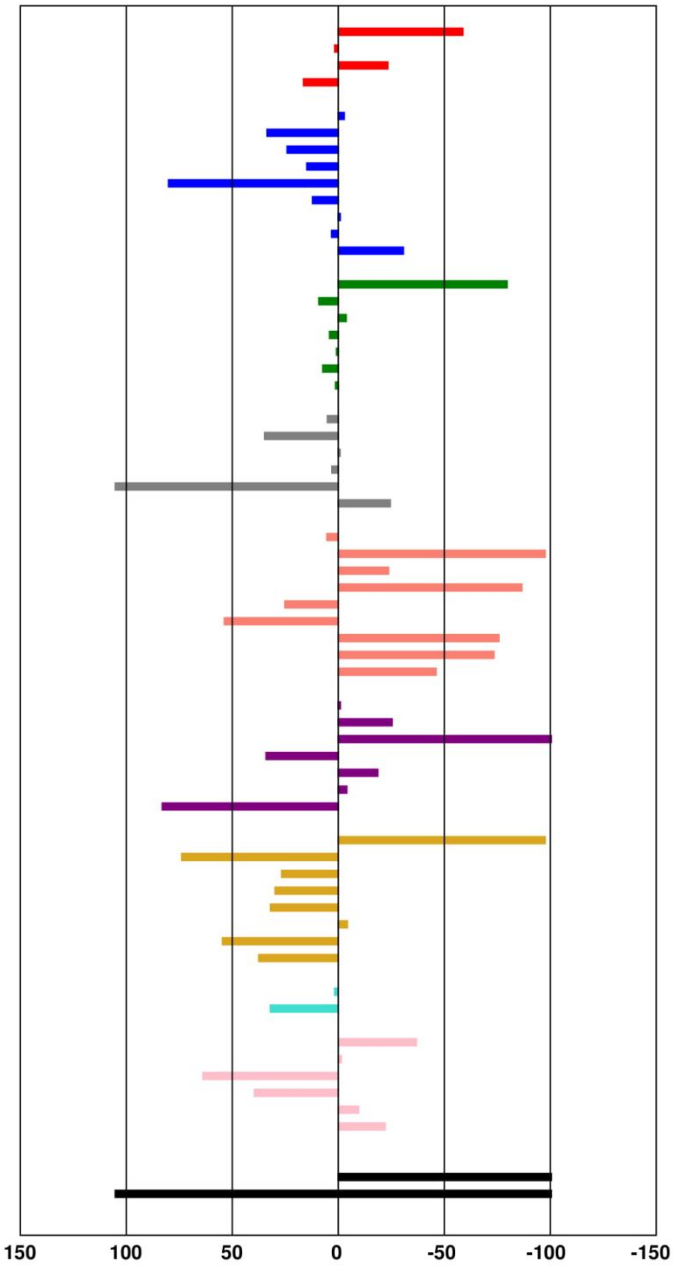

Figure S44 *NCI One Dose (10 µM) data for compound **18***

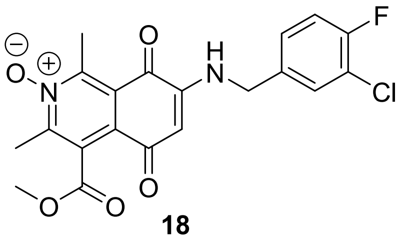

18

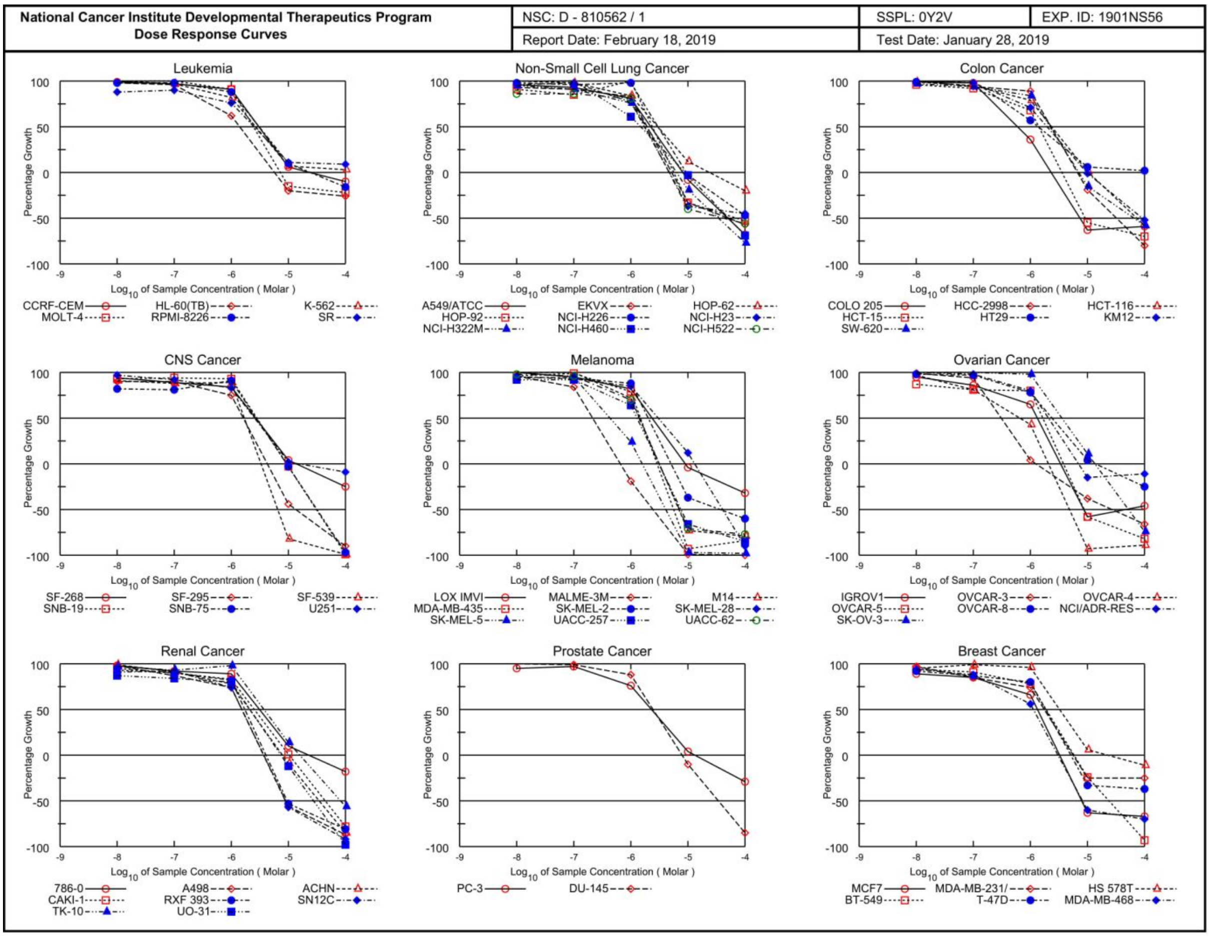

Figure S45 *NCI Five Dose response summary data for compound* ***18***

Table S15** NCI Five Dose raw data for compound **18

National Cancer Institute Developmental Therapeutics Program In-Vitro Testing Results			
NSC : D - 810562 / 1	Experiment ID : 1901NS56	Test Type : 08	Units : Molar
Report Date : February 18, 2019	Test Date : January 28, 2019	QNS :	MC :
COMI : RK 6.3.9	Stain Reagent : SRB Dual-Pass Related	SSPL : 0Y2V	

			Log10 Concentration												
	Time		Mean Optical Densities					Percent Growth							
Panel/Cell Line	Zero	Ctrl	-8.0	-7.0	-6.0	-5.0	-4.0	-8.0	-7.0	-6.0	-5.0	-4.0	GI50	TGI	LC50
Leukemia															
CCRF-CEM	0.580	2.998	2.971	2.931	2.784	0.719	0.521	99	97	91	6	-10	3.03E-6	2.28E-5	> 1.00E-4
HL-60(TB)	0.877	3.265	3.223	3.160	2.347	0.702	0.652	98	96	62	-20	-26	1.39E-6	5.68E-6	> 1.00E-4
K-562	0.214	2.586	2.644	2.503	2.164	0.370	0.284	102	97	82	7	3	2.67E-6	> 1.00E-4	> 1.00E-4
MOLT-4	0.550	2.892	2.941	2.881	2.674	0.467	0.431	102	100	91	-15	-22	2.42E-6	7.20E-6	> 1.00E-4
RPMI-8226	1.128	3.105	3.073	3.073	2.867	1.329	0.942	98	98	88	10	-16	3.08E-6	2.41E-5	> 1.00E-4
SR	0.281	1.644	1.483	1.512	1.312	0.435	0.409	88	90	76	11	9	2.50E-6	> 1.00E-4	> 1.00E-4
Non-Small Cell Lung Cancer															
A549/ATCC	0.373	2.495	2.410	2.340	2.091	0.344	0.118	96	93	81	-8	-68	2.23E-6	8.15E-6	4.95E-5
EKVX	0.679	1.950	1.866	1.836	1.738	0.453	0.293	93	91	83	-33	-57	1.93E-6	5.18E-6	5.11E-5
HOP-62	0.451	1.964	1.973	1.928	1.725	0.627	0.359	101	98	84	12	-20	2.96E-6	2.31E-5	> 1.00E-4
HOP-92	1.205	2.032	1.961	1.911	2.078	0.809	0.572	91	85	106	-33	-53	2.52E-6	5.79E-6	7.44E-5
NCI-H226	1.465	3.063	3.029	2.998	3.030	1.420	0.778	98	96	98	-3	-47	2.98E-6	9.32E-6	> 1.00E-4
NCI-H23	0.538	1.654	1.610	1.633	1.400	0.341	0.298	96	98	77	-37	-45	1.74E-6	4.77E-6	> 1.00E-4
NCI-H322M	0.694	2.006	1.942	1.886	1.689	0.565	0.157	95	91	76	-19	-77	1.88E-6	6.35E-6	3.42E-5
NCI-H460	0.284	3.012	3.080	3.094	1.961	0.277	0.089	102	103	61	-3	-69	1.51E-6	9.10E-6	5.19E-5
NCI-H522	0.911	2.854	2.585	2.586	2.494	0.550	0.404	86	86	81	-40	-56	1.82E-6	4.70E-6	4.43E-5
Colon Cancer															
COLO 205	0.443	2.003	1.989	1.971	0.999	0.162	0.180	99	98	36	-63	-59	5.88E-7	2.29E-6	7.32E-6
HCC-2998	0.652	2.429	2.432	2.323	2.225	0.529	0.130	100	94	89	-19	-80	2.28E-6	6.66E-6	3.22E-5
HCT-116	0.242	2.514	2.496	2.397	2.033	0.256	0.101	99	95	79	1	-58	2.34E-6	1.02E-5	7.19E-5
HCT-15	0.355	2.519	2.425	2.348	1.819	0.162	0.107	96	92	68	-55	-70	1.39E-6	3.58E-6	9.19E-6
HT29	0.240	2.130	2.110	2.182	1.309	0.357	0.274	99	103	57	6	2	1.35E-6	> 1.00E-4	> 1.00E-4
KM12	0.467	2.853	2.885	2.810	2.163	0.462	0.225	101	98	71	-1	-52	1.96E-6	9.63E-6	9.21E-5
SW-620	0.200	1.491	1.455	1.415	1.283	0.171	0.084	97	94	84	-15	-58	2.21E-6	7.12E-6	6.48E-5
CNS Cancer															
SF-268	0.759	2.556	2.451	2.366	2.265	0.823	0.566	94	89	84	4	-25	2.64E-6	1.32E-5	> 1.00E-4
SF-295	0.484	1.701	1.584	1.580	1.395	0.271	0.046	90	90	75	-44	-90	1.62E-6	4.26E-6	1.34E-5
SF-539	0.884	2.739	2.573	2.521	2.538	0.159	0.007	91	88	89	-82	-99	1.69E-6	3.32E-6	6.50E-6
SNB-19	0.749	2.668	2.525	2.559	2.534	0.728	0.009	93	94	93	-3	-99	2.81E-6	9.33E-6	3.10E-5
SNB-75	0.890	1.760	1.600	1.598	1.683	0.865	0.030	82	81	91	-3	-97	2.74E-6	9.32E-6	3.18E-5
U251	0.256	1.723	1.677	1.612	1.471	0.279	0.232	97	92	83	2	-9	2.53E-6	1.38E-5	> 1.00E-4
Melanoma															
LOX IMVI	0.406	2.977	2.992	2.851	2.512	0.388	0.275	101	95	82	-4	-32	2.34E-6	8.88E-6	> 1.00E-4
MALME-3M	0.679	1.751	1.704	1.579	0.553	0.009	-0.004	96	84	-19	-99	-100	2.14E-7	6.58E-7	2.47E-6
M14	0.388	1.831	1.846	1.909	1.423	0.104	0.081	101	105	72	-73	-79	1.41E-6	3.12E-6	6.90E-6
MDA-MB-435	0.443	2.302	2.234	2.291	1.913	0.029	0.073	96	99	79	-93	-84	1.47E-6	2.87E-6	5.60E-6
SK-MEL-2	1.315	3.052	2.974	2.955	2.846	0.828	0.527	95	94	88	-37	-60	2.02E-6	5.06E-6	3.68E-5
SK-MEL-28	0.607	2.248	2.173	2.135	2.003	0.808	0.059	95	93	85	12	-90	3.03E-6	1.32E-5	4.04E-5
SK-MEL-5	0.752	3.199	3.194	3.074	1.329	0.022	0.012	100	95	24	-97	-98	4.26E-7	1.57E-6	4.07E-6
UACC-257	1.097	2.811	2.673	2.672	2.193	0.371	0.160	92	92	64	-66	-85	1.28E-6	3.10E-6	7.51E-6
UACC-62	1.057	3.125	3.089	3.037	2.496	0.303	0.239	98	96	70	-71	-77	1.38E-6	3.12E-6	7.06E-6
Ovarian Cancer															
IGROV1	0.358	1.942	1.857	1.724	1.381	0.151	0.195	95	86	65	-58	-46	1.32E-6	3.37E-6	.
OVCAR-3	0.547	2.127	2.115	2.027	0.609	0.342	0.186	99	94	4	-38	-66	3.06E-7	1.24E-6	2.74E-5
OVCAR-4	0.555	1.324	1.297	1.177	0.885	0.038	0.061	97	81	43	-93	-89	6.52E-7	2.07E-6	4.81E-6
OVCAR-5	0.528	1.721	1.564	1.499	1.488	0.223	0.093	87	81	80	-58	-82	1.66E-6	3.82E-6	8.79E-6
OVCAR-8	0.418	2.376	2.336	2.309	1.940	0.498	0.315	98	97	78	4	-25	2.38E-6	1.39E-5	> 1.00E-4
NCI/ADR-RES	0.621	2.243	2.243	2.209	1.925	0.526	0.552	100	98	80	-15	-11	2.08E-6	6.92E-6	> 1.00E-4
SK-OV-3	0.927	2.214	2.277	2.220	2.184	1.075	0.239	105	100	98	11	-74	3.57E-6	1.36E-5	5.22E-5
Renal Cancer															
786-0	0.623	2.736	2.687	2.570	2.497	0.836	0.509	98	92	89	10	-18	3.10E-6	2.27E-5	> 1.00E-4
A498	1.828	2.878	2.855	2.746	2.600	0.798	0.231	98	87	74	-56	-87	1.52E-6	3.68E-6	8.94E-6
ACHN	0.320	1.628	1.611	1.508	1.340	0.292	0.048	99	91	78	-9	-85	2.10E-6	7.90E-6	3.46E-5
CAKI-1	0.613	2.536	2.395	2.350	2.200	0.642	0.137	93	90	83	1	-78	2.52E-6	1.04E-5	4.47E-5
RXF 393	1.197	1.740	1.716	1.687	1.644	0.562	0.227	95	90	82	-53	-81	1.73E-6	4.05E-6	9.49E-6
SN12C	0.664	2.693	2.505	2.536	2.165	0.283	0.053	91	92	74	-57	-92	1.52E-6	3.66E-6	8.79E-6
TK-10	0.733	2.290	2.251	2.184	2.266	0.955	0.326	97	93	98	14	-56	3.76E-6	1.60E-5	8.32E-5
UO-31	0.630	1.981	1.811	1.770	1.705	0.557	0.011	87	84	80	-12	-98	2.11E-6	7.46E-6	2.77E-5
Prostate Cancer															
PC-3	0.511	2.361	2.274	2.304	1.910	0.578	0.365	95	97	76	4	-29	2.27E-6	1.29E-5	> 1.00E-4
DU-145	0.346	1.759	1.783	1.747	1.590	0.311	0.053	102	99	88	-10	-85	2.44E-6	7.89E-6	3.43E-5
Breast Cancer															
MCF7	0.309	1.888	1.710	1.652	1.344	0.114	0.103	89	85	66	-63	-67	1.32E-6	3.23E-6	7.91E-6
MDA-MB-231/ATCC	0.656	1.662	1.634	1.510	1.404	0.492	0.492	97	85	74	-25	-25	1.76E-6	5.60E-6	> 1.00E-4
HS 578T	0.915	1.961	1.914	1.947	1.918	0.974	0.811	95	99	96	6	-11	3.22E-6	2.15E-5	> 1.00E-4
BT-549	1.174	2.620	2.535	2.496	2.300	0.892	0.088	94	91	78	-24	-93	1.88E-6	5.81E-6	2.39E-5
T-47D	1.075	2.782	2.651	2.555	2.443	0.718	0.674	92	87	80	-33	-37	1.84E-6	5.09E-6	> 1.00E-4
MDA-MB-468	0.915	1.605	1.562	1.524	1.298	0.370	0.277	94	88	56	-60	-70	1.12E-6	3.04E-6	8.25E-6

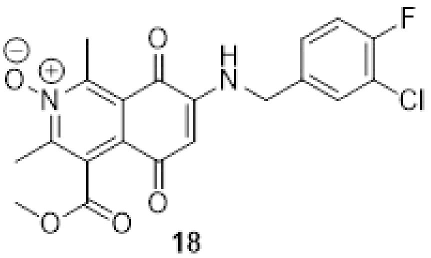

18

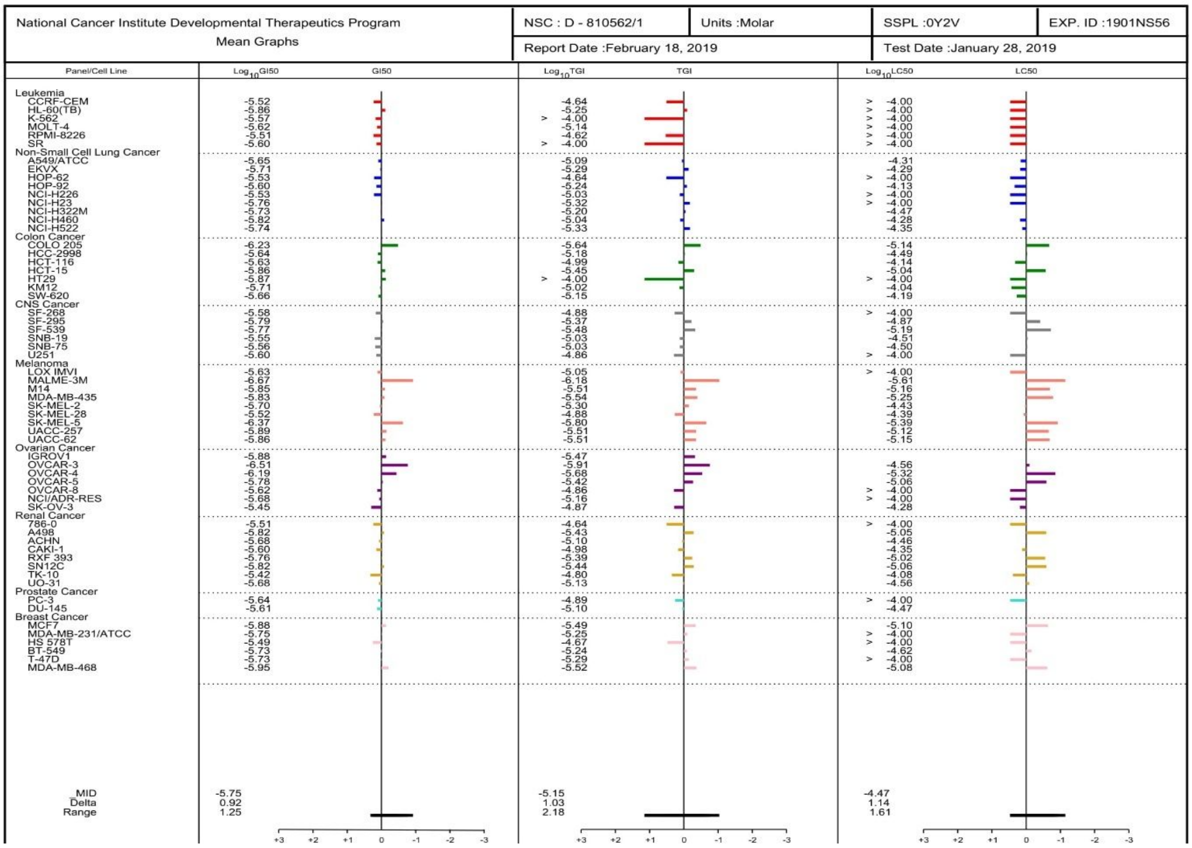

Mean of GI_{50} across 60 cell lines for compound **18** as Log_{10} Concentration (SD): -5.745 (±0.24)

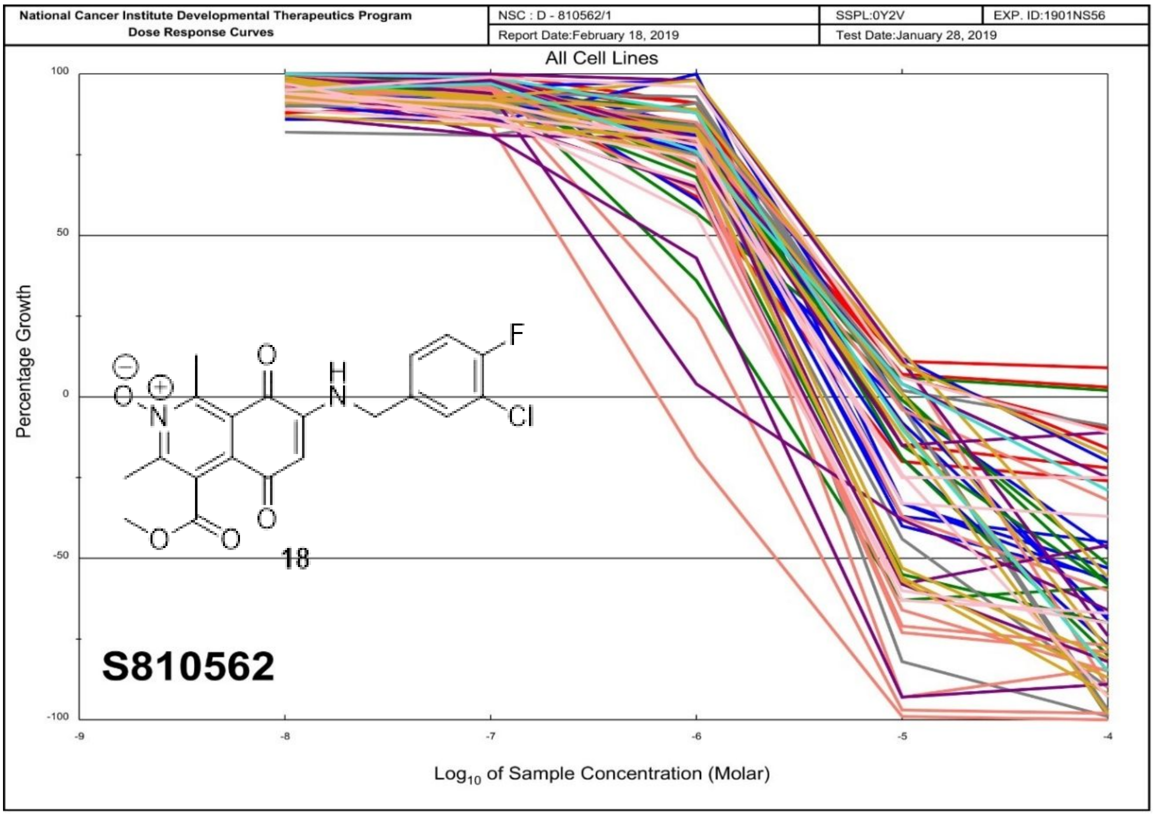

Figure S46** NCI Five Dose data cell line comparison summary for compound **18

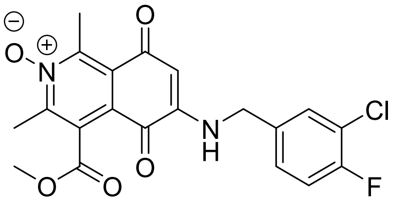

19

Developmental Therapeutics Program One Dose Mean Graph	NSC: D-815404 / 1	Conc: 1.00E-5 Molar	Test Date: May 20, 2019
	Experiment ID: 1905OS11		Report Date: Jun 10, 2019

Panel/Cell Line	Growth Percent
**Leukemia**	
CCRF-CEM	-2.70
HL-60(TB)	-28.88
K-562	-7.41
MOLT-4	-35.58
RPMI-8226	-32.70
SR	-1.19
**Non-Small Cell Lung Cancer**	
A549/ATCC	-47.37
EKVX	-63.27
HOP-62	-51.91
HOP-92	-42.56
NCI-H23	-65.87
NCI-H322M	-38.92
NCI-H460	-38.68
NCI-H522	-39.44
**Colon Cancer**	
COLO 205	-60.65
HCC-2998	-59.25
HCT-116	-48.58
HCT-15	-70.40
HT29	3.68
KM12	-16.54
SW-620	-29.63
**CNS Cancer**	
SF-268	-9.98
SF-295	-97.48
SF-539	-89.69
SNB-19	-48.46
SNB-75	-96.94
U251	-21.74
**Melanoma**	
LOX IMVI	-64.51
MALME-3M	-98.30
M14	-88.44
MDA-MB-435	-96.91
SK-MEL-2	-42.82
SK-MEL-28	-11.76
SK-MEL-5	-97.69
UACC-257	-82.75
UACC-62	-70.82
**Ovarian Cancer**	
IGROV1	-55.25
OVCAR-3	-41.36
OVCAR-5	-91.81
OVCAR-8	-21.24
NCI/ADR-RES	-18.61
SK-OV-3	-5.41
**Renal Cancer**	
786-0	3.92
ACHN	-38.40
CAKI-1	-16.15
RXF 393	-41.10
SN12C	-85.69
TK-10	21.19
UO-31	-49.52
**Prostate Cancer**	
PC-3	-1.13
DU-145	-98.99
**Breast Cancer**	
MCF7	-61.86
MDA-MB-231/ATCC	-25.58
HS 578T	-24.23
BT-549	-96.86
T-47D	-41.93
MDA-MB-468	-70.53
Mean	-46.61
Delta	52.38
Range	120.18

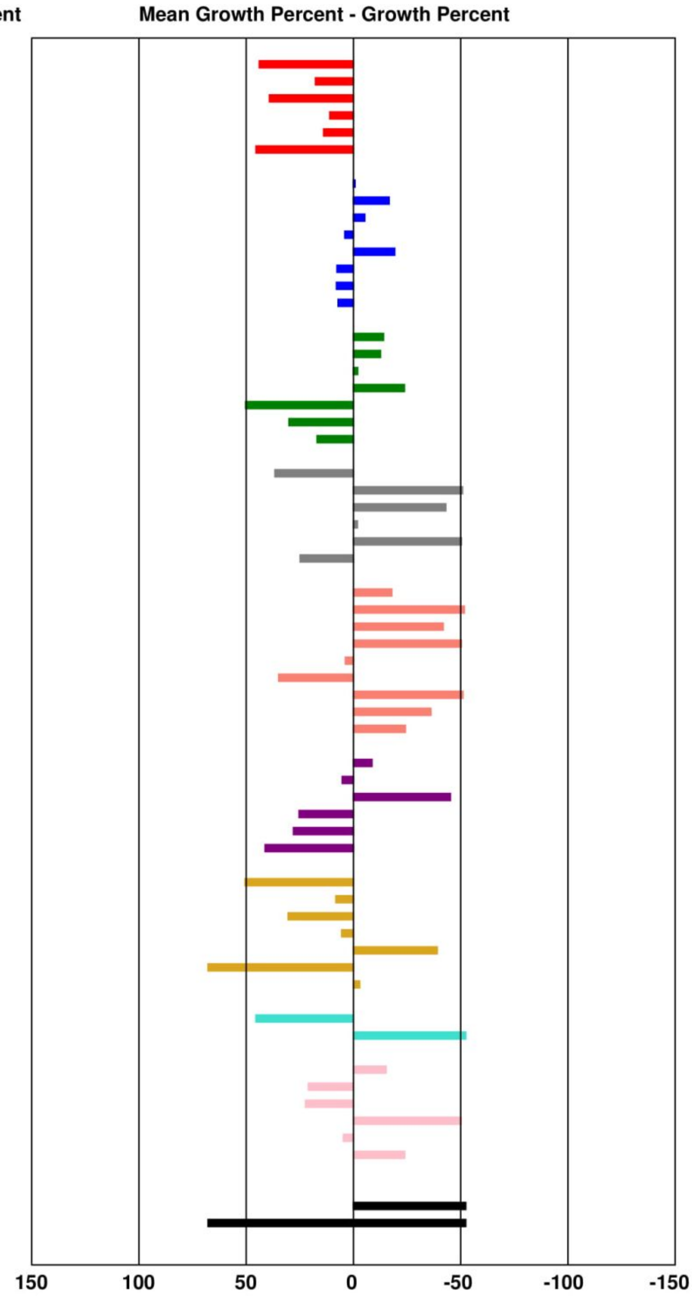

Figure S47 *NCI One Dose (10 μM) data for compound* ***19***

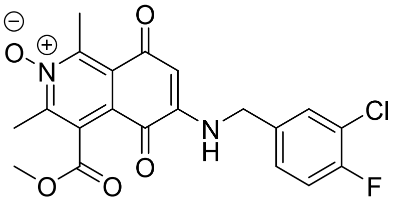

19

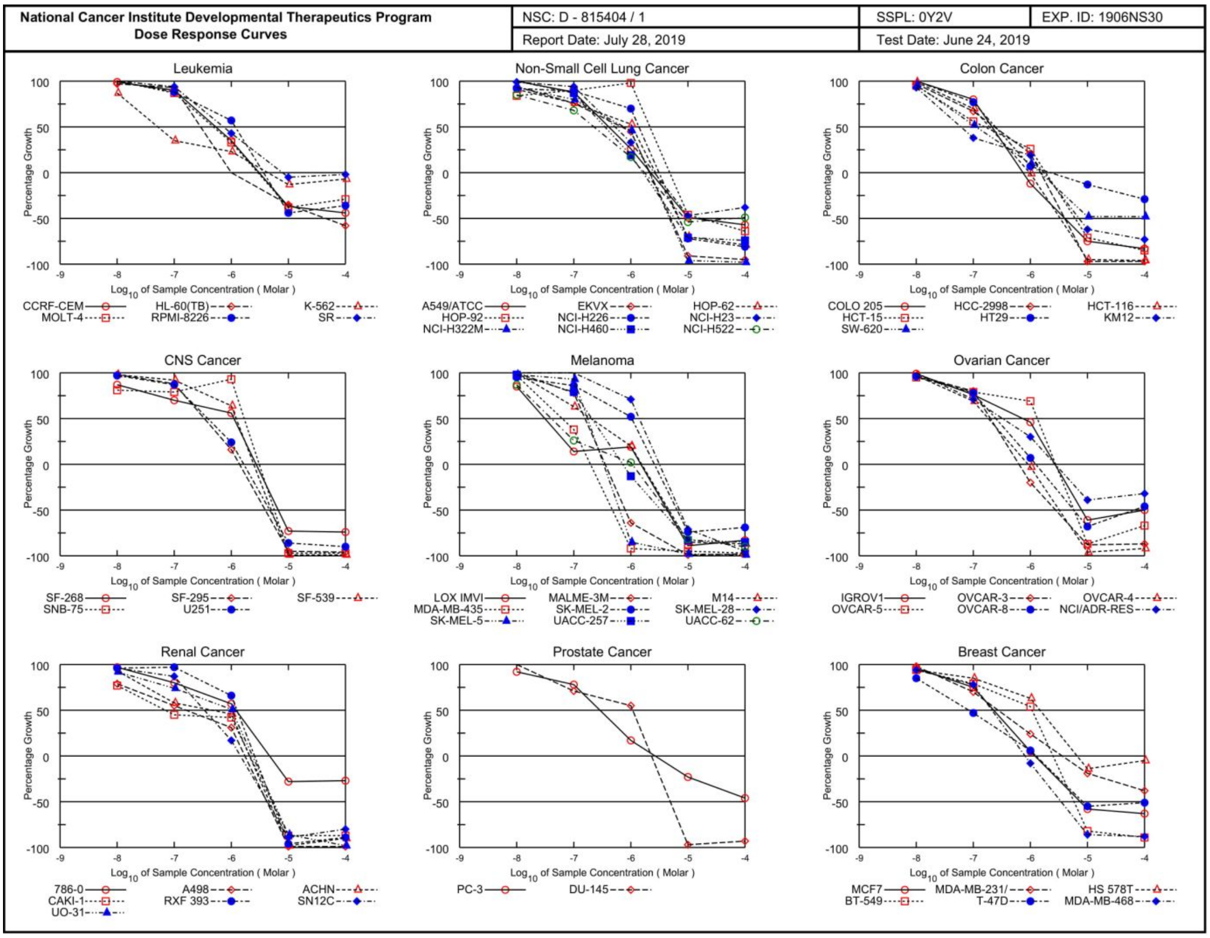

Figure S48** NCI Five Dose response summary data for compound **19

Table S16** NCI Five Dose raw data for compound **19

National Cancer Institute Developmental Therapeutics Program
In-Vitro Testing Results

NSC : D - 815404 / 1	Experiment ID : 1906NS30	Test Type : 08	Units : Molar
Report Date : July 28, 2019	Test Date : June 24, 2019	QNS :	MC :
COMI : RK 6.3.9.1	Stain Reagent : SRB Dual-Pass Related	SSPL : 0Y2V	

Panel/Cell Line	Time Zero	Ctrl	Mean Optical Densities -8.0	-7.0	-6.0	-5.0	-4.0	Percent Growth -8.0	-7.0	-6.0	-5.0	-4.0	GI50	TGI	LC50
**Leukemia**															
CCRF-CEM	0.555	2.292	2.280	2.124	1.157	0.352	0.312	99	90	35	-37	-44	5.30E-7	3.06E-6	> 1.00E-4
HL-60(TB)	0.970	3.231	3.166	3.064	0.967	0.631	0.408	97	93	.	-35	-58	2.87E-7	9.91E-7	4.51E-5
K-562	0.175	1.302	1.152	0.568	0.436	0.153	0.163	87	35	23	-13	-7	5.10E-8	4.40E-6	> 1.00E-4
MOLT-4	0.690	2.644	2.676	2.381	1.331	0.425	0.488	102	87	33	-38	-29	4.78E-7	2.88E-6	> 1.00E-4
RPMI-8226	0.896	2.668	2.667	2.462	1.907	0.504	0.578	100	88	57	-44	-36	1.18E-6	3.68E-6	> 1.00E-4
SR	0.401	1.217	1.252	1.168	0.748	0.382	0.392	104	94	43	-5	-2	7.16E-7	7.90E-6	> 1.00E-4
**Non-Small Cell Lung Cancer**															
A549/ATCC	0.290	1.839	1.847	1.660	0.695	0.148	0.126	101	88	26	-49	-57	4.13E-7	2.23E-6	1.37E-5
EKVX	0.554	1.801	1.723	1.503	1.118	0.053	0.029	94	76	45	-91	-95	7.01E-7	2.15E-6	5.03E-6
HOP-62	0.711	1.825	1.725	1.554	1.301	0.213	0.150	91	76	53	-70	-79	1.06E-6	2.69E-6	6.87E-6
HOP-92	1.036	1.570	1.486	1.517	1.559	0.564	0.376	84	90	98	-46	-64	2.16E-6	4.81E-6	1.75E-5
NCI-H226	1.126	2.489	2.375	2.335	2.082	0.315	0.215	92	89	70	-72	-81	1.38E-6	3.11E-6	7.00E-6
NCI-H23	0.613	1.938	1.928	1.863	1.046	0.328	0.378	99	94	33	-47	-38	5.23E-7	2.58E-6	> 1.00E-4
NCI-H322M	0.743	2.017	1.934	1.766	1.335	0.027	0.014	93	80	46	-96	-98	7.86E-7	2.11E-6	4.73E-6
NCI-H460	0.287	2.898	2.965	2.555	0.770	0.083	0.076	103	87	19	-71	-74	3.46E-7	1.61E-6	5.82E-6
NCI-H522	0.820	2.327	2.107	1.849	1.077	0.379	0.420	85	68	17	-54	-49	2.27E-7	1.74E-6	.
**Colon Cancer**															
COLO 205	0.585	2.207	2.232	1.881	0.513	0.148	0.098	102	80	-12	-75	-83	2.11E-7	7.34E-7	4.01E-6
HCC-2998	0.932	2.634	2.584	2.080	1.294	0.032	0.032	97	67	21	-97	-97	2.39E-7	1.51E-6	4.02E-6
HCT-116	0.244	1.914	1.895	1.412	0.241	0.013	0.010	99	70	-1	-95	-96	1.91E-7	9.61E-7	3.32E-6
HCT-15	0.278	2.075	1.977	1.283	0.746	0.081	0.043	95	56	26	-71	-85	1.58E-7	1.86E-6	6.09E-6
HT29	0.208	1.618	1.685	1.297	0.301	0.181	0.149	105	77	7	-13	-29	2.43E-7	2.15E-6	> 1.00E-4
KM12	0.579	2.940	2.778	1.488	1.035	0.220	0.155	93	38	19	-62	-73	6.15E-8	1.73E-6	7.12E-6
SW-620	0.258	2.054	1.980	1.190	0.444	0.134	0.133	96	52	10	-48	-48	1.11E-7	1.50E-6	> 1.00E-4
**CNS Cancer**															
SF-268	0.838	2.470	2.261	1.988	1.747	0.223	0.215	87	70	56	-73	-74	1.11E-6	2.70E-6	6.59E-6
SF-295	0.781	2.841	2.800	2.601	1.118	0.036	0.028	98	88	16	-95	-96	3.41E-7	1.40E-6	3.92E-6
SF-539	1.054	2.820	2.789	2.678	2.176	0.009	0.006	98	92	64	-99	-99	1.21E-6	2.46E-6	4.98E-6
SNB-75	1.027	1.755	1.619	1.605	1.705	0.030	0.032	81	79	93	-97	-97	1.69E-6	3.09E-6	5.66E-6
U251	0.272	1.521	1.488	1.362	0.567	0.039	0.028	97	87	24	-86	-90	3.85E-7	1.64E-6	4.72E-6
**Melanoma**															
LOX IMVI	0.496	2.745	2.416	0.818	0.917	0.056	0.084	85	14	19	-89	-83	3.14E-8	1.49E-6	4.36E-6
MALME-3M	0.763	1.612	1.636	1.428	0.278	0.010	0.016	103	78	-64	-99	-98	1.58E-7	3.56E-7	8.02E-7
M14	0.430	1.655	1.694	1.199	0.677	0.064	0.056	103	63	20	-85	-87	1.99E-7	1.55E-6	4.64E-6
MDA-MB-435	0.512	2.494	2.439	1.265	0.044	0.027	0.018	97	38	-92	-95	-97	6.27E-8	1.97E-7	4.78E-7
SK-MEL-2	1.127	2.574	2.500	2.378	1.882	0.293	0.344	95	86	52	-74	-69	1.04E-6	2.59E-6	6.45E-6
SK-MEL-28	0.772	1.994	2.051	2.015	1.635	0.223	0.042	105	102	71	-71	-95	1.40E-6	3.15E-6	7.09E-6
SK-MEL-5	0.784	3.133	3.080	2.961	0.115	0.014	0.005	98	93	-85	-98	-99	1.74E-7	3.31E-7	6.33E-7
UACC-257	0.739	1.807	1.781	1.586	0.647	0.126	0.113	98	79	-13	-83	-85	2.09E-7	7.31E-7	3.40E-6
UACC-62	0.975	2.644	2.421	1.415	1.015	0.179	0.098	87	26	2	-82	-90	4.05E-8	1.07E-6	4.20E-6
**Ovarian Cancer**															
IGROV1	0.547	2.188	2.177	1.795	1.306	0.215	0.272	99	76	46	-61	-50	7.49E-7	2.71E-6	7.94E-6
OVCAR-3	0.469	1.572	1.538	1.349	0.376	0.057	0.059	97	80	-20	-88	-87	1.99E-7	6.31E-7	2.77E-6
OVCAR-4	0.560	1.552	1.520	1.245	0.544	0.024	0.047	97	69	-3	-96	-92	1.84E-7	9.13E-7	3.22E-6
OVCAR-5	0.600	1.647	1.594	1.426	1.318	0.078	0.201	95	79	69	-87	-67	1.32E-6	2.76E-6	5.78E-6
OVCAR-8	0.444	2.325	2.247	1.908	0.585	0.142	0.242	96	78	7	-68	-46	2.49E-7	1.26E-6	.
NCI/ADR-RES	0.563	2.064	2.067	1.626	1.010	0.344	0.383	100	71	30	-39	-32	3.21E-7	2.71E-6	> 1.00E-4
**Renal Cancer**															
786-0	0.921	2.660	2.607	2.316	1.915	0.667	0.673	97	80	57	-28	-27	1.21E-6	4.73E-6	> 1.00E-4
A498	1.553	2.116	1.998	1.863	1.728	0.015	0.009	79	55	31	-99	-99	1.62E-7	1.73E-6	4.20E-6
ACHN	0.354	1.698	1.610	1.128	0.966	0.008	0.035	93	58	46	-98	-90	4.27E-7	2.08E-6	4.64E-6
CAKI-1	0.772	2.663	2.227	1.632	1.568	0.100	0.102	77	45	42	-87	-87	7.18E-8	2.12E-6	5.16E-6
RXF 393	0.883	1.651	1.621	1.628	1.392	0.040	0.101	96	97	66	-96	-89	1.26E-6	2.57E-6	5.23E-6
SN12C	0.558	1.950	1.900	1.775	0.791	0.062	0.109	96	87	17	-89	-80	3.38E-7	1.44E-6	4.28E-6
UO-31	0.542	1.659	1.570	1.364	1.112	0.074	0.009	92	74	51	-86	-98	1.02E-6	2.35E-6	5.44E-6
**Prostate Cancer**															
PC-3	0.573	1.595	1.512	1.374	0.751	0.444	0.311	92	78	17	-23	-46	2.92E-7	2.72E-6	> 1.00E-4
DU-145	0.410	1.745	1.806	1.359	1.139	0.013	0.028	105	71	55	-97	-93	1.07E-6	2.29E-6	4.90E-6
**Breast Cancer**															
MCF7	0.467	2.408	2.325	1.926	0.567	0.194	0.171	96	75	5	-58	-63	2.29E-7	1.20E-6	7.36E-6
MDA-MB-231/ATCC	0.775	1.830	1.806	1.509	1.033	0.627	0.479	98	70	24	-19	-38	2.71E-7	3.63E-6	> 1.00E-4
HS 578T	0.869	1.985	1.917	1.823	1.567	0.750	0.826	94	85	63	-14	-5	1.46E-6	6.61E-6	> 1.00E-4
BT-549	1.031	1.911	1.856	1.730	1.507	0.190	0.116	94	79	54	-82	-89	1.07E-6	2.50E-6	5.85E-6
T-47D	0.723	1.620	1.483	1.141	0.774	0.324	0.355	85	47	6	-55	-51	8.14E-8	1.24E-6	8.20E-6
MDA-MB-468	0.732	1.340	1.303	1.205	0.674	0.103	0.092	94	78	-8	-86	-88	2.11E-7	8.07E-7	3.46E-6

Log10 Concentration

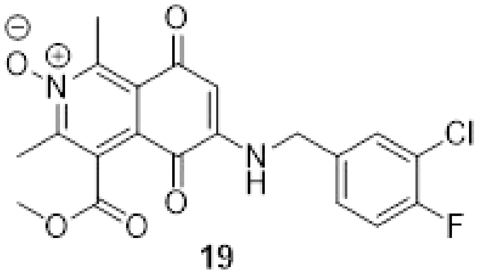

19

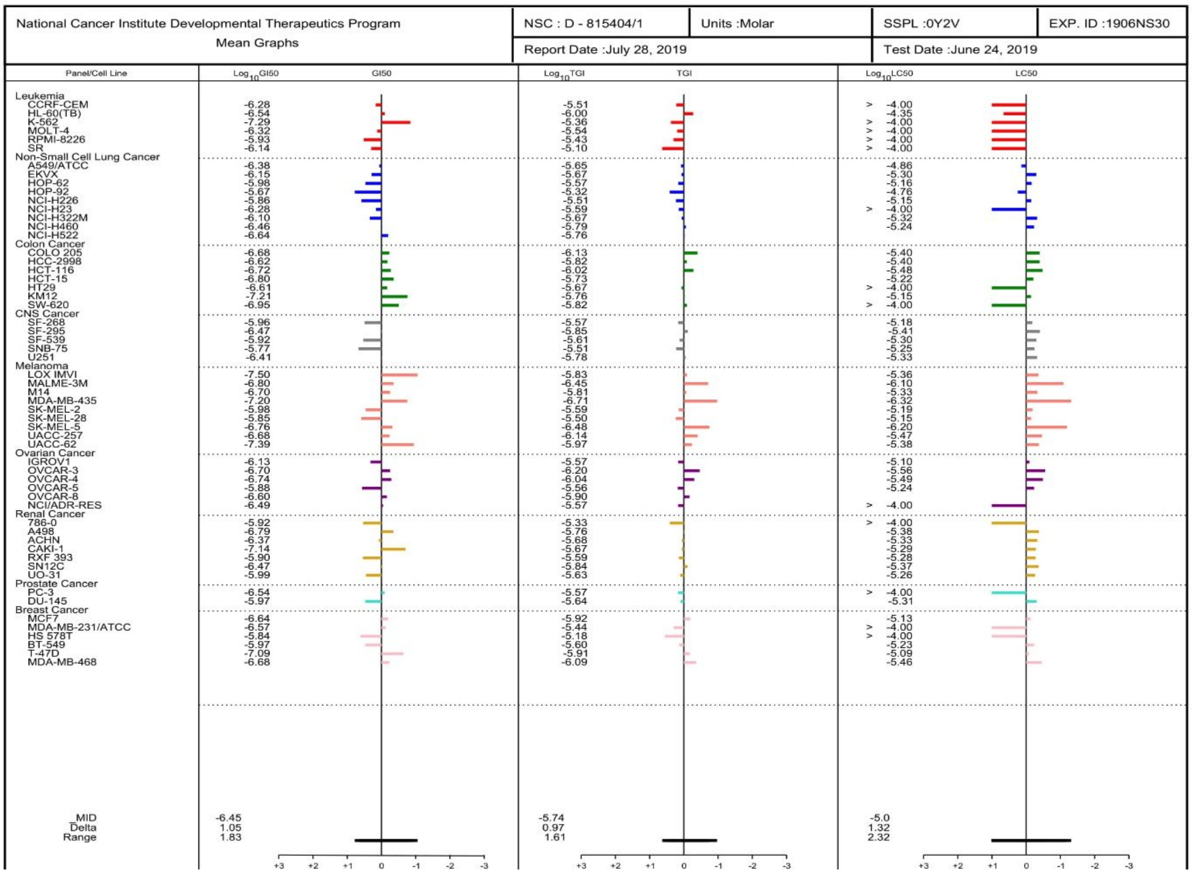

Mean of GI_{50} across 58 cell lines for compound **19** as Log_{10} Concentration (SD): -6.538 (±0.49)

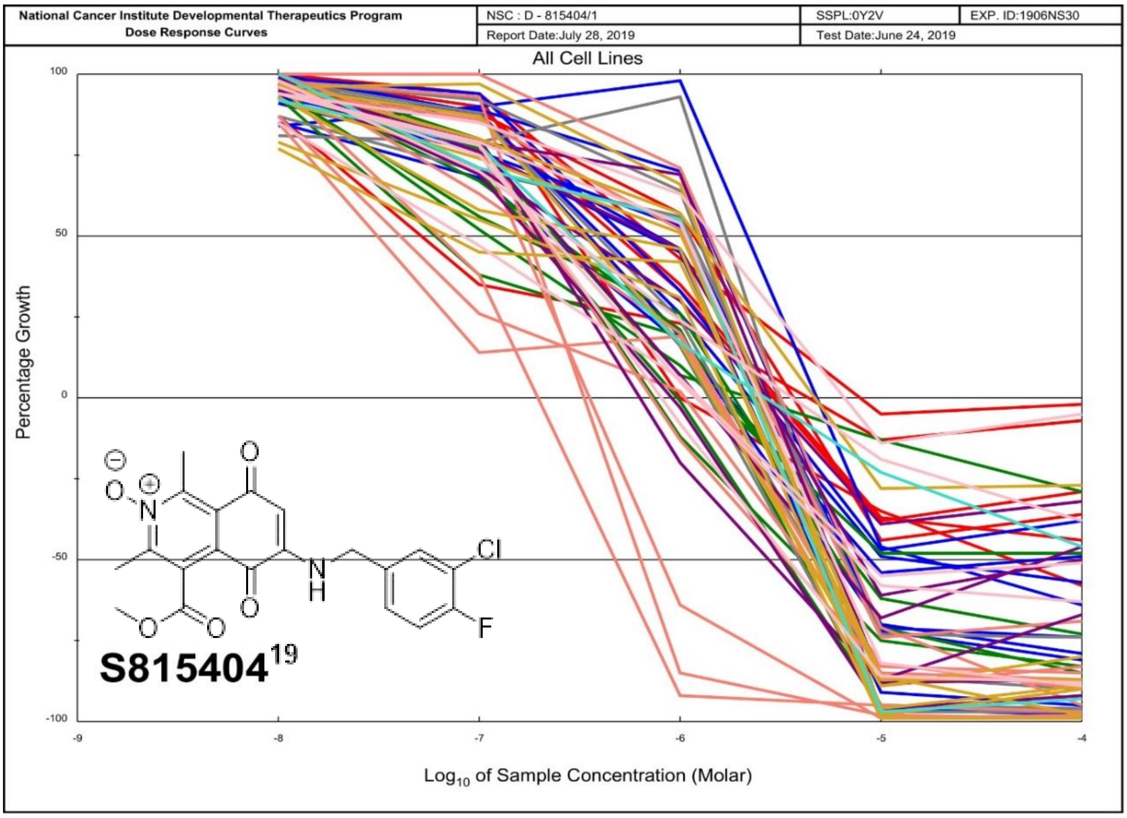

Figure S49 *NCI Five Dose data cell line comparison summary for compound **19***

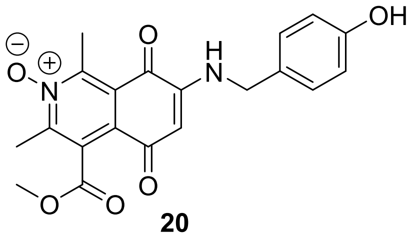

20

Developmental Therapeutics Program	NSC: D-815371 / 1	Conc: 1.00E-5 Molar	Test Date: May 20, 2019
One Dose Mean Graph	Experiment ID: 1905OS11		Report Date: Jun 10, 2019

Panel/Cell Line	Growth Percent
Leukemia	
CCRF-CEM	6.08
HL-60(TB)	-8.85
K-562	6.74
MOLT-4	-1.35
RPMI-8226	-2.15
SR	11.08
Non-Small Cell Lung Cancer	
A549/ATCC	4.46
EKVX	-34.47
HOP-62	-7.28
HOP-92	34.05
NCI-H23	-57.37
NCI-H322M	0.62
NCI-H460	-3.38
NCI-H522	-33.21
Colon Cancer	
COLO 205	0.42
HCC-2998	-20.08
HCT-116	-6.79
HCT-15	-43.45
HT29	3.68
KM12	2.07
SW-620	-3.33
CNS Cancer	
SF-268	-9.31
SF-295	-9.04
SF-539	-59.72
SNB-19	-28.51
SNB-75	-29.49
U251	-0.07
Melanoma	
LOX IMVI	-89.59
MALME-3M	-98.54
M14	-76.49
MDA-MB-435	-96.49
SK-MEL-2	-12.53
SK-MEL-28	-3.61
SK-MEL-5	-99.66
UACC-257	-62.36
UACC-62	-73.80
Ovarian Cancer	
IGROV1	-45.03
OVCAR-3	-33.14
OVCAR-4	-97.41
OVCAR-5	-16.97
OVCAR-8	5.26
NCI/ADR-RES	-6.46
SK-OV-3	22.32
Renal Cancer	
786-0	7.96
ACHN	8.06
CAKI-1	7.36
RXF 393	-4.53
SN12C	-89.93
TK-10	74.09
UO-31	6.95
Prostate Cancer	
PC-3	7.51
DU-145	-69.18
Breast Cancer	
MCF7	-63.44
MDA-MB-231/ATCC	-10.33
HS 578T	-22.46
BT-549	34.45
T-47D	-7.43
MDA-MB-468	-44.22
Mean	-21.35
Delta	78.31
Range	173.75

Mean Growth Percent - Growth Percent

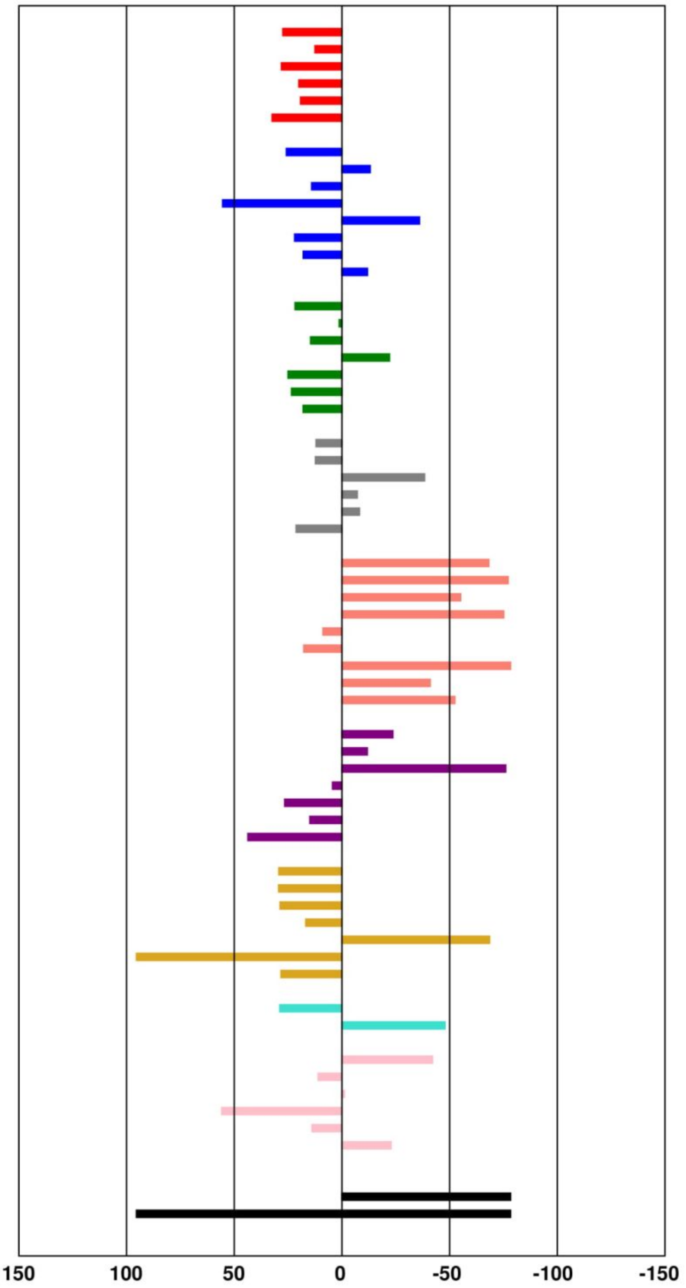

Figure S50 *NCI One Dose (10 µM) data for compound **20***

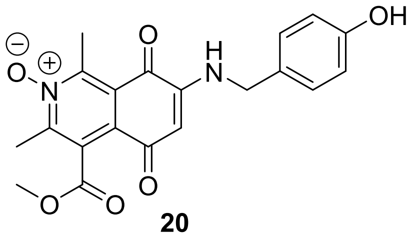

20

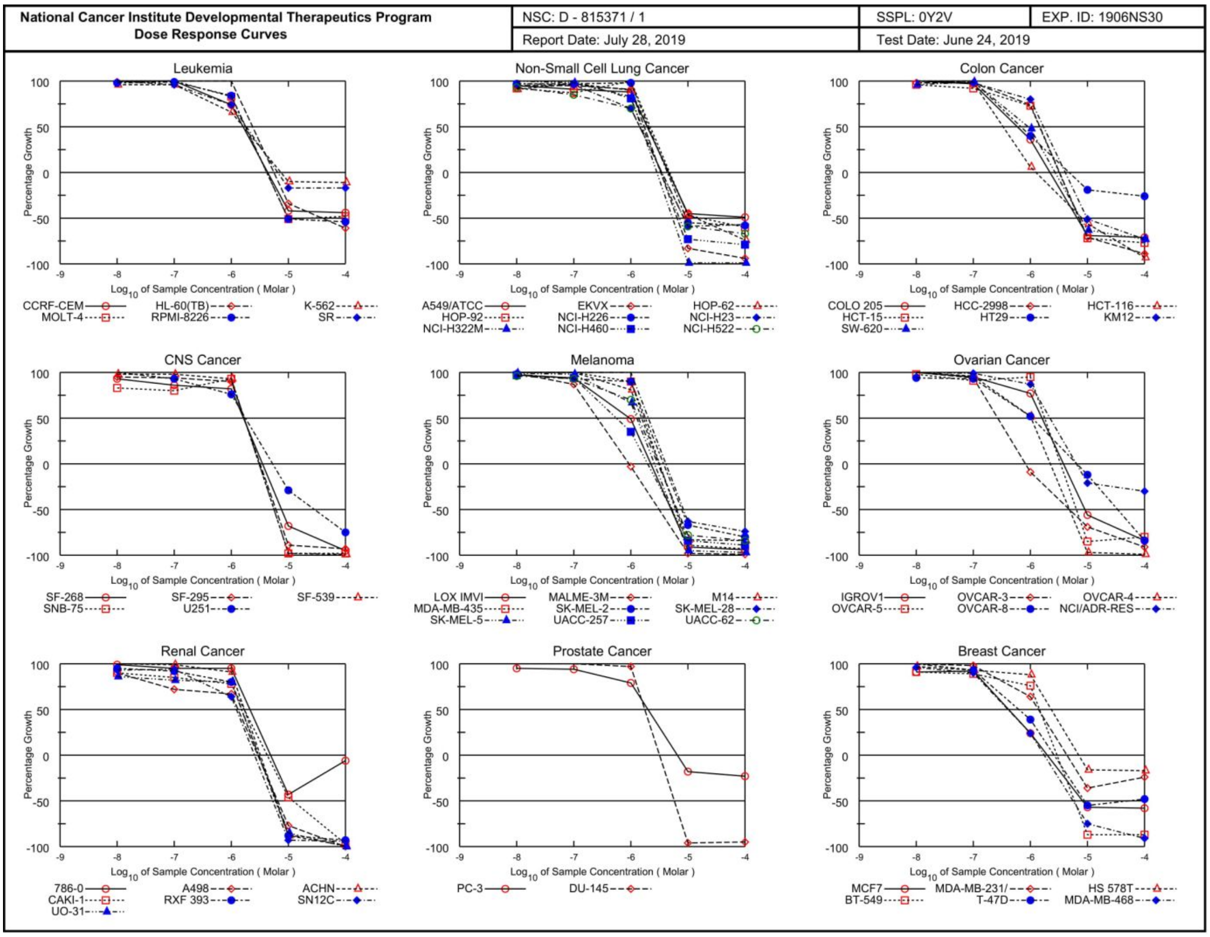

Figure S51 *NCI Five Dose response summary data for compound **20***

Table S17 *NCI Five Dose raw data for compound* ***20***

National Cancer Institute Developmental Therapeutics Program
In-Vitro Testing Results

NSC : D - 815371 / 1	Experiment ID : 1906NS30	Test Type : 08	Units : Molar
Report Date : July 28, 2019	Test Date : June 24, 2019	QNS :	MC :
COMI : RK-CC-007	Stain Reagent : SRB Dual-Pass Related	SSPL : 0Y2V	

			Log10 Concentration												
	Time		Mean Optical Densities					Percent Growth							
Panel/Cell Line	Zero	Ctrl	-8.0	-7.0	-6.0	-5.0	-4.0	-8.0	-7.0	-6.0	-5.0	-4.0	GI50	TGI	LC50
**Leukemia**															
CCRF-CEM	0.555	2.401	2.413	2.609	1.914	0.324	0.309	101	111	74	-42	-44	1.60E-6	4.35E-6	> 1.00E-4
HL-60(TB)	0.970	3.106	3.083	3.104	3.186	0.644	0.378	99	100	104	-34	-61	2.46E-6	5.69E-6	3.94E-5
K-562	0.175	1.458	1.412	1.413	1.025	0.158	0.156	96	96	66	-10	-11	1.63E-6	7.39E-6	> 1.00E-4
MOLT-4	0.690	2.580	2.725	2.675	2.252	0.339	0.360	108	105	83	-51	-48	1.76E-6	4.16E-6	.
RPMI-8226	0.896	2.799	2.763	2.774	2.486	0.439	0.408	98	99	84	-51	-54	1.78E-6	4.18E-6	9.83E-6
SR	0.401	1.186	1.198	1.159	0.981	0.333	0.334	102	96	74	-17	-17	1.83E-6	6.51E-6	> 1.00E-4
**Non-Small Cell Lung Cancer**															
A549/ATCC	0.290	1.888	1.806	1.749	1.692	0.161	0.147	95	91	88	-45	-49	1.93E-6	4.60E-6	> 1.00E-4
EKVX	0.554	1.902	1.836	1.835	1.785	0.093	0.031	95	95	91	-83	-94	1.73E-6	3.34E-6	6.45E-6
HOP-62	0.711	1.837	1.739	1.808	1.722	0.382	0.187	91	97	90	-46	-74	1.96E-6	4.57E-6	1.37E-5
HOP-92	1.036	1.598	1.552	1.524	1.682	0.548	0.418	92	87	115	-47	-60	2.52E-6	5.12E-6	1.70E-5
NCI-H226	1.126	2.495	2.448	2.456	2.468	0.503	0.477	97	97	98	-55	-58	2.06E-6	4.36E-6	9.23E-6
NCI-H23	0.613	1.993	1.957	1.943	1.579	0.254	0.261	97	96	70	-59	-57	1.43E-6	3.50E-6	8.58E-6
NCI-H322M	0.743	1.948	1.962	1.926	1.747	0.011	0.005	101	98	83	-99	-99	1.52E-6	2.87E-6	5.41E-6
NCI-H460	0.287	2.849	2.929	2.933	2.352	0.078	0.061	103	103	81	-73	-79	1.58E-6	3.35E-6	7.08E-6
NCI-H522	0.820	2.305	2.206	2.080	1.858	0.336	0.268	93	85	70	-59	-67	1.43E-6	3.49E-6	8.51E-6
**Colon Cancer**															
COLO 205	0.585	2.317	2.347	2.257	1.200	0.182	0.169	102	97	36	-69	-71	5.79E-7	2.19E-6	6.59E-6
HCC-2998	0.932	2.655	2.629	2.628	2.200	0.271	0.104	98	98	74	-71	-89	1.46E-6	3.23E-6	7.16E-6
HCT-116	0.244	2.217	2.241	2.184	0.360	0.108	0.018	101	98	6	-56	-93	3.33E-7	1.24E-6	8.07E-6
HCT-15	0.278	2.272	2.202	2.108	1.726	0.078	0.064	96	92	73	-72	-77	1.43E-6	3.18E-6	7.03E-6
HT29	0.208	1.724	1.837	1.843	0.817	0.170	0.155	107	108	40	-19	-26	7.15E-7	4.83E-6	> 1.00E-4
KM12	0.579	2.992	3.051	2.936	2.507	0.287	0.158	102	98	80	-51	-73	1.70E-6	4.10E-6	9.91E-6
SW-620	0.258	2.044	1.981	2.034	1.124	0.096	0.069	96	99	48	-63	-73	9.34E-7	2.72E-6	7.65E-6
**CNS Cancer**															
SF-268	0.838	2.402	2.294	2.181	2.127	0.267	0.043	93	86	82	-68	-95	1.64E-6	3.53E-6	7.58E-6
SF-295	0.781	3.054	2.945	2.918	2.819	0.085	0.056	95	94	90	-89	-93	1.67E-6	3.17E-6	6.04E-6
SF-539	1.054	2.928	2.898	2.883	2.792	0.023	0.006	98	98	93	-98	-99	1.68E-6	3.07E-6	5.61E-6
SNB-75	1.027	1.799	1.666	1.648	1.743	0.021	0.016	83	80	93	-98	-98	1.68E-6	3.06E-6	5.60E-6
U251	0.272	1.512	1.532	1.424	1.216	0.192	0.069	102	93	76	-29	-75	1.77E-6	5.26E-6	2.84E-5
**Melanoma**															
LOX IMVI	0.496	2.843	2.778	2.695	1.640	0.047	0.031	97	94	49	-91	-94	9.38E-7	2.24E-6	5.12E-6
MALME-3M	0.763	1.681	1.671	1.557	0.742	0.012	0.006	99	87	-3	-98	-99	2.56E-7	9.30E-7	3.12E-6
M14	0.430	1.854	1.855	1.945	1.589	0.073	0.069	100	106	81	-83	-84	1.55E-6	3.12E-6	6.29E-6
MDA-MB-435	0.512	2.681	2.694	2.693	2.471	0.056	0.037	101	101	90	-89	-93	1.68E-6	3.19E-6	6.05E-6
SK-MEL-2	1.127	2.540	2.508	2.442	2.394	0.367	0.221	98	93	90	-67	-80	1.79E-6	3.72E-6	7.74E-6
SK-MEL-28	0.772	2.020	2.071	2.111	2.168	0.286	0.197	104	107	112	-63	-74	2.26E-6	4.36E-6	8.42E-6
SK-MEL-5	0.784	3.231	3.218	3.189	2.425	0.039	0.027	99	98	67	-95	-97	1.27E-6	2.59E-6	5.27E-6
UACC-257	0.739	1.874	1.837	1.803	1.137	0.117	0.079	97	94	35	-84	-89	5.56E-7	1.97E-6	5.17E-6
UACC-62	0.975	2.647	2.577	2.524	2.138	0.219	0.158	96	93	70	-78	-84	1.36E-6	2.97E-6	6.50E-6
**Ovarian Cancer**															
IGROV1	0.547	2.176	2.194	2.099	1.798	0.239	0.089	101	95	77	-56	-84	1.59E-6	3.78E-6	8.97E-6
OVCAR-3	0.469	1.592	1.632	1.520	0.428	0.146	0.043	104	94	-9	-69	-91	2.66E-7	8.20E-7	4.85E-6
OVCAR-4	0.560	1.481	1.513	1.440	1.039	0.016	0.008	104	96	52	-97	-99	1.03E-6	2.23E-6	4.83E-6
OVCAR-5	0.600	1.686	1.663	1.583	1.627	0.089	0.121	98	91	95	-85	-80	1.77E-6	3.36E-6	6.37E-6
OVCAR-8	0.444	2.334	2.219	2.211	1.431	0.390	0.071	94	93	52	-12	-84	1.08E-6	6.47E-6	3.36E-5
NCI/ADR-RES	0.563	2.153	2.179	2.144	1.948	0.444	0.394	102	99	87	-21	-30	2.20E-6	6.37E-6	> 1.00E-4
**Renal Cancer**															
786-0	0.921	2.807	2.792	2.708	2.706	0.525	0.869	99	95	95	-43	-6	2.11E-6	4.87E-6	> 1.00E-4
A498	1.553	2.118	2.061	1.957	1.931	0.360	0.007	90	72	67	-77	-100	1.31E-6	2.92E-6	6.51E-6
ACHN	0.354	1.674	1.710	1.662	1.557	0.042	0.003	103	99	91	-88	-99	1.70E-6	3.22E-6	6.13E-6
CAKI-1	0.772	2.678	2.490	2.398	2.268	0.416	0.023	90	85	78	-46	-97	1.69E-6	4.26E-6	1.19E-5
RXF 393	0.883	1.713	1.672	1.650	1.544	0.104	0.064	95	92	80	-88	-93	1.50E-6	2.98E-6	5.92E-6
SN12C	0.558	1.998	1.895	1.917	1.480	0.041	0.033	93	94	64	-93	-94	1.23E-6	2.56E-6	5.34E-6
UO-31	0.542	1.616	1.463	1.418	1.405	0.080	0.002	86	82	80	-85	-100	1.52E-6	3.06E-6	6.13E-6
**Prostate Cancer**															
PC-3	0.573	1.662	1.612	1.600	1.438	0.471	0.443	95	94	79	-18	-23	2.00E-6	6.55E-6	> 1.00E-4
DU-145	0.410	1.776	1.849	1.808	1.736	0.018	0.021	105	102	97	-96	-95	1.75E-6	3.19E-6	5.79E-6
**Breast Cancer**															
MCF7	0.467	2.634	2.446	2.453	0.996	0.203	0.195	91	92	24	-57	-58	4.16E-7	2.00E-6	8.28E-6
MDA-MB-231/ATCC	0.775	1.814	1.835	1.791	1.439	0.494	0.586	102	98	64	-36	-24	1.38E-6	4.34E-6	> 1.00E-4
HS 578T	0.869	2.001	1.962	1.918	1.860	0.729	0.718	97	93	88	-16	-17	2.30E-6	6.98E-6	> 1.00E-4
BT-549	1.031	2.085	1.991	1.973	1.827	0.131	0.138	91	89	76	-87	-87	1.43E-6	2.91E-6	5.90E-6
T-47D	0.723	1.711	1.719	1.638	1.105	0.327	0.375	101	93	39	-55	-48	6.15E-7	2.59E-6	.
MDA-MB-468	0.732	1.390	1.364	1.323	0.890	0.182	0.066	96	90	24	-75	-91	4.02E-7	1.74E-6	5.58E-6

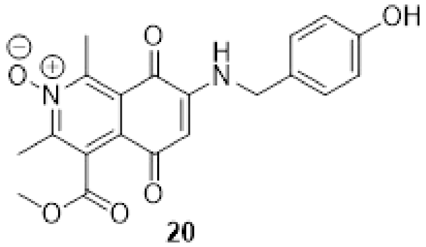

20

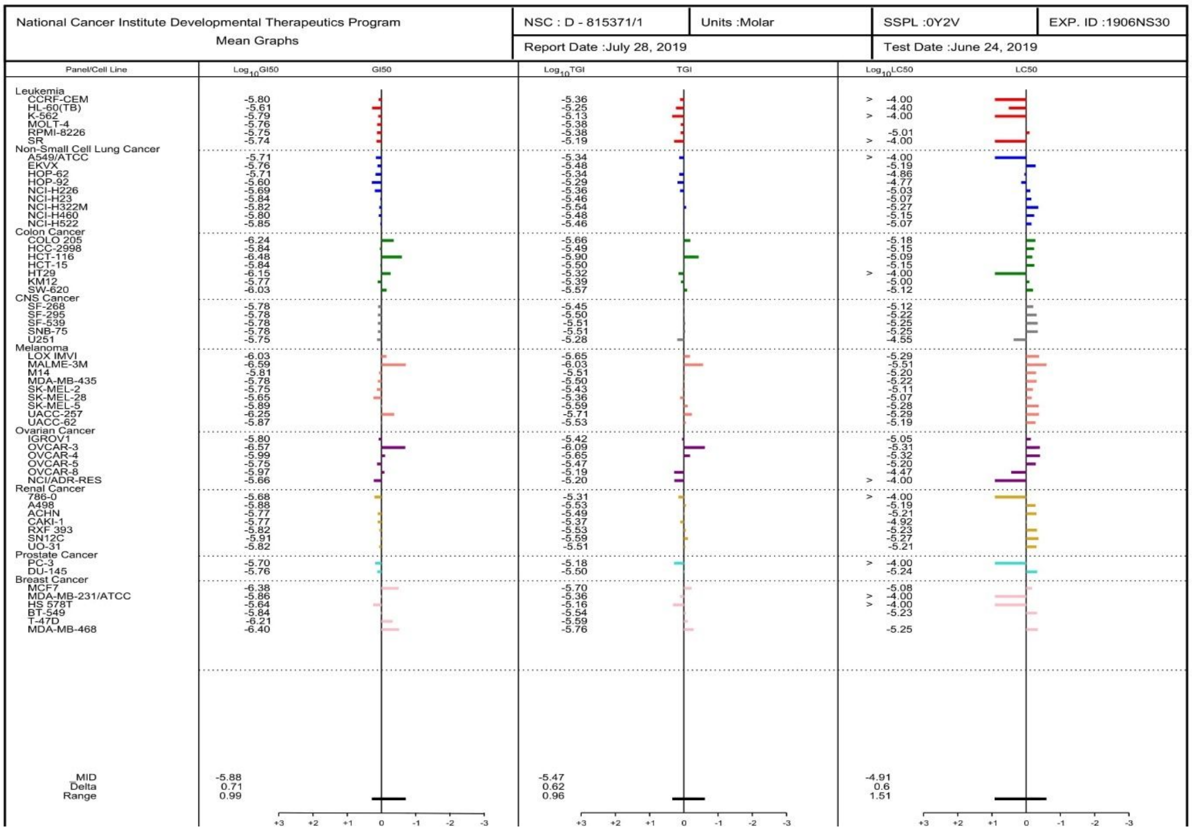

Mean of GI_{50} across 57 cell lines for compound **20** as Log_{10} Concentration (SD): -5.882 (±0.24)

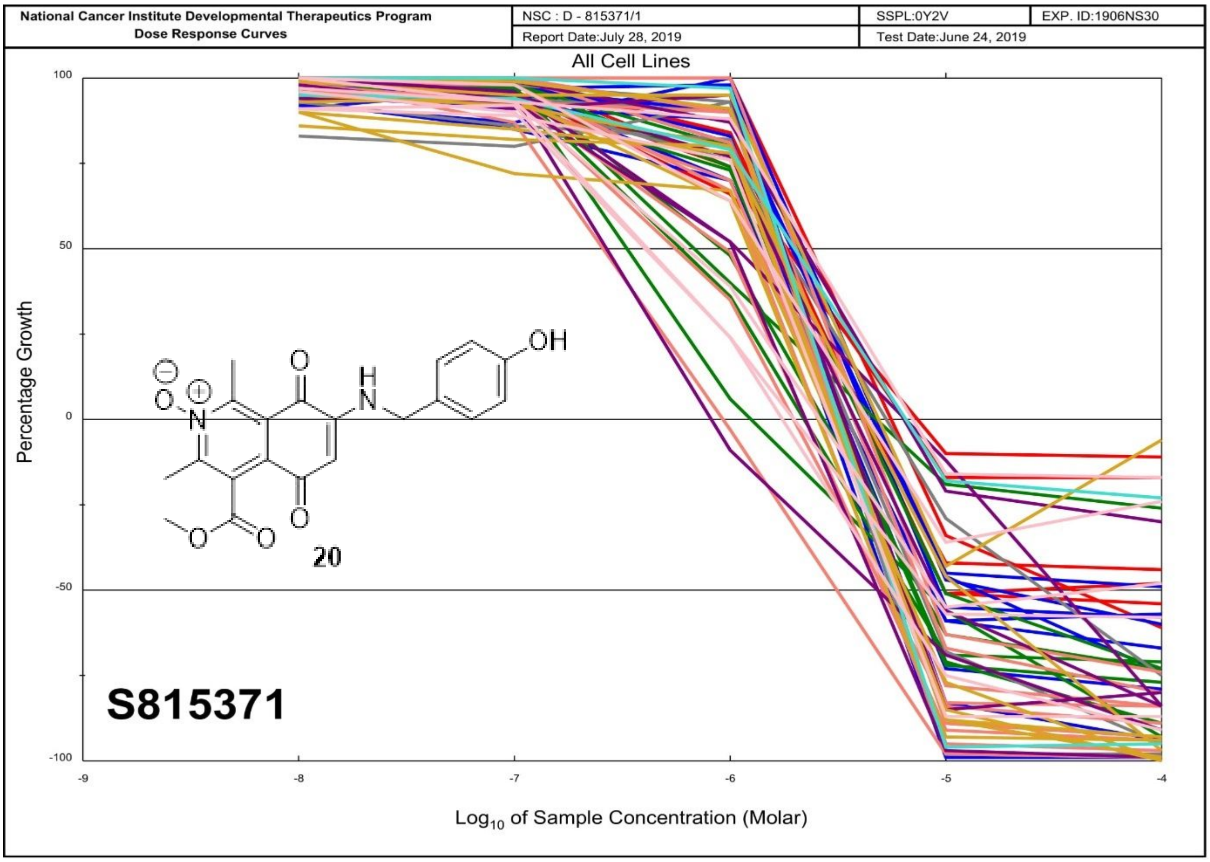

Figure S52** NCI Five Dose data cell line comparison summary for compound **20

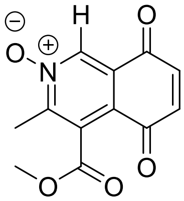

23

Developmental Therapeutics Program

One Dose Mean Graph

**NSC:** D-810563 / 1	**Conc:** 1.00E-5 Molar	**Test Date:** Dec 03, 2018	
**Experiment ID:** 1812OS40		**Report Date:** Dec 20, 2018	

Panel/Cell Line	Growth Percent
Leukemia	
HL-60(TB)	28.10
K-562	2.05
MOLT-4	-17.42
RPMI-8226	57.62
Non-Small Cell Lung Cancer	
A549/ATCC	99.95
EKVX	101.18
HOP-62	104.27
HOP-92	70.82
NCI-H226	98.06
NCI-H23	60.43
NCI-H322M	109.71
NCI-H460	98.38
NCI-H522	-69.89
Colon Cancer	
COLO 205	33.51
HCC-2998	88.09
HCT-116	-9.77
HCT-15	12.39
HT29	105.74
KM12	98.74
SW-620	0.11
CNS Cancer	
SF-268	68.69
SF-295	102.52
SF-539	83.49
SNB-19	79.76
SNB-75	124.82
U251	37.66
Melanoma	
LOX IMVI	47.27
MALME-3M	-97.14
M14	-64.49
MDA-MB-435	-81.80
SK-MEL-2	58.45
SK-MEL-28	48.96
SK-MEL-5	71.95
UACC-257	-35.06
UACC-62	0.93
Ovarian Cancer	
IGROV1	2.42
OVCAR-3	-66.28
OVCAR-4	-13.22
OVCAR-5	117.63
OVCAR-8	35.28
NCI/ADR-RES	97.12
SK-OV-3	96.97
Renal Cancer	
786-0	91.60
A498	106.15
ACHN	14.25
CAKI-1	62.09
RXF 393	57.28
SN12C	59.71
TK-10	101.12
UO-31	46.54
Prostate Cancer	
PC-3	65.80
DU-145	74.14
Breast Cancer	
MCF7	-61.98
MDA-MB-231/ATCC	49.86
HS 578T	86.30
BT-549	71.36
T-47D	-11.91
MDA-MB-468	-17.15
Mean	44.54
Delta	141.68
Range	221.96

Mean Growth Percent - Growth Percent

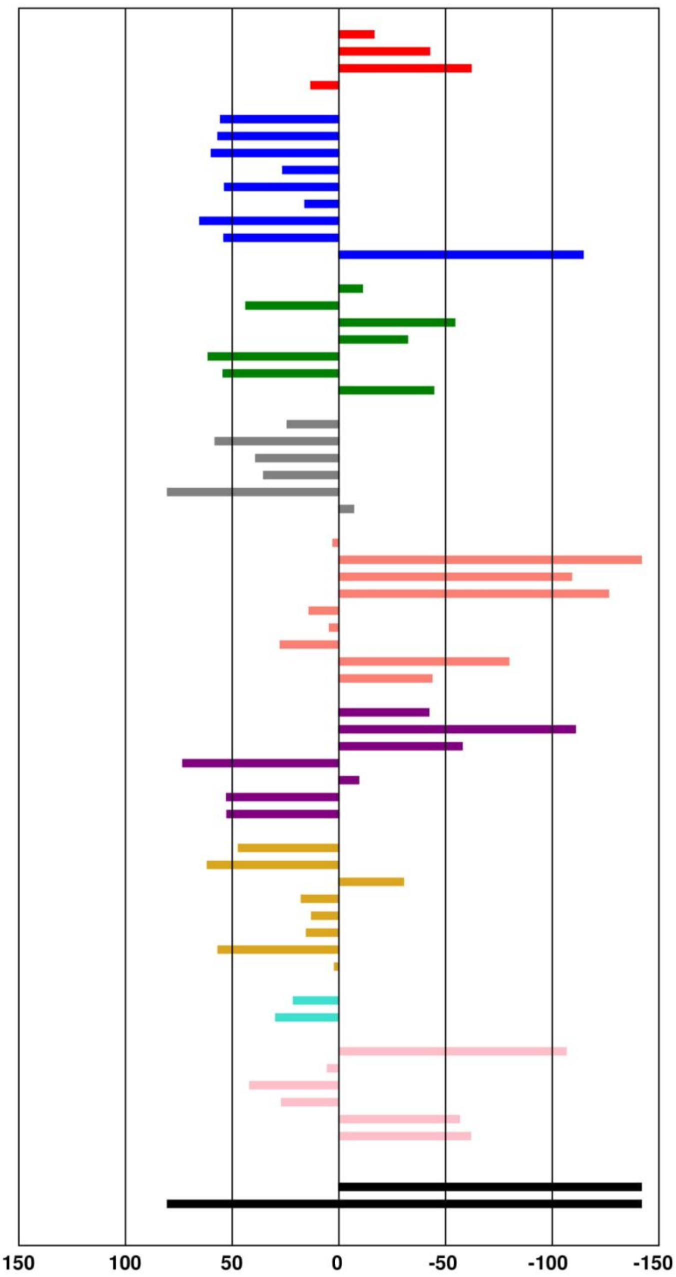

Figure S53 *NCI One Dose (10 µM) data for compound* ***23***

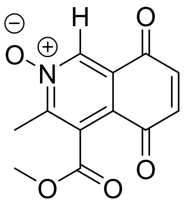

23

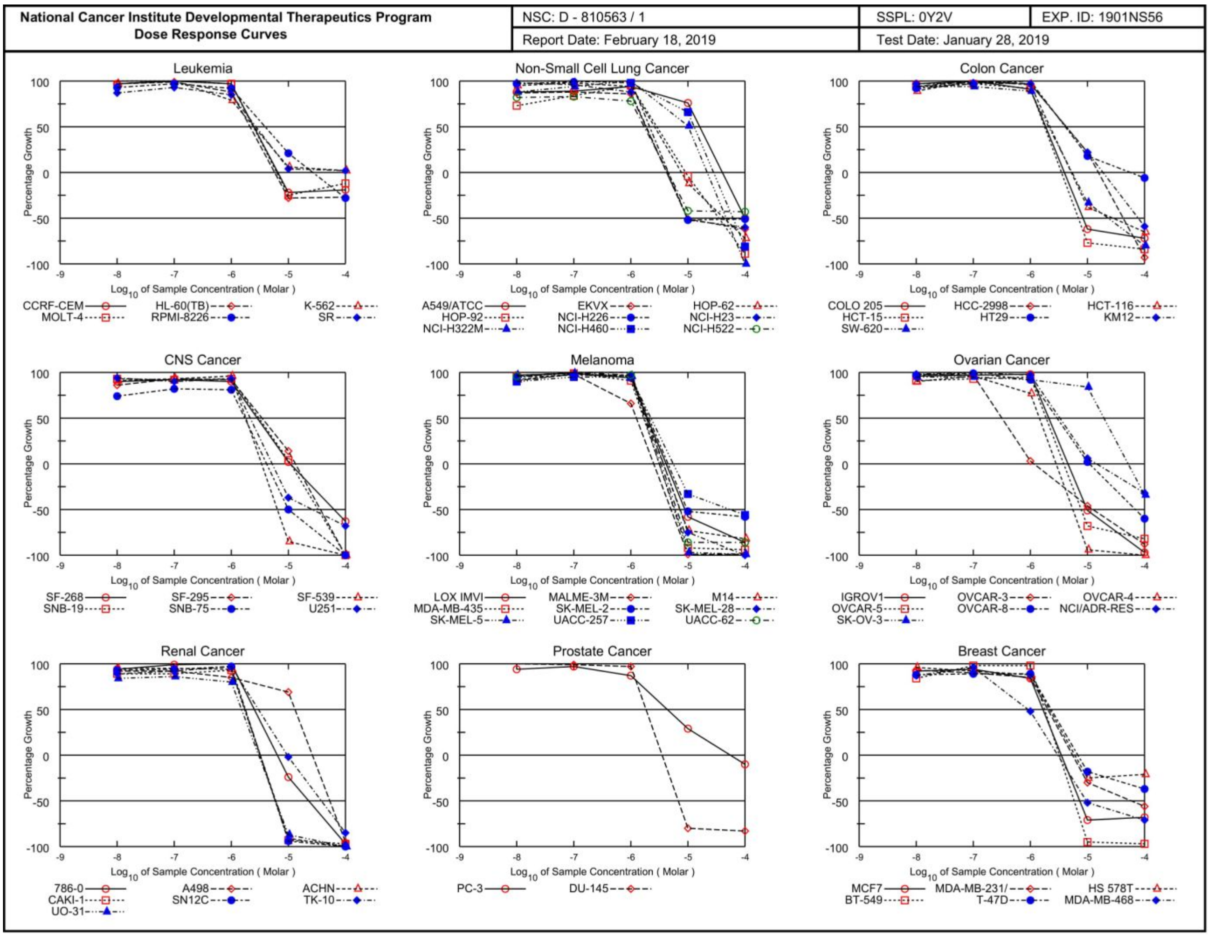

Figure S54 *NCI Five Dose response summary data for compound* ***23***

Table S18** NCI Five Dose raw data for compound **23

National Cancer Institute Developmental Therapeutics Program
In-Vitro Testing Results

NSC : D - 810563 / 1	Experiment ID : 1901NS56	Test Type : 08	Units : Molar
Report Date : February 18, 2019	Test Date : January 28, 2019	QNS :	MC :
COMI : RK 6.3.2	Stain Reagent : SRB Dual-Pass Related	SSPL : 0Y2V	

Panel/Cell Line	Time Zero	Ctrl	Mean Optical Densities (Log10 Concentration)					Percent Growth (Log10 Concentration)					GI50	TGI	LC50
			-8.0	-7.0	-6.0	-5.0	-4.0	-8.0	-7.0	-6.0	-5.0	-4.0			
**Leukemia**															
CCRF-CEM	0.580	3.110	3.038	3.166	3.043	0.454	0.469	97	102	97	-22	-19	2.50E-6	6.57E-6	> 1.00E-4
HL-60(TB)	0.877	3.308	3.231	3.276	3.015	0.633	0.645	97	99	88	-28	-27	2.13E-6	5.75E-6	> 1.00E-4
K-562	0.214	2.678	2.607	2.696	2.168	0.359	0.273	97	101	79	6	2	2.51E-6	> 1.00E-4	> 1.00E-4
MOLT-4	0.550	2.987	2.991	3.006	2.920	0.411	0.483	100	101	97	-25	-12	2.43E-6	6.21E-6	> 1.00E-4
RPMI-8226	1.128	3.138	2.989	3.071	2.986	1.543	0.814	93	97	92	21	-28	3.90E-6	2.66E-5	> 1.00E-4
SR	0.281	1.723	1.532	1.623	1.514	0.338	0.312	87	93	85	4	2	2.72E-6	> 1.00E-4	> 1.00E-4
**Non-Small Cell Lung Cancer**															
A549/ATCC	0.373	2.307	2.072	2.100	2.188	1.844	0.186	88	89	94	76	-50	1.61E-5	4.01E-5	9.98E-5
EKVX	0.679	1.985	1.818	1.828	1.801	0.335	0.260	87	88	86	-51	-62	1.83E-6	4.25E-6	9.88E-6
HOP-62	0.451	2.006	1.921	1.967	1.913	0.397	0.129	95	98	94	-12	-72	2.60E-6	7.71E-6	4.35E-5
HOP-92	1.205	1.998	1.788	1.868	1.958	1.154	0.139	73	84	95	-4	-89	2.84E-6	9.06E-6	3.49E-5
NCI-H226	1.465	3.101	3.046	3.087	3.096	0.696	0.715	97	99	100	-52	-51	2.12E-6	4.52E-6	9.63E-6
NCI-H23	0.538	1.702	1.673	1.658	1.568	0.258	0.215	98	96	88	-52	-60	1.88E-6	4.26E-6	9.66E-6
NCI-H322M	0.694	2.020	1.863	1.939	2.031	1.364	-0.012	88	94	101	51	-100	1.01E-5	2.17E-5	4.65E-5
NCI-H460	0.284	3.025	3.112	3.078	2.958	2.088	0.054	103	102	98	66	-81	1.28E-5	2.80E-5	6.14E-5
NCI-H522	0.911	2.813	2.473	2.490	2.402	0.533	0.518	82	83	78	-42	-43	1.72E-6	4.50E-6	> 1.00E-4
**Colon Cancer**															
COLO 205	0.443	2.016	1.965	2.015	1.874	0.168	0.123	97	100	91	-62	-72	1.85E-6	3.93E-6	8.34E-6
HCC-2998	0.652	2.522	2.418	2.518	2.470	1.065	0.043	94	100	97	22	-93	4.25E-6	1.55E-5	4.21E-5
HCT-116	0.242	2.582	2.337	2.580	2.479	0.150	0.084	89	100	96	-38	-65	2.19E-6	5.18E-6	2.72E-5
HCT-15	0.355	2.616	2.479	2.555	2.440	0.082	0.057	94	97	92	-77	-84	1.78E-6	3.51E-6	6.92E-6
HT29	0.240	2.144	1.987	2.096	2.171	0.574	0.226	92	97	101	18	-6	4.10E-6	5.63E-5	> 1.00E-4
KM12	0.467	2.980	2.902	2.927	2.911	1.028	0.193	97	98	97	22	-59	4.27E-6	1.88E-5	7.79E-5
SW-620	0.200	1.533	1.447	1.459	1.383	0.134	0.041	94	94	89	-33	-80	2.08E-6	5.36E-6	2.32E-5
**CNS Cancer**															
SF-268	0.759	2.576	2.399	2.433	2.388	0.795	0.279	90	92	90	2	-63	2.83E-6	1.07E-5	6.25E-5
SF-295	0.484	1.746	1.572	1.662	1.644	0.655	0.002	86	93	92	14	-100	3.43E-6	1.32E-5	3.64E-5
SF-539	0.884	2.840	2.660	2.710	2.754	0.134	-0.005	91	93	96	-85	-100	1.79E-6	3.39E-6	6.41E-6
SNB-19	0.749	2.687	2.548	2.524	2.542	0.826	-0.013	93	92	93	4	-100	3.02E-6	1.09E-5	3.30E-5
SNB-75	0.890	1.792	1.555	1.633	1.618	0.443	-0.003	74	82	81	-50	-100	1.72E-6	4.13E-6	9.96E-6
U251	0.256	1.738	1.656	1.594	1.639	0.162	0.081	94	90	93	-37	-68	2.15E-6	5.22E-6	2.63E-5
**Melanoma**															
LOX IMVI	0.406	3.043	2.972	3.009	2.897	0.172	0.059	97	99	94	-58	-86	1.96E-6	4.18E-6	8.91E-6
MALME-3M	0.679	1.759	1.664	1.742	1.395	0.005	0.010	91	98	66	-99	-99	1.25E-6	2.51E-6	5.04E-6
M14	0.388	1.827	1.711	1.802	1.767	0.104	0.071	92	98	96	-73	-82	1.87E-6	3.69E-6	7.29E-6
MDA-MB-435	0.443	2.281	2.202	2.266	2.120	0.037	0.027	96	99	91	-92	-94	1.68E-6	3.15E-6	5.91E-6
SK-MEL-2	1.315	3.004	2.915	2.956	2.957	0.637	0.557	95	97	97	-52	-58	2.08E-6	4.50E-6	9.76E-6
SK-MEL-28	0.607	2.348	2.265	2.433	2.557	0.151	.	95	105	112	-75	-100	2.14E-6	3.97E-6	7.34E-6
SK-MEL-5	0.752	3.208	3.145	3.194	3.116	0.024	0.004	97	99	96	-97	-99	1.74E-6	3.15E-6	5.72E-6
UACC-257	1.097	2.653	2.502	2.580	2.580	0.736	0.479	90	95	95	-33	-56	2.26E-6	5.54E-6	5.35E-5
UACC-62	1.057	3.020	2.938	3.040	2.967	0.152	0.152	96	101	97	-86	-86	1.81E-6	3.40E-6	6.39E-6
**Ovarian Cancer**															
IGROV1	0.358	1.916	1.858	1.868	1.891	0.175	0.013	96	97	98	-51	-97	2.11E-6	4.54E-6	9.81E-6
OVCAR-3	0.547	2.169	2.138	2.117	0.603	0.295	0.065	98	97	3	-46	-88	3.17E-7	1.17E-6	1.24E-5
OVCAR-4	0.555	1.333	1.259	1.300	1.157	0.033	-0.002	90	96	77	-94	-100	1.44E-6	2.83E-6	5.53E-6
OVCAR-5	0.528	1.778	1.664	1.694	1.720	0.171	0.094	91	93	95	-68	-82	1.90E-6	3.85E-6	7.80E-6
OVCAR-8	0.418	2.365	2.314	2.350	2.208	0.459	0.168	97	99	92	2	-60	2.93E-6	1.08E-5	6.94E-5
NCI/ADR-RES	0.621	2.279	2.241	2.343	2.223	0.721	0.423	98	104	97	6	-32	3.27E-6	1.44E-5	> 1.00E-4
SK-OV-3	0.927	2.341	2.271	2.277	2.233	2.113	0.608	95	95	92	84	-34	1.93E-5	5.11E-5	> 1.00E-4
**Renal Cancer**															
786-0	0.623	2.647	2.533	2.633	2.678	0.471	0.018	94	99	102	-24	-97	2.56E-6	6.39E-6	2.25E-5
A498	1.828	2.859	2.773	2.777	2.707	2.541	0.099	92	92	85	69	-95	1.31E-5	2.64E-5	5.34E-5
ACHN	0.320	1.787	1.712	1.721	1.696	0.029	-0.015	95	95	94	-91	-100	1.73E-6	3.22E-6	6.00E-6
CAKI-1	0.613	2.558	2.347	2.350	2.422	0.044	0.020	89	89	93	-93	-97	1.70E-6	3.16E-6	5.88E-6
SN12C	0.664	2.704	2.555	2.582	2.640	0.043	-0.001	93	94	97	-94	-100	1.76E-6	3.23E-6	5.90E-6
TK-10	0.733	2.318	2.148	2.184	2.273	0.719	0.111	89	92	97	-2	-85	2.99E-6	9.57E-6	3.80E-5
UO-31	0.630	2.080	1.850	1.880	1.793	0.085	-0.017	84	86	80	-87	-100	1.52E-6	3.03E-6	6.04E-6
**Prostate Cancer**															
PC-3	0.511	2.432	2.310	2.379	2.186	1.065	0.460	94	97	87	29	-10	4.33E-6	5.51E-5	> 1.00E-4
DU-145	0.346	1.777	1.794	1.759	1.741	0.069	0.060	101	99	97	-80	-83	1.85E-6	3.54E-6	6.77E-6
**Breast Cancer**															
MCF7	0.309	1.876	1.757	1.777	1.619	0.089	0.098	92	94	84	-71	-68	1.65E-6	3.46E-6	7.28E-6
MDA-MB-231/ATCC	0.656	1.686	1.612	1.579	1.532	0.458	0.286	93	90	85	-30	-56	2.01E-6	5.46E-6	5.69E-5
HS 578T	0.915	2.023	1.975	1.937	1.890	0.689	0.726	96	92	88	-25	-21	2.17E-6	6.04E-6	> 1.00E-4
BT-549	1.174	2.532	2.317	2.509	2.507	0.065	0.041	84	98	98	-95	-97	1.78E-6	3.23E-6	5.88E-6
T-47D	1.075	2.782	2.571	2.602	2.599	0.886	0.674	88	89	89	-18	-37	2.33E-6	6.85E-6	> 1.00E-4
MDA-MB-468	0.915	1.611	1.519	1.583	1.250	0.436	0.263	87	96	48	-52	-71	9.14E-7	3.01E-6	9.46E-6

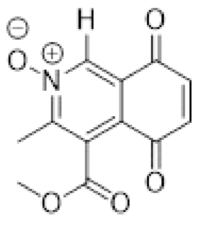

23

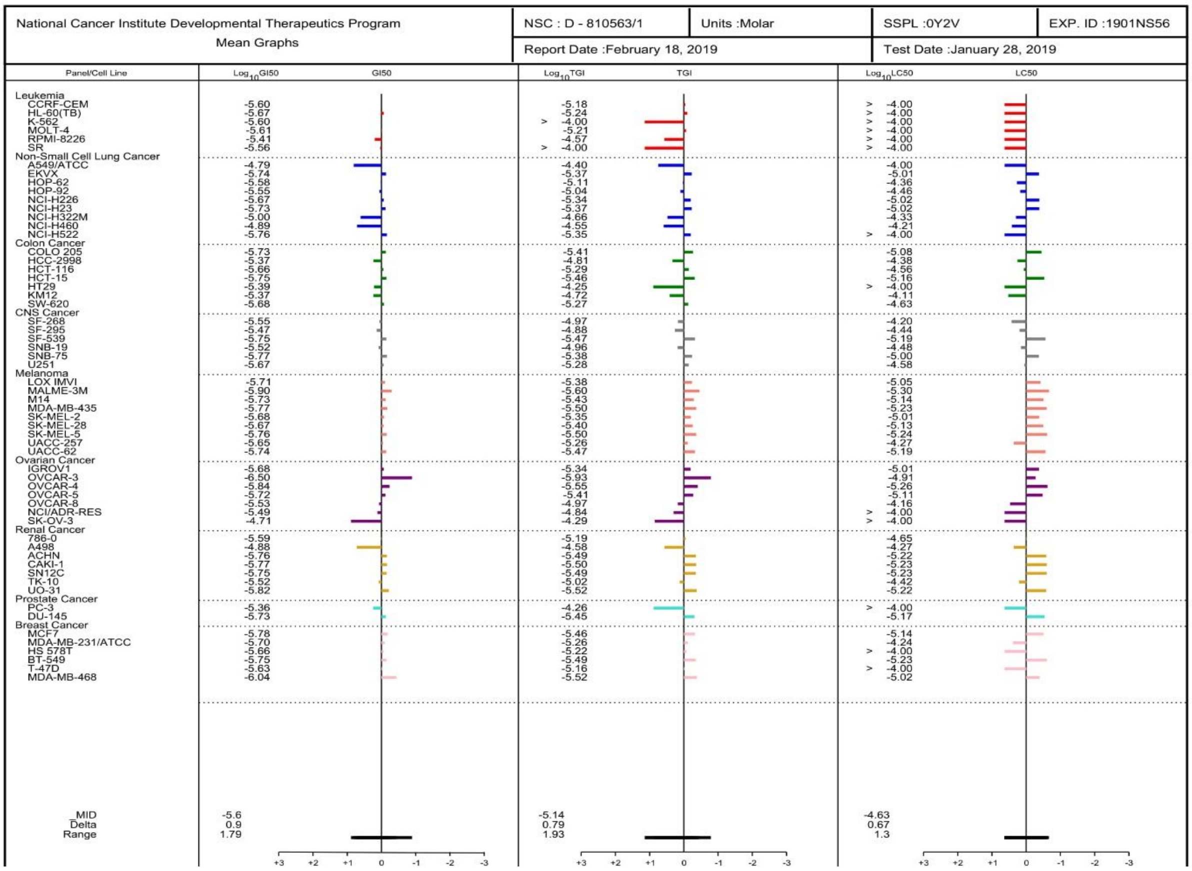

Mean of GI_{50} across 59 cell lines for compound **23** as Log_{10} Concentration (SD): -5.604 (±0.29)

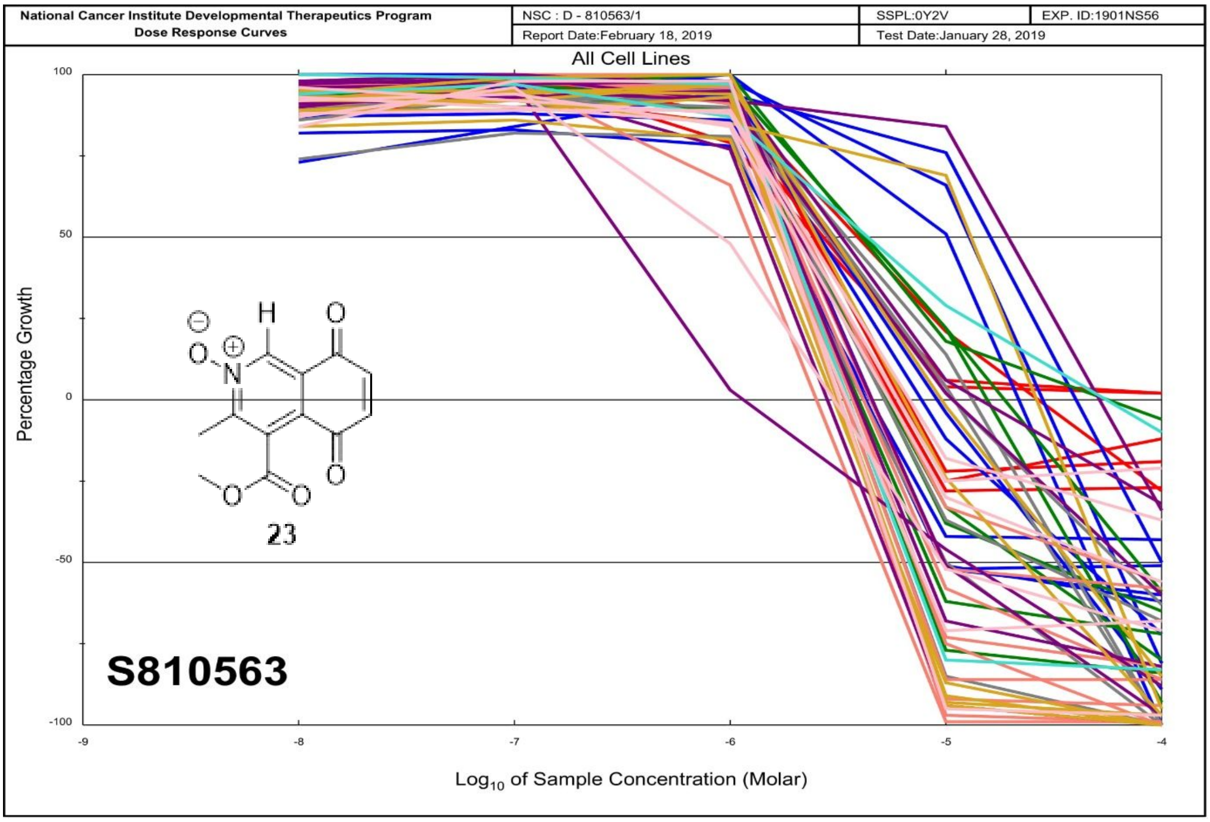

Figure S55** NCI Five Dose data cell line comparison summary for compound **23

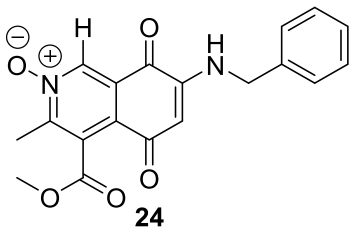

24

Developmental Therapeutics Program One Dose Mean Graph	NSC: D-820594 / 1	Conc: 1.00E-5 Molar	Test Date: Nov 18, 2019
	Experiment ID: 1911OS93		Report Date: Dec 19, 2019

Panel/Cell Line	Growth Percent
**Leukemia**	
CCRF-CEM	2.92
HL-60(TB)	-34.50
K-562	1.41
MOLT-4	-29.42
RPMI-8226	-24.50
SR	2.60
**Non-Small Cell Lung Cancer**	
A549/ATCC	-19.77
EKVX	-45.07
HOP-62	4.14
HOP-92	-29.71
NCI-H226	-9.27
NCI-H23	-34.15
NCI-H322M	-8.65
NCI-H460	-30.27
NCI-H522	-30.45
**Colon Cancer**	
COLO 205	-52.55
HCT-116	1.80
HCT-15	-60.05
HT29	-0.49
KM12	-0.69
SW-620	-18.99
**CNS Cancer**	
SF-268	-13.93
SF-295	-45.31
SF-539	-71.69
SNB-19	-23.76
SNB-75	-97.92
U251	-57.42
**Melanoma**	
LOX IMVI	-30.08
MALME-3M	-93.41
M14	-77.84
MDA-MB-435	-93.70
SK-MEL-2	-44.43
SK-MEL-28	-29.33
SK-MEL-5	-97.05
UACC-257	-81.46
UACC-62	-78.46
**Ovarian Cancer**	
IGROV1	-54.25
OVCAR-3	-35.33
OVCAR-4	-97.05
OVCAR-5	-79.88
OVCAR-8	-2.43
NCI/ADR-RES	-14.11
SK-OV-3	8.10
**Renal Cancer**	
786-0	10.45
A498	-4.89
ACHN	-18.43
CAKI-1	3.84
RXF 393	-26.02
SN12C	-78.85
TK-10	39.00
UO-31	-14.81
**Prostate Cancer**	
PC-3	-11.08
DU-145	-87.06
**Breast Cancer**	
MCF7	-58.26
MDA-MB-231/ATCC	-3.95
HS 578T	-15.31
BT-549	-88.74
T-47D	-31.83
MDA-MB-468	-57.71
Mean	-35.09
Delta	62.83
Range	136.92

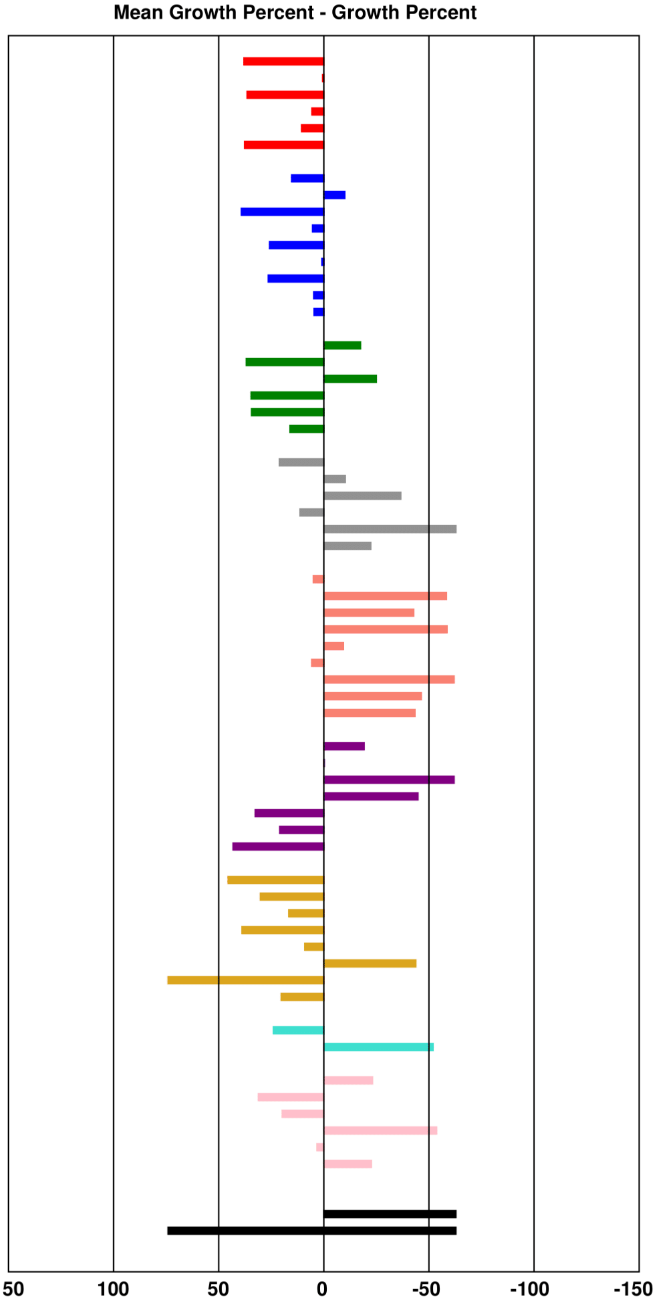

Figure S56 *NCI One Dose (10 µM) data for compound **24***

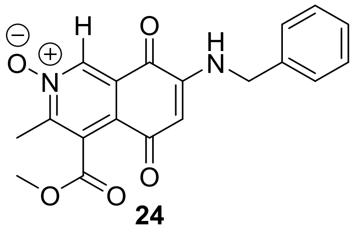

24

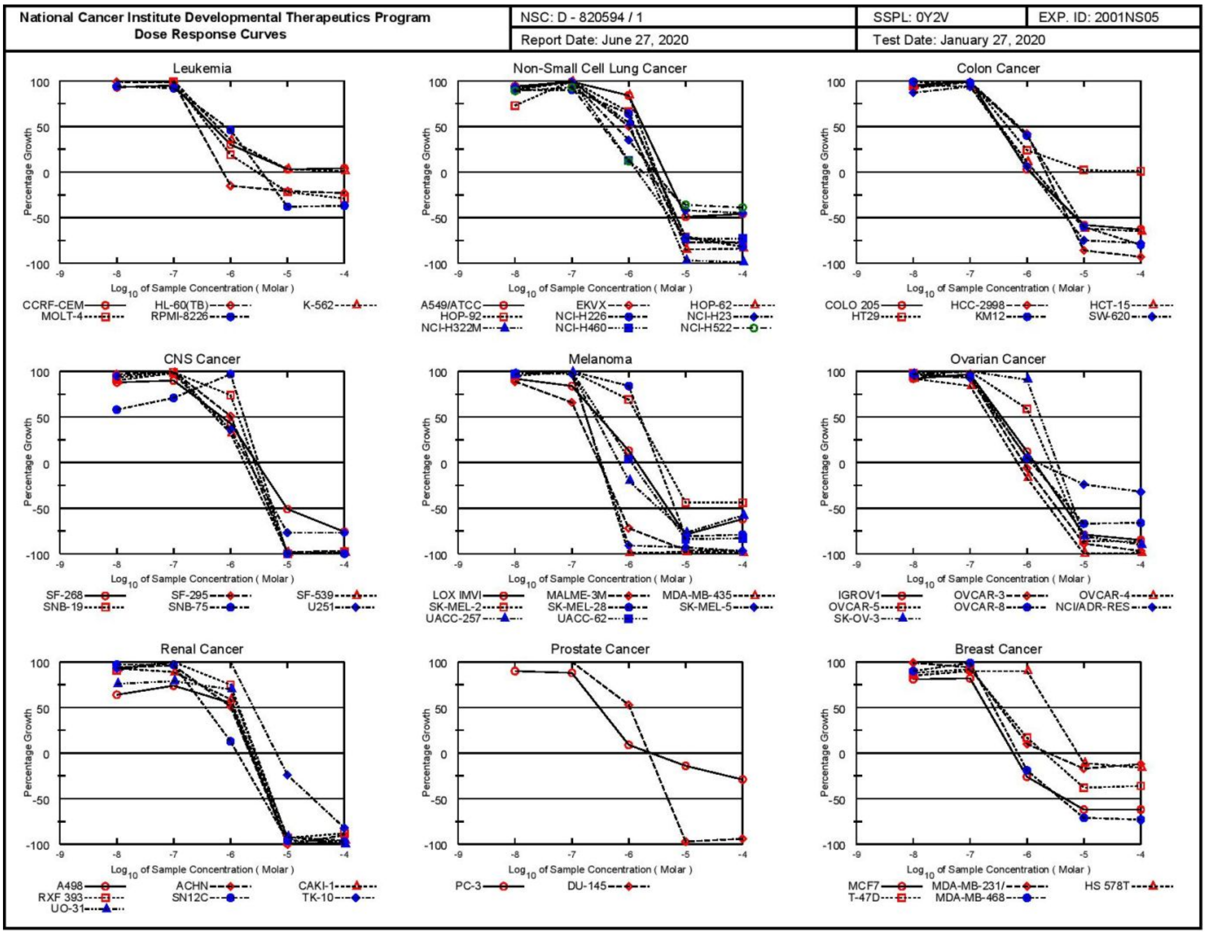

Figure S57 *NCI Five Dose response summary data for compound* ***24***

Table S19** NCI Five Dose raw data for compound **24

National Cancer Institute Developmental Therapeutics Program
In-Vitro Testing Results

NSC : D - 820594 / 1	Experiment ID : 2001NS05	Test Type : 08	Units : Molar
Report Date : June 27, 2020	Test Date : January 27, 2020	QNS :	MC :
COMI : RK 7.0.4	Stain Reagent : SRB Dual-Pass Related	SSPL : 0Y2V	

	Log10 Concentration														
	Time		Mean Optical Densities					Percent Growth							
Panel/Cell Line	Zero	Ctrl	-8.0	-7.0	-6.0	-5.0	-4.0	-8.0	-7.0	-6.0	-5.0	-4.0	GI50	TGI	LC50
Leukemia															
CCRF-CEM	0.435	2.162	2.033	2.077	0.961	0.483	0.500	93	95	30	3	4	4.99E-7	> 1.00E-4	> 1.00E-4
HL-60(TB)	0.661	2.817	2.793	2.765	0.565	0.523	0.508	99	98	-15	-21	-23	2.66E-7	7.42E-7	> 1.00E-4
K-562	0.196	1.638	1.804	1.756	0.697	0.242	0.218	111	108	35	3	1	6.20E-7	> 1.00E-4	> 1.00E-4
MOLT-4	0.835	3.198	3.203	3.184	1.287	0.653	0.592	100	99	19	-22	-29	4.12E-7	2.93E-6	> 1.00E-4
RPMI-8226	0.857	2.313	2.219	2.198	1.534	0.529	0.544	94	92	46	-38	-37	8.38E-7	3.54E-6	> 1.00E-4
Non-Small Cell Lung Cancer															
A549/ATCC	0.949	3.082	2.964	3.052	2.734	0.484	0.515	94	99	84	-49	-46	1.79E-6	4.27E-6	> 1.00E-4
EKVX	0.899	2.116	2.016	2.152	1.510	0.211	0.204	92	103	50	-77	-77	1.00E-6	2.49E-6	6.18E-6
HOP-62	0.823	2.624	2.448	2.598	2.334	0.125	0.132	90	99	84	-85	-84	1.59E-6	3.14E-6	6.21E-6
HOP-92	1.421	2.097	1.914	2.117	1.870	0.415	0.303	73	103	66	-71	-79	1.32E-6	3.05E-6	7.05E-6
NCI-H226	0.750	1.713	1.616	1.616	1.364	0.207	0.133	90	90	64	-72	-82	1.26E-6	2.94E-6	6.84E-6
NCI-H23	0.833	2.268	2.167	2.252	1.331	0.483	0.461	93	99	35	-42	-45	5.78E-7	2.83E-6	> 1.00E-4
NCI-H322M	0.923	2.464	2.321	2.490	1.748	0.028	0.007	91	102	54	-97	-99	1.06E-6	2.27E-6	4.87E-6
NCI-H460	0.258	2.735	2.743	2.783	0.577	0.069	0.070	100	102	13	-73	-73	3.83E-7	1.41E-6	5.37E-6
NCI-H522	1.079	2.959	2.762	2.835	1.296	0.692	0.657	89	93	12	-36	-39	3.39E-7	1.75E-6	> 1.00E-4
Colon Cancer															
COLO 205	0.655	2.906	2.804	2.929	0.713	0.275	0.244	95	101	3	-58	-63	3.30E-7	1.10E-6	7.37E-6
HCC-2998	0.527	2.138	2.017	2.117	1.206	0.075	0.039	92	99	42	-86	-93	7.27E-7	2.14E-6	5.25E-6
HCT-15	0.373	2.483	2.371	2.350	0.603	0.142	0.129	95	94	11	-62	-65	3.37E-7	1.41E-6	6.83E-6
HT29	0.439	2.805	2.653	2.838	1.005	0.482	0.458	94	101	24	2	1	4.61E-7	> 1.00E-4	> 1.00E-4
KM12	0.853	3.326	3.303	3.287	1.832	0.340	0.170	99	98	40	-60	-80	6.65E-7	2.49E-6	7.91E-6
SW-620	0.336	2.302	2.051	2.178	0.463	0.086	0.075	87	94	6	-75	-78	3.17E-7	1.20E-6	4.98E-6
CNS Cancer															
SF-268	1.051	2.869	2.659	2.683	1.826	0.519	0.257	88	90	43	-51	-76	6.97E-7	2.86E-6	9.84E-6
SF-295	0.766	3.099	3.020	3.094	1.951	0.017	0.022	97	100	51	-98	-97	1.01E-6	2.20E-6	4.77E-6
SF-539	0.914	2.765	2.578	2.729	1.515	0.005	0.010	90	98	32	-99	-99	5.40E-7	1.76E-6	4.22E-6
SNB-19	0.621	2.153	2.048	2.141	1.762	-0.003	0.010	93	99	74	-100	-98	1.38E-6	2.67E-6	5.17E-6
SNB-75	1.678	2.475	2.137	2.241	2.453	0.001	0.003	58	71	97	-100	-100	1.74E-6	3.11E-6	5.58E-6
U251	0.659	2.779	2.673	2.814	1.416	0.152	0.155	95	102	36	-77	-77	6.07E-7	2.07E-6	5.77E-6
Melanoma															
LOX IMVI	0.438	3.070	2.862	2.655	0.775	0.094	0.169	92	84	13	-79	-62	3.01E-7	1.38E-6	4.86E-6
MALME-3M	0.917	2.242	2.102	1.787	0.255	0.040	0.028	89	66	-72	-96	-97	1.30E-7	2.99E-7	6.90E-7
MDA-MB-435	0.874	3.319	3.314	3.324	0.010	0.017	0.012	100	100	-99	-98	-99	1.79E-7	3.19E-7	5.68E-7
SK-MEL-2	1.256	2.936	2.850	2.951	2.414	0.698	0.698	95	101	69	-44	-44	1.47E-6	4.05E-6	> 1.00E-4
SK-MEL-28	0.831	2.582	2.630	2.855	2.309	0.159	0.175	103	116	84	-81	-79	1.61E-6	3.24E-6	6.50E-6
SK-MEL-5	0.771	3.313	3.275	3.226	0.070	0.054	0.021	98	97	-91	-93	-97	1.77E-7	3.27E-7	6.05E-7
UACC-257	1.224	2.816	2.773	2.793	0.977	0.286	0.516	97	99	-20	-77	-58	2.56E-7	6.76E-7	3.37E-6
UACC-62	0.988	2.837	2.777	2.931	1.063	0.157	0.172	97	105	4	-84	-83	3.51E-7	1.11E-6	4.10E-6
Ovarian Cancer															
IGROV1	0.506	2.246	2.104	2.199	0.709	0.108	0.077	92	97	12	-79	-85	3.57E-7	1.35E-6	4.81E-6
OVCAR-3	0.877	2.553	2.490	2.434	0.822	0.099	0.023	96	93	-6	-89	-97	2.71E-7	8.64E-7	3.39E-6
OVCAR-4	1.006	2.148	2.054	1.961	0.839	0.003	0.009	92	84	-17	-100	-99	2.16E-7	6.82E-7	2.52E-6
OVCAR-5	0.431	1.429	1.407	1.475	1.023	0.062	0.054	98	105	59	-86	-87	1.16E-6	2.57E-6	5.68E-6
OVCAR-8	0.600	2.610	2.555	2.505	0.702	0.198	0.204	97	95	5	-67	-66	3.15E-7	1.17E-6	5.80E-6
NCI/ADR-RES	0.576	2.173	2.118	2.065	0.661	0.436	0.395	97	93	5	-24	-32	3.10E-7	1.51E-6	> 1.00E-4
SK-OV-3	0.951	2.633	2.591	2.703	2.484	0.185	0.098	97	104	91	-81	-90	1.74E-6	3.39E-6	6.64E-6
Renal Cancer															
A498	2.141	2.905	2.628	2.705	2.558	0.017	0.031	64	74	55	-99	-99	1.07E-6	2.26E-6	4.79E-6
ACHN	0.327	1.622	1.547	1.564	0.993	0.002	0.033	94	96	51	-100	-90	1.02E-6	2.19E-6	4.70E-6
CAKI-1	1.085	2.946	2.808	2.739	2.184	0.083	0.040	93	89	59	-92	-96	1.15E-6	2.45E-6	5.25E-6
RXF 393	1.369	2.225	2.148	2.232	2.009	0.099	0.166	91	101	75	-93	-88	1.41E-6	2.79E-6	5.56E-6
SN12C	0.489	2.244	2.200	2.192	0.717	0.020	0.013	97	97	13	-96	-97	3.63E-7	1.32E-6	3.79E-6
TK-10	0.899	2.169	2.074	2.215	2.480	0.687	0.158	93	104	124	-24	-82	3.18E-6	6.92E-6	2.81E-5
UO-31	0.612	1.814	1.524	1.556	1.451	0.047	0.003	76	79	70	-92	-100	1.32E-6	2.69E-6	5.48E-6
Prostate Cancer															
PC-3	0.753	2.592	2.414	2.371	0.922	0.649	0.534	90	88	9	-14	-29	3.03E-7	2.50E-6	> 1.00E-4
DU-145	0.418	1.814	1.864	1.906	1.156	0.012	0.026	104	107	53	-97	-94	1.04E-6	2.25E-6	4.85E-6
Breast Cancer															
MCF7	0.732	2.647	2.292	2.305	0.542	0.279	0.280	81	82	-26	-62	-62	1.98E-7	5.75E-7	4.66E-6
MDA-MB-231/ATCC	0.607	1.616	1.604	1.558	0.712	0.506	0.532	99	94	10	-17	-12	3.37E-7	2.42E-6	> 1.00E-4
HS 578T	1.654	2.795	2.619	2.684	2.686	1.471	1.391	85	90	90	-11	-16	2.50E-6	7.77E-6	> 1.00E-4
T-47D	0.914	2.442	2.258	2.326	1.180	0.564	0.587	88	92	17	-38	-36	3.67E-7	2.05E-6	> 1.00E-4
MDA-MB-468	0.960	2.122	2.010	2.115	0.779	0.283	0.263	90	99	-19	-71	-73	2.62E-7	6.93E-7	4.01E-6

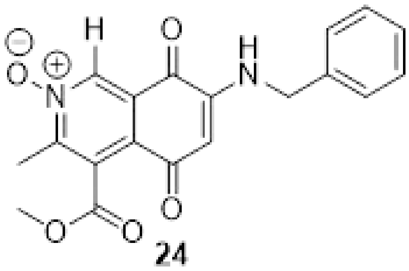

24

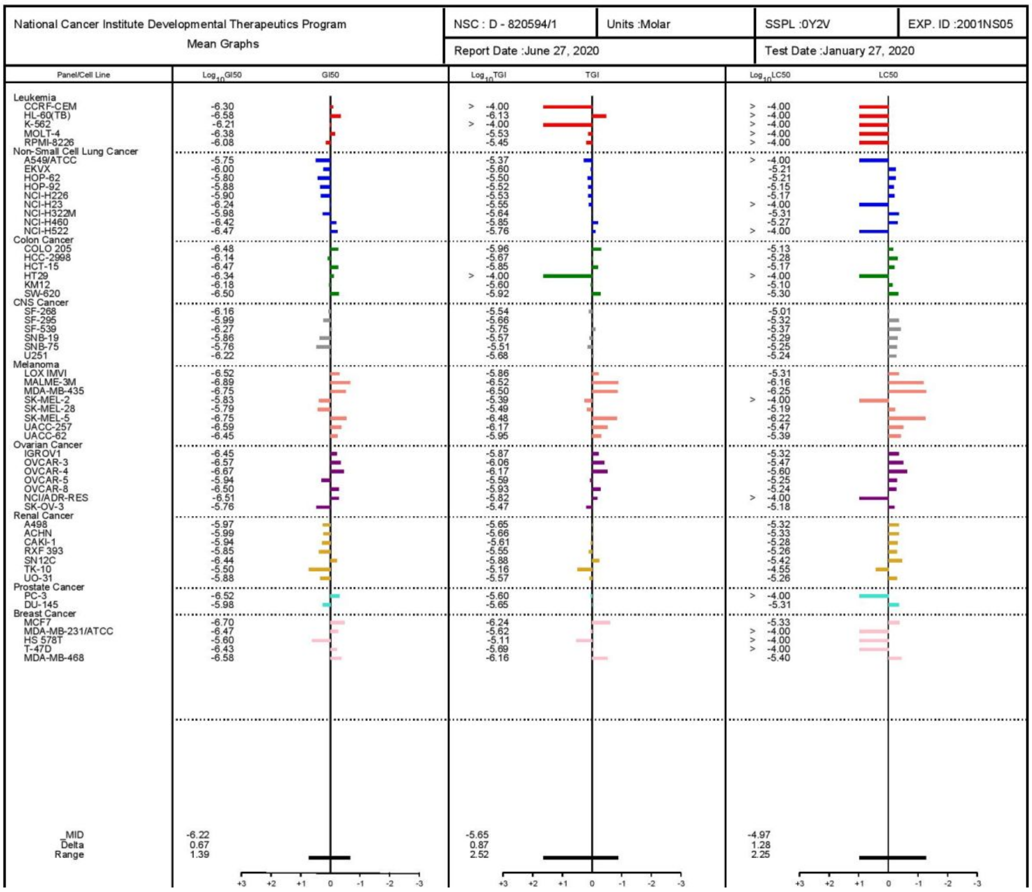

Mean of GI_{50} across 58 cell lines for compound **24** as Log_{10} Concentration (SD): -6.237 (±0.34)

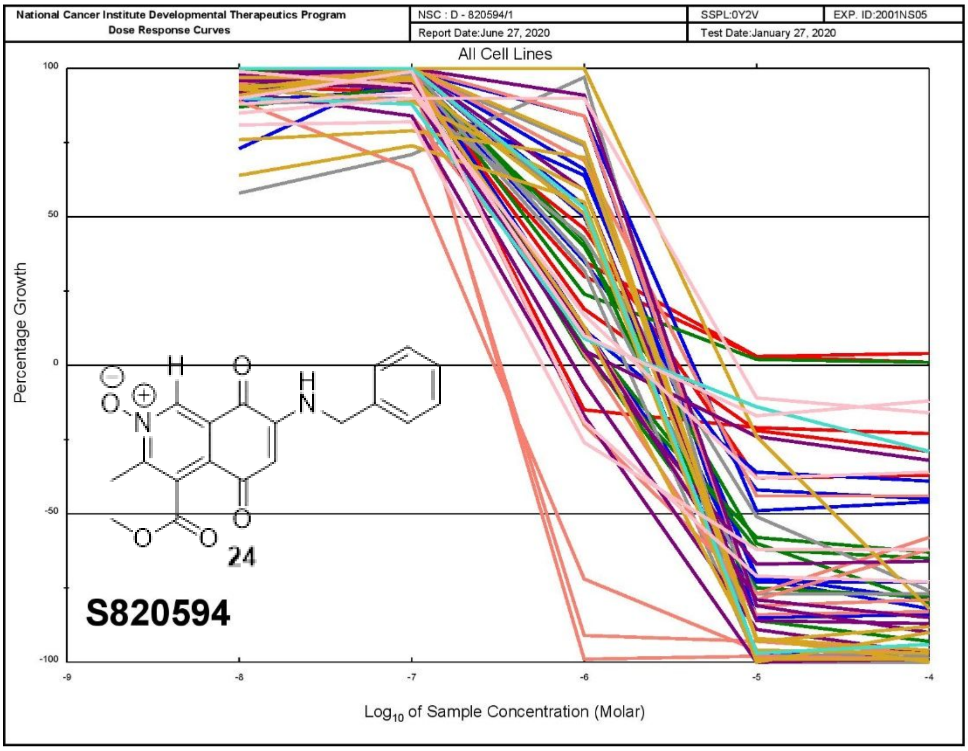

Figure S58 *NCI Five Dose data cell line comparison summary for compound **24***

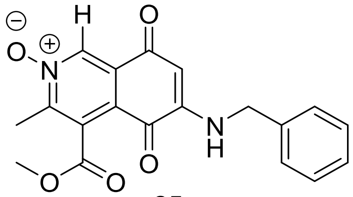

25

Developmental Therapeutics Program

One Dose Mean Graph

**NSC:** D-810564 / 1	**Conc:** 1.00E-5 Molar	**Test Date:** Dec 03, 2018
**Experiment ID:** 1812OS40		**Report Date:** Dec 20, 2018

Panel/Cell Line	Growth Percent
Leukemia	
CCRF-CEM	0.67
HL-60(TB)	-49.38
K-562	-28.99
MOLT-4	-44.29
RPMI-8226	-22.59
SR	-10.25
Non-Small Cell Lung Cancer	
A549/ATCC	-64.18
EKVX	-98.78
HOP-62	-95.33
HOP-92	-74.47
NCI-H226	-90.78
NCI-H23	-39.08
NCI-H322M	-100.00
NCI-H460	-79.77
NCI-H522	-57.94
Colon Cancer	
COLO 205	-73.71
HCC-2998	-89.49
HCT-116	-79.51
HCT-15	-58.81
HT29	0.45
KM12	-83.17
SW-620	-78.95
CNS Cancer	
SF-268	-51.57
SF-295	-93.66
SF-539	-99.04
SNB-19	-99.39
SNB-75	-97.27
U251	-93.14
Melanoma	
LOX IMVI	-74.57
MALME-3M	-100.00
M14	-92.19
MDA-MB-435	-86.50
SK-MEL-2	-70.85
SK-MEL-28	-71.37
SK-MEL-5	-100.00
UACC-257	-92.24
UACC-62	-81.30
Ovarian Cancer	
IGROV1	-66.87
OVCAR-3	-68.72
OVCAR-4	-98.78
OVCAR-5	-90.03
OVCAR-8	-60.37
NCI/ADR-RES	-12.07
SK-OV-3	-87.62
Renal Cancer	
786-0	-68.98
A498	-99.34
ACHN	-89.70
CAKI-1	-98.17
RXF 393	-94.15
SN12C	-91.55
TK-10	-82.42
UO-31	-99.97
Prostate Cancer	
PC-3	-10.78
DU-145	-94.69
Breast Cancer	
MCF7	-57.34
MDA-MB-231/ATCC	-17.69
HS 578T	-15.47
BT-549	-87.26
T-47D	-47.72
MDA-MB-468	-74.88
Mean	-70.60
Delta	29.40
Range	100.67

Mean Growth Percent - Growth Percent

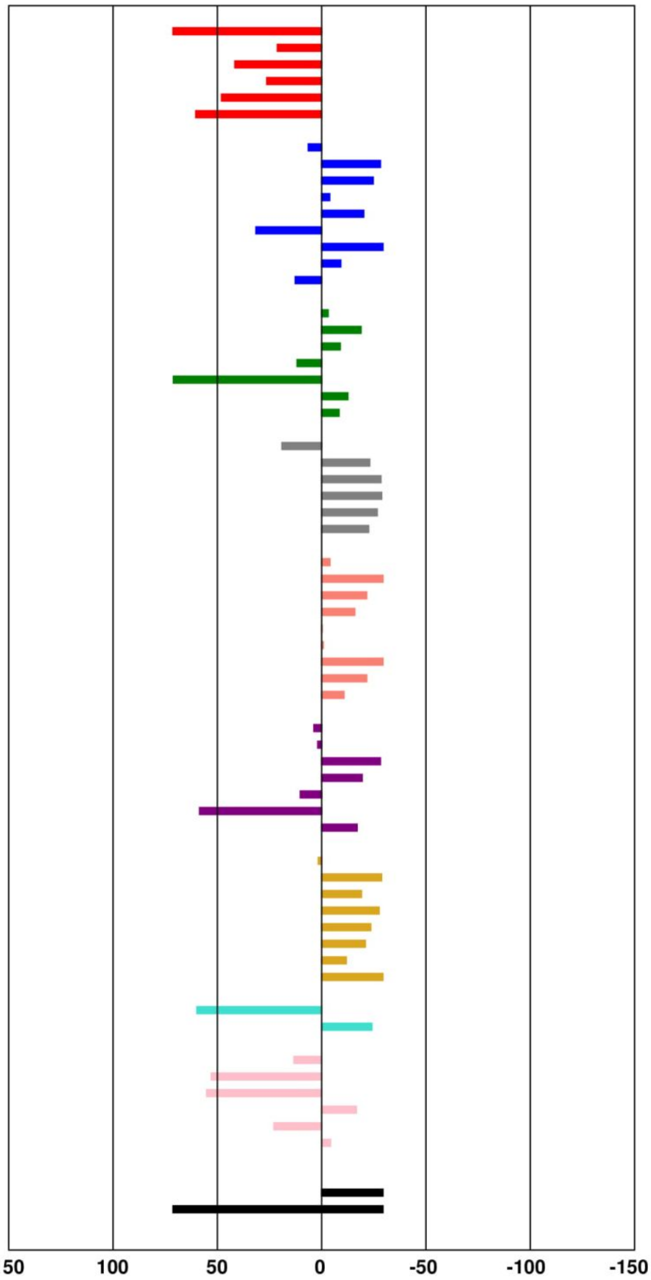

Figure S59 *NCI One Dose (10 µM) data for compound* ***25***

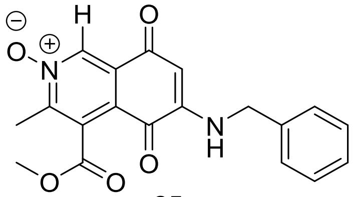

25

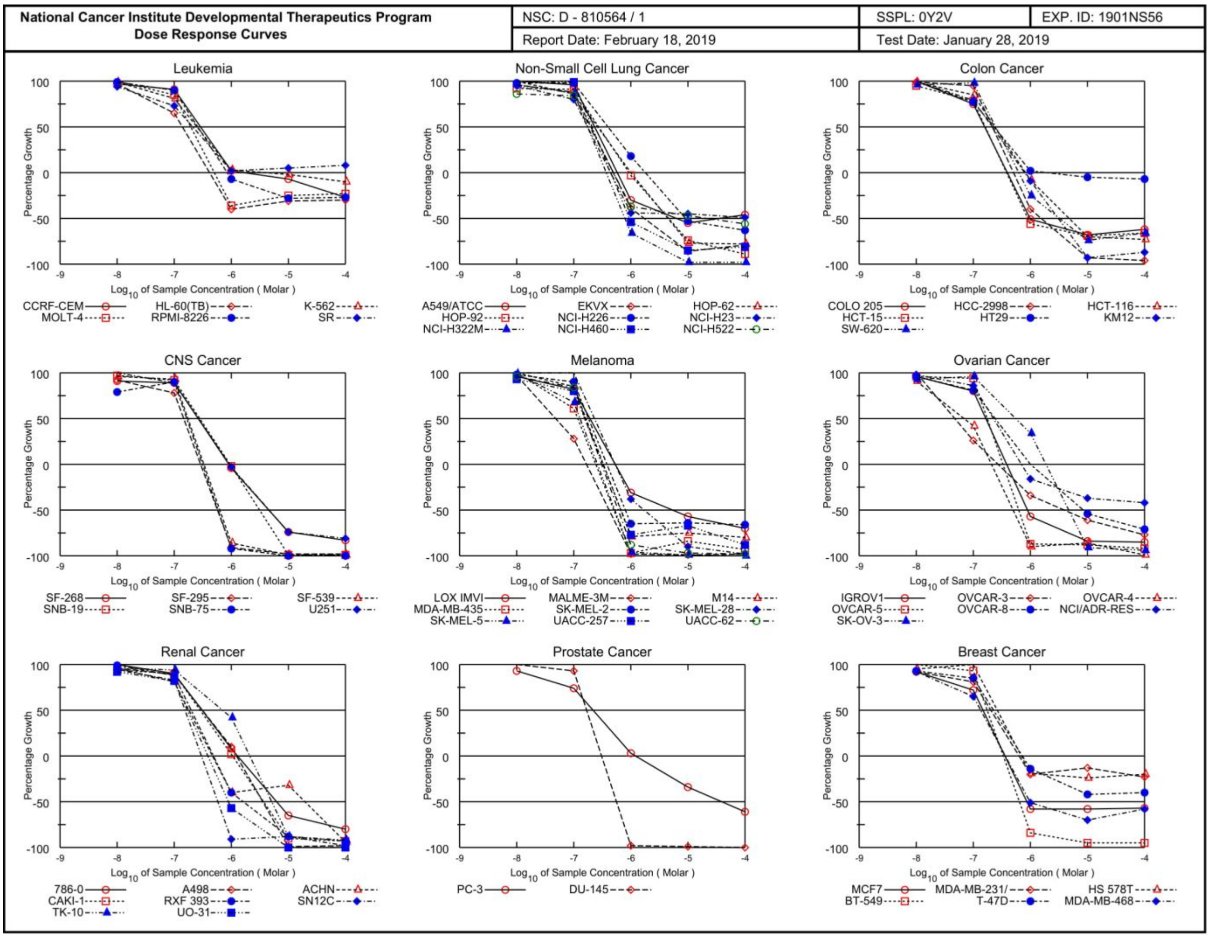

Figure S60 *NCI Five Dose response summary data for compound* ***25***

Table S20** NCI Five Dose raw data for compound **25

National Cancer Institute Developmental Therapeutics Program
In-Vitro Testing Results

NSC : D - 810564 / 1	Experiment ID : 1901NS56	Test Type : 08	Units : Molar
Report Date : February 18, 2019	Test Date : January 28, 2019	QNS :	MC :
COMI : RK 6.3.5	Stain Reagent : SRB Dual-Pass Related	SSPL : 0Y2V	

Log10 Concentration

Panel/Cell Line	Time Zero	Ctrl	Mean Optical Densities -8.0	-7.0	-6.0	-5.0	-4.0	Percent Growth -8.0	-7.0	-6.0	-5.0	-4.0	GI50	TGI	LC50
**Leukemia**															
CCRF-CEM	0.580	3.058	2.991	2.823	0.621	0.542	0.425	97	91	2	-7	-27	2.86E-7	1.58E-6	> 1.00E-4
HL-60(TB)	0.877	3.240	3.179	2.403	0.523	0.610	0.618	97	65	-40	-31	-30	1.38E-7	4.12E-7	> 1.00E-4
K-562	0.214	2.531	2.513	2.093	0.272	0.211	0.193	99	81	3	-2	-10	2.49E-7	4.03E-6	> 1.00E-4
MOLT-4	0.550	2.764	2.827	2.435	0.350	0.411	0.424	103	85	-36	-25	-23	1.95E-7	5.02E-7	> 1.00E-4
RPMI-8226	1.128	3.122	3.108	2.923	1.047	0.811	0.825	99	90	-7	-28	-27	2.58E-7	8.43E-7	> 1.00E-4
SR	0.281	1.570	1.498	1.227	0.304	0.350	0.381	94	73	2	5	8	2.12E-7	> 1.00E-4	> 1.00E-4
**Non-Small Cell Lung Cancer**															
A549/ATCC	0.373	2.413	2.407	2.337	0.260	0.169	0.202	100	96	-30	-55	-46	2.32E-7	5.75E-7	.
EKVX	0.679	2.006	1.959	1.848	0.417	0.097	0.144	96	88	-39	-86	-79	2.00E-7	4.95E-7	1.74E-6
HOP-62	0.451	1.843	1.887	1.642	0.450	0.103	0.100	103	86	.	-77	-78	2.59E-7	9.91E-7	4.43E-6
HOP-92	1.205	1.993	1.930	1.919	1.168	0.310	0.133	92	91	-3	-74	-89	2.71E-7	9.27E-7	4.56E-6
NCI-H226	1.465	3.109	3.070	3.081	1.754	0.693	0.539	98	98	18	-53	-63	3.97E-7	1.78E-6	9.15E-6
NCI-H23	0.538	1.765	1.705	1.523	0.300	0.298	0.273	95	80	-44	-45	-49	1.75E-7	4.41E-7	> 1.00E-4
NCI-H322M	0.694	2.103	2.043	1.920	0.234	0.016	0.012	96	87	-66	-98	-98	1.74E-7	3.69E-7	7.82E-7
NCI-H460	0.284	2.979	3.105	2.961	0.131	0.044	0.054	105	99	-54	-85	-81	2.10E-7	4.44E-7	9.41E-7
NCI-H522	0.911	2.810	2.547	2.507	0.574	0.485	0.404	86	84	-37	-47	-56	1.91E-7	4.94E-7	2.29E-5
**Colon Cancer**															
COLO 205	0.443	1.974	2.006	1.584	0.217	0.140	0.167	102	75	-51	-68	-62	1.57E-7	3.92E-7	9.82E-7
HCC-2998	0.652	2.565	2.537	2.478	0.393	0.043	0.027	99	95	-40	-93	-96	2.17E-7	5.08E-7	1.55E-6
HCT-116	0.242	2.362	2.333	2.033	0.222	0.073	0.066	99	85	-8	-70	-73	2.35E-7	8.15E-7	4.76E-6
HCT-15	0.355	2.663	2.542	2.155	0.156	0.110	0.119	95	78	-56	-69	-66	1.62E-7	3.82E-7	9.01E-7
HT29	0.240	2.060	2.111	1.633	0.269	0.228	0.224	103	77	2	-5	-7	2.26E-7	1.72E-6	> 1.00E-4
KM12	0.467	2.825	2.827	2.337	0.427	0.031	0.063	100	79	-9	-93	-87	2.16E-7	7.99E-7	3.08E-6
SW-620	0.200	1.459	1.411	1.439	0.150	0.052	0.069	96	98	-25	-74	-66	2.47E-7	6.27E-7	3.22E-6
**CNS Cancer**															
SF-268	0.759	2.444	2.300	2.264	0.731	0.201	0.130	91	89	-4	-74	-83	2.65E-7	9.11E-7	4.60E-6
SF-295	0.484	1.729	1.629	1.451	0.046	0.012	0.009	92	78	-91	-98	-98	1.46E-7	2.90E-7	5.74E-7
SF-539	0.884	2.770	2.702	2.633	0.120	0.007	0.007	96	93	-86	-99	-99	1.73E-7	3.29E-7	6.26E-7
SNB-19	0.749	2.678	2.628	2.531	0.735	0.005	0.005	97	92	-2	-99	-99	2.81E-7	9.54E-7	3.12E-6
SNB-75	0.890	1.703	1.531	1.620	0.072	0.002	0.001	79	90	-92	-100	-100	1.66E-7	3.12E-7	5.88E-7
U251	0.256	1.719	1.730	1.560	0.248	0.068	0.048	101	89	-3	-74	-81	2.66E-7	9.25E-7	4.62E-6
**Melanoma**															
LOX IMVI	0.406	3.053	2.957	2.581	0.280	0.175	0.121	96	82	-31	-57	-70	1.92E-7	5.31E-7	5.35E-6
MALME-3M	0.679	1.776	1.744	0.984	0.014	0.009	0.011	97	28	-98	-99	-98	4.77E-8	1.66E-7	4.16E-7
M14	0.388	1.824	1.865	1.603	0.081	0.098	0.077	103	85	-79	-75	-80	1.63E-7	3.29E-7	6.64E-7
MDA-MB-435	0.443	2.226	2.274	1.526	0.015	0.073	0.030	103	61	-97	-84	-93	1.17E-7	2.43E-7	5.06E-7
SK-MEL-2	1.315	2.961	2.921	2.801	0.457	0.474	0.442	98	90	-65	-64	-66	1.81E-7	3.80E-7	7.97E-7
SK-MEL-28	0.607	2.331	2.347	2.329	0.376	0.059	0.010	101	100	-38	-90	-98	2.30E-7	5.30E-7	1.69E-6
SK-MEL-5	0.752	3.242	3.216	2.437	0.031	0.007	0.004	99	68	-96	-99	-100	1.28E-7	2.59E-7	5.24E-7
UACC-257	1.097	2.736	2.618	2.405	0.247	0.367	0.135	93	80	-77	-67	-88	1.55E-7	3.22E-7	6.69E-7
UACC-62	1.057	3.140	3.063	2.799	0.127	0.035	0.026	96	84	-88	-97	-98	1.57E-7	3.07E-7	6.00E-7
**Ovarian Cancer**															
IGROV1	0.358	1.965	1.894	1.645	0.153	0.057	0.053	96	80	-57	-84	-85	1.66E-7	3.82E-7	8.83E-7
OVCAR-3	0.547	2.024	2.048	0.930	0.360	0.211	0.128	102	26	-34	-61	-77	4.81E-8	2.69E-7	3.79E-6
OVCAR-4	0.555	1.277	1.209	0.859	0.058	0.078	0.004	91	42	-90	-86	-99	6.88E-8	2.09E-7	5.00E-7
OVCAR-5	0.528	1.777	1.723	1.702	0.068	0.064	0.042	96	94	-87	-88	-92	1.75E-7	3.30E-7	6.23E-7
OVCAR-8	0.418	2.285	2.216	1.925	0.415	0.193	0.121	96	81	.	-54	-71	2.38E-7	9.77E-7	8.47E-6
NCI/ADR-RES	0.621	2.308	2.278	2.078	0.523	0.389	0.360	98	86	-16	-37	-42	2.27E-7	7.01E-7	> 1.00E-4
SK-OV-3	0.927	2.231	2.142	2.181	1.374	0.086	0.060	93	96	34	-91	-94	5.56E-7	1.88E-6	4.72E-6
**Renal Cancer**															
786-0	0.623	2.683	2.715	2.467	0.787	0.215	0.127	102	90	8	-65	-80	3.05E-7	1.28E-6	6.15E-6
A498	1.828	2.814	2.779	2.701	1.923	0.025	0.028	96	89	10	-99	-98	3.08E-7	1.23E-6	3.55E-6
ACHN	0.320	1.703	1.652	1.445	0.192	0.218	0.020	96	81	-40	-32	-94	1.81E-7	4.67E-7	1.95E-5
CAKI-1	0.613	2.457	2.356	2.280	0.645	0.064	0.040	94	90	2	-90	-93	2.85E-7	1.04E-6	3.68E-6
RXF 393	1.197	1.713	1.708	1.650	0.720	0.142	0.080	99	88	-40	-88	-93	1.98E-7	4.87E-7	1.62E-6
SN12C	0.664	2.702	2.583	2.360	0.061	0.078	0.017	94	83	-91	-88	-98	1.55E-7	3.01E-7	5.83E-7
TK-10	0.733	2.212	2.153	2.118	1.349	0.087	0.059	96	94	42	-88	-92	6.90E-7	2.09E-6	5.08E-6
UO-31	0.630	2.023	1.905	1.777	0.269	0.001	0.003	92	82	-57	-100	-100	1.70E-7	3.88E-7	8.85E-7
**Prostate Cancer**															
PC-3	0.511	2.375	2.249	1.895	0.572	0.338	0.201	93	74	3	-34	-61	2.20E-7	1.22E-6	3.98E-5
DU-145	0.346	1.598	1.731	1.509	0.006	0.004	0.001	111	93	-98	-99	-100	1.68E-7	3.06E-7	5.59E-7
**Breast Cancer**															
MCF7	0.309	1.953	1.829	1.488	0.131	0.129	0.132	92	72	-58	-58	-57	1.47E-7	3.58E-7	8.71E-7
MDA-MB-231/ATCC	0.656	1.669	1.590	1.476	0.525	0.570	0.504	92	81	-20	-13	-23	2.02E-7	6.33E-7	> 1.00E-4
HS 578T	0.915	1.891	1.846	1.908	0.742	0.695	0.728	95	102	-19	-24	-20	2.68E-7	6.96E-7	> 1.00E-4
BT-549	1.174	2.493	2.491	2.406	0.182	0.055	0.056	100	93	-84	-95	-95	1.75E-7	3.35E-7	6.40E-7
T-47D	1.075	2.710	2.602	2.462	0.922	0.622	0.646	93	85	-14	-42	-40	2.25E-7	7.18E-7	> 1.00E-4
MDA-MB-468	0.915	1.680	1.621	1.411	0.446	0.273	0.382	92	65	-51	-70	-58	1.34E-7	3.62E-7	9.75E-7

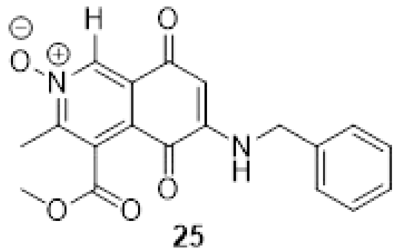

25

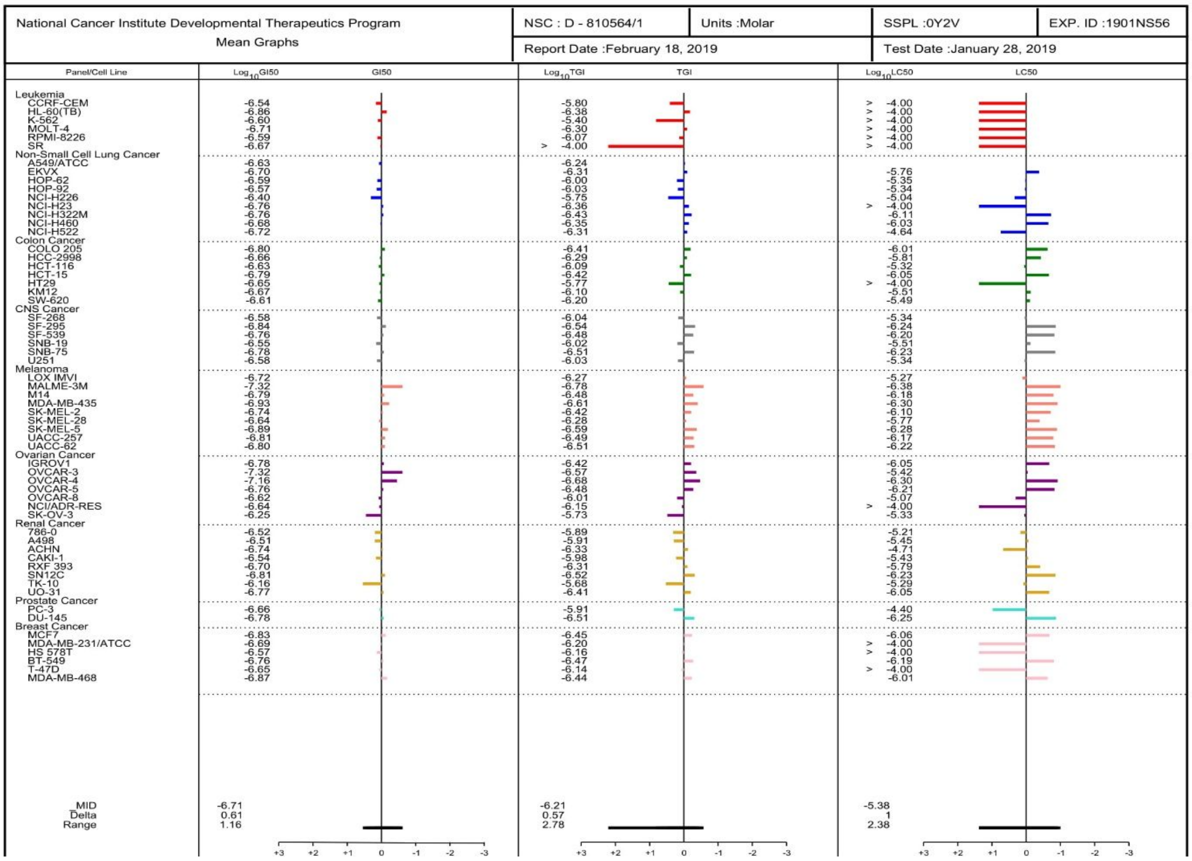

Mean of GI_{50} across 59 cell lines for compound **25** as Log_{10} Concentration (SD): -6.722 (±0.18)

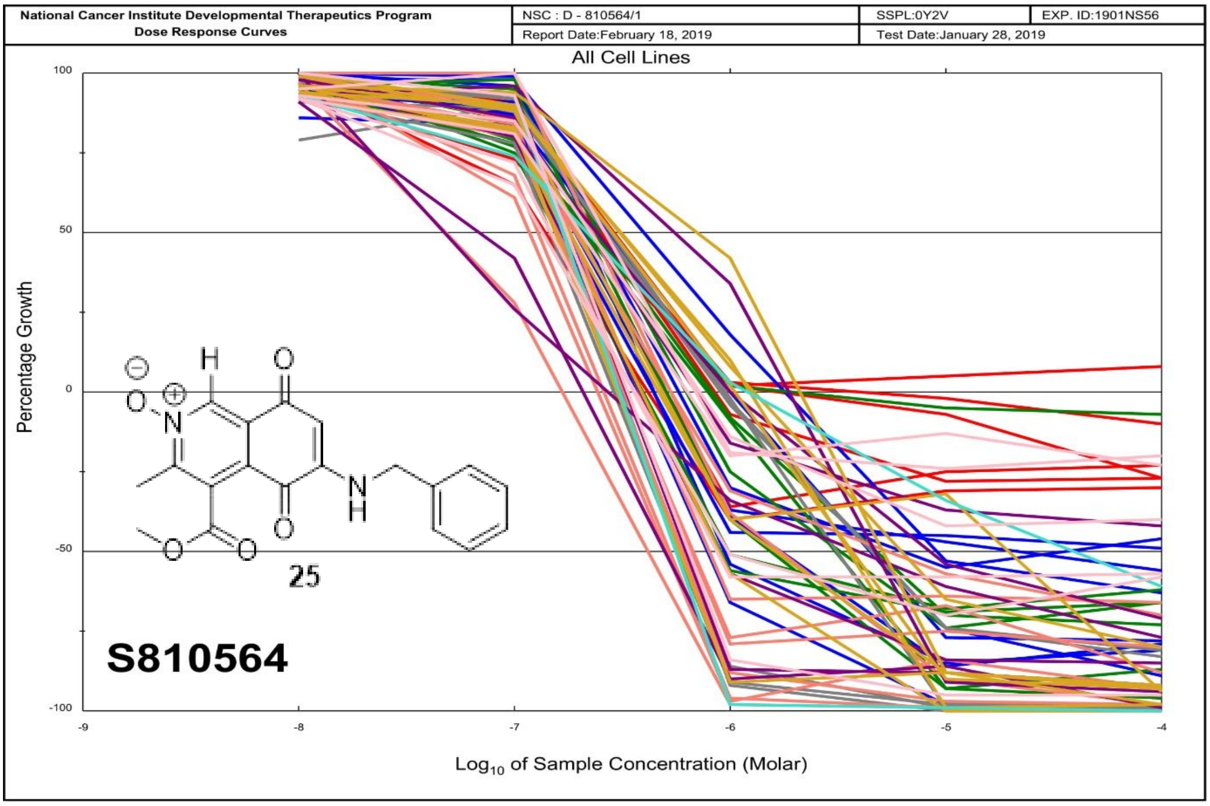

Figure S61 *NCI Five Dose data cell line comparison summary for compound* ***25***

S2.3 Comparison of NCI-H460 cell line GI50‘s

Table S21 *Comparison of NCI-H460 cell line GI_{50}‘s*

	NSCLC NCI-H460 GI_{50} (μM)	
**Compounds**	**IPATIMUP[a]**	**NCI[b]**
**1**	3.06 ± 0.15	2.72
**2**	1.43 ± 0.13	2.74
**3**	8.43 ± 1.41	0.731
**4**	2.70 ± 0.30	0.792
**5**	0.56 ± 0.20	0.525
**20**	6.02 ± 0.34	1.58
**23**	38.02 ± 8.32	12.8
**24**	0.75 ± 0.14	0.383
**25**	0.35 ± 0.02	0.21

[a] Screening was performed using the SRB assay, Results were determined with the SRB assay following 48 h treatment. GI_{50} concentration for each compound was determined by interpolation on the attained concentration–response curves. Results are the mean ± SEM from at least three independent experiments.
[b] NCI data for each compound against NCI-H460 provided for comparison purposes

S3: Growth effects of 25 on parental cells and their P-gp overexpressing MDR pairs.

S3.1 Dose response of NCI-H460/NCI-H460/R NSCLC cell lines to **25** (*Figure S62*)

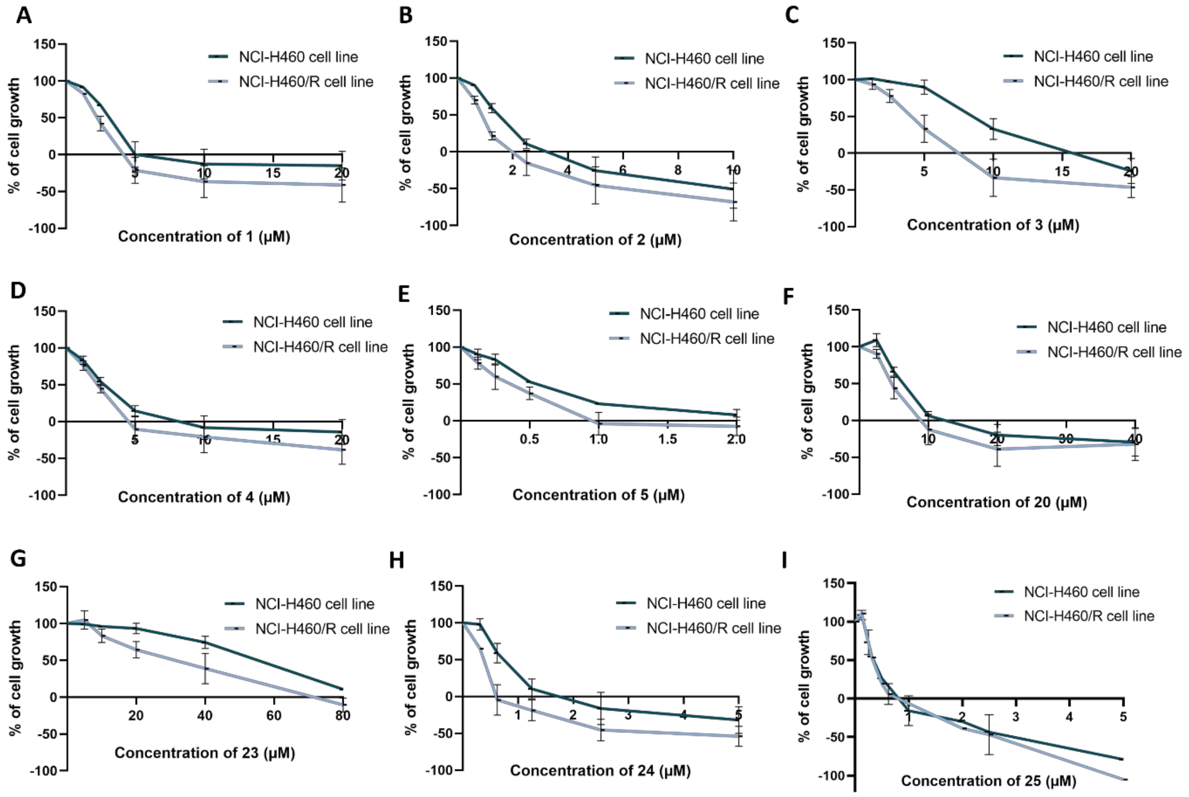

Figure S62 *Concentration – response curves of NCI-H460 (dark blue) and NCI-H460/R (light blue) NSCLC cell lines to the different compounds: (A)* ***1****, (B)* ***2****, (C)* ***3****, (D)* ***4****, (E)* ***5****, (F)* ***20****, (G)* ***23****, (H)* ***24****, (I)* ***25****. Results were determined with the SRB assay following 48 h treatment. Results are presented as percentage (%) of cell growth compared to control cells (vehicle, DMSO). Results are the mean ± SEM from at least three independent experiments.*

S3.2 Dose response of DLD1/DLD1-TxR cancer cell lines to **25** (*Figure S63*)

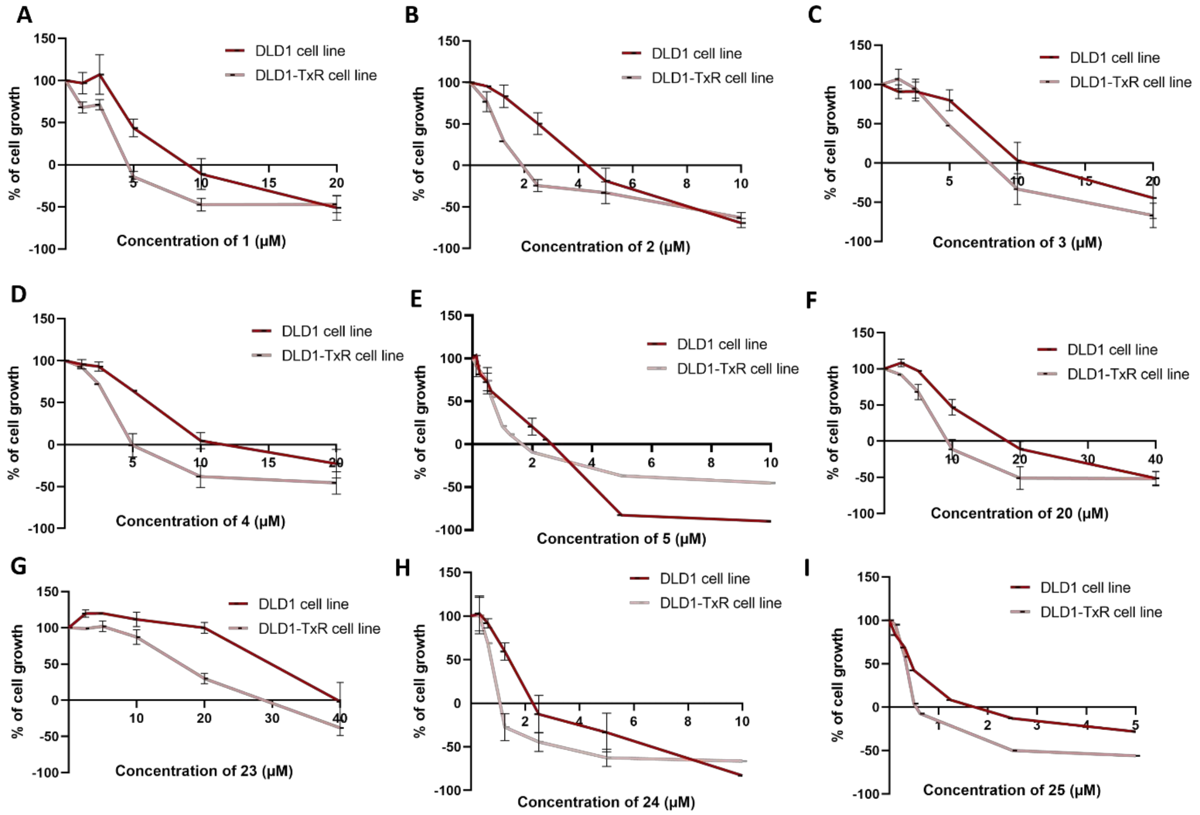

Figure S63 *Concentration – response curves of DLD1 (dark red) and DLD1-TxR (light red) colorectal cancer cell lines to the different compounds: (A)* ***1****, (B)* ***2****, (C)* ***3****, (D)* ***4****, (E)* ***5****, (F)* ***20****, (G)* ***23****, (H)* ***24****, (I)* ***25****. Results were determined with the SRB assay following 48 h of treatment. Results are presented as percentage (%) of cell growth compared to control cells (vehicle, DMSO). Results are the mean ± SEM from at least three independent experiments.*

S3.3 Dose response curves of A549/NCI-H322 NSCLC cell lines to **25** *(Figure S64)*

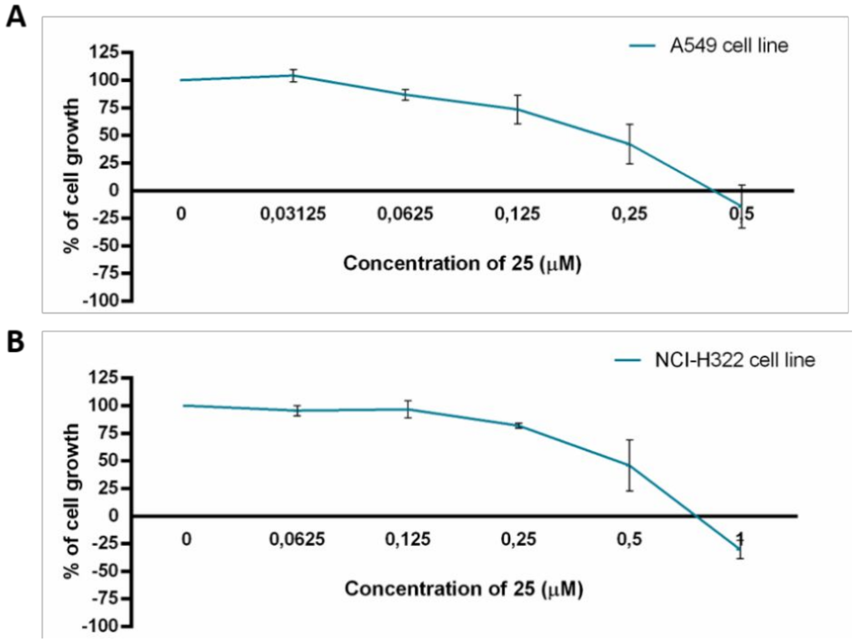

Figure S64 *Concentration – response curves of A549 (A) and NCI-H322 (B) NSCLC cell lines to compound* ***25****. Results were determined with the SRB assay following 48 h treatment. Results are presented as percentage (%) of cell growth compared to control cells (vehicle, DMSO). Results are the mean ± SEM from at least three independent experiments.*

S3.4 Effect of **25** on the viable cell number of NCI-H460 cells (*Figure S65*)

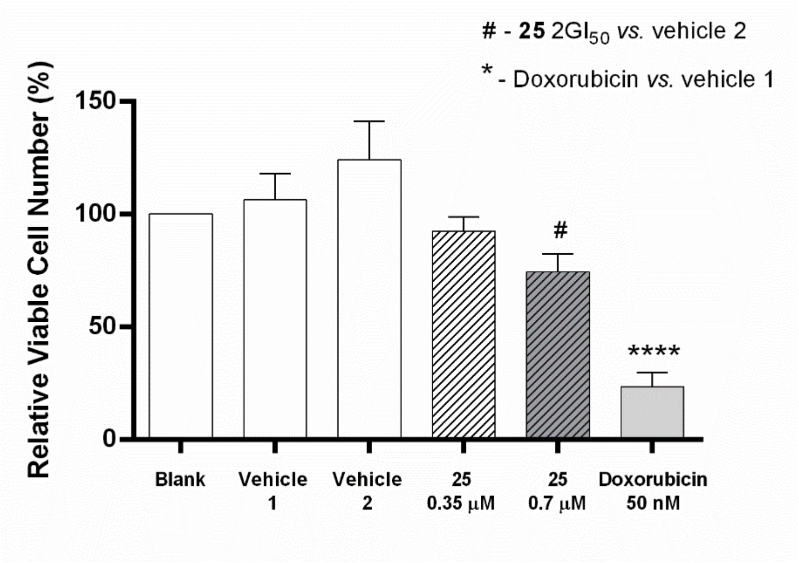

***Figure S65** Effect of **25** on the viable cell number of NCI-H460 cells, determined with the Trypan blue exclusion assay. Cells were incubated for 48 h with medium, DMSO (vehicle 1: % of vehicle used at the GI_{50} concentration of compound; vehicle 2: % of vehicle used at the $2GI_{50}$ concentration of compound), doxorubicin (positive control), GI_{50} concentration (0.35 µM) or the $2GI_{50}$ concentration (0.7 µM) of compound **25**. Results are the mean ± SEM from at least three independent experiments. # $p \leq 0.05$ and **** $p \leq 0.0001$, when comparing DMSO vs. compound treatment.*

S3.5 Effect of **25** on the non-tumorigenic MCF12A cell line (*Figure S66*)

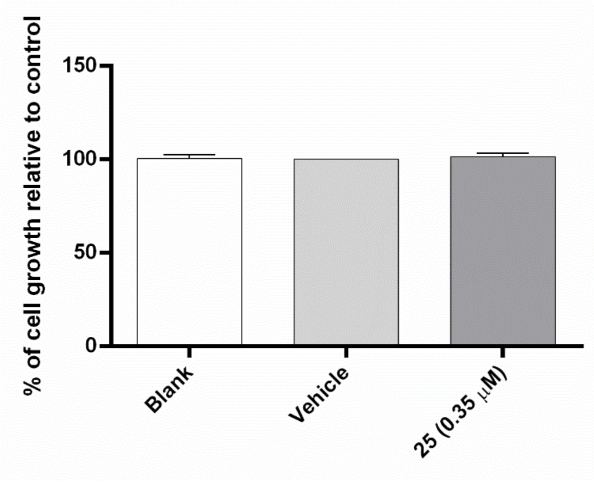

Figure S66 *Cytotoxic effect of compound **25** on the non-tumorigenic MCF12A cell line, analysed by the SRB assay. Cells were treated for 48h with compound **25** at the highest GI_{50} concentration previously obtained in the NCI-H460 and NCI-H460/R pair of counterpart cell lines. Results are presented as the % of cell growth relative to the control cells (treated with the compound vehicle, DMSO at the % used in the GI_{50} concentration of **25**). Data are expressed as mean ± SEM from 3 independent experiments.*

S3.6 Effect of **25** on spheroids from NSCLC and colorectal cancer (*Figure S67*)

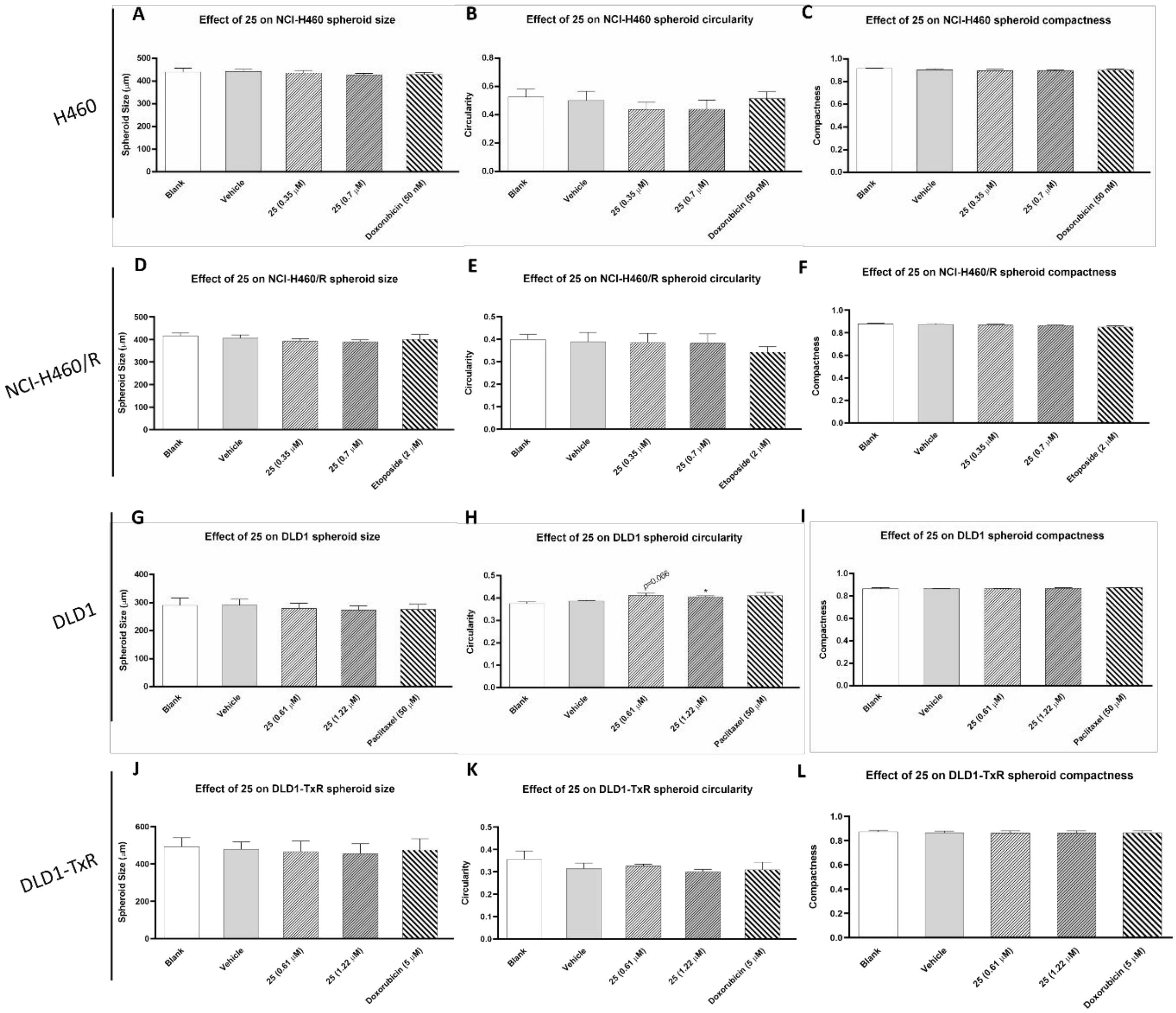

Figure S67 *Effect of **25** on morphological parameters of spheroids from 2 counterpart pairs of sensitive and MDR cell lines, from NSCLC (NCI-H460 and NCI-H460/R) and from colorectal cancer (DLD1 and DLD1-TxR), grown in Microtissues® 3D petri dishes. Effect of **25** on (A) NCI-H460 spheroid size, (B) NCI-H460 spheroid circularity, (C) NCI-H460 spheroid compactness, (D) NCI-H460/R spheroid size, (E) NCI-H460/R spheroid circularity, (F) NCI-H460/R spheroid compactness, (G) DLD1 spheroid size, (H) DLD1 spheroid circularity, (I) DLD1 spheroid compactness, (J) DLD1-TxR spheroid size, (K) DLD1-TxR spheroid circularity, (L) DLD1-TxR spheroid compactness. Spheroids were treated for 48h with medium (blank), DMSO (vehicle at the % used in the highest tested concentration of **25**) or compound **25** at the GI_{50} and $2GI_{50}$ concentrations previously determined in a 2D setting. Doxorubicin (50 nM), Etoposide (2 μM), Paclitaxel (50 μM) and Doxorubicin (5 μM) were used as positive controls for NCI-H460, NCI-H460/R, DLD1 and DLD1-TxR cell lines, respectively. Results are the mean ± SEM from at least three independent experiments. * $p \leq 0.05$ when comparing DMSO vs. compound treatment.*

S4: COMPARE analysis and Predicted physicochemical properties of IQQ *N*-oxides

S4.1 COMPARE Analysis

NCI COMPARE analysis: COMPARE analysis was conducted using the private access system provided by the National Cancer Institute (https://dtp.cancer.gov/databases_tools/compare.htm). Seed compounds were analysed using a number of target sets: synthetic compounds, BEC referral set, mechanistic set, standard agents, marketed drugs and diversity set. While the minimum correlation was set to 0.4, correlations of less than 0.6 were discounted. All other criteria were unchanged. The top 2–4 correlations between all target sets were chosen. Experiments that were carried out at different concentrations to the seed compound were ignored unless the concentration deviated by ±0.1.[7]

S4.2 Predicted physicochemical properties of IQQ *N*-oxides

Table S22 *In silico prediction of IQQ N-oxide lipophilicity*[a]

Compound	Lipophilicity[b]	Solubility	Druglikeness[c]	Pains	Brenk
**1**	1.86	Soluble	Yes	Quinone	*N*-oxide
**2**	1.85	Soluble	Yes	Quinone	*N*-oxide
**3**	0.73	Very soluble	Yes	Quinone	*N*-oxide
**4**	2.15	Soluble	Yes	Quinone	*N*-oxide
**5**	2.16	Soluble	Yes	Quinone	*N*-oxide
**6**	1.85	Soluble	Yes	Quinone	*N*-oxide
**7**	1.87	Soluble	Yes	Quinone	*N*-oxide
**8**	1.88	Soluble	Yes	Quinone	*N*-oxide
**9**	1.86	Soluble	Yes	Quinone	*N*-oxide
**10**	2.37	Moderately soluble	Yes	Quinone	*N*-oxide
**11**	2.38	Moderately soluble	Yes	Quinone	*N*-oxide
**12**	2.38	Moderately soluble	Yes	Quinone	*N*-oxide
**13**	2.38	Moderately soluble	Yes	Quinone	*N*-oxide
**14**	2.47	Moderately soluble	Yes	Quinone	*N*-oxide
**15**	2.47	Moderately soluble	Yes	Quinone	*N*-oxide
**16**	2.45	Soluble	Yes	Quinone	*N*-oxide
**17**	2.46	Soluble	Yes	Quinone	*N*-oxide
**18**	2.67	Moderately soluble	Yes	Quinone	*N*-oxide
**19**	2.68	Moderately soluble	Yes	Quinone	*N*-oxide
**20**	1.43	Soluble	Yes	Quinone	*N*-oxide
**23**	0.35	Very soluble	Yes	Quinone	*N*-oxide
**24**	1.51	Soluble	Yes	Quinone	*N*-oxide
**25**	1.48	Soluble	Yes	Quinone	*N*-oxide

[a] All physicochemical properties of compounds **1**-**25** computed via Swiss ADME.[6]

[b] Lipophilicity assigned by computational Log $P_{o/w}$ (the average of 5 predictions).

[c] As assessed under the rules of Lipinski, Ghose, Veber, Egan and Muegge.

Table S23 In silico ADME parameters of IQQ N-*oxide* ***25***.

Structure	Oral Bioavailability Radar	
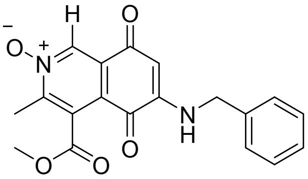 **25**	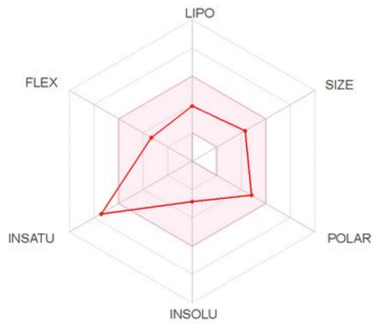	
**Lipophilicity**	Log $P_{o/w}$ (Av. of 5 predictions)	1.48
**Water Solubility**	Log S (Av. of two predictions)	-2.93 (soluble)
**Pharmacokinetics**	GI Absorption	High
	BBB Permeable	No
	P-glycoprotein substrate	No
	CYP (1A2, 2C19, 2C9, 2D6, 3A4) inhibitor	No
**Druglikeness**	Lipinski, Ghose, Veber, Egan, Muegge	Yes (0 violations)
	Abbot Bioavailability Score	0.55
**Medicinal Chem.**	PAINS	1 alert (quinone)
	Brenk	2 alerts (*N*-oxide, quaternary nitrogen)

Red coloured zone suitable for oral bioavailability

Supplementary Information References

1. Valderrama, J. A.; González, M. F.; Pessoa-Mahana, D.; Tapia, R. A.; Fillion, H.; Pautet, F.; Rodriguez, J. A.; Theoduloz, C.; Schmeda-Hirschmann, G. Studies on quinones. Part 41: Synthesis and cytotoxicity of isoquinoline-containing polycyclic quinones. *Bioorganic & Medicinal Chemistry*, **2006**, *14*, 5003-5011.

2. Kruschel, R. D.; Buzid, A.; Khandavilli, U. B. R.; Lawrence, S. E.; Glennon, J.D.; McCarthy, F. O. Isoquinolinequinone N-oxides as anticancer agents effective against drug resistant cell lines. *Org. Biomol. Chem*., **2020**, *18*, 557-568.

3. Delgado, V.; Ibacache, A.; Arancibia, V.; Theoduloz, C.; Valderrama, J. A. Synthesis and in Vitro Antiproliferative Activity of New Phenylaminoisoquinolinequinones against Cancer Cell Lines. *Molecules*, **2013**, *18*, 721-734.

4. Ibacache, A. J.; Delgado, V.; Benites, J.; Theoduloz, C.; Arancibia, V.; Muccioli, G. G.; Valderrama, A. J. Synthesis, Half-Wave Potentials and Antiproliferative Activity of 1-Aryl-substituted Aminoisoquinolinequinones. *Molecules*, **2014**, *19*, 726-739.

5. National Cancer Institute. Developmental Therapeutics Program. NCI-60 Human Tumour Cell Lines Screen. Available online: https://dtp.cancer.gov/discovery_development/nci-60/default.htm (accessed on 11 December 2023).

6. Shoemaker, R. H. The NCI60 human tumour cell line anticancer drug screen. *Nature Reviews Cancer*, **2006**, *6*, 813–823.

7. Daina, A.; Michielin, O.; Zoete, V. SwissADME: a free web tool to evaluate pharmacokinetics, drug-likeness and medicinal chemistry friendliness of small molecules. *Sci. Rep.* **2017**, *7*, 42717.

8. Paull, K. D.; Shoemaker, R. H.; Hodes, L.; Monks, A.; Scudiero, D. A.; Rubinstein, L.; Plowman, J.; Boyd, M. R. Display and analysis of patterns of differential activity of drugs against human tumor cell lines: development of mean graph and COMPARE algorithm. *J. Natl. Cancer Inst.*, **1989**, *81*, 1088–1092.
